# Pembrolizumab followed by irreversible electroporation of a liver metastasis in pancreatic cancer patients

**DOI:** 10.1016/j.isci.2024.111026

**Published:** 2024-09-24

**Authors:** Rasmus Virenfeldt Flak, Emil Kofod-Olsen, Nikolaj Dich Sølvsten, Gintare Naujokaite, Ralf Agger, Mogens Tornby Stender, Signe Christensen, Susy Shim, Laurids Østergaard Poulsen, Sönke Detlefsen, Ole Thorlasius-Ussing, Morten Ladekarl

**Affiliations:** 1 Department of Gastrointestinal Surgery and Clinical Cancer Research Center, Aalborg University Hospital, Aalborg, Denmark; 2 Department of Health Science and Technology, Aalborg University, Aalborg, Denmark; 3 Department of Radiology, Aalborg University Hospital, Aalborg, Denmark; 4 Department of Clinical Medicine, Aalborg University, Aalborg, Denmark; 5 Department of Oncology and Clinical Cancer Research Center, Aalborg University Hospital, Aalborg, Denmark; 6 Department of Pathology, Odense Pancreas Center (OPAC), Odense University Hospital, Odense, Denmark; 7 Department of Clinical Research, Faculty of Health Sciences, University of Southern Denmark, Odense, Denmark

**Keywords:** Health sciences, oncology, clinical finding, clinical stage

## Abstract

Preclinical studies suggest that irreversible electroporation (IRE) increases the effect of immune checkpoint inhibition in pancreatic cancer (PC). Patients with PC received PD-1 inhibitor pembrolizumab and, on day 10, percutaneous IRE of a liver metastasis. Blood samples were analyzed for immune cell subsets and inflammation related proteins. mRNA expression profiling was done in sequential biopsies. Treatment was safe, but the trial was terminated early. The response rate in eight patients was 0% and tumor growth was exponential. A drop in circulating plasmacytoid dendritic cells and a rise in several cytokines and proteins, especially PD-1, after immunotherapy was observed. In liver metastases, immune stimulatory genes were upregulated and immune suppressive genes were downregulated after pembrolizumab, while markers of effector T cells were unchanged. Treatment was safe but showed no efficacy in PC. Immunotherapy induced an immune permissive tumor microenvironment but with no increase in effector cells.

## Highlights

• Anti-PD-1 with IRE was safe but ineffective in liver metastatic pancreatic cancer
• Blood plasmacytoid dendritic cells dropped and plasma PD-1 rose after immunotherapy
• Immune permissive changes to the TME were observed longitudinally
• No changes were found in effector T cells in the circulation or in the TME

## Graphical abstract

**Figure:**
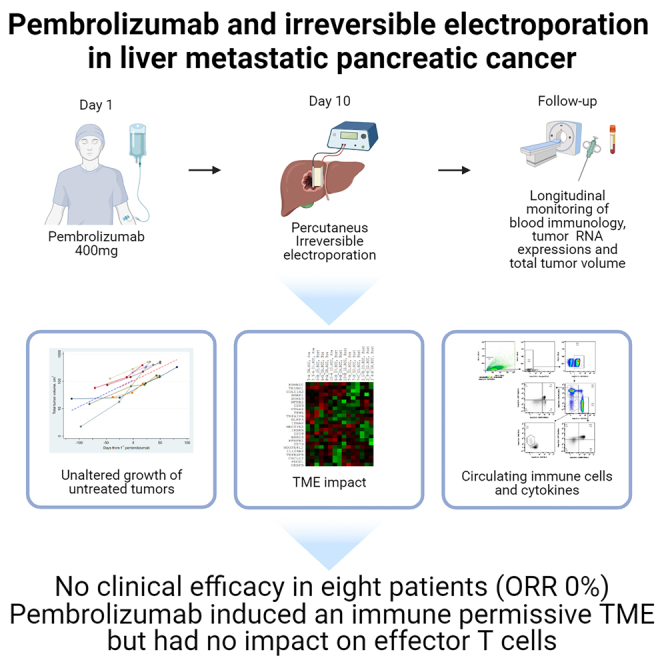

## Introduction

Immune checkpoint inhibitors have changed the treatment paradigms of multiple cancers. Unfortunately, in pancreatic cancer (PC) no survival benefit of immunotherapy has been demonstrated,^1^ and only the rare patients with tumors displaying microsatellite instability or mismatch repair-deficiency seem to respond with high frequencies.^2^

There are several mechanisms potentially contributing to the lack of effect of immunotherapy in PC.^3^ One possible factor is the poor antigenicity of tumors. This is related to the low mutational rate with an average of 45 somatic mutations, which negatively affects the production of neoantigens and immune recognition.^4^ Another unique characteristic of PC is the desmoplastic, immunosuppressive, and hypoxic microenvironment.^3^^,^^5^ This is the result of tumors comprising a mixture of cancer-associated fibroblasts, endothelial cells, extracellular matrix proteins, and immunosuppressive cells including myeloid-derived suppressor cells (MDSCs), regulatory T cells (Tregs), and tumor-associated macrophages (TAMs). In contrast, effector T cells are few.^5^ The low leukocyte infiltration displayed by most pancreatic tumors and its composition is contrasting to many of the tumor types where immunotherapy has shown effect.^4^

It has been hypothesized that immunologically “cold” tumors may be transformed to “hot” by local ablation or irradiation.^3^ Destruction of tumor cells *in situ* may lead to increased presentation of neoantigens to the immune system and the induction of a pro-inflammatory, permissive microenvironment.^6^ In a PC mouse model, anti-PD-L1 therapy combined with radiotherapy significantly increased the CD8/Treg ratio as compared with PD-L1 therapy alone and prevented the formation of liver metastases.^3^ Several clinical trials exploring the potential interactions of radiotherapy and immune checkpoint blockade have been published with most promising results achieved with dual checkpoint inhibition, targeting both PD-1/PD-L1 and CTLA-4.^7^ Further, radiofrequency ablation may also have an abscopal effect. This was investigated in a study of 10 patients with locally advanced PC demonstrating a general activation of the adaptive immune response and a decrease in immunosuppression, lasting for some weeks after the procedure.^8^

First described as a non-thermal clinical ablation modality in 2007,^9^ irreversible electroporation (IRE) is currently being implemented and studied for treatment of locally advanced PC, liver tumors, prostate cancer, kidney cancers, and others. IRE works by applying high-current short duration electrical pulses to the treated tissues. IRE induces cell death by a combination of apoptosis and necrosis with little effect on extracellular matrix by inducing multiple nanoscale pores in the cell membranes. This allows uncontrolled influx and efflux of ions and macromolecules leading to loss of homeostasis and rupture of the cell and thus release of both intracellular and extracellular proteins.^10^^,^^11^

Several preclinical experiments have demonstrated that IRE has strong immunogenic properties that may outperform radiotherapy and radiofrequency ablation.^12^ Through an abscopal effect, IRE may increase the efficacy of concomitant treatment with immune checkpoint inhibitors. In a PC model, mice treated with a combination of IRE and anti-PD-1 had more CD8 cells and a higher CD8/Treg ratio in tumors than mice treated with immune checkpoint inhibitors and radiation or immune checkpoint inhibitors alone.^12^ IRE induced the release of damage-associated molecular patterns (DAMPs) and lead to a 2.3-fold increase of interferon (IFN)γ-secreting tumor antigen-specific splenocytes in long-term surviving mice compared to treatment-naive, as well as a greatly increased frequency of CD4 and CD8 memory cells.^12^

Only one prospective clinical trial of combined IRE and immune checkpoint inhibitors in PC has been published. O’Neill et al. included ten patients with locally advanced PC that were treated with IRE during open surgery in conjunction to palliative surgical procedures.^13^ Nivolumab started on postoperative day one to five and continued for up to eight weeks. The addition of nivolumab to IRE did not contribute to grade 3–4 adverse events (AEs), and no dose-limiting toxicities were reported. Median progression-free survival (PFS) and median overall survival (OS) was 6.3 months and 18 months, respectively. Authors were unable to assess the abscopal effect of IRE; however, little impact on circulating immune cells was found besides a rise in peripheral effector memory T cells by day 90. In addition to the study presented here, an ongoing phase II trial (NCT04212026) will similarly investigate the possible abscopal effect on residual disease of combined IRE and immune checkpoint inhibition in metastatic PC. In contrast to the present, this study will use PD-1 blockade by nivolumab administered from day one after IRE.

Taken together, preclinical evidence suggests that IRE-treatment of a tumor lesion may increase the effect of immune checkpoint inhibitors in untreated tumor lesions by an abscopal effect. The aims of the current study were to investigate the efficacy and safety of combined IRE and PD-1 inhibition by pembrolizumab in patients with treatment-refractory, liver-metastatic PC. We also studied the immunological impact of PD-1 inhibition in blood and tumor tissue.

## Results

From May 27^th^ to December 3^rd^, 2021, we included eight patients with liver-metastatic adenocarcinoma of the pancreas (Table 1). Inclusion was stopped prematurely by the trial safety board at the planned interim analysis, as no signs of clinical efficacy were apparent.

**Table 1:** Baseline characteristics of included patients

Age (years)	*Median (range)*	61 (39–71)
Time since diagnosis of inoperable disease (months)	*Median (range)*	13.2 (4.4–47.6)
Serum Ca 19-9 (kU/l)	*Median (range)*	7,360 (65–28,176)
Gender	*N*	
Male		6
Female		2
ECOG performance status	*N*	
0		3
1		5
Body mass index	*N*	
Normal (18.5–25)		6
High (>25)		2
Disease presentation	*N*	
Inoperable at diagnosis		6
Recurrent after prior curative resection		2
Number of prior lines of chemotherapy	*N*	
1		1
2		3
3		4
Prior chemotherapy regimens∗	*N*	
FOLFIRINOX		7
Gemcitabine and *nab*-paclitaxel		7
FOLFIRI		1
5-fluorouracil		1
Tocilizumab		1
Gemcitabine		2
Number of metastatic sites	*N*	
1		2
2		2
3		3
4		1
Serum lactate dehydrogenase	*N (median value in U/l)*	
Normal		6
Elevated		2 (359)
Serum albumen	*N (median value in g/l)*	
Normal		5
Decreased		3 (33)
Serum alkaline phosphatase	*N (median value in U/l)*	
Normal		2
Elevated		6 (241)
Plasma C-reactive protein	*N (median value in mg/l)*	
Normal		3
Elevated		5 (36)

^a^Patients may be counted more than once. For information about representativeness of participants see Table S1.

All patients were in Eastern Cooperative Oncology Group (ECOG) performance status (PS) 0–1, but otherwise displayed poor prognostic factors; seven patients had received at least two prior lines of chemotherapy, six patients had progressive disease (PD) at inclusion, six patients had additional extrahepatic metastatic sites, and serum cancer antigen (Ca) 19-9 was highly elevated (median approx. 7,000 kU/L).

### Safety outcome

All patients received the first dose of pembrolizumab and were subsequently treated with IRE of a liver metastasis, while only one patient received a 2^nd^ pembrolizumab-treatment following IRE. No dose modifications or delays were required due to toxicity. All AEs were deemed unrelated to treatment, except for three cases of pruritus evolving at day 10. AEs were mild (Common Terminology Criteria [CTC] AEs grade 1–2), except one case of CTCAE grade 3 anemia on day 10. All AEs are listed in Table 2. A total of six serious adverse events (SAEs) registered included hospitalization due to vomiting, pulmonary embolism, and ascites; and three cases of infection. All SAEs were deemed related to the disease and unrelated to treatment.

**Table 2:** Adverse events during trial

	Baseline	Day 10 ± 2	Day 24 ± 2
Evaluable patients (*n*)	8	8	6
Toxicity type (*n*)			
Fatigue			
*Grade 1*	3	4	2
*Grade 2*	3	3	3
Nausea			
*Grade 1*	4	2	0
*Grade 2*	0	2	2
Vomiting			
*Grade 1*	2	2	2
Anorexia			
*Grade 1*	3	2	2
*Grade 2*	2	3	1
Diarrhea			
*Grade 1*	4	4	0
Constipation			
*Grade 1*	3	2	0
*Grade 2*	0	0	1
Anemia			
*Grade 1*	2	3	3
*Grade 2*	0	1	0
*Grade 3*	0	1	0
Dizziness			
*Grade 1*	4	3	3
Dyspnea			
*Grade 1*	2	3	1
*Grade 2*	0	1	0
Edema			
*Grade 1*	3	2	0
*Grade 2*	0	0	1
Cough			
*Grade 1*	1	1	2
Arthralgia			
*Grade 1*	2	1	0
Myalgia			
*Grade 1*	0	1	1
Abdominal pain			
*Grade 1*	5	3	3
*Grade 2*	4	4	2
Pruritus			
*Grade 1*	0	3	1
Peripheral motor neuropathy			
*Grade 1*	1	1	1
Peripheral sensory neuropathy			
*Grade 1*	4	3	1
*Grade 2*	2	1	0
Back pain			
*Grade 1*	3	0	0
*Grade 2*	1	1	1
Eczema			
*Grade 1*	0	1	0
Venous thrombo-embolism			
*Grade 1*	0	0	0
*Grade 2*	0	1	0
Thrush			
*Grade 1*	0	1	0

Worst toxicity grade according to CTCAE version 5.0. Day counted from first administration of pembrolizumab.

### Efficacy outcome

**Figure 1:**
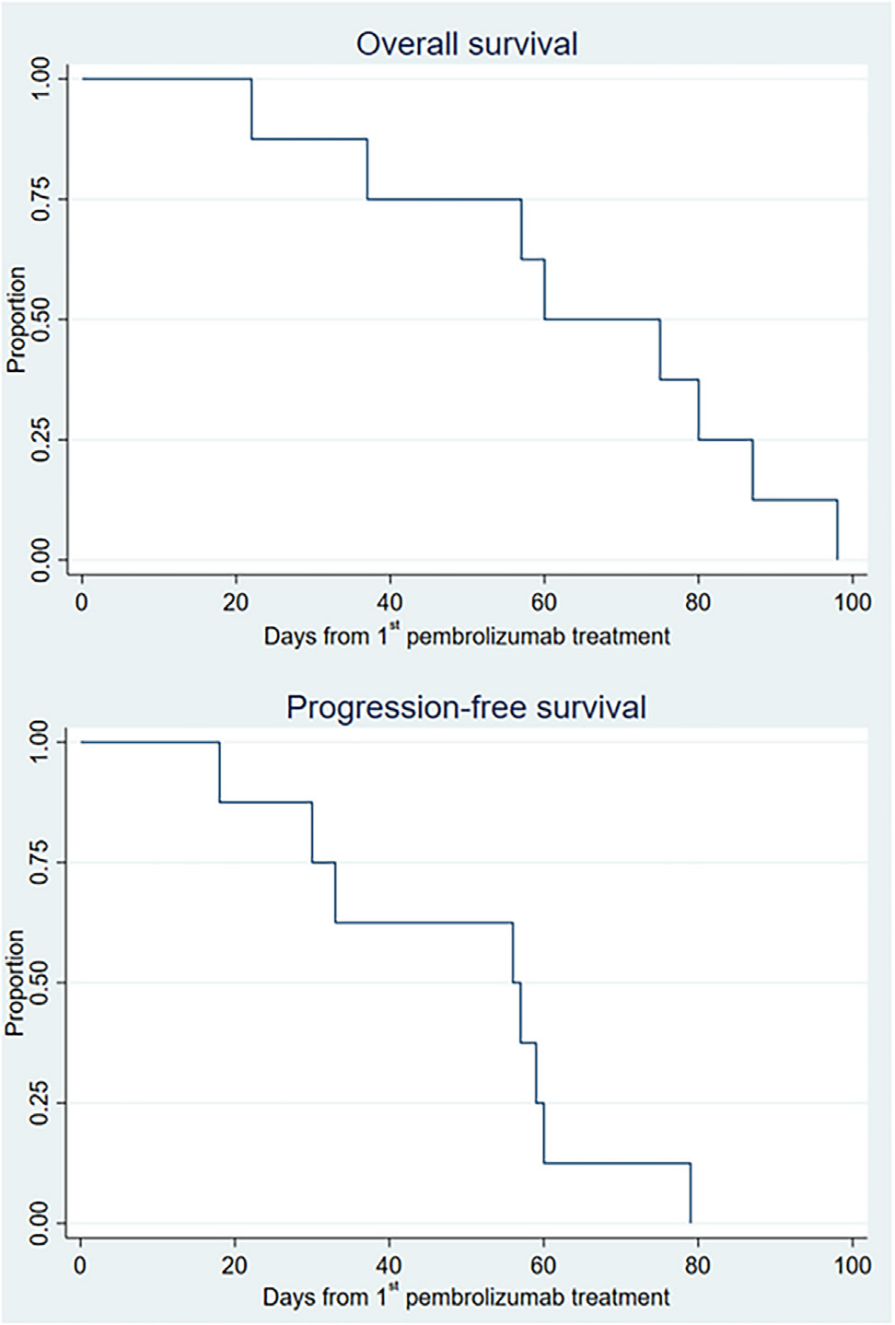
Kaplan-Meier plots of survival Overall survival (top) and progression-free survival (bottom) after initiation of treatment with pembrolizumab and IRE of a liver metastasis on day 10 ± 2 days.

All patients’ disease rapidly progressed resulting in an objective response rate (ORR) of 0% in eight evaluable patients. The reason for stopping treatment was PD in all cases. The median PFS was 1.8 months, and the median OS was 2.0 months (Figure 1). None of the patients received active oncological treatment after study termination.

**Figure 2:**
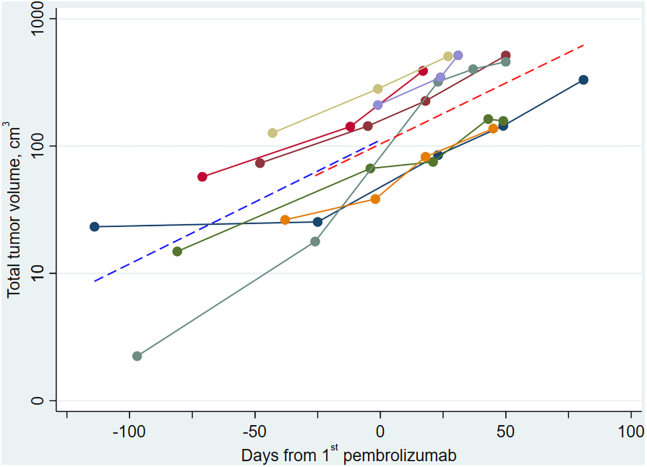
Longitudinal total tumor volumes Total tumor volumes obtained from 3D-segmented CT-scans for each patient plotted against time in days. Day 0 is the day of study treatment initiation. The IRE treated lesion and necrotic areas were excluded from measurements. Dashed lines are linear regression models of measurements prior to (blue) and after (red) treatment initiation. The scale on y axis is logarithmic.

Total tumor volume (Figure 2) increased exponentially with time in all patients. The mean volume doubling time (VDT) was 35 days (95% confidence interval [CI], 23–80 days) with no significant impact of the study treatment (*p* = 0.76).

### Patient reported outcomes

Quality of life (QoL) measured using the European Organisation for Research and Treatment of Cancer (EORTC) QoL Questionnaire C30 version 3 and nutritional status assessed by the short form version of The Scored Patient-Generated Subjective Global Assessment (PG-SGA) was assessed at baseline, but none of these assessments were later repeated (data not shown).

### Blood biomarkers

No patients had a Ca 19-9 biomarker response to pembrolizumab defined as a decline of at least 25% in samples taken prior to IRE compared to baseline. On the contrary, a rise >25% in Ca 19-9 after the first pembrolizumab was observed in three patients. In five paired samples taken immediately prior to IRE and one day after IRE, no significant changes in Ca 19-9 were observed, except for one patient, who had a 29% rise. Of five patients having at least two samples taken after IRE, one patient experienced a transient 41% drop at day 30, while the Ca 19-9 values of all other patients increased significantly.

**Figure 3:**
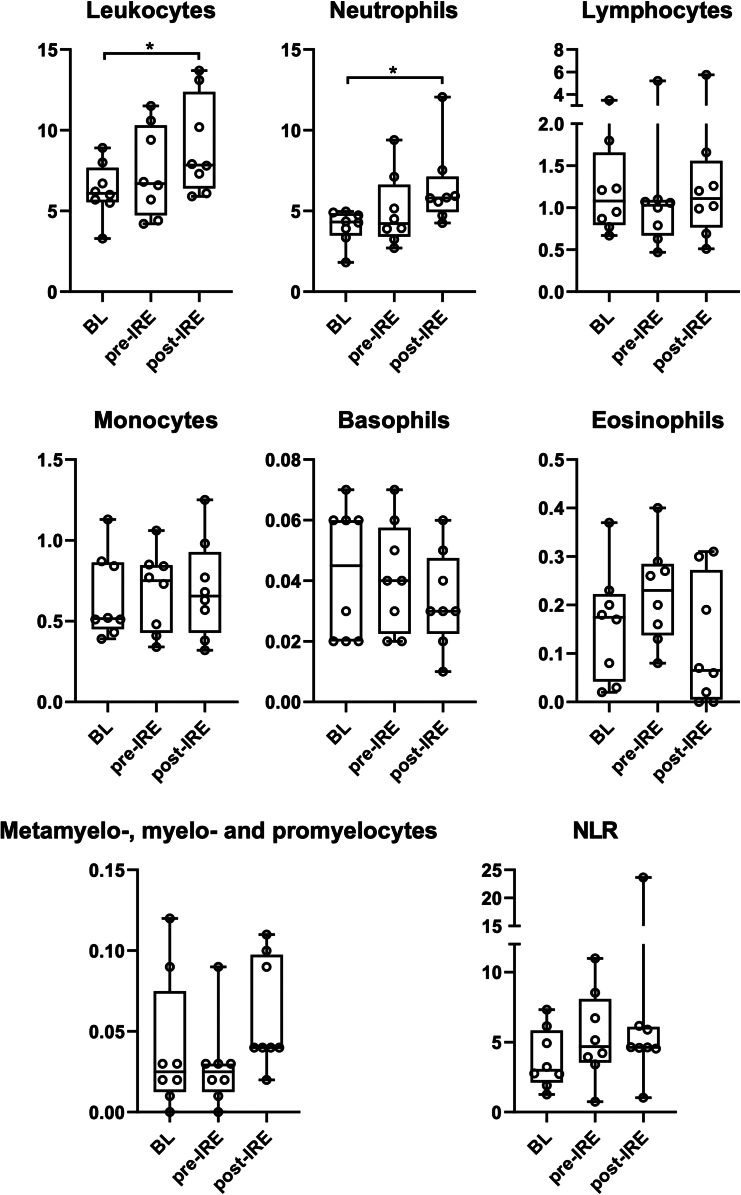
White blood cell counts White blood cells were counted at baseline (BL), 10 ± 2 days after treatment with pembrolizumab prior to IRE ablation of a liver metastasis (pre-IRE), and 1 day after IRE (post-IRE). The scale on y axes indicates the number of cells in 10^9^/L. The mean is indicated by horizontal bars, the standard deviation by boxes and the 95% CIs by vertical lines bars. Horizontal brackets with ∗ indicate ANOVA tests with significant outcome. NLR; neutrophil-to-lymphocyte ratio.

No significant changes in white blood cell counts were found comparing baseline with samples taken day 10 ± 2 days after pembrolizumab, however, the mean total leukocyte and neutrophil counts increased significantly the day after IRE treatment (Figure 3).

**Figure 4:**
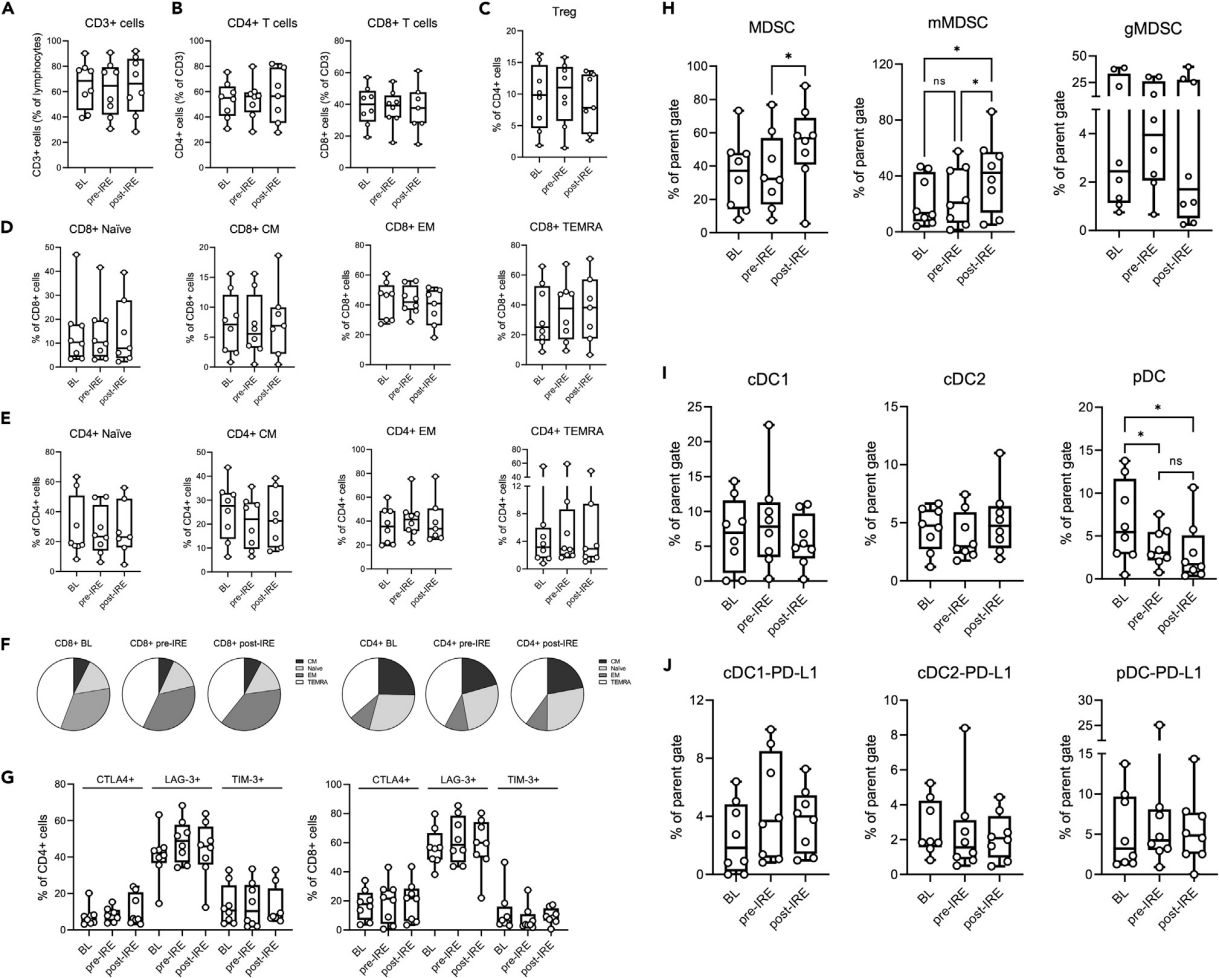
Frequency of myeloid and lymphocyte subpopulations Cell populations were analyzed at baseline (BL), 10 ± 2 days after treatment with pembrolizumab prior to irreversible electroporation of a liver metastasis (pre-IRE), and 1 day after IRE (post-IRE). The scale on y axes indicates the percentage cells. The mean is indicated by horizontal bars, the standard deviation by boxes and the min-max by vertical lines bars. Horizontal brackets indicate statistical comparison done and ∗ indicates a significant outcome (*p* < 0.05). (A) Fraction of circulating CD3^+^ cells out of total lymphocytes. (B) CD4^+^ and CD8^+^ T cells out of total CD3^+^ cells. (C) Tregs out of total CD3^+^ cells. (D) Naive, effector memory (EM), central memory (CM), and terminally differentiated effector memory (TEMRA) subpopulations out of total CD8^+^ cells. (E) Naive, EM, CM, and TEMRA subpopulations out of total CD4^+^ cells. (F) Parts-of-whole distribution of naive, EM, CM, and TEMRA subpopulations. (G) Exhaustion marker expression in CD4^+^ and CD8^+^ T cells. (H) Myeloid-derived suppressor cells (MDSC) (out of Lin^−^HLA-DR^-^CD16^−^CD33^+^CD11b^+^ cells), monocyte-like MDSC (mMDSC) and granulocyte-like MDSC (gMDSC) subpopulations (out of total MDSC). (I) Conventional dendritic cell (cDC) 1, cDC2, and plasmacytoid dendritic cell (pDC) subpopulations as a fraction of HLA-DR^+^Lin^−^. (J) PD-L1 expression on DC subsets as a fraction of total cDC1, cDC2, and pDC cells. Gating strategies are available in Figures S1–S4. Full list of cell population markers is available in Table S2.

Results of flow cytometry are summarized in Figure 4. The mean number of monocytic MDSCs (mMDSCs) was unchanged 10 ± 2 days after immunotherapy compared to baseline but increased immediately after IRE, whereas mean numbers of granulocytic MDSCs (gMDSCs) were not significantly altered. The mean number of plasmacytoid dendritic cells (pDCs) dropped significantly 10 ± 2 days after immunotherapy and tended to be even lower on the day after IRE, while the mean numbers of other subtypes of dendritic cells and PD-L1-expressing pDCs were not significantly altered. No significant changes during treatment were observed in other immune cell types investigated, including effector T cells.

**Figure 5:**
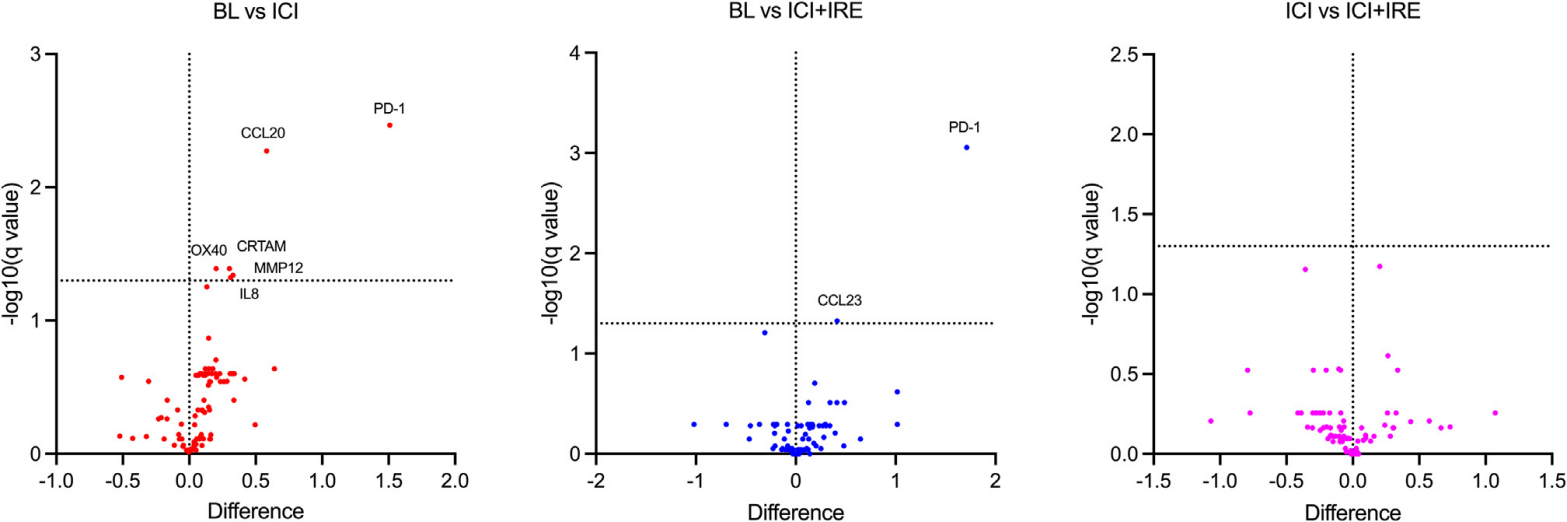
Differences in plasma concentration of selected cytokines and inflammation-related proteins Analyses were performed between baseline (BL) and 10 ± 2 days after treatment with immune checkpoint inhibitor (ICI) (left), baseline and one day after IRE (ICI+IRE) (middle) and after treatment with ICI and one day after IRE (right). Horizontal line specifies significance (*p* ≤ 0.01) without corrections for multiple testing. A list of upregulated cytokines and proteins and their expression and function is provided in Table S3. None of the assessed cytokines or proteins was significantly downregulated.

In plasma, a significant increase was observed in seven cytokines and proteins in samples taken after immunotherapy compared to baseline, especially PD-1, while none decreased (Figure 5). IRE had no apparent effect.

### Tissue analysis

**Figure 6:**
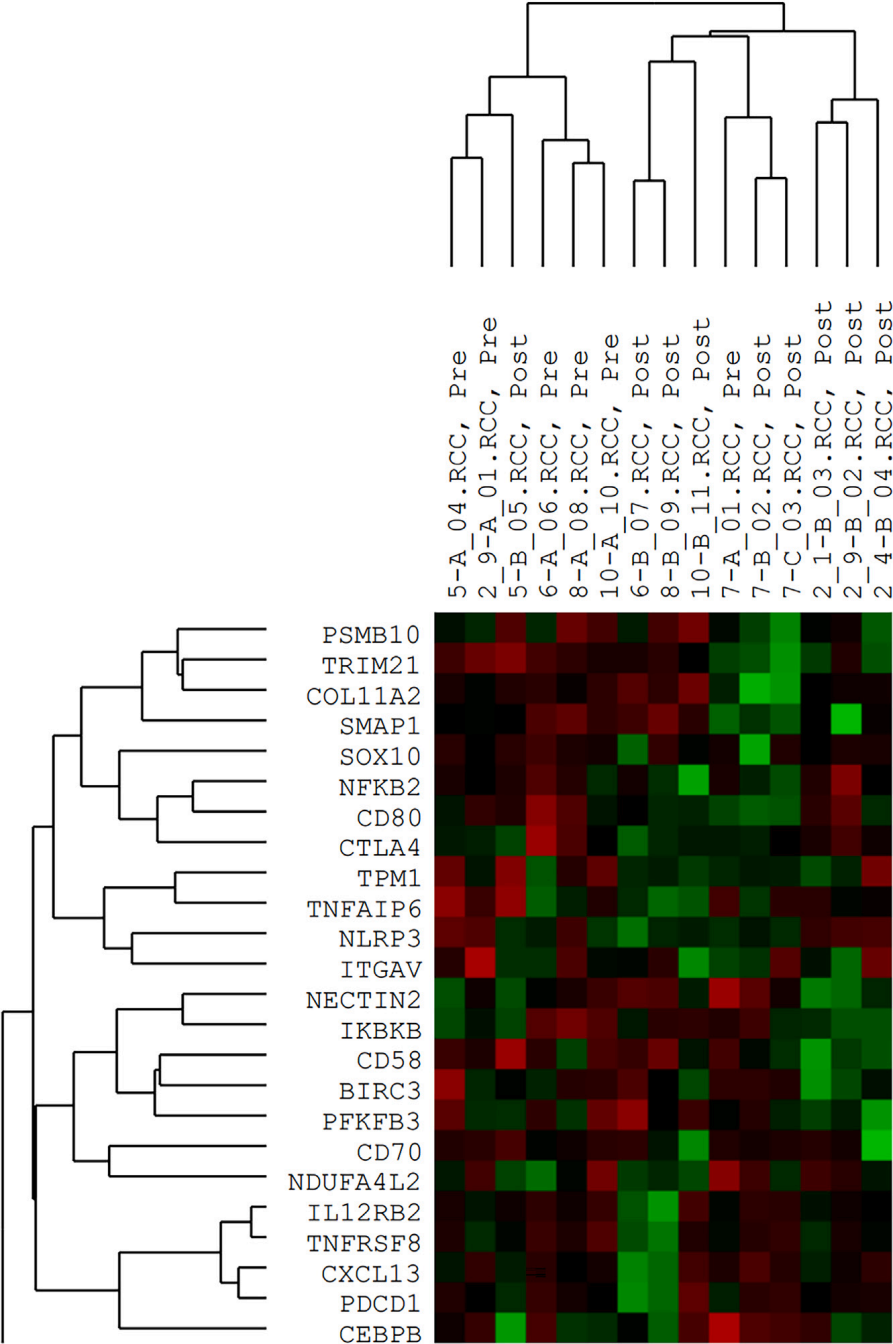
Heatmap of differentially expressed genes Analyses were performed at baseline (Pre) (*n* = 6) and on-treatment day 10 ± 2 days after pembrolizumab (Post) (*n* = 9) in liver metastases from PC patients.

Fifteen biopsies from eight patients were included. From four patients, the new baseline biopsy was chosen, while more adequate archival tissue was available for baseline assessment in two other patients. Baseline biopsies were not available in two patients. From all patients, an on-treatment liver tumor biopsy, performed on day 10 ± 2 days after initiation of immunotherapy but prior to IRE, were available, and from one patient also a post-IRE biopsy was available. In bulk analysis of six baseline versus nine on-treatment biopsies, mRNA gene expressions in liver biopsies clustered according to exposure to pembrolizumab, except for two specimens (Figure 6). Using *p* ≤ 0.10 as threshold without correction for multiple testing due to low number of specimens, we found upregulation of 24 and downregulation of seven immune-related gene transcripts in tumor tissue after pembrolizumab. Genes with differential expression between baseline and on-treatment biopsies are listed in Table 3, including their associations to immunological hallmarks. No significant differences in gene expression markers of effector T cells were observed (data not shown).

**Table 3:** Gene expression alterations in liver metastases after pembrolizumab

Hallmarks	TNF alpha signaling	Allograft rejection	IL2-STATS signaling	Inflammatory response	IFN gamma response	KRAS signaling	EMT	PIK3K-AKT-MTOR signaling	Complement	Hypoxia	Gene description
Upregulated genes											
*CD80*	X	X	–	–	–	X	–	–	–	–	CD80 molecule
*TNFAIP6*	X	–	–	X	X	–	–	–	–	–	TNF alpha induced protein 6
*PFKFB3*	X	–	–	–	–	–	–	–	–	–	6-phosphofructo-2-kinase/fructose-2,6-biphosphatase 3
*CEBPB*	X	–	–	–	–	–	–	–	–	–	CCAAT enhancer binding protein beta
*BIRC3*	X	–	–	–	–	–	–	–	–	–	Baculoviral IAP repeat containing 3
*NFKB2*	X	–	–	–	–	–	–	–	–	–	Nuclear factor kappa B subunit 2
*NLRP3*	–	X	–	X	–	–	–	–	–	–	NLR family pyrin domain containg 3
*PSMB10*	–	X	–	–	X	–	–	–	–	–	Proteasome 20S subunit beta 10
*CXCL13*	–	X	–	–	–	–	–	–	–	–	C-X-C motif chemokine ligand 13
*IKBKB*	–	X	–	–	–	–	–	–	–	–	Inhibitor of nuclear factor kappa B kinase subunit beta
*ITGAV*	–	–	X	–	–	–	–	–	–	–	Integrin subunit alpha V
*CTLA4*	–	–	X	–	–	–	–	–	–	–	Cytotoxic T lymphocyte associated protein 4
*TNFRSF8*	–	–	X	–	–	–	–	–	–	–	TNF receptor superfamily member 8
*CD70*	–	–	–	X	–	–	–	–	–	–	CD70 molecule
*TRIM21*	–	–	–	–	X	–	–	–	–	–	Tripartite motif containing 21
*PDCD1*	–	–	–	–	–	X	–	–	–	–	Programmed cell death 1 (*PD1*)
*SOX10*	–	–	–	–	–	X	–	–	–	–	SRY-box transcription factor 10
*TPM1*	–	–	–	–	–	–	X	–	–	–	Tropomysin 1
*NECTIN2*	–	–	–	–	–	–	–	–	–	–	Nectin cell adhesion molecule 2
*IL12RB2*	–	–	–	–	–	–	–	–	–	–	Interleukin 12 receptor subunit beta 2
*COL11A2*	–	–	–	–	–	–	–	–	–	–	Collagen type XI alpha 2 chain
*NDUFA4L2*	–	–	–	–	–	–	–	–	–	–	NDUFA4 mitochondrial complex associated like 2
*SMAP1*	–	–	–	–	–	–	–	–	–	–	Small ArfGAP 1
*CD58*	–	–	–	–	–	–	–	–	–	–	CD58 molecule
Downregulated genes											
*LCK*	–	X	–	–	–	–	–	X	X	–	LCK proto-oncogene, Src family tyrosine kinase
*EGFR*	–	X	–	–	–	–	–	X	–	X	Epidermal growth factor receptor
*DPP4*	–	–	–	–	–	–	–	–	X	–	Dipeptidylpeptidase 4
*FBP1*	–	–	–	–	–	–	–	–	–	X	Fructose-bisphosphatase 1
*NCAM1*	–	–	–	–	–	–	–	–	–	–	Neural cell adhesion molecule 1
*SPR54*	–	–	–	–	–	–	–	–	–	–	Signal recognition particle 54
*API5*	–	–	–	–	–	–	–	–	–	–	Apoptosis inhibitor 5

All altered gene expressions after 10 ± 2 days on pembrolizumab compared to baseline, and their associations to immunological hallmarks. *p* ≤ 0.10 without correction for multiple testing was used as threshold.

## Discussion

We here present data on a clinical study of IRE combined with immune checkpoint blockade in patients with metastatic PC. Despite promising preclinical results in PC mouse models, we found no indications of clinical activity and the trial was terminated early due to futility. No patients responded as per RECIST (response evaluation criteria in solid tumors) or biochemically. Tumor VDT was similar prior to and after study treatment initiation, and tumor growth followed an unbroken, exponential curve. Compared to previously published estimates of VDT of primary pancreatic tumors of, on average, 132 days in untreated individuals,^14^ the growth of disease was very rapid with a mean VDT of only 33 days. In agreement with others,^13^ the combined IRE and immune checkpoint inhibitor treatment was tolerable and safe, and most AEs and all SAEs were attributable to disease rather than treatment.

Several explanations of the negative result may apply. Six of eight patients had PD at inclusion and several other poor prognostic characteristics were evident. As treatment with pembrolizumab was inefficient in our patients, the delay of IRE probably had a detrimental effect on the outcome of the combined procedure. Moreover, the large tumor burden of patients may have contributed to the poor immune response.^15^ Timing may be important for efficacy. Our hypothesis was that activation of the immune system by immunotherapy first would allow a rapid tumor response when massive amounts of tumor-antigens were to be present in the circulation ten days after, due to IRE. In a proof-of-concept study of anti-PD1 therapy given for three doses before radiation therapy, indications of an abscopal effect of radiotherapy were observed,^16^ but authors noted that concurrent priming may be most optimal. This was based on results of a mouse model showing that priming may lead to induction of dysfunctional CD8 cells due to CD8^+^ T cell exhaustion.^17^ However, in the current study, we saw no significant changes in numbers of exhausted T cells in the circulation ten days after pembrolizumab. We chose to use monotherapy only as pembrolizumab is well tolerated and as patients with chemotherapy-resistant metastatic PC are often intolerant to side effects. Used at an earlier stage of disease, future experiments may involve inhibitors of other checkpoints and combinations of these, such as PD-L1/PD-1 combined with CTL-4 blockade^7^ or blockade of LAG-3.^18^ Finally, combinations with chemotherapy may also be feasible to increase efficacy as has been demonstrated in clinical studies of several other cancers.^19^ Although it could increase toxicity, treating more than one metastasis by IRE in order to maximize the abscopal effect may be considered as metastases may be biologically heterogeneous.^20^

Efficacy of immune checkpoint inhibitors is dependent on the immunological properties of the tumor microenvironment (TME)^21^ and to some extent, changes in circulating immune cells may reflect changes in tumors *in situ.*^22^ In a recent feasibility study of locally advanced PC combining IRE followed by nivolumab,^13^ peripheral effector memory T cell numbers increased, while no differences at day 90 in CD4, naive or-central memory T cells were found. We were unable to assess the long-term impact on circulating immune cells but found no short-term changes in subpopulations of T cells during treatment. We observed an increase in mMDSCs and neutrophils immediately after IRE. Both cell types have been correlated with immunosuppression and poor outcome of immunotherapy,^23^ which may imply a negative impact of IRE. Indeed, hyperprogression of pancreatic cancers after a surgical trauma has been suggested,^24^ but our measurements of tumor VDT did not support this hypothesis. Moreover, with longer observation others found no effect on myeloid cell lines after IRE of PC^25^ as well as blood T cell immunoglobulin and mucin domain (TIM-3), which may be associated with non-response to PD-L1/PD-1 inhibition,^26^ and cytotoxic T lymphocyte-associated protein (CTLA)-4, was unchanged in our patients. We found that pDCs decreased in the circulation ten days after initiation of immunotherapy and tended to be even lower after IRE, however, their expression of PD-L1 was unchanged. PDCs are known to secrete large amounts of IFNα/β on antigen exposure, which has been shown to have a potent anti-tumor effect in mouse models.^27^ A high expression of PD-L1 is typical for DCs in the tolerogenic state,^28^ and it is possible that DCs were stimulated to migrate but were not immunogenic. Future studies involving RNA sequencing (RNA-seq) of blood leukocytes may disclose alterations in gene expressions beyond alterations in surface protein expression disclosed by flow cytometry.^29^

The longitudinal analyses of cytokines and inflammation-related proteins in plasma revealed a significant increase of several cytokines after treatment with pembrolizumab. In particular, circulating PD-1 increased markedly confirming prior findings in, e.g., malignant melanoma, but showing no significant predictive value for tumor response.^30^^,^^31^ Of interest, OX40, that also increased after treatment with pembrolizumab, has been suggested as a novel target for immunotherapies in combination with PD-1 inhibition.^32^

Studies of dynamics in the TME during immunotherapy of PC in humans are very few. In a randomized phase II trial, including a treatment-arm of gemcitabine and *nab*-paclitaxel combined with PD-1 blockade, analysis of paired pre-treatment and on-treatment biopsies from five patients revealed a numerically decreased percentage of tumor cells expressing PD-L1 in all samples measured during treatment,^33^ but the impact of chemotherapy could not be assessed. In 32 paired tumor biopsies taken at baseline and two weeks after treatment initiation in a randomized phase II study of stereotactic body radiotherapy of a liver metastasis and immunotherapy (with either PD-1- or combined PD-1- and CTLA-4-blockade), only a minority demonstrated an increase in tumor-associated immune cells. Notably, increased infiltration of CD8^+^ T cells was seen in only 2 of 27 cases.^7^ We found that expression patterns of immunologically significant genes separated pretreatment and on-treatment biopsies, indicating a rapid *in situ* effect of PD-1 blockade. Transcripts of 24 genes that were upregulated at day ten after treatment indicated mostly immune activation and included those involved in TNFα signaling, and hallmarks of IL2-STATS (signal transducer and activator or transcription signaling) and IFNγ response, including *CTLA4*. However, upregulation was also seen of *PD1*, and of *CD80* and *SOX10* that might contribute to immunosuppression^34^ and are all included in “genes down-regulated by *KRAS*.” Moreover, some gene expressions related to epithelial-mesenchymal transition (EMT) were upregulated as were *CD58*, which in PC has been correlated with poor prognosis.^35^^,^^36^ Downregulation was seen of seven genes, including those involved in the *KRAS* oncogenic down-stream cascade (hallmarks of *PIK3-AKT-MTOR* signaling), in hallmarks of complement that might have an immunosuppressive function in the TME,^37^ and in hallmarks of hypoxia—a possible mechanism of resistance to immunotherapy.^38^ In addition, downregulation of *EGFR* expression was observed which may reduce expression of PD-L1 and increase efficacy of immunotherapy.^39^ Although biopsies were prepared to include tumor tissue only, the methodology did not allow for assigning gene expressions to specific types of cells.^40^ It is notable, but in agreement with prior findings using semi-quantitative assessments by immunohistochemistry,^7^ that no significant changes were observed in gene expressions directly related to effector T cells. Except for PD1 that was upregulated in tissue and increased in serum, none of the longitudinal changes observed in circulating immune cells, plasma cytokines and proteins corresponded to differences found in RNA expressions in paired tissue samples, suggesting that the activity of checkpoint inhibition did not affect the tumor directly. In addition, as no difference was found in plasma cytokines and proteins between measurements taken before or after IRE, the suggested effect of IRE on immune signaling could not be confirmed by this study.

Although treatment with pembrolizumab followed by IRE of a liver metastasis is feasible and safe in patients with metastatic PC, we found no clinical or biochemical effect in the first eight of 16 planned patients, and the study was terminated early for futility by the trial safety board at a planned interim analysis. Due to rapidly progressing disease, we were unable to study long-term effects of the combined treatment. Future studies should engage patients at an earlier phase of disease. Immunological analyses showed a drop in blood pDCs after immunotherapy and an increase in mMDSCs and neutrophils immediately after IRE, but no changes in numbers of effector T cells. Several plasma cytokines and proteins increased after treatment with pembrolizumab, especially PD-1, but IRE had no apparent added effect. Analysis of gene expressions in sequential biopsies suggested that PD-1 blockade may rapidly stimulate a predominantly immune permissive environment in metastases, but seemingly has no effect on effector cells. These findings support that modalities to recruit effector cells to the TME together with PD-1 blockade and, possibly, IRE may pave the way for the long-awaited break-thorough for immunotherapy in PC.

### Limitations of the study

Major limitations are the small sample size, lack of post-IRE biopsies and short time on treatment limiting the statistics. As this was an uncontrolled study, we cannot exclude that the observed immunological changes may in part be caused by effects of progressing cancer rather than the intervention and we were unable to conclude on a possible abscopal effect of IRE.

## Resource availability

### Lead contact

Further information and requests for resources and reagents should be directed to and will be fulfilled by the lead contact, Morten Ladekarl (morten.ladekarl@rn.dk).

### Materials availability

This study did not generate new unique reagents.

### Data and code availability

• The human gene expression data generated in this study are not publicly available due to patient privacy requirements but are available upon reasonable request from the lead contact. Other data generated in this study are available within the article and its supplementary data files.
• This paper does not report original code.
• Any additional information required to reanalyze the data reported in this paper is available from the lead contact upon request.

## Acknowledgments

The study was supported by a grant from the 10.13039/100008363Danish Cancer Society (Professor Morten Ladekarl, grant number R269-A15935). Additional funding was obtained from 10.13039/501100006304Aalborg University Hospital (Dr. Rasmus V. Flak), H.E. og N.C. Brogaards Legat til Kræftforskningens Fremme (Professor Morten Ladekarl), and Lions Denmark (Ralf Agger).

## Author contributions

R.V.F.: conception and design, provision of patients and clinical data, data interpretation, final draft manuscript writing, accountable for all aspects of the work. E.K.-O.: collection and assembly of data, data analysis and interpretation, final draft manuscript writing. N.D.S.: collection and assembly of data, data analysis. R.A.: collection and assembly of data, data interpretation, administrative support. M.T.S.: collection of clinical data. G.N., S.C., and S.S.: provision of patients and clinical data. L.Ø.P.: provision of clinical data, administrative support. S.D.: collection and assembly of data, data analysis and interpretation, final draft manuscript writing. O.T.-U.: conception and design, administrative support. M.L.: conception and design, provision of patients and clinical data, data interpretation, first and final draft manuscript writing, accountable for all aspects of the work.

## Declaration of interests

M.L. received a research grant from Scandion Oncology A/S, Denmark.

## STAR★Methods

### Key resources table

**Table:** 

REAGENT or RESOURCE	SOURCE	IDENTIFIER
**Antibodies**		

Mouse monoclonal anti-human CD3, mIgG1, κ, FITC (SK7)	BioLegend	RRID:AB_204399
Mouse monoclonal anti-human CD3, mIgG1, V500 (SP34-2)	BD Horizon	RRID:AB_1937330
Mouse monoclonal anti-human CD4, mIgG_1_, κ, BV711 (SK3)	BD Horizon	RRID:AB_2916890
Mouse monoclonal anti-human CD4, mIgG_1_, κ, FITC (RPA-T4)	BD Pharmingen	RRID:AB_395751
Mouse monoclonal anti-human CD8, StarBright 515, mIgG1 (LT8)	Bio-Rad	RRID:AB_3100053
Mouse monoclonal anti-human CD8, mIgG_1_, κ, BV711 (RPA-T8)	BD Horizon	RRID:AB_2744463
Mouse monoclonal anti-human PD-1, mIgG_1_, κ, APC (MIH4)	BD Pharmingen	RRID:AB_1645458
Mouse monoclonal anti-human CTLA-4, mIgG_2a_, κ, BV421 (BNI3)	BD Horizon	RRID:AB_2737762
Mouse monoclonal anti-human TIM-3, PE, mIgG1, κ (7D3)	BD Pharmingen	RRID:AB_2739296
Mouse monoclonal anti-human LAG-3, BV605, mIgG_1_, κ (T47-503)	BD Pharmingen	RRID:AB_2742761
Mouse monoclonal anti-human CD45, V500, mIgG1, κ (HI30)	BD Horizon	RRID:AB_1937332
Mouse monoclonal anti-human CD45RA, BV421, mIgG_1_, κ (5H9)	BD Optibuild	RRID:AB_2739846
Mouse monoclonal anti-human CCR7, BV605, mIgG1, κ (2-L1-A)	BD Horizon	RRID:AB_2869850
Mouse monoclonal anti-human CD19, FITC, mIgG_1_, κ (HIB19)	BD Pharmingen	RRID:AB_395812
Mouse monoclonal anti-human CD20, FITC, mIgG1, κ (L27)	BD	RRID:AB_2868818
Mouse monoclonal anti-human CD56, PE-Cy7, mIgG_1_, κ (CMSSB)	Thermo Fisher	RRID:AB_11181516
Mouse monoclonal anti-human HLA-DR, APC-H7, mIgG_2a_, κ (L243)	BD Pharmingen	RRID:AB_1645738
Mouse monoclonal anti-human HLA-DR, BV605, mIgG_2a_, κ (G46-6)	BD Horizon	RRID:AB_2744478
Mouse monoclonal anti-human CD33, PE, mIgG1, κ (WM53)	BD Pharmingen	RRID:AB_394173
Mouse monoclonal anti-human CD11b, APC, mIgG_1_, κ (ICRF44)	BD Pharmingen	RRID:AB_396860
Mouse monoclonal anti-human CD14, BV421, mIgG2b, κ (MφP9)	BD Horizon	RRID:AB_2870488
Mouse monoclonal anti-human CD14, FITC, mIgG2a, κ (M5E2)	BD Pharmingen	RRID:AB_395798
Mouse monoclonal anti-human CD16, BV785, ImgG1, κ (3G8)	BioLegend	RRID:AB_2916901
Mouse monoclonal anti-human CD15, BV650, mIgG1, κ (W6D3)	BioLegend	RRID:AB_2562131
Mouse monoclonal anti-human CD303, buv563, mIgG_2b_, κ (V24-785)	BD Optibuild	RRID:AB_2872834
Mouse monoclonal anti-human CD1c, PE, mIgG_1_, κ (F10/21A3)	BD Pharmingen	RRID:AB_2739006
Mouse monoclonal anti-human CD141, APC, mIgG_1_, κ (1A4)	BD Pharmingen	RRID:AB_2738608
Mouse monoclonal anti-human PD-L1 (CD274), BV421, mIgG_1_, κ (MIH1)	BD Horizon	RRID:AB_2738396

**Software and algorithms**		

FlowJo, version10	FlowJo LLC, Beckton Dickinson	
Prism, version 10.2.2	Graphpad	
Expression Heatmapper	http://heatmapper.ca/expression/	
nSolver, version 4.0	NanoString Technologies, Seattle, WA https://nanostring.com/	
3D Slicer, version 4.11	https://www.slicer.org/	
Stata, version 16	StataCorp LLC, College Station, TX, USA	

**Other**		

High Pure FFPE RNA Isolation kit	Roche Diagnostics GmbH, Mannheim, Germany	06650775001
NanoKnife™, version 3.0	Angiodynamics, Queensbury, NY, USA	

### Experimental model and study participant details

#### Patients

Patients included were >18 years of age, had histologically verified liver-metastatic pancreatic ductal adenocarcinoma with one liver metastasis treatable by IRE and at least one additional measurable lesion. One liver lesion, not selected for IRE, should be suited for repeated biopsy by transcutaneous core needle. Patients should have failed or be intolerant to at least one line of palliative chemotherapy and be in ECOG PS 0–1. Additional requirements were adequate bone marrow, liver and renal function, no uncontrolled medical disease, no history of serious autoimmune disorder, no need for systemic immunosuppressive drugs (a dose of prednisone-equivalent ≤10 mg/day for maximally 7 consecutive days was permitted), no coexisting malignant disease, symptomatic CNS metastases or decompensated liver cirrhosis. Exclusion criteria specifically aimed at IRE included major dilation of veins or bowel obstructing the needle path, persistent atrial fibrillation, metal objects, *e.g*., biliary self-expandable metallic stents within 50 mm of ablation target, and cardiac pacemaker or implantable defibrillator, that could not be safely disconnected during IRE treatment. All patients signed informed consent prior to enrollment.

#### Ethical approval

The protocol was approved by the regional ethics committee of Region North, Denmark (*N*-20200085), and the Danish Medical Agency and conducted in accordance with The International Council for Harmonisation of Technical Requirements for Registration of Pharmaceuticals for Human Use (ICH) Good Clinical Practice guidelines.

### Method details

#### Aims

In this single center, investigator-initiated, prospective trial we wanted to explore whether combining PD-1 blockade and IRE of a liver metastasis yielded an acceptable ORR to warrant further investigations. The coprimary outcome was rate of AEs ≥ grade 3 according to CTCAE ver. 5. Secondary outcomes included all treatment-related AEs, median OS, median PFS, mean difference in perceived QoL and nutrition status. Exploratory biomarkers included changes in blood immune cell numbers, immuno-oncology related cytokines and inflammation related proteins in plasma and serum Ca 19-9, and changes in RNA expressions in sequential liver biopsies. Finally, the impact of treatment on tumor volume growth rate was assessed radiologically.

#### Treatment

Anti-PD-1 antibody pembrolizumab was administered at a fixed dose of 400 mg intravenously every 6 weeks. Pembrolizumab could be continued for six months, or until intolerability or progression. During the active treatment, patients were submitted to clinical assessment, bloodwork and contrast-enhanced CT-scans of the thorax and abdomen every 2 months.

The IRE procedure was carried out percutaneously on day 10 ± 2 days from the first administration of pembrolizumab under general anesthesia with ultrasound-guidance. The commercially available NanoKnife system (Angiodynamics, Queensbury, NY) was used, with fixed pulse parameters. Deep neuromuscular blockade was utilized during the procedure. Electrical pulse delivery was synced to the refractory phase of the patient’s electrocardiogram (ECG) using a commercial device. Two IRE probes were placed in parallel 10–26 mm apart, bracketing the tumor. 90 pulses per pair of electrodes were given with a pulse length of 90 μsec and an electrical field strength of 1500 V/cm. After the IRE procedure, patients were transferred to the surgical ward for at least 24 h of observation.

### Biopsies and blood sampling

Core needle biopsies from liver metastases were planned at three different timepoints; baseline, on-treatment day 10 ± 2, and day 10 ± 2 after the 2^nd^ immune checkpoint inhibitor-dose. The timing of biopsies was planned to minimize clinical visits and patient discomfort, the first on-treatment biopsy coinciding with the IRE ablation and the second coinciding with the first response evaluation. The baseline biopsy was omitted if sufficient archival tissue from a liver metastasis was present. All biopsies were immediately fixed in formalin and embedded in paraffine (FFPE).

Standard blood tests and blood biomarker analyses were performed at baseline, 10 ± 2 days after the first treatment with pembrolizumab prior to IRE, and one day after IRE. Further blood tests were planned prior to subsequent treatments with pembrolizumab. For biomarker assessment, 9 mL sodium heparinized blood samples were drawn and transferred on ice for immediate flow cytometry of whole blood. Blood for cytokine analyses were collected in EDTA tubes and centrifuged at 2200g for 10 min. Plasma samples were collected and immediately frozen at −80°C for bulk analysis.

#### Peripheral blood immunological analyses

Whole blood was collected, and myeloid cells and lymphocytes were purified by Lymphoprep separation (Sigma Aldrich). Myeloid populations were analyzed on freshly isolated cells, and cells used for T cell subpopulation investigation were frozen before analysis. Cells were labeled with four panels of fluorochrome conjugated antibodies specific for multiple subpopulations of leukocytes, and the status of and possible changes within the populations was monitored by multicolor flow cytometry using a Cytoflex S flowcytometer (Beckman Coulter). Standard complete blood cell counts were used for assessment of total leukocytes, neutrophils, lymphocytes, monocytes, basophils and eosinophils. Proteomic analysis was performed by a third party (BioXpedia A/S, Denmark) using an assay of 96 immuno-oncology related cytokines and inflammation related proteins (Olink target 96).

#### Tumor tissue analyses

For RNA extraction, sections of 10 μm thickness were cut from FFPE liver biopsies. Macrodissection for reduction of non-metastatic tissue was performed, when applicable. The number of sections cut depended on the size of the metastatic area on the slide. At least 100 mm^2^ total area was included from all biopsies. The sections were prepared and processed according to the Prosigna Breast Cancer Prognostic Gene Signature Assay package insert (2016-09 LBL-C0223-06). Total RNA was extracted using High Pure FFPE RNA Isolation kit (Roche Diagnostics GmbH, Mannheim, Germany, 06650775001), according to the Prosigna protocol. The RNA samples were stored at −80°C.

mRNA gene expression levels were assessed using 100 ng RNA and the PanCancer immune-profiling panel, Immuno-Oncology 360 (NanoString Technologies, Seattle, WA), consisting of 770 genes and giving a 360° view of tumor biology-related gene expression in the metastases. Normalization of RNA was performed using the geometric mean of negative internal controls, positive controls, and 20 housekeeping genes. Target-probe complexes were read, counted, and processed within the Counter Digital Analyzer (NanoString Technologies, Seattle, WA). The raw digital counts of expression were exported to the nSolver v4.0 software (NanoString) for downstream analysis following the manufacturer’s protocol.

#### Tumor response assessments

Radiological response was prospectively evaluated by RECIST 1.1 by an experienced radiologist. Furthermore, total tumor volume was assessed by blinded review of consecutive CT-scans available prior to and after study enrollment. Tumor lesions were segmented using an open-source software (3D slicer version 4.11), excluding the IRE treated lesion and necrotic areas, and the total tumor volume was calculated by summing up all individual tumor volumes of the patient. The VDT was calculated based on linear regression of logarithmic transformed estimates.

### Quantification and statistical analysis

Using the Wald z test for one-sample proportions, a study power of ≈75%, an assumed ORR of checkpoint inhibition alone of 0.1% and of combined checkpoint inhibition with IRE of >25%, we aimed to include 16 patients. A high rate of benefit was required for this demanding treatment to be of interest for further development. Descriptive statistics were applied to the safety data. Survival plots were constructed using the Kaplan-Meier estimator. Markedly non-normally distributed data were transformed prior to inferential comparisons. Flow cytometry data and blood cell counts was analyzed by one-way ANOVA with Turkey correction using Stata v16 (StataCorp LLC, College Station, TX). An overall α-level of 0.05 was used as a cut-point for statistical significance and statistical tests were, except for the study sample size calculation, two-sided. Differential gene expression was analyzed based on bulk analysis of baseline and on-PD-1 inhibitor treatment biopsies, where the normalized data were used to calculate the *p*-value for differences in expression in liver metastases before and after treatment for each gene. Due to the limited number of available biopsies, a cut-off of *p* ≤ 0.10 was used.

### Additional resources and availability

The protocol was registered at EudraCT (2020-004536-22) and was made available from clinicaltrials.gov (NCT04835402). The following supplementary data are available.

## Supplemental information

Document S1. Figures S1‒S4 and Tables S1‒S3

Data S1. Study protocol, related to STAR methods
