# Supplementary material for: Pembrolizumab followed by irreversible electroporation of a liver metastasis in pancreatic cancer patients
Source: iScience. 2024 Sep 24;27(10):111026. doi: 10.1016/j.isci.2024.111026 (PMC11602522; doi:10.1016/j.isci.2024.111026)
Supplement: Document S1. Figures S1‒S4 and Tables S1‒S3 [file mmc1.pdf]

iScience, Volume *27*

Supplemental information

Pembrolizumab followed by irreversible electroporation of a liver metastasis in pancreatic cancer patients

Rasmus Virenfeldt Flak, Emil Kofod-Olsen, Nikolaj Dich Sølvsten, Gintare Naujokaite, Ralf Agger, Mogens Tornby Stender, Signe Christensen, Susy Shim, Laurids Østergaard Poulsen, Sönke Detlefsen, Ole Thorlasius-Ussing, and Morten Ladekarl

Supplementary figure S1: T memory cell and T regulatory cell gating strategy, related to figure 4
Total lymphocytes were gated based on side scatter (SSC) and forward scatter (FSC) (color is heatmap of the CD3 marker). Live cells were gated using the eFlour780 live/dead stain. T cells were identified as CD3⁺ cells, and Th and Tc cells were subsequently gated as CD4⁺ and CD8⁺. Naïve, CM, EM and TEMRA populations were identified based on CCR7 and CD45RA expression. Tregs were identified as CD4⁺CD25ʰⁱCD127ˡᵒ. Numbers in the plots represent percentage gated cells.

T memory and Treg gating

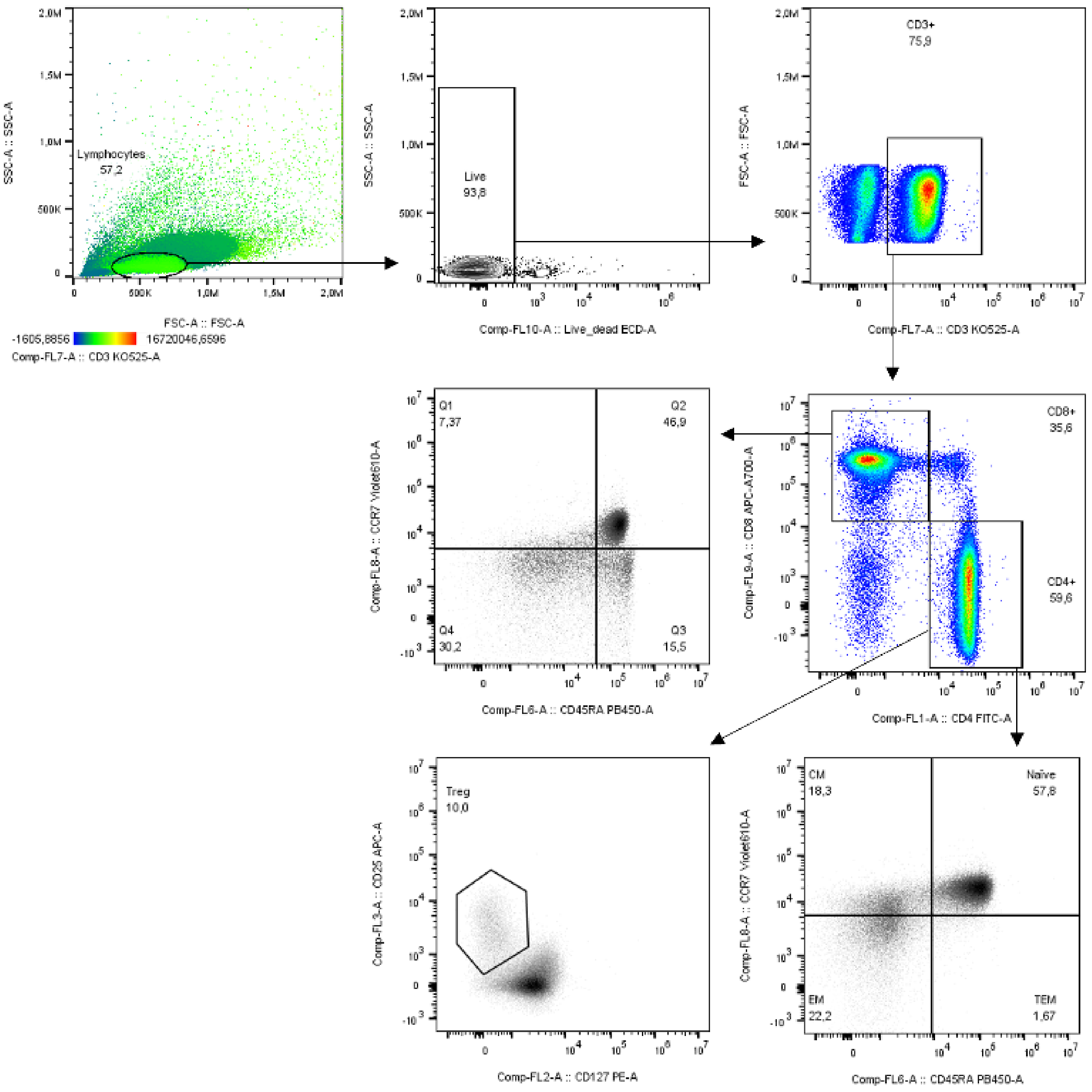

Supplementary figure S2: Exhausted T cell gating strategy, related to figure 4

Total lymphocytes were gated based on side scatter (SSC) and forward scatter (FSC). Live cells were gated using the eFlour780 live/dead stain. T cells were identified as $CD3^+$ cells, and Th and Tc cells were subsequently gated as $CD4^+$ and $CD8^+$. The two T cell populations were subsequently gated for the exhaustion markers TIM-3, LAG-3 and CTLA4.

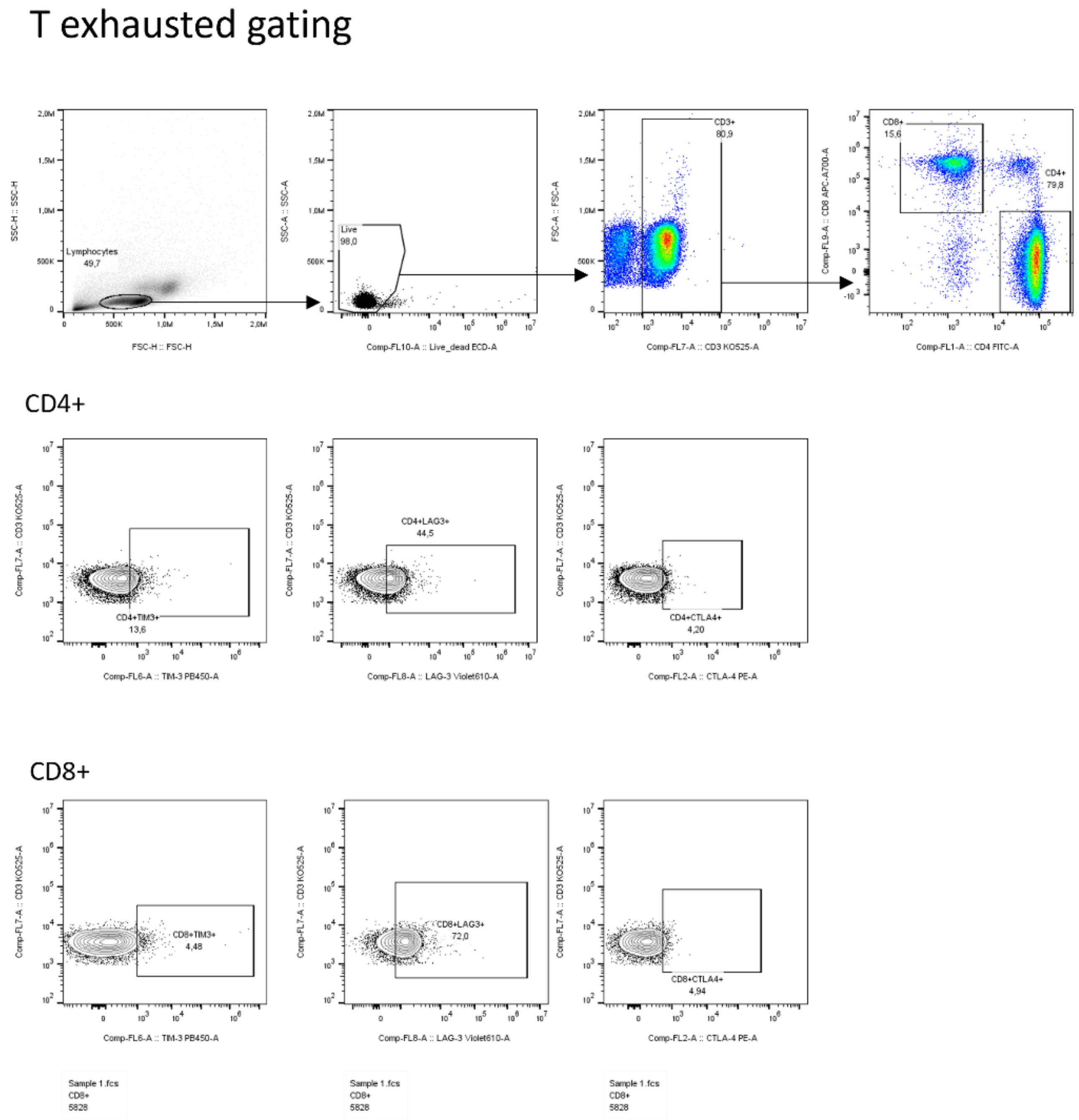

Supplementary figure S3: MDSC gating strategy, related to figure 4

Cells were identified based (SSC) and forward scatter (FSC). Leukocytes were identified based on SSC and CD45 expression. $SSC^{hi}CD45^{+}$ cells were excluded as this population largely consists of eosinophils. Single cells were gated based on FSC-H vs. FSC-A. B cells, T cells and NK cells were excluded in a CD19, CD20, CD3, CD56 dumb channel (Lin^{-}). Monocytes and dendritic cells were excluded based on HLA-DR expression. Neutrophils (and monocytes and NK cells) were excluded using CD16 expression. MDSCs were identified as $CD33^{+}CD11b^{+}$ cells, and gMDSC and mMDSC were identified using CD15 and CD14. Numbers in the plots represent percentage gated cells.

MDSC gating

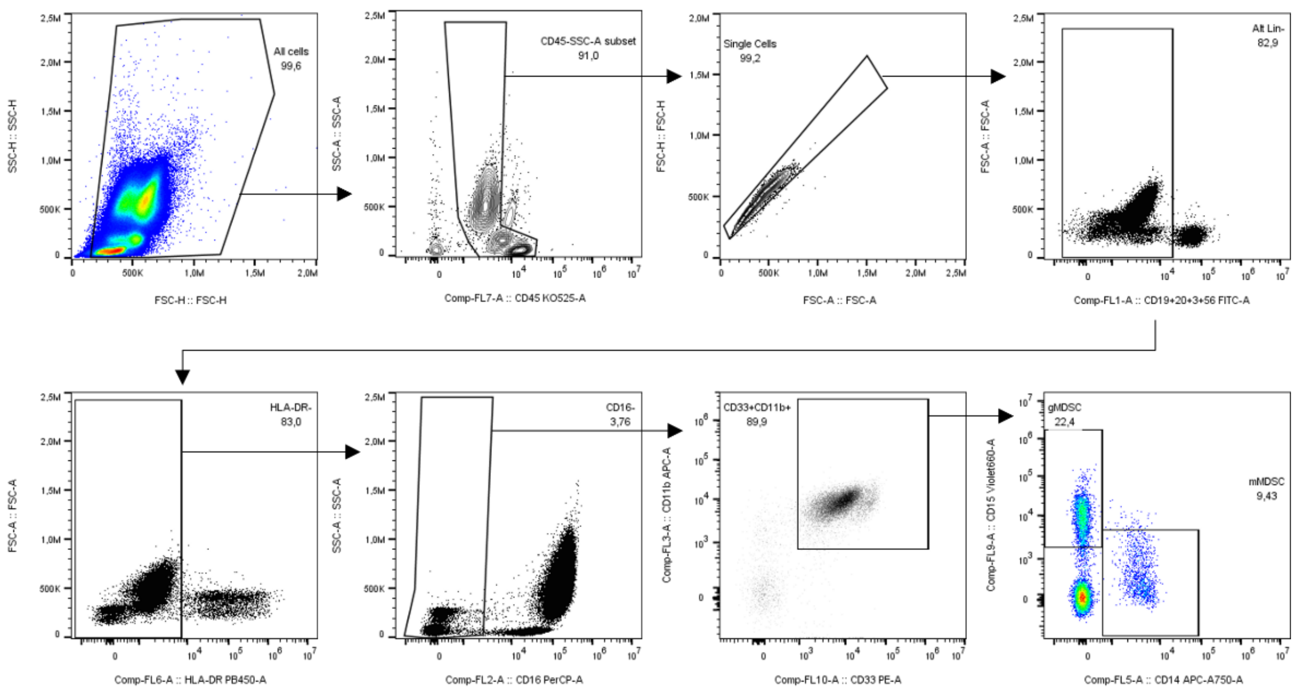

Supplementary figure S4: DC gating strategy, related to figure 4

Cells were identified based (SSC) and forward scatter (FSC). Single cells were gated based on FSC-H vs. FSC-A. Monocytes and dendritic cells were identified as HLA-DR^{+} cells. Monocytes and B cells were excluded using a CD14/CD19 dumb channel (Lin^{-}). cDC2 cells were identified as CD1c^{+}CD303^{-}. cDC1 cells were identified as CD1c^{-}CD303^{-}CD141^{+} cells. pDC were identified as CD1c^{-}CD303^{+} cells. All DC subpopulations were subsequently analyzed for expression of PD-L1. Numbers in the plots represent percentage gated cells.

DC gating

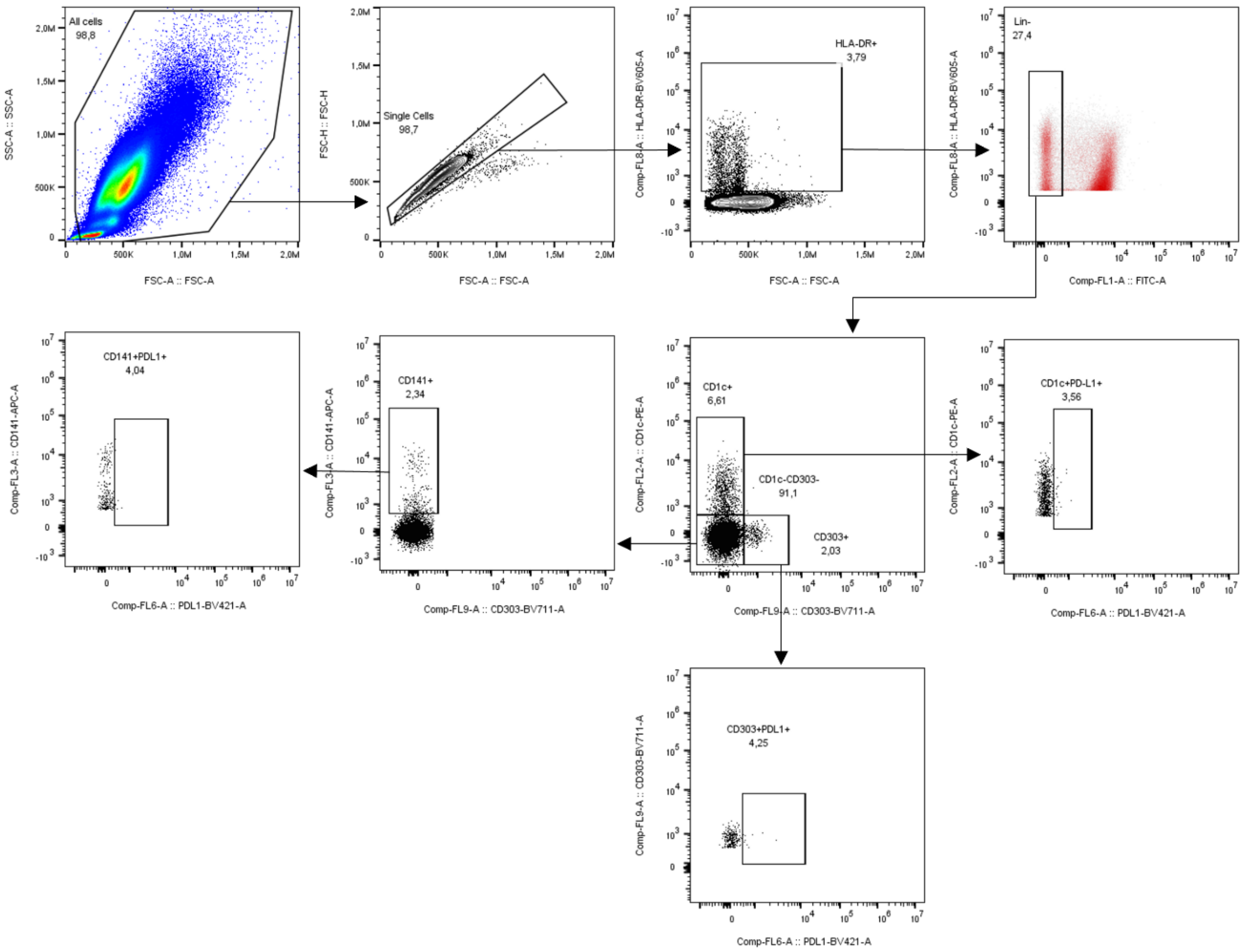

Supplementary table S1: Representativeness of study participants. Related to table 1.

**Cancer type**	Metastatic pancreas cancer (PC)
**Considerations related to:**	
Sex	Both sexes could be included, 6 male and 2 female patients were included by chance. The sex distribution is approximately equal in the general population of PC-patients.
Age	Patients >18 years of age could be included. The median age was 61 years which is younger than the median age in the general population of PC-patients of around 70 years.
Race/ethnicity and ancestry	All patients were Danish citizens and Caucasian. In Denmark almost all citizens are of this ethnicity.
Geography	In Denmark, about 1000 new cases of PC are diagnosed per year and about the same number die from the disease. Patients were treated at a single institution but were recruited from the whole country.
**Overall representativeness of this study**	The age distribution of our study is lower than the average age of PC in the literature.<br>The sex distribution (mostly men) is different from the sex distribution of PC-patients in the literature.<br>All patients were Caucasian which reflects that in Denmark almost all citizens are of this ethnicity. This was not an exclusion criterium, however.

Supplementary table S2. Immune cell population markers. Related to figure 4.

Anti	Fluorochrome	Clone, isotype	Producer
CD3	FITC	SK7, mIgG1, κ	BioLegend
CD3	V500	SP34-2, mIgG1,	BD Horizon
CD4	BV711	SK3, $mIgG_1$, κ	BD Horizon
CD4	FITC	RPA-T4, $mIgG_1$, κ	BD Pharmingen
CD8	StarBright 515	LT8, mIgG1	Bio-Rad
CD8	BV711	RPA-T8, $mIgG_1$, κ	BD Horizon
PD-1	APC	MIH4, $mIgG_1$, κ	BD Pharmingen
CTLA-4	BV421	BNI3, $mIgG_{2a}$, κ	BD Horizon
TIM-3	PE	7D3, mIgG1, κ	BD Pharmingen
LAG-3	BV605	T47-503, $mIgG_1$, κ	BD Pharmingen
CD45	V500	HI30, mIgG1, κ	BD Horizon
CD45RA	BV421	5H9, $mIgG_1$, κ	BD Optibuild
CCR7	BV605	2-L1-A, mIgG1, κ	BD Horizon
CD19	FITC	HIB19, $mIgG_1$, κ	BD Pharmingen
CD20	FITC	L27, mIgG1, κ	BD
CD56	PE-Cy7	CMSSB, $mIgG_1$, κ	Thermo Fisher Invitrogen
HLA-DR	APC-H7	L243, $mIgG_{2a}$, κ	BD Pharmingen
HLA-DR	BV605	G46-6, $mIgG_{2a}$, κ	BD Horizon
CD33	PE	WM53, mIgG1, κ	BD Pharmingen
CD11b	APC	ICRF44, $mIgG_1$, κ	BD Pharmingen
CD14	BV421	MϕP9, mIgG2b, κ	BD Horizon
CD14	FITC	M5E2, mIgG2a, κ	BD Pharmingen
CD16	BV785	3G8, ImgG1, κ	BioLegend
CD15	BV650	W6D3, mIgG1, κ	BioLegend
CD303	buv563	V24-785, $mIgG_{2b}$, κ	BD Optibuild
CD1c	PE	F10/21A3, $mIgG_1$, κ	BD Pharmingen
CD141	APC	1A4, $mIgG_1$, κ	BD Pharmingen
PD-L1 (CD274)	BV421	MIH1, $mIgG_1$, κ	BD Horizon

Supplementary table S3: Cytokine changes during trial, related to figure 5
All listed cytokines were upregulated. No significantly downregulated cytokines were discovered.

	Expression	Function
PD-1	Activated T-cells	Inhibitor of T-cell function
OX40	Broad array of lymphoid and non-lymphoid cells	Involved in development of effector and memory T-cells. Inhibitory effect on Tregs
MMP12	Secreted	Involved in tissue remodeling and repair
CRTAM	Activated T-cells	Increase IFNgamma secretion by CD8+ T-cells
CCL20	Secreted	Chemotactic on lymphocytes
IL8	Secreted	Proinflammatory cytokine
CCL23	Secreted	Chemotactic on monocytes, neutrophils and resting T-cells
