# Supplementary material for: Pembrolizumab followed by irreversible electroporation of a liver metastasis in pancreatic cancer patients
Source: iScience. 2024 Sep 24;27(10):111026. doi: 10.1016/j.isci.2024.111026 (PMC11602522; doi:10.1016/j.isci.2024.111026)
Supplement: Data S1. Study protocol, related to STAR methods [file mmc2.zip › EPIC-1 protocol.pdf]

A phase II-study of electroporation potentiated immunotherapy in liver metastatic pancreatic cancer (EPIC-1)

Version 1.5 (04.03.2021)
EudraCT: 2020-004536-22
VEK-id: N-20200085
Region Nordjylland-id: 2021-018

Participating centers:
Department of Oncology and Department of Gastrointestinal Surgery
Clinical Cancer Research Center
Aalborg University Hospital
Hobrovej 18-22, 9000 Aalborg, Denmark
+45 97 66 12 85

Data center:
Clinical Research Unit
Department of Oncology
Aalborg University Hospital
Hobrovej 18-22, 9000 Aalborg, Denmark
+45 97 66 12 85

Principal investigators signature: ____

Morten Ladekarl, professor, MD, DMSc

Sponsor signature: ____

Ole Thorlacius-Ussing, professor, MD, DMSc

TABLE OF CONTENTS

Table of Contents 2
Protocol summary 4
Introduction 11
Background 12
Checkpoint inhibitors in pancreatic cancer 12
Irreversible electroporation 12
Irreversible electroporation and immunotherapy 13
Choice of intervention 14
Safety considerations 14
Irreversible elctroporation 14
Pembrolizumab 15
Biomarkers of immunological effects 16
Tumor biomarkers 18
Blood biomarkers 18
Methods 20
Study aim and hypothesis 20
Primary outcomes: 20
Secondary outcomes: 20
Study design 21
Study population and sample size 21
Inclusion criteria: 21
Exclusion criteria: 22
Recruitment of study subjects 23
Historical Data 24
Study interventions 24
Immunotherapy 25
IRE procedure 25
Treatment duration and follow-up 26
Non-therapeutic interventions and assessments during trial 28
Imaging 28
Safety assessment and clinical evaluation 28
Biopsies 29

Blood sampling 29
Patient reported outcomes 30
Biomarker studies 31
Tissue analyses 31
Histopathology 31
Gene expression analysis 31
Tumor DNA analysis 32
Blood analyses 32
Cellular immunology 32
Cytokines 33
Cell-free tumor DNA analyses 33
Statistical methods 34
Perspectives 35
Ethical presentation 36
Potential benefits, risks and side-effects by participation 36
Ethical considerations 36
Project Management 38
Timeline and study feasibility 38
Data analysis plan 38
Data management and security 38
Biobanks 39
Good clinical practice 39
Project group and management structure 39
Publications and authorship 40
Financial aspects and conflicts of interest 40
References 41
Appendix A: Preclinical studies 46
Appendix B: Product resumé (Danish) 48
Appendix C: Adverse event registration and exemptions 215
Appendix D: Vurdering og håndtering af bivirkninger relateret til immunterapi med check-point hæmmere (anti-CTLA-4, anti PD-1 og anti-PD-L1) 218
Appendix E: Genes included in the TruSight™ Oncology 500 and TruSight™ Oncology High Throughput panel 253

PROTOCOL SUMMARY

**Title:**	**A phase II Study of Electroporation Potentiated Immunotherapy in Liver Metastatic Pancreatic Cancer**
**Acronym:**	**EPIC-1** (**E**lectroporation **P**otentiated **I**mmunotherapy in **C**ancer – **1**.st study)
**Study Sponsor:**	Ole Thorlacius-Ussing, Aalborg University Hospital
**Protocol version:**	Version 1.5
**Principal investigator:**	Morten Ladekarl, Aalborg University Hospital
**Study site:**	Department of Oncology & Department of Gastrointestinal Surgery, Aalborg University Hospital
**Investigators and clinical study coordinators:**	Rasmus Virenfeldt Flak, Department of Gastrointestinal Surgery, Aalborg University Hospital<br>Laurids Østergaard Poulsen, Department of Oncology, Aalborg University Hospital<br>Susy Shim, Department of Oncology, Aalborg University Hospital
**Other collaborators:**	INTERNAL:<br>*Surgery:*<br>Mogens Tornby Stender<br>*Radiology:*<br>Gintare Naujokaite<br>Olga Tcacenco<br>*Pathology:*<br>Alkwin Wanders<br>*Blood sampling and biobank management:*<br>June Lundtoft Køjborg<br>*Genetic analysis:*<br>Inge Søkilde Pedersen<br>Henrik Krarup<br><br>EXTERNAL:<br>*Pathology and gene expression analysis:*<br>Sönke Detlefsen<br>*Immunology:*<br>Ralf Agger

	Emil Kofod-Olsen
**Funding organizations**	The Danish Cancer Society
**Protocol Phase:**	Phase II
**Indication:**	Adult patients in WHO performance status 0-1 with liver metastatic pancreatic cancer intolerant to or progressing on first or further lines of chemotherapy
**Aim:**	To investigate the efficacy and safety of combined irreversible electroporation (IRE) and checkpoint inhibition in liver metastatic pancreatic cancer
**Hypothesis:**	IRE treatment of a single liver metastasis with concomitant checkpoint inhibition can elicit a systemic objective response
**Objectives:**	**Primary outcomes:**<br>1. Objective response rate (ORR) according to RECIST 1.1 (in patients receiving at least one dose of pembrolizumab, IRE and an evaluable CT-scan)<br>2. Serious adverse reaction (SAR) rate according to CTCAEv5 (in patients receiving at least one dose of pembrolizumab)<br>**Secondary outcomes:**<br>3. Median overall survival (mOS)<br>4. Median progression-free survival (mPFS)<br>5. ORR (in patients that have received at least one dose of pembrolizumab and an evaluable CT-scan)<br>6. Clinical benefit ratio (defined as objective response or stable disease for at least 8 weeks during treatment in patients receiving at least one dose of pembrolizumab, IRE and an evaluable CT-scan)<br>7. Serum CA 19-9 response (response defined as a decrease of at least 20 % observed during treatment in patients with elevated CA 19-9 at baseline)<br>8. Survival rate after 6 and 12 months<br>9. Progression-free survival rate after 6 and 12 months<br>10. Mean difference (multiple timepoints) in perceived quality of life (for subscales of EORTC QLQ C-30 v3 including Global health status / QoL (QL2), Physical functioning (PF2), Fatigue (FA), Nausea and vomiting (NV), Pain (PA), Appetite loss (AP) and Diarrhea (DI)<br>11. Mean difference (multiple timepoints) in nutrition status (numerical score of PG-SGA-SF)<br>12. Difference in peripheral blood immune cell composition and function during active treatment and follow-up compared to baseline

	13. Histological tumor regression grade in tumor biopsies after pembrolizumab and after pembrolizumab + IRE compared to baseline<br>14. Difference in tumor RNA expression patterns after pembrolizumab and after pembrolizumab + IRE compared to baseline<br>15. Difference in peripheral blood levels of cytokines during active treatment and follow-up compared to baseline<br>16. Difference in (histological) tumor infiltrating leukocyte (TIL) pattern after pembrolizumab and after pembrolizumab + IRE compared to baseline<br>17. Difference in peripheral blood cell-free tumor DNA concentration immediately after IRE compared to before IRE<br>18. Difference in the number of detectable cell-free DNA mutations immediately before and after IRE compared to DNA mutations found in tumor tissue samples from the IRE ablated metastasis<br>19. Adverse event rate (CTCAEv5, all grades)
**Study design:**	Prospective open-label, single-arm clinical trial
**Planned sample size:**	16 evaluable patients (patients not receiving at least one dose of pembrolizumab and IRE, and a CT-scan evaluation are replaced by others)
**Total number of centers:**	1
**Selection criteria:**	Inclusion criteria:<br>1. Histologically verified pancreatic adenocarcinoma, based on either a biopsy of the primary tumor or a metastasis<br>2. One liver metastasis treatable by IRE (as determined by MDT at Aalborg University Hospital)<br>3. One tumor lesion suited for repeated biopsy by transcutaneous core needle (preferably another lesion than that used for IRE)<br>4. At least one measurable lesion (RECIST version 1.1) other than the liver metastasis to be treated by IRE<br>5. At least one course of chemotherapy for metastatic or inoperable disease discontinued due to treatment failure or intolerance<br>6. Performance status 0-1<br>7. ASA ≤ 3<br>8. ≥ 18 years of age<br>9. Written and orally informed consent<br>10. Sufficient available histological tumor material stored in biobank or obtainable by new biopsy<br>11. Patient acceptance of collection of blood samples for translational research and two additional biopsies during treatment<br>12. Adequate bone marrow function, liver function, and renal function (within 7 days prior to enrollment):

	a. Neutrophils (ANC) ≥ 1.5 x 10^9/l<br>b. Platelet count ≥ 100 x 10^9/l<br>c. Hemoglobin ≥ 6 mmol/l<br>d. Plasma bilirubin ≤ 1.5 x ULN<br>e. Plasma alanine transaminase (ALAT) < 5 x ULN<br>f. Plasma creatinine ≤ 1.5 x ULN<br>g. INR ≤ 1.5<br><br>Exclusion criteria:<br>1. Underlying medical disease not adequately treated (e.g. poorly regulated diabetes and symptomatic cardiac disease)<br>2. Prior or current autoimmune disorder with risk of serious toxicity during treatment with checkpoint inhibitor<br>3. Acute myocardial infarction, cerebral vascular attack, transient ischemic attack or subarachnoid hemorrhage within 6 months from start of treatment<br>4. Previous reception of allogeneic stem cells or solid organ donation<br>5. Active infection requiring systemic therapy within 7 days prior to treatment initiation<br>6. Positive HIV, HBV, and HCV test results (prior testing or new testing in patients at risk)<br>7. Active psychiatric disease or history of drug or alcohol abuse affecting participation<br>8. Allergy to active substance or any of the auxiliary agents, including known severe allergy to anesthetic agent, paralytic agent or any of the equipment used during treatment<br>9. Expected need for systemic corticosteroid or other systemic immunosuppressive drug during the course of this clinical trial. A low dose of e.g. prednisone ≤ 10 mg/day is permitted for maximally 7 consecutive days<br>10. Coexisting malignant disease, except non-melanoma skin cancer<br>11. Symptomatic or untreated CNS metastases<br>12. Liver cirrhosis Child Pugh >A<br>13. Pregnant or breast-feeding patients. For women of childbearing potential, a negative pregnancy test (minimum sensitivity 25mIU(hCG)/ml) is mandatory prior to inclusion and every month during the trial<br>14. Women of childbearing potential not willing to use effective methods of contraception* during treatment and for 6 months after the end of treatment. Male patients with a fertile partner are also required to secure effective methods of contraception*<br>15. Previous immunotherapy

	16. Patients referred from a hospital outside of Denmark<br>17. Major dilation of veins or bowel obstructing the needle path<br>18. Persistent atrial fibrillation<br>19. Metal objects (e.g. biliary SEMS) within 5 cm of ablation target<br>20. Cardiac pacemaker or ICD, that cannot be safely disconnected during IRE treatment<br><br>*Definition available in full text
**Study interventions:**	Irreversible electroporation + pembrolizumab
**Main parameters of safety:**	Continuous evaluation and registration of adverse events according to ICH-GCP definition

Study procedures:

Patients with metastatic pancreatic cancer with at least one liver metastasis and one other measurable tumor lesion can be included in the study if all the inclusion- and none of the exclusion criteria are met. Patients will all have gone through at least one course of first-line palliative chemotherapy and stopped treatment due to progressing or intolerability.

Because of the multidisciplinary nature of this intervention, inclusion in the trial will require a prior evaluation of feasibility by the pancreas MDT conference at Aalborg University Hospital.

Included patients will undergo baseline testing as described below. Pembrolizumab will be administered intravenously every 6 weeks at a fixed dose of 400 mg according to routine practice. Between the first and second treatment with pembrolizumab, patients will be treated with IRE of a single liver metastasis, in order to enhance the immune response against the patients' cancer cells. Pembrolizumab will be administered for 6 months or until intolerability or progression. The active treatment will be stopped prematurely if the participant withdraws consent or if the treatment responsible physician assesses that the continued treatment will pose a threat to the safety of the participant.

During the active treatment, patients will be submitted to clinical assessment, bloodwork and CT-scans every 2 months. Patients with progressing disease will be offered further treatment (if feasible) according to standard practice.

After the treatment has ended patients will be followed per protocol for 6 months until progression or permanent, serious deterioration of performance status precluding further active oncological treatment (PS >2). During follow-up, patients will be submitted to clinical assessment, bloodwork and CT-scans every 2 months. The patients will also be asked to complete an electronic survey, to investigate how the treatment affects quality of life, nutritional status and pain perception.

Any serious adverse events (SAEs) during treatment will lead to further investigation and will be reported to the sponsor.

**Informed consent:**	Oral and written informed consent must be obtained prior to any study-specific procedures.
**Screening:**	Baseline screening must be performed within two weeks before start of treatment and includes:<br>- Eligibility assessment

	○ Medical history, concurrent illness<br>○ Physical examination, height and weight, vital functions including blood pressure and pulse<br>○ Eastern Cooperative Oncology Group (ECOG) performance status<br>○ ASA score<br>○ Pregnancy test (women in fertile age)<br>○ HIV and Hepatitis B/C test (patients at risk)<br>○ Blood test including B-hemoglobin, B-leucocytes, B-leucocyte types, B-thrombocytes, P-c reactive peptide, P-Na+, P-K+, P-magnesium, P-calcium (corrected), P-creatinine, P-LDH, P-bilirubin, P-alkalic phosphatase, P-ALAT, P-amylase (pancreatic type), P-albumin, P-cortisol, P-TSH, P-T4, P-glucose, P-CA 19-9, Quatiferon test, P-Troponine-T, INR and APTT<br>○ Electrocardiogram (ECG)<br>○ CT scan of chest and abdomen with liver specific contrast protocol<br>○ Evaluation of quality of archival biopsy material<br>- Additional baseline testing for comparison during treatment/follow-up<br>○ Blood sample for research (including flow cytometry and cytokines)<br>○ Baseline tumor biopsy (if archival biopsy material is insufficient (for histology, immunohistochemistry and RNA analysis (Nanostring))<br>○ Quality of life assessment using EORCT QLQ-C30<br>○ Nutritional status using PG-SGA-SF
**Assessments during treatment:**	- Clinical assessment (with registration of adverse events)<br>- CT scans of chest and abdomen with liver specific contrast protocol<br>- Standard blood tests (B-hemoglobin, B-leucocytes, B-leucocyte types, B-thrombocytes, P-c reactive peptide, P-Na+, P-K+, P-magnesium, P-calcium (corrected), P-creatinine, P-LDH, P-bilirubin, P-alkalic phosphatase, P-ALAT, P-amylase (pancreatic type), P-albumin, P-cortisol, P-TSH, P-T4, P-glucose, P-CKMB, P-Troponin-T and P-CA 19-9)<br>- Blood sample for research (including ctDNA, flow cytometry and cytokines)<br>- Biopsies day 10 (±5 days) and day 52 (±5 days) (for histology, immunohistochemistry and molecular analysis)<br>- ECOG performance status<br>- Quality of life assessment using EORCT QLQ-C30<br>- Nutritional status using PG-SGA-SF

**Assessments during follow-up:**	- Clinical assessment (with registration of adverse events)<br>- CT scans of chest and abdomen with liver specific contrast protocol<br>- Standard blood tests (B-hemoglobin, B-leucocytes, B-leucocyte types, B-thrombocytes, P-c reactive peptide, P-Na+, P-K+, P-magnesium, P-calcium (corrected), P-creatinine, P-LDH, P-bilirubin, P-alkalic phosphatase, P-ALAT, P-amylase (pancreatic type), P-albumin, P-cortisol, P-TSH, P-T4, P-glucose, P-CKMB, P-Troponin-T and P-CA 19-9)<br>- Blood sample for research (including flow cytometry and cytokines)<br>- ECOG performance status<br>- Quality of life assessment using EORCT QLQ-C30<br>- Nutritional status using PG-SGA-SF
**Analysis plan:**	Parameters of safety will be monitored continuously. Participants will undergo protocoled safety assessments prior to IRE and prior to administration of pembrolizumab (see detailed description below).<br>Analysis of long-term effects will be carried out after all patients have completed at least 6 months of follow-up or have progressed. Oncological results will be compared to results of matched patients (patients in ECOG PS 0-1 with liver metastatic pancreatic cancer treated with 2nd or further lines of chemotherapy at our clinic and in the published literature.
**First patient included and duration of study:**	It is expected that the first patient will be included in Q2 2021.<br>Based on the institutional throughput of possible eligible patients, the inclusion should be completed within two years. Given an additional 6 months of treatment and 6 months of follow-up + possible delays, the study period is tentatively set to 01.01.2021 – 31.12.2025.

INTRODUCTION

Pancreatic cancer (PC) is the fourth leading cause of cancer-related death and the incidence is rising[1]. The only possibility of cure is surgical resection, but only 20% of patients are eligible for surgery at diagnosis[2]. Metastatic disease is diagnosed in approximately half of new PC patients and the majority of patients initially presenting with non-metastatic disease develops metastases during the disease course[3]. The long-term prognosis of metastatic PC is dismal even with modern chemotherapy[4]. The median overall survival (mOS) in these patients range from <6 months to approximately 1 year, based on data from clinical trials[5]. However, survival is lower in real-world samples. A Danish nationwide unselected cohort study by our group, comprising all newly diagnosed PC patients between 2011 to 2016, revealed a mOS of 6.9 months in patients receiving chemotherapy as initial therapy[6]. Only around half of patients receiving palliative chemotherapy undergo second-line chemotherapy, which only rarely induces an objective response and results in an estimated mOS of 4.4 months[5,7]. Evidently, PC patients need more efficient treatment with a greater impact on survival.

Immunotherapy with checkpoint inhibitors (CPIs) has shown impressive results in several tumor types, where chemotherapy has been largely ineffective, but unfortunately this is not the case in PC. Several preclinical trials indicate, however, that irreversible electroporation (IRE), a novel ablative treatment, may have the potential to increase the efficacy of immune CPIs (see section **Irreversible electroporation and immunotherapy**) by reverting immunologically “cold” tumors to “hot”. To date, only one phase 1b clinical trial examining this combination has been published[8]. However, this trial was performed in patients with locally advanced PC (LAPC) and thus is not able to evaluate whether IRE can potentiate the immunological response in untreated tissue, i.e. metastases.

The primary aim of this study is to investigate the efficacy and safety of combined IRE and CPIs in metastatic PC. If successful, the results of this trial will also have implications for studies of this combined treatment in other cancer types.

In addition to evaluating the impact of treatment on tumor burden and disease outcome, we will conduct translational biomolecular research, which will give insight into the possible immunostimulatory mechanisms of IRE. Patient reported outcomes will be monitored throughout the trial to estimate the implications of the therapy on quality of life and nutritional status.

BACKGROUND

Checkpoint inhibitors in pancreatic cancer

Recently, immune CPIs have shown efficacy and changed the treatment paradigm of multiple cancers, including malignant melanoma, non-small cell lung cancer and renal cell carcinoma. Unfortunately, in PC the trials published at this point are unsuccessful, and only the 1% of patients with tumors displaying microsatellite instability or mismatch repair deficiency seem to benefit from treatment[9–11]. Royal et al investigated ipilimumab, a CTLA4 antagonist, in PC but the trial was terminated early because none of twenty patients responded to the therapy[12]. Le et al randomized previously treated PC patients to receive either high dose ipilimumab or high dose ipilimumab + GVAX, a cancer vaccine based on allogeneic pancreatic tumor cells transfected with a GM-CSF gene[13]. Although the difference in mOS (3.6 months versus 5.7 months) was not statistically significant, the authors found a significant difference in 1-year survival rate favoring the ipilimumab + GVAX group. Even so, only two out of 15 patients in this group displayed stable disease and none had a response. Aglietta et al conducted a nonrandomized dose escalation trial of tremelimumab, another CTLA4 antagonist, with concurrent gemcitabine in 38 patients with metastatic PC[14]. In this trial, two patients achieved a partial response. The entire cohort reached a mOS of 7.4 months. Finally, Brahmer et al conducted a dose escalation trial of a PD-L1 antagonist in a mixed patient cohort including 14 PC patients[15]. None of the included PC patients achieved an objective response.

There are several potential mechanisms contributing to the lack of effect of immunotherapy in PC[16]. One possible factor is the poor tumor antigenicity of pancreatic adenocarcinomas. This is related to the low mutational rate of PC with an average of 45 somatic mutations, which negatively affects the production of neoantigens and immune recognition[16]. In contrast, melanomas, where immunotherapy is highly efficient, harbor an average of 135 somatic mutations. Another unique characteristic of PC is the desmoplastic, immunosuppressive and hypoxic microenvironment. The pancreatic tumor microenvironment comprises cancer-associated fibroblasts, endothelial cells, extracellular matrix proteins and immunosuppressive cells including myeloid-derived suppressor cells, regulatory T-cells (Tregs) and tumor-associated macrophages. In contrast, effector T cells are few. Hence, it is likely that the poor results observed with immunotherapy in PC so far is primarily due to the low leucocyte infiltration displayed by most tumors and its composition. In contrast, many of the tumor types where immunotherapy has shown good results displays a high degree of leucocyte infiltration[16], and are characterized as immunologically “hot”.

Irreversible electroporation

IRE is a novel non-thermal ablative technique that is currently being implemented and studied for treatment of LAPC, liver tumors, prostate cancer, kidney cancers and other. In contrast to thermal ablation, IRE induces cell death by a combination of apoptosis and necrosis with little effect on extracellular matrix. IRE works by applying high-current short duration electrical pulses to the treated tissues. This, in term, creates multiple nanoscale pores in the cell membrane allowing uncontrolled influx and efflux of ions and macromolecules leading to loss of homeostasis. IRE can be applied either percutaneously, laparoscopically or during open surgery. In either case, patients undergoing the procedure must be under general anesthesia with deep neuromuscular blockade. Furthermore, to prevent cardiac arrythmias, the energy delivery must be synced to the refractory period of the electrocardiogram.

In our institution we have previously conducted an uncontrolled phase 1 study of IRE in LAPC[17], and we are currently running a phase 2 trial in the same patient group (VEK Project ID: N-20190013; https://clinicaltrials.gov/ct2/show/NCT04093141). To date, we have treated approximately 40 patients with percutaneous ultrasound-guided IRE of the pancreas and one patient with an open technique. In this cohort 20% of patients experienced major adverse events and one patient died as a result of the treatment, which is on par with larger international trials[17]. Adverse events after IRE were generally manageable and many were not directly attributable to IRE, but most likely resulted from the disease. The most problematic IRE-related adverse event when applied to the pancreas was abscess formation (in 12%), as this often temporarily precluded patients from receiving subsequent chemotherapy. We expect the rate of adverse events to be smaller with IRE used for liver ablation in the current study, as most studies of IRE in liver tumors report low levels of adverse events (see section **Safety considerations**). This is most likely due to the environment of the ablation. Crucially, abscess formation only rarely happens after IRE in liver tumors. Currently, we are the only Danish hospital conducting active research in IRE.

Irreversible electroporation and immunotherapy

Several preclinical experiments have demonstrated that IRE treatment has immunogenic properties[18–21]. In a study by Zhao et al, IRE was combined with CPIs in a murine PC model[18]. The researcher demonstrated that response after IRE + CPIs far outperformed IRE only, CPIs only and radiation + CPIs. Other trials have shown IRE to be more immunogenic than radiofrequency ablation and cryoablation[19–21]. For further information about the results from preclinical trials see **Appendix A: Preclinical studies**.

At this point in time, only one clinical trial of combined IRE and CPIs has been published. O'Neill et al performed an uncontrolled phase 1b clinical trial in 10 patients with LAPC receiving IRE with concurrent nivolumab[8]. Patients with non-progressive disease on chemotherapy could be included. Patients were treated with IRE during open surgery in conjunction to palliative surgical procedures on a case-by-case basis. Nivolumab (240mg) was started on postoperative day one to five and was continued every two weeks for 8 weeks in total or until disease progression or unacceptable toxicity. Seven of the ten patients experienced a total of 11 grade 3/4 toxicities (CTCAEv4) during the first 90 days after IRE. Six grade 3/4 toxicities where related to the surgical intervention/IRE while no grade 3/4 toxicities where attributed to nivolumab. No mortalities and no dose-limiting toxicities were reported. Two patients only received the first nivolumab dose, because of surgical complications. Both patients experience recurrence after 3 months, while the remaining 8 patients had a mean PFS of 6.8 months. Median OS was 18 months for the entire cohort.

Lin et al performed a non-randomized study of IRE with or without concurrent allogenic natural killer (NK) cell therapy in 71 PC patients comprising both stage III and IV[22]. The investigators found, that in both disease stages, allogenic NK therapy improved the disease control rate (RECIST 1.1) and the mOS. However, the results did not display whether a synergistic effect with IRE was present, as there was no control group receiving NK therapy only.

In conclusion, preclinical and clinical results suggest that IRE could have a large impact on the efficacy of CPIs in PC. To date, only one ongoing phase 2 trial examining this combination in metastatic PC is registered on http://www.clinicaltrials.gov/ct2/show/NCT04212026.

Choice of intervention

Based on the background evidence presented above we believe that the best choice of immunotherapy for combination with IRE is a PD1 inhibitor. Specifically, pembrolizumab is chosen because of the well-known safety profile of the drug and long treatment intervals (compared to nivolumab). The comparatively longer treatment interval (up to six weeks between administrations) allow for less in-person visits during the study which is less intrusive to the participants and keep down the running costs. Currently, less visits are also desirable due to the global COVID-19 pandemic, to minimize the risk of infection at hospitals.

Pembrolizumab, like all other currently available CPIs, are not approved for treating PC. Thus, the main intervention of the current study is pembrolizumab (from a regulatory perspective). IRE treatment, while not best practice, is applied using a CE-marked device, and thus does not require any formal monitoring besides from ethical committee approval. However, because the study is designed to explore the effect of the treatment combination, formal requirements (i.e. reporting and monitoring of adverse events and reactions) will be upheld for both interventions. The most common adverse reactions resulting from either intervention will not be reported immediately to the sponsor. Please see details below.

Safety considerations

Irreversible elctroporation

Multiple clinical trials have shown IRE to be safe in the treatment of liver tumors, even when applied in locations normally associated with high-risk of complications after radiofrequency ablation (RFA). Additionally, we expect IRE of liver metastases to be less prone to complication when compared to pancreatic ablations. Adverse events observed after IRE of the liver include abdominal pain, nausea, vomiting, intraabdominal bleeding, abscess formation, bile duct fistula, needle tract seeding, ascites and, in rare cases, death. The reported rate of major complications ranges from 0% to 7.5% (**Table 1**). In total, one fatality has been reported as a result of IRE of liver tumors[23]. The patient died due to liver failure after being treated for hepatocellular carcinoma. The patient had poor liver function and poor general health prior to the intervention. In the largest study to date regarding safety, 5.2% of patients experienced major complications and 13.8% experienced minor complications[23]. While the available studies show that safety of IRE ablation of liver tumors is on par with RFA, only two non-randomized comparative studies have been done. These do not consider that IRE can be performed on comparative high-risk tumors, and therefore results should be interpreted with caution. In a controlled trial conducted by Bhutiani et al, 55 patients were treated for hepatocellular carcinoma with either IRE or microwave ablation (MWA)[24]. In this study 27% of IRE patients experienced adverse events (any grade) compared to 76% in the MWA group. Verloh et al conducted a case-control study of 47 patients treated with IRE and 117 patients treated with RFA or MWA[25]. This study found that 2.1% of IRE patients experienced major complications compared to 3.4% in the other group.

Authors	Year	Study design	N	Histology	Safety results
**Scheffer et al**[26]	2014	Ablate and resect	10	CRLM	No IRE-related serious AEs observed.
**Hosein et al**[27]	2014	Retrospective	29	CLRM	2 patients (7%) experienced serious AEs

**Langan et al**[28]	2017	Retrospective	40	CLRM, HCC, other	3 patients (7.5%) experienced IRE-related complications. Overall 14 patients (35%) experienced any complications.
**Frühling et al**[29]	2017	Prospective single arm	30	CRLM, HCC, other	1 patient (3.3%) experienced a major complication. 6 patients (20%) experiences minor complications.
**Eisele et al**[30]	2014	Prospective single arm	14	CRLM, HCC, CCA	No immediate IRE-related AEs observed.
**Stillström et al**[31]	2019	Prospective single arm	42	CRLM, HCC, CCA, other	2 patients (5%) experienced major complications. 8 patients (15%) experienced minor complications
**Zeng et al**[32]	2017	Prospective single arm	14	HCC	No IRE-related major complications observed.
**Distelmaier et al**[33]	2017	Prospective single arm	29	CRLM, HCC, CCA, other	No IRE-related major complications observed.
**Beyer et al**[34]	2017	Retrospective	35	CRLM, HCC, other metastases	No IRE-related complications observed.
**Dollinger et al**[35]	2015	Retrospective	56	HCC, CRLM, CCA, other metastases	Major complications occurred after 6 procedures (7.1%). Minor complications occurred after 16 procedures (18.8%).
**Cannon et al**[36]	2012	Retrospective	44	CRLM, HCC, other metastases	IRE-related complications occurred after 3 procedures (6.3%).
**Eller et al**[37]	2015	Prospective single arm	14	CRLM, HCC, CCA, other	Major complications occurred in 1 patient (7.1%). Minor complications occurred in 3 patients (21.4%).
**Giorgio et al**[38]	2019	Prospective single arm	16	HCC, CCA, other	No major complications occurred.
**Sutter et al**[23]	2017	Retrospective	58	HCC	Major complications occurred in 3 patients (5.2%). One patient died due to complications. Minor complications occurred in 8 patients (13.8%).
**Cheung et al**[39]	2013	Prospective single arm	11	HCC	No IRE-related major complications observed.
**Sugimoto et al**[40]	2015	Prospective single arm	5	HCC	No IRE-related major complications observed.
**Bhutiani et al**[24]	2016	Non-randomized controlled trial	IRE (30), MWA (25)	HCC	27% of IRE patients experienced AEs compared to 76% of MWA patients.
**Verloh et al**[25]	2019	Case-control study	IRE (47), RFA/MWA (117)	HCC	1 patient (2.1%) of IRE patients experienced major complication compared to 4 patients (3.4%) in the RFA/MWA group.
**Beyer et al**[41]	2016	Prospective	20	HCC, other	No complications observed

Table 1: Clinical trials reporting safety outcomes after IRE in liver ablations

Abbreviations: IRE = Irreversible electroporation; MWA = Microwave ablation; RFA = Radiofrequency ablation; CRLM = Colorectal liver metastases; HCC = Hepatocellular carcinoma; CCA = Cholangiocarcinoma; AE = Adverse event.

Pembrolizumab

CPIs are routinely used for treatment of numerous cancers. The associated safety profile is very well known. In the current study, pembrolizumab will be administered as a fixed dose (400 mg) intravenously every 6 weeks. The dosing is equal to approved treatment regimens in other cancers. The most common

serious side effects are induction of autoimmune disease, which has been seen in most or all organs of the body, and rare allergic reactions. Autoimmune disease is manageable in most cases but may lead to treatment pause or abandonment, and toxicities resulting in death have been seen. The most common side effects are: Anemia, hypothyroidism, appetite loss, headache, shortness of breath, coughing, diarrhea, stomach pain, nausea, vomiting, constipation, itching (pruritus), rashes, muscle pain, joint pain, fatigue, edemas and fever. The full list of known side-effects of pembrolizumab is available in **Appendix B: Product resumé (Danish)**.

To date only one study of combined liver ablation and CPIs has been published to our knowledge[42]. In this study 32 patients with HCC was treated with tremelimumab (anti-CTLA4) and either RFA or trans-arterial chemoembolization (TACE). It is not possible to differentiate between the two treatment modalities in the study, but of the 25 patients receiving combination therapy, 3 patients experienced toxicities requiring discontinuation of treatment. Combinations of IRE on liver tumors and CPIs has not previously been used in humans, but we do not expect an increase in toxicity compared to that of each modality used as single treatment. As described above (see section **Irreversible electroporation and immunotherapy**), only one study has combined IRE and CPIs in PC treatment[8]. This study found no serious adverse events attributable to treatment with nivolumab, only events related to the IRE treatment, which was confounded by simultaneous palliative procedures.

Biomarkers of immunological effects

Studies of biomarkers of the immunological effects of IRE alone or of combined treatment with immune CPIs and IRE are few. In a mouse model (Zhao et al) the researchers performed flow cytometry and showed that mice treated with a combination of IRE and antiPD1 had significantly more CD8+ cells and a higher CD8+/Treg ratio in tumor samples[18]. In the experiment comparing IRE + antiPD1 with radiation therapy (10Gy) + antiPD1, the intratumoral CD8+ count, while higher than the control group, was only half of the IRE group. Similarly, the CD8+/Treg ratio was significantly higher in the IRE group. Long term surviving mice where sacrificed and splenocytes where collected. Splenocytes where incubated with KRAS tumor lysate. Using an ELISPOT assay, researcher found that IFNγ secreting splenocytes where 2.3 times higher in long-term surviving mice compared to treatment naive mice. Similarly, the frequency of CD4+ and CD8+ memory cells were increased 2.1- and 5.7-fold, respectively, in splenocytes of long-term survivors when compared to treatment naive mice. IRE was also found to induce the release of danger-associated molecular patterns (DAMPs). Scheffer et al conducted an immunological study in 10 patients undergoing IRE for LAPC[43]. In this study, peripheral blood samples were subjected to flowcytometry before IRE and twice during follow-up after 2 weeks and 3 months. The results showed a transient decrease in Tregs two weeks after IRE. At the same time, expression of PD1 was significantly increased in both CD4+ and CD8+ cells. These effects were consistent across 9/10 patients. Expression of other immunological checkpoints (LAG3, TIM3 and CTLA4) remained low and did not change after IRE. No effect was observed regarding frequency or activation of myeloid cell lines. Furthermore, tumor antigen specific response was measured by subjecting T cells to the tumor antigen Wilms tumor 1 (WT1). In this analysis, samples from two patients showed an increased IFNγ response compared to pre-IRE samples from the same patients. At the same time, samples from two other patients showed a de-novo IFNγ response. A positive post-IRE IFNγ response was linked to increased survival (p=.055). Beitel-White et al conducted a flowcytometric study in 8 LAPC patients undergoing IRE[44]. The authors found a correlation between the changes in electrical impedance during IRE treatment and the

immune cell composition in peripheral blood taken 24 hours after treatment. Larger changes in impedance was associated with decreased concentration of CD4+ CD25+ and CD4+ CD25+ FoxP3+ cells. Furthermore, a trend towards longer survival in patients with at least 2% decrease in the measured immune cells was noted. Finally, O'Neill et al performed an uncontrolled phase 1b clinical trial of 10 patients with LAPC receiving IRE with concurrent nivolumab[8]. The researchers found a statistically significant rise in T-effector memory cell concentration by day 90. No difference was found in CD4+ T cells, naïve T cells or T-central memory cells.

Biomarker	Potential mechanisms/clinical applicability
Tumor cell:	
PD-L1	Expression associated with worse outcome<br>Does not necessarily predict response to CPI<br>Dynamic expression in time and space<br>Different cut-off values and polymorphism<br>Reproducibility problems
Mutational burden (TMB) and neoantigen burden	High TMB associated with response to CPI<br>No cut-off value
Mismatch repair (MMR) status/microsatellite instability (MSI)	MSI-high associated with response to CPI<br>Approved by FDA across different tumor types
Somatic mutations	TP53, STK11, EGFR
CD38/adenosine	Inhibits CD8+ T cell function through adenosine receptor signaling
β-catenin/Wnt-pathway	Pathway mediating immune avoidance
MET	MET-mutation associated with less response to CPI<br>MET overexpression associated with favorable outcome with CPI
Tumor microenvironment:	
Extracellular matrix	AXL, EZH2, FAP, MMP, PIK3CA, VEGF
Inhibitory metabolism	Hypoxia and glycolysis, IL-10, TGF-β, adenosine, LDH, COX, lactate, arginine, glutamine, PGE2
IL-6 family cytokines	Involved in inhibition of apoptosis
Immune system:	
Tumor infiltrating lymphocytes (TILs)	Favorable prognostic factor
IFNγ	High baseline expression associated with better survival after CPI
T-cell receptor diversity	TCR clonal expansion associated with response to CPI
Immunosuppressive factors	Tregs, MDSCs, IDO
Host environment:	
Gut microbiota	Diversity associated with better response to CPI
HLA genotype	Lack or downregulation make tumor cells invisible to T cells
Immunosenescence	Decreased cytotoxic capability and increased inflammation in elderly patients<br>Loss of CD27 and CD28<br>Expression of TIM-3 and CD57 on T-cells
Basic laboratory tests	LDH, absolute lymphocyte count, neutrophil-to-lymphocyte ratio, CRP, eosinophil count

Table 2: Suggested biomarkers for immunotherapy with CPIs

Many biomarkers of effect of CPIs have been suggested and evaluated in clinical studies (**Table 2**), however, few have proven clinically useful.

In the current study, we intend, in patients with metastatic PC, to assess the immunological effects of IRE-treatment of liver metastases combined with pembrolizumab by repeated tumor biopsies and serial blood tests. It is plausible that manipulations, such as IRE, if leading to an increase in predictive biomarkers for response to immunotherapy may lead to increased anti-tumor effect of the combined treatment.

Tumor biomarkers

Although it is a poor biomarker, expression of PD-L1 assessed through IHC in tumor tissue has been the major initial molecular determinant of clinical benefit from the CPIs targeting PD-1 and PD-L1. Expression of PD-L1 is considered to be induced in response to interferon-γ (INFγ) released by activated T cells. We hypothesize that IRE leads to increased presentation of neoantigens to the immune system and, possibly, the induction of a pro-inflammatory, permissive microenvironment and therefore increased PD-L1 expression in tumor tissue.

Assessments of tumor infiltrating lymphocytes (TIL) in multiple cancer types have generally shown that increased numbers of cytotoxic T cells, natural killer cells, mature dendritic cells (DCs) and M1 macrophages are associated with improved prognosis, Tregs are associated with poorer prognosis, whereas inconclusive results have been found for neutrophils and B cells[45]. In PC, studies on lymphocytes[46–48], macrophages[46,49] and neutrophils[46] suggest that adequate activation of the *in situ* immune system is important for the prognosis after resection. Studies of the ratio of neutrophils to lymphocytes in peripheral blood hint that the balance of tumor-promoting and anti-tumoral immune cells in the circulation is a marker of outcome of PC[50–53].

Assessments of TILs are of particular interest in patients treated with CPIs. For example, studies of the PD-L1 inhibitor atezolizumab have shown predictive value of PD-L1 expression on tumor infiltrating immune cells. Response to atezolizumab also correlated with high expression of effector T-cell and INFγ associated gene signatures in tumor tissue.

Several gene signatures have been developed for assessments of tumor immune response. Among these, the Tumor Inflammation Signature (TIS) may provide both quantitative and qualitative information about the immune environment within a tumor, reporting on the composition of an immune infiltrate as well as the functional status of T cells. Key biological activities that shape the immune response to the tumor are measured, including dMMR, antigen presentation, tumor cell proliferation, cytotoxic activity, glycolysis, and oncogenic pathways.

Blood biomarkers

Most studies of blood biomarkers evaluate circulating T cell populations in CPI-treated cancer patients have been done on cohorts of melanoma or non-small cell lung cancer (NSCLC) patients treated with ipilimumab or pembrolizumab. As described in detail below, studies have included memory and effector T cells, exhaustion phenotype T cells, Tregs, the granulocyte/lymphocyte ratio, myeloid-derived suppressor cells and DCs.

A number of studies found altered composition of circulating memory and effector subpopulations in CPI-treated cancer patients. In a study on NSCLC patients who received nivolumab (n = 22), the ratio of central memory (T_{CM}) and effector T cells (T_{EFF}) was found to be high in patients with prolonged progression free survival[56]. CM CD8 T cells were also found to be elevated in partial and complete responders in a study on melanoma patients co-treated with radiotherapy and ipilimumab (n = 9)[57]. A study on ipilimumab-treated melanoma patients (n = 137), found that increased effector memory T cells (T_{EM}) was associated with

increased OS, whereas high baseline level of terminally differentiated effector T cells (T_{TE}) was associated with poor response[58]. This is supported by a study on ipilimumab- (n = 22) or pembrolizumab- (n = 9) treated advanced stage melanoma, which found reduced baseline CD45RO$^+$CD8$^+$ T cells in non-responders treated with ipilimumab, although no significant association was observed in the pembrolizumab-treated patients[59].

Many cancer patients harbor tumor specific CD8+ T cells in circulation. However, these T cells obviously fail to eradicate tumors, and the T cells display an exhausted phenotype, due to prolonged antigen stimulation. The level and clinical correlation of exhausted circulating T cells (T_{EX}) has been investigated in stage IV melanoma patients treated with pembrolizumab (n = 29)[60]. Here it was found that the ratio of T_{ex} cell (Ki67$^+$PD-1$^+$CTLA-4$^+$Tim-3$^+$CD38$^+$) reinvigoration to tumor burden, correlated with clinical outcome. Patients who responded showed post treatment expansion of the T_{ex} population, whereas non-responders showed expansion of the T_{eff} population. Another study on a cohort of metastatic melanoma patients, treated with pembrolizumab (n = 16), found that expression of exhaustion markers LAG-3 and Tim-3 was significantly higher in non-responders[61].

Tregs are known to play a prominent role in tumor resistance to treatment. The amount of tumor infiltrating Tregs has been investigated in numerous studies, with varying outcome. A few studies have investigated circulating Tregs, defined as CD4$^+$FoxP3$^+$CD25$^+$. A study on late stage melanoma patients treated with ipilimumab (n = 95), found an association between low Treg numbers (at week 12) and disease control/survival, supporting a notion that ipilimumab strongly targets Tregs, although the study did not correlate this to Treg levels at baseline[62]. This was done in a study on ipilimumab-treated melanoma patients (n = 209), where they found that a high baseline Treg count was associated with a positive clinical outcome[63]. Taken together, this supports a hypothesis that patients with a high Treg baseline will tend to be patients, where Tregs play a prominent role in the tumor immune escape, and thus these patients are likely to respond to CPI treatment.

Changes in the ratio of the absolute blood granulocyte count to the absolute lymphocyte count has been shown to be a nonlinear predictor of patient outcomes in immunotherapy of advanced cancers[64].

Circulating myeloid-derived suppressor cells (MDSC) have been shown in a number of publications to correlate with a poor outcome of treatments of cancer patients with chemotherapy as well as several types of immunotherapy[65].

DCs control whether an antigen is ignored, whether active tolerance develops or whether a specific immune response is mounted. DCs activate both CD4+ and CD8+ T cells and potently shape the type and strength of the immune mechanisms evoked[67]. DCs are thus essential, also for adaptive immune responses against cancer. Three main populations of DCs are present in blood: Conventional DCs type 1 and type 2 (cDC1, cDC2) and plasmacytoid DCs (pDC). In cancer patients, DCs in the tumor environment and in the draining lymph nodes are often induced to become tolerogenic rather than immunogenic. Changes in the numbers of circulating DCs have been demonstrated in some types of cancer, but the subtypes of DCs have rarely been systematically monitored during immunotherapy of cancer[68,69].

METHODS

Study aim and hypothesis

The aim of the study is to investigate the efficacy and safety of combined IRE and checkpoint inhibition in metastatic pancreatic cancer. The hypothesis is that IRE relieves resistance to checkpoint inhibition and thus is able to provoke a systemic anticancer immune response.

The current study is designed to explore whether combining IRE and pembrolizumab yields an acceptable effect size (ORR) and an acceptable treatment-related toxicity to warrant further investigation given the increased invasiveness and economical expense of the intervention compared to standard treatment. Standard second-line chemotherapy is usually associated with response rates below 15% in patients with metastatic pancreas cancer[54]. It is the opinion of the investigators that the current study should result in an ORR of at least 25% to be clinically relevant.

Primary outcomes:

1. Objective response rate (ORR) according to RECIST 1.1 (in patients receiving at least one dose of pembrolizumab, IRE and an evaluable CT-scan)
2. Serious adverse reaction (SAR) rate according to CTCAEv5 (in patients receiving at least one dose of pembrolizumab)

Secondary outcomes:

3. Median overall survival (mOS)
4. Median progression-free survival (mPFS)
5. ORR (in patients that have received at least one dose of pembrolizumab and an evaluable CT-scan)
6. Clinical benefit ratio (defined as objective response or stable disease for at least 8 weeks during treatment in patients receiving at least one dose of pembrolizumab, IRE and an evaluable CT-scan)
7. Serum CA 19-9 response (response defined as a decrease of at least 20 % observed during treatment in patients with elevated CA 19-9 at baseline)
8. Survival rate after 6 and 12 months
9. Progression-free survival rate after 6 and 12 months
10. Mean difference (multiple timepoints) in perceived quality of life (for subscales of EORTC QLQ C-30 v3 including Global health status / QoL (QL2), Physical functioning (PF2), Fatigue (FA), Nausea and vomiting (NV), Pain (PA), Appetite loss (AP) and Diarrhea (DI)
11. Mean difference (multiple timepoints) in nutrition status (numerical score of PG-SGA-SF)
12. Difference in peripheral blood immune cell composition and function during active treatment and follow-up compared to baseline
13. Histological tumor regression grade in tumor biopsies after pembrolizumab and after pembrolizumab + IRE compared to baseline
14. Difference in tumor RNA expression patterns after pembrolizumab and after pembrolizumab + IRE compared to baseline
15. Difference in peripheral blood levels of cytokines during active treatment and follow-up compared to baseline

16. Difference in (histological) tumor infiltrating leukocyte (TIL) pattern after pembrolizumab and after pembrolizumab + IRE compared to baseline
17. Difference in peripheral blood cell-free tumor DNA concentration immediately after IRE compared to before IRE
18. Difference in the number of detectable cell-free DNA mutations immediately before and after IRE compared to DNA mutations found in tumor tissue samples from the IRE ablated metastasis
19. Adverse event rate (CTCAEv5, all grades)

Study design

Single center, single arm prospective clinical trial

Study population and sample size

The planned sample size is 16 patients treated with at least one dose of pembrolizumab and IRE and evaluable for response. Using the Wald z test for one-sample proportions, with a one-sided alpha of .05 the power of the study is ≈ 75%, using the assumption: ORR_{CPI} = 0.1% and $ORR_{CPI+IRE\ (true)}$ > 25%. ORR_{CPI} is assumed based on the prior publications of CPI only in metastatic PC[12,13,15].

Inclusion will continue until 16 patients have received at least one dose of pembrolizumab, IRE and reached the first imaging response evaluation timepoint (two months) in order to fulfil the primary objective of the study.

Inclusion criteria:

1. Histologically verified pancreatic adenocarcinoma, based on either a biopsy of the primary tumor or a metastasis
2. One liver metastasis treatable by IRE (as determined by MDT at Aalborg University Hospital)
3. One tumor lesion suited for repeated biopsy by transcutaneous core needle (preferably another lesion than that used for IRE)
4. At least one measurable lesion (RECIST version 1.1) other than the liver metastasis to be treated by IRE
5. At least one course of chemotherapy for metastatic or inoperable disease discontinued due to treatment failure or intolerance
6. Performance status 0-1
7. ASA ≤ 3
8. ≥ 18 years of age
9. Written and orally informed consent
10. Sufficient available histological tumor material stored in biobank or obtainable by new biopsy
11. Patient acceptance of collection of blood samples for translational research and two additional biopsies during treatment
12. Adequate bone marrow function, liver function, and renal function (within 7 days prior to enrollment):
    h. Neutrophils (ANC) ≥ 1.5 x 10^9/l
    i. Platelet count ≥ 100 x 10^9/l
    j. Hemoglobin ≥ 6 mmol/l

k. Plasma bilirubin ≤ 1.5 x ULN
l. Plasma alanine transaminase (ALAT) < 5 x ULN
m. Plasma creatinine ≤ 1.5 x ULN
n. INR ≤ 1.5

Exclusion criteria:

1. Underlying medical disease not adequately treated (e.g. poorly regulated diabetes and symptomatic cardiac disease)
2. Prior or current autoimmune disorder with risk of serious toxicity during treatment with CPI
3. Acute myocardial infarction, cerebral vascular attack, transient ischemic attack or subarachnoid hemorrhage within 6 months from start of treatment
4. Previous reception of allogeneic stem cells or solid organ donation
5. Active infection requiring systemic therapy within 7 days prior to treatment initiation
6. Positive HIV, HBV, and HCV test results (prior testing or new testing in patients at risk)
7. Active psychiatric disease or history of drug or alcohol abuse affecting participation
8. Allergy to active substance or any of the auxiliary agents, including known severe allergy to anesthetic agent, paralytic agent or any of the equipment used during treatment
9. Expected need for systemic corticosteroid or other systemic immunosuppressive drug during the course of this clinical trial. A low dose of e.g. prednisone ≤ 10 mg/day is permitted for maximally 7 consecutive days
10. Coexisting malignant disease, except non-melanoma skin cancer
11. Symptomatic or untreated CNS metastases
12. Liver cirrhosis Child Pugh >A
13. Pregnant or breast-feeding patients. For women of childbearing potential, a negative pregnancy test (minimum sensitivity 25mIU(hCG)/ml) is mandatory prior to inclusion and every month during the trial
14. Women of childbearing potential not willing to use effective methods of contraception* during treatment and for 6 months after the end of treatment. Male patients with a fertile partner are also required to secure effective methods of contraception*
15. Previous immunotherapy
16. Patients referred from a hospital outside of Denmark
17. Major dilation of veins or bowel obstructing the needle path
18. Persistent atrial fibrillation
19. Metal objects (e.g. biliary SEMS) within 5 cm of ablation target
20. Cardiac pacemaker or ICD, that cannot be safely disconnected during IRE treatment

* The following is considered an effective method of contraception: Intrauterine device or hormonal stimulation preventing ovulation, i.e. birth control pills, hormonal implant, prolonged release injection, transdermal prolonged release patch or vaginal hormonal ring. A stable sterile sexual partner or abstinence is also acceptable. To be considered infertile the following definition is used: Postmenopausal (absence of menstruation for at least 12 months), surgical sterilization (vasectomy/bilateral salpingectomy, hysterectomy, or bilateral oophorectomy). In special cases these rules may be deviated from by the investigator, however any deviation must be registered in the patient chart and the case report form.

Recruitment of study subjects

Patients are either recruited at our institution (from the oncological out-patient clinic) or referred from other oncological departments treating patients with PC in Denmark. A flow diagram of the inclusion process is available in **Figure 1**.

Patients with metastatic PC, who progresses on or are intolerable to palliative chemotherapy, will be given oral and written information about the trial at the Department of Oncology and invited to enter the screening phase. Information from the electronic patient chart from patients who enter the screening phase will be passed on to the investigator and this will be noted in the patient chart. All study candidates will be discussed at the pancreas MDT conference in Aalborg. If the candidate meets the inclusion criteria (as determined at the MDT conference), the candidate may proceed to the inclusion phase, and will be seen in the out-patient clinic at the Department of Oncology, Aalborg University Hospital. A medical history and physical examination will be conducted by a member of the gastrointestinal cancer. Patients meeting the selection criteria will receive further oral information about study-specific procedures. Patients will be asked to give a written informed consent. Informed consent will take place in a quiet room without interruption. Patients will be asked to read the information in entirety at home before signing, if they have not already done so. All patients are invited to bring a relative or other entrusted person, if they choose to. All patients will also meet the study-responsible study-coordinator, who will supply patients with additional information and contact information.

Written information is available in appendix.

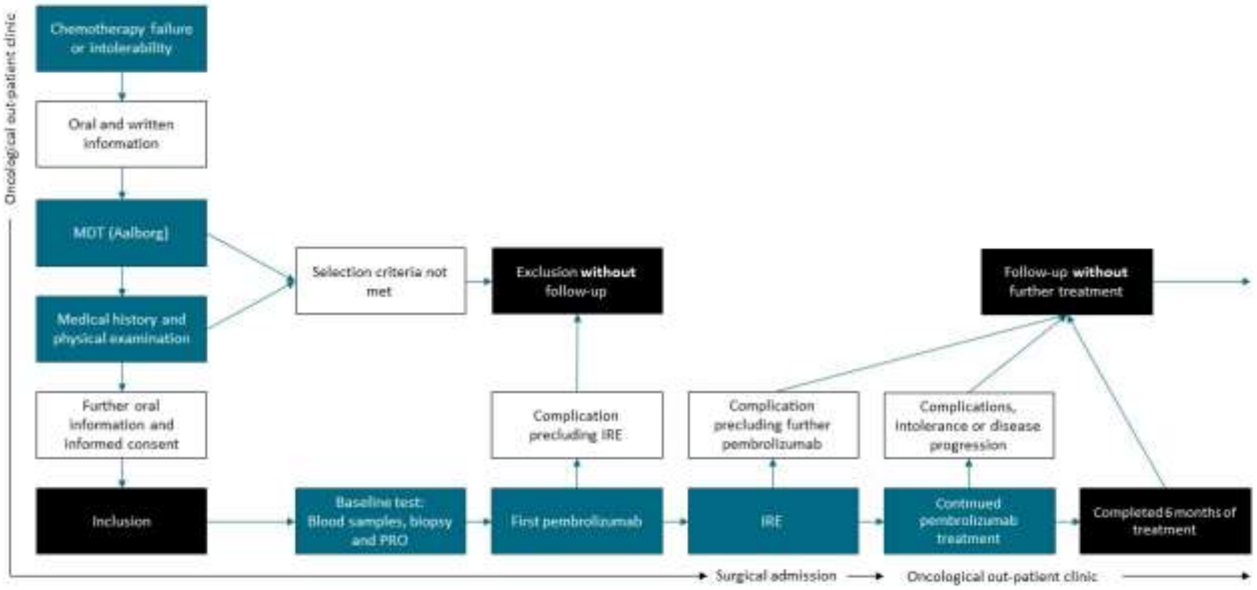

Figure 1: Patient recruitment process

Abbreviations: MDT = Multidisciplinary team; PRO = Patient reported outcomes; IRE = Irreversible electroporation.

Historical Data

OS, ORR and other clinical outcome data will be compared to those of a cohort of similar patients (PS 0-1, liver metastatic pancreas cancer) treated with chemotherapy beyond 1st line at the Department of Oncology at Aalborg University Hospital, and to reports of similar patients in the literature. A systematic retrospective examination of electronic patient records during the period 1.1.2010-31.12.2020 will be conducted to identify possible patients for comparison.

Study interventions

Patients entering the trial will receive treatment in accordance with the timeline outlined in **Figure 2**.

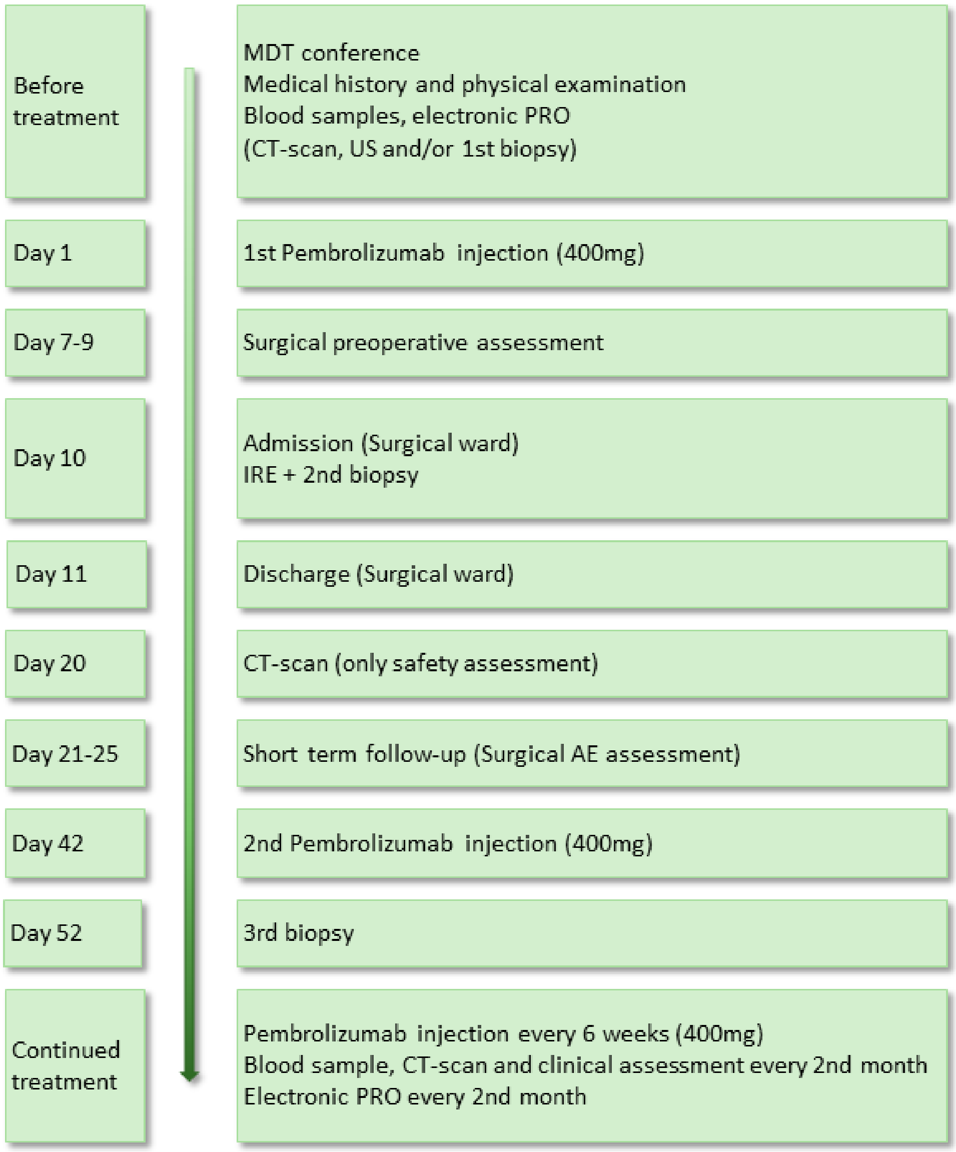

Figure 2: Treatment schedule

Notes: CT-scan, US and/or 1st biopsy will only be performed if it has not been performed prior to inclusion as part of the standard workup or if biopsy material is insufficient for further studies or if CT/US scan is more than 1 month old or of insufficient quality.

Abbreviations: MDT = Multidisciplinary team; PRO = Patient reported outcomes; CT = Computed tomography; US = Ultrasound scan; IRE = Irreversible electroporation.

Immunotherapy

Pembrolizumab is a readily available drug, that is in use at the Department of Oncology. The drugs for the trial will be acquired through the University Hospital Pharmacy. Pembrolizumab will be administered intravenously every 6 weeks at a fixed dose (400mg). Drug administration will be performed in accordance with department standard operating procedures.

IRE procedure

The IRE procedure will be carried out between the first and second administration. Prior to the procedure the patients will be assessed by a surgeon from the Hepato-Pancreatic-Biliary (HPB) team at Aalborg University Hospital to ensure that it is safe to proceed with the IRE procedure.

The IRE procedure will be carried out under general anesthesia with ultrasound or CT guidance either percutaneously or during laparotomy. The treatment is carried out using the commercially available NanoKnife™ system (Angiodynamics, Queensbury, NY). Needle spacing and electrical pulse delivery will be performed as described below. The guidelines are adapted from the recommendation by Martin et al.[55].

Periprocedural anesthesia:

- Will be carried out by experienced anesthesiologists.
- Deep neuromuscular blockade (zero twitches using train of four (TOF) monitoring) will be utilized during procedure.
- Electrical pulse delivery will be synced to the refractory phase of the patient ECG using a commercial device.

IRE probe placement and optimal needle selection:

- The IRE procedure is executed by a skilled interventional radiologist with prior experience in percutaneous IRE.
- Probes are places between 1.0-2.6 cm apart (ideally 1.5-2.0 cm), bracketing the tumor and ideally 0.5-1.0 cm margin of healthy tissue.
- 2 probes are used.
- Probes will be placed in parallel with a maximum deviation of 5 degrees.
- Probes may be rearranged to cover a larger area.

Setup and troubleshooting:

- Initially a tissue conductivity test is performed.
- 20 pulses per pair of electrodes are given with a pulse length of 90 µsec and an electrical field strength of 1,500 V/cm.
- An amperage level of 20-35 A must be reached for each probe pair.

Treatment completion and evaluating adequate increase in current:

- After tissue connectivity test yields results within the desired range a baseline current is recorded to assess treatment success.
- The treatment is carried out using a setting of 90 pulses per probe pair.
- Throughout the procedure, the apparatus is checked for high current warnings.
- If necessary one or several pull backs are performed, and the process is repeated.

After the IRE procedure, patients will be transferred to the surgical ward for at least 24 hours of observation. Upon discharge from the surgical ward the patients will be referred to a CT-scan and blood tests approximately 10 days after the IRE procedure. Patients will be scheduled for a phone call a few days after the CT-scan (termed short-term follow-up in Figure 2) to ensure that no serious complications have occurred as a result of the IRE treatment. Any adverse events will be registered.

Treatment duration and follow-up

Patients will be followed in the oncological out-patient clinic throughout the trial. Pembrolizumab will be administered for 6 months (5 series total) or until intolerability or progression. During the active treatment, patients will be submitted to CT-scans every 2 months. Patients with confirmed progressing disease will be offered further treatment according to standard practice.

Active treatment will be stopped at any point if the participant withdraws consent or if the treating physician assesses that the continued treatment poses a risk to the health of the patient.

After the treatment has ended, patients will be followed per protocol for 6 months or until permanent, serious deterioration of performance status precluding further active oncological treatment (PS >2). During follow-up, patients will be submitted to clinical assessment, bloodwork and CT-scans every 2 months. The assessment plan during active treatment and follow-up is outline below (**Table 3**):

	Screening	Inclusion	Before treatment	Active treatment													Follow-up		
				Cycle 1	PreOP assessment	IRE	First postOP day	Surgical AE assessment	Cycle 2	Biopsy	2 months visit	Cycle 3	4 months visit	Cycle 4	Cycle 5	6 months visit	2 months after discont.	4 months after discont.	6 months after discont.
Eligibility assessment (MDT conference)	x																		
Medical history, informed consent		x																	
Clinical assessment		x		x	x	x		x	x			x		x	x		x	x	x
Vital signs		x		x	x				x			x		x	x		x	x	x
ECOG performance status		x		x					x			x		x	x		x	x	x
ASA score		x																	
Pregnancy test (fertile women)		x							x			x		x	x				
CT scan			(x)					x			x		x			x	x	x	x
AE registration		x			x			x	x			x		x	x		x	x	x
Blood sample standard (type)		A	D	C	B		C	C	C	D	C	C	C	C	C	C	C	C	C
Blood sample research			Flow-D + Bio		Flow-D + Bio	ctDNA x2	Flow-D + Bio			Flow-D + Bio	Flow-F + Bio		Flow-F + Bio			Flow-F + Bio	Flow-F + Bio	Flow-F + Bio	Flow-F + Bio
Tissue biopsies			(x)			x				x									
EORTC QLQ-C30 questionnaire			x								x		x			x	x	x	x
PG SGA-SF questionnaire			x								x		x			x	x	x	x
Survival information + subsequent treatment																			x

Table 3: Follow-up schedule

Notes: Blood sample types are described in ***Table 4.*** *Abbreviations: ECOG = Eastern Cooperative Oncology Group; ASA = American Society of Anesthesiologists; AE = Adverse event; CT = Computed tomography; EORTC = European Organization for Research and Treatment of Cancer; QLQ-C30 = Quality of Life Questionnaire – Core 30; PG SGA-SF = Scored Patient-Generated Subjective Global Assessment – short form; IRE = Irreversible electroporation; Flow-D = flowcytometry direct analysis; Bio = biobank sample; ctDNA = circulating tumor DNA analysis (frozen for post-hoc analysis); Flow-F = Flowcytometry (frozen for post-hoc analysis).*

Non-therapeutic interventions and assessments during trial

Imaging

The planned imaging in the study is contrast enhancement CT-scans of the thorax and abdomen. The scan protocols in the study will be identical to standard institutional practices for CT scans used in diagnosing and monitoring disseminated PC. Imaging will be carried out every 2 months and will be assessed using RECIST 1.1 by a radiologist with experience in systematic response evaluation[56]. An additional CT scan will be performed approximately 10 days after the IRE procedure to rule out any complications, that may preclude the patients from receiving any more pembrolizumab treatments.

Safety assessment and clinical evaluation

Patients will be asked to attend the oncological outpatient clinic every 2 months to receive information about the newest CT-scan and standard test results.. Additionally, patients will see a physician on the day of each pembrolizumab treatment. Patients will be specifically asked about adverse events and given a physical examination, during the visit. Furthermore, the physician will assess the patients Eastern Cooperative Oncology Group (ECOG) performance status to assess whether it is safe to proceed with treatment.

Adverse events will be assessed, and registered, after the IRE treatment, prior to all pembrolizumab infusions and every 2 months during the follow-up period. The severity of events will be scaled using the NCI-CTCAE, version 5.0[57] and according to the ICH-GCP guidelines.

The ICH-GCP guidelines defines and categorizes adverse events in the following way:

Adverse event (AE): Any ‘untoward medical occurrence’ (unfavorable sign, symptom, laboratory finding, disease) happening during the study period.

Adverse reaction (AR): Any AE were a causal relationship to the study intervention cannot be disproven.

Serious adverse event (SAE): Any serious AE (definition of serious below)

Serious adverse reaction (SAR): Any serious AR (definition of serious below)

Suspected unknown serious adverse reaction (SUSAR): Any SAR considered unexpected because its nature or severity is not consistent with the applicable product information

In order to be considered serious according to the ICH-GCP the following criteria is used.

Any event or reaction resulting in:

Death

Life threatening illness

Hospital admission or prolonging of admission

Permanent/severe handicap or inability to work

Congenital disease or birth defects

Detailed information on the AE registration process is available in **Appendix C**: Adverse event registration and exemptions.

Any immunological side effects will be treated in accordance with best practice (more information in **Appendix D**: Vurdering og håndtering af bivirkninger relateret til immunterapi med check-point hæmmere (anti-CTLA-4, anti PD-1 og anti-PD-L1)). Any IRE related adverse events will also be treated in accordance with best practice.

Serious adverse events will be reported to the study sponsor and the monitoring authorities within the timeline defined by the ICH-GCP guidelines. Any serious adverse event not mentioned in the exceptions will be reported to the sponsor as soon as possible and under no circumstances later than 24 hours after learning about them. Any and all suspected unexpected serious adverse reactions (SUSARs) will be reported to the sponsor and the authorizing authorities immediately in accordance with ICH-GCP.

Biopsies

Biopsies are taken from a malignant lesion at three different, predefined, timepoints for monitoring of treatment and anti-cancer immunological effects. The first biopsy (at baseline) is omitted if enough archival tissue remains to perform the analysis. If the original sample has been taken at another hospital, this sample will be acquired for evaluation after the patient has consented to enter the trial. The second biopsy is taken during the IRE treatment (under general anesthesia) to avoid unnecessary burdens to the patient. The third biopsy is taken after the second pembrolizumab administration under local anesthesia. All samples are performed by an experienced interventional radiologist using US guidance. Ideally, three Tru-Cut™ biopsies are obtained at each timepoint. The tissues samples are handled in accordance with institutional practice but are marked as research samples. One tissue sample, taken prior to the IRE ablation, is taken from the metastasis designated for ablation and designated for tumor DNA sequencing for comparison to blood sequencing results (outcome 18) (more information in sections **Tissue analyses** and **Cell-free tumor** DNA). The other samples are taken from another lesion (ideally the same for all three samples). A standard operating procedure detailing the sampling method is provided to the physicians performing the biopsies. Proper precautions are taken to minimize the risk of infection, bleeding and tumor seeding. Tissue samples will be saved in a biobank for bulk analysis (for more information, see section **Biobanks**).

Blood sampling

Blood samples are drawn by a dedicated medical laboratory technologist using best-practice techniques. All samples, where research samples are taken, will be drawn at Aalborg University Hospital. Standard tests are analyzed by the biochemical department shortly after sampling. Results from standard blood tests will be accessed through the clinical system (LABKA) and registered in the research database. Standard tests will be segregated into three different categories, type A and B (**Table 4**), dependent on the sampling schedule (**Table 3**). Additional samples for immunological analyses will be transferred to Aalborg University Laboratory of Immunology for immediate flowcytometric analysis, and a fraction will be frozen and kept in a biobank for bulk analysis of cytokine concentrations, proteomics, DNA etc. (for more information, see section **Biobanks**) The total amount of blood drawn varies dependent on which type is drawn. Type A totals 16 ml while type B totals 12. The research samples comprise 42 ml in total per sample (18 ml for flow cytometry and 24 ml for the biobank). In addition, two 40 ml blood samples are taken at immediately before and after IRE respectively and are frozen in the biobank. The first sample is taken after the patient

has been sedated but before needle insertion. The second sample is taken within one hour after the last pulse has been given. These samples are used for analysis of cell free DNA (cfDNA) (outcome 17 & 18). More information in section **Cell-free tumor DNA analyses**.

**Analysis**	Type A	Type B	Type C	Type D
B-Leucocytes	x	x	x	x
B-Leucocytes (types)	x	x	x	x
B-Hemoglobin	x	x	x	
B-Thrombocytes	x	x	x	
P-Na+	x	x	x	
P-K+	x	x	x	
P-Mg+	x	x	x	
P-Ca++ (corrected)	x	x	x	
P-Creatinine	x	x	x	
P-Lactate Dehydrogenase	x	x	x	
P-Billirubin	x	x	x	
P-Alcalic Phosphatase	x	x	x	
P-Alanine Transaminase	x	x	x	
P-Amylase (pancreatic type)	x	x	x	
P-Albumin	x	x	x	
P-Cortisol	x	x	x	
P-Thyroid Stimulating Hormone	x	x	x	
P-Thyroxin (T4)	x	x	x	
P-Glucose	x	x	x	
P-Creatinine Kinase-MB		x	x	
P-Troponin T	x	x	x	
P-CA 19-9	x	x	x	
P-C Reactive Peptide (CRP)	x	x		
International Normalized Ratio (INR)	x	x		
Activated Partial Thromboplastin Time (APTT)	x	x		
Quatiferon test	x			

Table 4: Standard blood analysis used

*Notes: An overview of the different blood analysis packages utilized during the study. All displayed analyses will be performed immediately after sampling. For an information on the timing of the different packages please see **Table 3**.*

Patient reported outcomes

Patient reported outcomes (PRO) will be assessed at different timepoints throughout the study.

Quality of life will be measured using the scientifically and linguistically validated Danish version of the European Organisation for Research and Treatment of Cancer (EORTC) Quality of Life Questionnaire (QLQ)-C30 version 3. Raw scores will be calculated according to the manual. Items will be grouped in: Global

health status (range 0 - 100, high is better), functional scales (range 0 - 100, high is better) and symptom scales (range 0 - 100 low is better). Differences in each scale during the trial we be calculated separately.

Nutritional status will be assessed using the linguistically validated Danish version of The Scored Patient-Generated Subjective Global Assessment (PG-SGA) developed by Pt-Global. The short form includes only patient reported measures and will be combined into a single numerical score according to the manual (range 0 - 37, low score is better).

Biomarker studies

Factors concerning the tumor, the microenvironment and the immune system will be explored, and concordance between signatures and variables in blood and tissue will be investigated. Associations of variables with treatment and outcome of treatment will be assessed. The specific analyses are described below.

Tissue analyses

Histopathology

Subpopulations of infiltrating immune cells will be estimated in FFPE tumor material from sequential biopsies by the use of immunohistochemistry and image analysis including, but not limited to the four markers CD66b, CD3, CD8 and FOXp3 that visualizes granulocytes, T-lymphocytes, cytotoxic T-lymphocytes and Tregs respectively.

Gene expression analysis

Expression of specific genes in tumor will be analyzed with a next generation RNA expression technology, NanoString nCounter™ (NanoString Technologies, Seattle, WA) in FFPE tumor material. For this purpose, FFPE blocks with biopsy specimens will be shipped to the Department of Pathology, Odense University Hospital (Sönke Detlefsen). Here, sections of 8-10 µm thickness will be cut by a microtome and mounted on glass slides. Afterwards, paraffin will be removed using limonene treatment, and RNA will be extracted (Prosigna). To achieve the amounts of RNA needed for the RNA expression analysis, the number of slides cut will depend on the tumor area in the biopsy. If needed, macrodissection for reduction of the amount of non-tumor tissue will be performed. For most gene expression assays NanoString recommends 125-500 ng of total RNA (see **Table 5**). Because of the relatively high amounts of RNA needed for the analyses, we will try to obtain histological Tru-Cut™ biopsies (width 1.2 mm) wherever possible, as EUS-guided fine-needle biopsies only have a width of around 0.4 – 0.5 mm and will yield lover amounts of representative RNA.

Target area (mm^2)	Number of 10µm sections
4-19	6
20-99	3
≥100	1

Table 5: Laser microdissection and NanoString analyses.

Amount of needed tissue area to achieve sufficient RNA concentrations.

The NanoString nCounter™ method uses probes with 6-color bar codes to identify specific RNA sequencing (without gene amplification as is required with traditional RNA sequencing or qPCR technologies) and makes it possible to analyze up to 800 transcripts in sparse FFPE material. The PanCancer IO 360™ (NanoString Technologies, Seattle, WA) gene expression panel, a unique 770 gene expression panel, will be used. This panel covers factors as shown below (**Figure 3**):

TUMOR		MICROENVIRONMENT		IMMUNE RESPONSE	
Category/Gene Number		Category/Gene Number		Category/Gene Number	
Release of Cancer Cell Antigens	74	Angiogenesis	40	Cancer Antigen Presentation	95
Cell Cycling and Proliferation	54	Extracellular Matrix Remodelling	43	T cell priming and Activation	151
Tumor Intrinsic Factors	156	Collagens	6	Immune Cells Localization to Tumors	293
Common Signaling Pathways	172	Metastasis	20	Recognition of Cancer Cells by T cells	103
				Killing of Cancers Cells	177
				Myeloid Cell Activity	262
				NK Cell Activity	28
				Immunometabolism	99

Figure 3: NanoString PanCancer IO 360™ gene expression panel overview
Reprinted from http://www.nanostring.com[58]

Tumor DNA analysis

Mutational profiling of tumor DNA is carried out on the fresh-frozen tissue sample using an enrichment-based approach targeting 523 cancer-related genes, TruSight™ Oncology 500 High-Throughput kit (Illumina™). Some patients may have previously had tumor DNA whole genome sequencing (WGS) performed as part of the clinical practice in the institution. If this is the case, and the tissue samples for this analysis has been performed on the IRE ablated metastasis, the results from these clinical tests may be used instead of the gene panel. The genes included in the panel are presented in **Appendix E: Genes included in the TruSight™ Oncology 500 and TruSight™ Oncology High Throughput panel**.

The 1.94Mb panel is sequenced on a NovaSeq 6000 system (Illumina™).

Blood analyses

Cellular immunology

At baseline and at each subsequent visit two 9 ml (sodium heparinized) unfrozen samples are directly transferred to the Aalborg University laboratory of immunology for flowcytometric analysis. The cells are labeled using fluorochrome conjugated antibodies specific for multiple subpopulations of leucocytes.

We intend to monitor the status of and possible changes within the populations of naïve, EM, CM, EFF, TE, EX T cells, Tregs, cDC1, cDC2, pDC and MDSC in the blood of patients. The monocyte- and polymorphonuclear subsets of MDSC will be enumerated by a multicolor flow cytometry[66]. Furthermore, we intend to investigate whether there will be any changes in the surface expression of PD-L1 by some of these cells, as a high expression of PD-L1 is typical for DCs in the tolerogenic state. The granulocyte/lymphocyte ratio in the blood of the patients will be calculated based on standard complete blood cell counts.

Immunological analyses of blood samples will be performed in the Laboratory of Immunology, Medical Microbiology and Immunology, Department of Health Science and Technology, AAU. Ralf Agger and Emil Kofod-Olsen who will supervise these analyses have extensive experience within flow cytometry. All the necessary equipment is available.

Cytokines

The rest of the blood sample drawn is frozen for later analysis of cytokines, including but not limited to IL-2, -4, -6, -10, TNF, IFNγ, TGFβ and soluble PD-L1. The concentration of each variable will be compared to baseline values.

Cell-free tumor DNA analyses

It is hypothesized that the cell-free tumor DNA yield may increase in the immediate period after an electroporation ablation, as has been theorized in animal studies[59]. This may offer a more representative tumor profile compared to conventional biopsies, as the whole tumor "leaks" DNA because of the pore formation in electroporated tissue. If this hypothesis is true, the fraction of tumor specific cfDNA may be larger immediately after IRE in comparison to conventional liquid biopsies. Moreover, because of the spatial heterogeneity of mutations in tumors, the detectable mutations may be more representable of the whole tumor, compared to conventional tumor profiling using only DNA obtained from tissue samples, as these are limited by the size of the tumor biopsy. This gives some interesting aspects in the age of personalized medicine, as targeted therapies are only as good as the diagnostic genetic analysis being performed.

To assess the changes in cfDNA yield, quantitative polymerase chain reaction (qPCR) of a specific, tumor-associated, mutated gene will be performed and converted into cfDNA concentration. This analysis will be performed on plasma samples taken immediately before and after IRE. Subsequently, if this hypothesis is true, the cell-free tumor DNA content will be determined using the TruSight™ Oncology 500 (Illumina) for mutational profiling of the cfDNA, to examine whether the number of detectable mutations increase after IRE and whether the number of mutations are higher compared to the tissue samples taken from the IRE ablated metastasis.

The mutational profiling will elucidate the difference in cancer-related mutational diversity of the three "types" of biopsies (conventional liquid biopsies, tissue biopsies and electroporation-assisted liquid biopsies), by comparing sequencing data on tissue samples with sequencing performed on blood samples before and immediately after IRE.

The sequencing, data management and bioinformatics will be performed by the Department of Molecular Diagnostics, Aalborg University Hospital. In brief, DNA is isolated from each sample and sequenced. The raw data output of each sample is aligned for comparison with reference data. Somatic variants are identified, tumor mutational burden (TMB) and microsatellite instability calculated and compared across the three samples "types" from each patient.

Genetic analysis will be performed on samples stored in the biobank in bulk after all patients have been included in the trial.

Genetic analysis may lead to unrelated critical health findings. However, with a targeted approach and analysis focusing on somatic variants, the risk of incidental findings is slim. Most likely is detection of possible germline BRCA-mutations, which is present in approximately 5% of pancreatic cancers. More information on this is available in the section **Ethical considerations**.

Statistical methods

Descriptive statistics will be applied to the safety data. Survival analysis will be carried out in comparison to a matched cohort of previously treated patients. Propensity score matching will be used. Survival comparison will be conducted using the Kaplan-Meier estimator and tested for equality using the Log-rank test. Categorical outcomes from analysis of CT scans will be tested using Fishers exact test. PRO will be tested using mixed regression.

In general, continuous variables will be summarized with standard descriptive statistics including means, standard deviations, medians, and ranges. Categorical variables will be summarized with frequencies and percentages. Ninety-five percent confidence intervals will be provided for descriptive statistics. Markedly non-normally distributed data will be transformed prior to inferential comparisons. For variables that cannot be successfully transformed, nonparametric methods will be used. An overall alpha-level of 0.05 will be used as a cut-point for statistical significance and all statistical tests will be two-sided. All data will be analyzed using Stata v16 (StataCorp LLC, College Station, TX).

PERSPECTIVES

This trial is a pilot study primarily designed to estimate the feasibility, oncological effect and safety of this novel combined intervention strategy. If successful, the results will need to be validated in larger trials in order to make any conclusion regarding efficacy. However, preclinical trials are promising and thus warrant this clinical trial. If this treatment combination is efficacious in PC patients this would also warrant further investigations in other tumors. Immunotherapy has an alternative side effect profile than conventional chemotherapy and thus may be available to patients, that do not tolerate current chemotherapeutic treatments. Additionally, the study will provide valuable insight into the immunological changes happening in patients receiving IRE and CPIs. Finally, the study will provide insight into the quality of life and nutritional impact of both IRE ablations and CPIs, which is an area that is not well studied in pancreas cancer patients.

ETHICAL PRESENTATION

Patients entering the trial will be offered a novel treatment that is unproven in humane subjects. Second-line chemotherapy, on the other hand, has some (low-level) evidence of efficacy and is considered best practice. However, by participating, patients will be offered hope and ideally increased survival and quality of life, when compared to non-participants. Of special importance is the fact that CPI treatment offers a completely different toxicity profile when compared to conventional chemotherapy. Thus, this may be preferable for some patients wishing to participate in the study. After participation, patients who are sufficiently fit will be offered standard treatment. Thus, there will be an additional line of treatment for some patients.

Potential benefits, risks and side-effects by participation

By participating, patients are subjected to an increased risk of complications, as IRE is an invasive procedure that participants would not otherwise be subjected to. However, ablation of liver tumors with IRE has been examined in several clinical trials and only rarely leads to serious complications. The safety profile of pembrolizumab is well described and toxicity is most frequently mild and compares favorably with chemotherapy.

Serial liver biopsies are not standard in the treatment of PC patients. The most common symptoms after ultrasound-guided percutaneous biopsies are pain and irritation. Steps has been taken to minimize the number of biopsies as described above. However, biopsies may in rare cases cause intraabdominal bleeding or other serious adverse events. All biopsies will be taken in accordance with best practice guidelines, including proper treatment pause in anticoagulant treatment.

Participants may be subjected to more radiation than patients receiving the standard treatment. While the CT-scans during active treatment and the follow-up program (i.e. in treatment pause) is equivalent to patient receiving standard chemotherapeutic treatment, participant will be subject to between one and two extra CT scans (a safety scan 10 days after IRE and a baseline scan, if a high quality scan has not been performed within 1 month of inclusion). Based on the scan-protocols used this is an effective radiation dose of 17.6-35.2 mSv (dependent one whether a patient receives one or two extra scans). This dose corresponds to an increased risk of cancer death of 0.088% - 0.176% compared to the background population. However, for this patient group the risk of cancer death is ≈ 100%. Thus, there is no relevant increase in risk.

Ethical considerations

This protocol will be submitted for review by the North Denmark Region Committee on Health Research Ethics and to the Danish Medical Agency. Patients treated with IRE and CPIs will be submitted to a low risk of known complications as described above but may also be subjected to unknown complications resulting from the novel combination. We believe that this risk is acceptable, as most known complications are manageable. Any signs of serious illness will lead to further evaluation of the patient. The surgical department have prior experience in IRE treatment of LAPC as described above. All IRE treatments will be carried out by two experienced interventional radiologists with prior experience using IRE in PC. If laparotomy is necessary to safely perform the ablations, the treatment will be assisted by a board-certified HPB surgeon with IRE experience. Furthermore, as the institution is currently running another study

regarding IRE ablations of liver tumors, the radiologists and surgeons are currently planned (postponed due to COVID-19 pandemic) for surgical training from an international institution performing IRE ablations in liver tumors today. The involved radiologists and surgeons have previously carried out surgical training in live animals to perfect the technique and workflow. Treatment with pembrolizumab is routine practice in the oncological department, and the involved oncologists have experience in handling of adverse effects of immunotherapy and in conducting experimental clinical trials, including trials of immunotherapy and new combinations.

All the standard blood samples taken is part of routine care for patients receiving CPIs and/or liver ablations at this institution. Additional blood samples and biopsies taken for translational research purposes will be analyzed immediately or stored in a research biobank and analyzed in bulk (for more information, see section **Biobanks**). Blood samples for the biobank will be taken in conjunction with standard blood samples. Thus, the participants will not be subjected to more needle punctures than standard. Patients, who at any point during the follow-up period choose to withdraw consent, will not be subjected to any research-related blood sampling.

Patients entering the trial will be exposed to excess radiation. However, it is very unlikely to have any adverse effect on these patients as they already have an incurable malignant disease with a short life expectancy.

All inclusion criteria and none of the exclusion criteria must be met in order to enter the trial. Patients will be excluded from receiving further experimental treatment if they, during the trial, develop severe toxicity to the drug or severe complications to IRE precluding further immunotherapy. During the follow-up period there are no restriction on other treatments.

Samples of participants blood and tissue will be analyzed by targeted sequencing of 523 cancer related genes in the search for somatic variants. Hence, the risk of incidental findings is minor. Furthermore, the results of these analysis will likely not affect most of the participant themselves, because these analyses will be performed in bulk at the end of the trial, were the majority expectedly will be diseased. However, in the rare event that a germline genetic variant is identified incidentally, it will be discussed in an *ad hoc* multidisciplinary conference with representation of a Consultant in Clinical Genetics. If the results are deemed to have implications for living relatives of the participants and for the living participants, they will be offered genetic counseling. The patients will be explicitly informed about and asked to consent to the genetic analysis before entering the trial. Participant will have the option to opt out of receiving any information about the results of the analysis.

Study participants will not receive financial compensation or other benefits and will be treated according to the Helsinki declaration. Complications directly attributable to the treatment will be eligible for insurance claims from the Danish public patient insurance (“Patienterstatningen”).

PROJECT MANAGEMENT

Timeline and study feasibility

Based on the institutional throughput of PC patients, recruitment of 16 participants should take less than two years. Thus, the entire study can be concluded within 3 years including 6 months of active treatment and 6 months of follow-up for the last included patient. Translational studies will be finalized, when the clinical trial has concluded, and will have no impact on the treatment of patients in the trial. We currently have the expertise to perform the trial and all available equipment and support structures. Additionally, we have incorporated a workflow, which makes the feasibility of completing the trial highly likely.

To start the project, we need to get approval from the ethical committee and from the Danish Medical Agency. We aim to start recruiting patients on January 1st, 2021.

Data analysis plan

Data on safety will be monitored continuously throughout the trial. Final data on safety, survival, imaging response and PRO will be analyzed when the last included patient has been followed for at least 6 months after pembrolizumab treatment has concluded or discontinued. Finalization of translational studies will be achieved post-hoc.

Data management and security

Study data is collected and managed using the encrypted REDCap electronic data capture tools hosted at Region Nordjylland[60]. REDCap (Research Electronic Data Capture) is a secure, web-based application designed to support data capture for research studies, providing 1) an intuitive interface for validated data entry; 2) audit trails for tracking data manipulation and export procedures; 3) automated export procedures for seamless data downloads to common statistical packages; and 4) procedures for importing data from external sources.

The project will be registered in the North Denmark Region common registry for ongoing trials. When approved by the ethical board, the protocol will be made available on https://www.clinicaltrials.gov.

To ensure the safety of participant and to get the research data upon trial completion, access to electronic patient files will be needed throughout the trial. Access to electronic patient journal will be needed to get data on patient survival, adverse events, biochemistry and to do post-hoc expert interpretation of CT scans. Post-hoc data access to the time before inclusion will be needed to get information on date of diagnosis, prior chemo(radiation)therapy, concurrent medical conditions and histological examination. Patients will be asked to give explicit informed consent to data access for their electronic patient journal until the study period ends. Participant will be asked to give access to information to a 5-year period before and after inclusion. Information will be given about the intended use of the data as well as the intent to publish in international journals. Written information will contain information that researchers, sponsors, sponsor representatives and quality-control units will have direct access to the medical journal, and that data may be used to complete the research project, monitor adverse events and to do quality-control.

Patient data will be handled in accordance with “databeskyttelsesloven og -forordningen”, the Danish adaptation of the European Union General Data Protection Regulation (GDPR).

Biobanks

Two biobanks are created during the study. One containing the tissue samples and one containing the blood samples.

The tissue sample biobank will be situated at the Department of Pathology, Aalborg University Hospital. Tissue samples will be transferred in bulk to the Department of Pathology, Odense University Hospital for analysis. Any remaining material will be transferred back to the Department of Pathology, Aalborg University Hospital and kept for future research, if the patients has agreed to this in the informed consent form.

The blood sample biobank will be situated at the Department of Gastrointestinal Surgery. Any remaining blood in the biobank, after the study period has concluded, will be kept for future research if the patients has given consent to this.

If patients have not given consent to keep excess blood or tissue samples, the samples will be destroyed when the study period has concluded.

Good clinical practice

The project will adhere to the ICH good clinical practice (GCP) guidelines and monitored by the local GCP unit ("GCP enheden ved Aalborg og Aarhus Universitetshospital").

Project group and management structure

The main project group consists of four physicians from the Department of Gastrointestinal Surgery and the Department of Oncology at Aalborg University Hospital. Both departments have established research departments and a longstanding tradition of collaboration. The group consists of different competences which contribute to the project. Ole Thorlacius-Ussing is the clinical professor of surgery and has major experience in leading and coordinating clinical research. Ole will be serving as Sponsor in this project and thus will be responsible for the logistical management of the project as well as project oversight (including auditing and reporting of safety outcomes to the proper authorities). Morten Ladekarl is clinical professor in oncology and has done extensive research in PC throughout his career. Morten will be serving as Principal investigator and will be responsible for the clinical management of patients and the scientific goals of the project. Laurids Østergaard Poulsen holds a PhD and is a specialist in the field of oncology with clinical experience in trial conduct, treatment with CPIs and gastro-intestinal cancers. Rasmus Virenfeldt Flak is a PhD-student and surgical resident actively conducting research in IRE and PC. Laurids and Rasmus will serve as clinical study coordinators and investigators of the study.

Additionally, several experts have agreed to collaborate in the trial: Mogens Tornby Stender (Consultant surgeon, UEMS certified HPB surgeon) holds a PhD. Mogens will serve as the main surgical advisor. Ralf Agger and Emil Kofod-Olsen hold faculty positions at Aalborg university and are experts in cancer immunology. Both will plan, assist and supervise the flowcytometry-based immunological studies. Gintare Naujokaite and Olga Tcacenco are radiologist with specialty in abdominal imaging and interventional radiology. Both have prior experience in systematic response evaluation and percutaneous ablative procedures (including IRE). Both are also scheduled to receive specialized training in liver IRE at a major institution abroad. Sönke Detlefsen is the professor of pathology at Odense University Hospital. Sönke and

his team will analyze the biopsies using the Nanostring gene expression panel. The team has prior experience with this technique.

Publications and authorship

All results from the trial will be published in international peer-reviewed journals independently of study outcome. Authorship will be attributed according to the International committee of medical journal editors (ICMJE) guidelines[61].

Financial aspects and conflicts of interest

The cost of immunotherapy, salary for the project nurse and half of the cost of IRE treatments has been granted by the Danish Cancer Society (1,850,000 kr). Additional salaries and material costs is covered by the involved departments. None of the involved researchers has received any personal financial compensation or has other personal financial interest in the outcomes of the study. Additional funding will be sought to cover expenses related to translational studies.

REFERENCES

1. Siegel RL, Miller KD, Jemal A (2018). *Cancer Statistics, 2018*. *CA Cancer J Clin* 68: 7–30. https://doi.org/10.3322/caac.21442.
2. Konstantinidis IT, Warshaw AL, Allen JN, Blaszkowsky LS, Fernandez-Del Castillo C, Deshpande V, *et al* (2013). *Pancreatic Ductal Adenocarcinoma Is There a Survival Difference for R1 Resections Versus Locally Advanced Unresectable Tumors? What Is a 'True' R0 Resection? Ann Surg* 257: 731–5. https://doi.org/10.1097/SLA.0b013e318263da2f.
3. Stewart BW, Wild CP (2014). *IARC World Cancer Report 2014*. WHO Press. https://doi.org/10.1017/S0020860400079146.
4. Chan KKW, Guo H, Cheng S, Beca JM, Redmond-Misner R, Isaranuwatchai W, *et al* (2019). *Real-world Outcomes of FOLFIRINOX vs Gemcitabine and Nab-paclitaxel in Advanced Pancreatic Cancer: A Population-based Propensity Score-weighted Analysis*. *Cancer Med*: cam4.2705. https://doi.org/10.1002/cam4.2705.
5. Neoptolemos JP, Kleeff J, Michl P, Costello E, Greenhalf W, Palmer DH (2018). *Therapeutic Developments in Pancreatic Cancer: Current and Future Perspectives*. *Nat Rev Gastroenterol Hepatol* 15: 333–48. https://doi.org/10.1038/s41575-018-0005-x.
6. Rasmussen LS, Vittrup B, Ladekarl M, Pfeiffer P, Yilmaz MK, Poulsen LØ, *et al* (2018). *743PInitial Treatment and Survival in a National Unselected Danish Cohort of 4161 Patients with Pancreatic Cancer*. *Ann Oncol* 29. https://doi.org/10.1093/annonc/mdy282.126.
7. Conroy T, Desseigne F, Ychou M, Bouché O, Guimbaud R, Bécouarn Y, *et al* (2011). *FOLFIRINOX versus Gemcitabine for Metastatic Pancreatic Cancer*. *N Engl J Med* 364: 1817–25. https://doi.org/10.1056/NEJMoa1011923.
8. O'Neill C, Hayat T, Hamm J, Healey M, Zheng Q, Li Y, *et al* (2020). *A Phase 1b Trial of Concurrent Immunotherapy and Irreversible Electroporation in the Treatment of Locally Advanced Pancreatic Adenocarcinoma*. *Surg (United States)*: 1–7. https://doi.org/10.1016/j.surg.2020.04.057.
9. Luchini C, Bibeau F, Ligtenberg MJL, Singh N, Nottegar A, Bosse T, *et al* (2019). *ESMO Recommendations on Microsatellite Instability Testing for Immunotherapy in Cancer, and Its Relationship with PD-1/PD-L1 Expression and Tumour Mutational Burden: A Systematic Review-Based Approach*. *Ann Oncol Off J Eur Soc Med Oncol* 30: 1232–43. https://doi.org/10.1093/annonc/mdz116.
10. Humphris JL, Patch A-M, Nones K, Bailey PJ, Johns AL, McKay S, *et al* (2017). *Hypermutation In Pancreatic Cancer*. *Gastroenterology* 152: 68-74.e2. https://doi.org/10.1053/j.gastro.2016.09.060.
11. Johansson H, Andersson R, Bauden M, Hammes S, Holdenrieder S, Ansari D (2016). *Immune Checkpoint Therapy for Pancreatic Cancer*. *World J Gastroenterol* 22: 9457–76. https://doi.org/10.3748/wjg.v22.i43.9457.
12. Royal RE, Levy C, Turner K, Mathur A, Hughes M, Kammula US, *et al* (2010). *Phase 2 Trial of Single Agent Ipilimumab (Anti-CTLA-4) for Locally Advanced or Metastatic Pancreatic Adenocarcinoma*. *J Immunother* 33: 828–33. https://doi.org/10.1097/CJI.0b013e3181eec14c.
13. Le DT, Lutz E, Uram JN, Sugar EA, Onners B, Solt S, *et al* (2013). *Evaluation of Ipilimumab in Combination with Allogeneic Pancreatic Tumor Cells Transfected with a GM-CSF Gene in Previously Treated Pancreatic Cancer*. *J Immunother* 36: 382–9. https://doi.org/10.1097/CJI.0b013e31829fb7a2.
14. Aglietta M, Barone C, Sawyer MB, Moore MJ, Miller WH, Bagalà C, *et al* (2014). *A Phase I Dose Escalation Trial of Tremelimumab (CP-675,206) in Combination with Gemcitabine in Chemotherapy-Naive Patients with Metastatic Pancreatic Cancer*. *Ann Oncol* 25: 1750–5. https://doi.org/10.1093/annonc/mdu205.
15. Brahmer JR, Tykodi SS, Chow LQM, Hwu WJ, Topalian SL, Hwu P, *et al* (2012). *Safety and Activity of*

Anti-PD-L1 Antibody in Patients with Advanced Cancer. *N Engl J Med* 366: 2455–65. https://doi.org/10.1056/NEJMoa1200694.

16. Andersson R, Pereira C, Bauden M, Ansari D (2019). *Is Immunotherapy the Holy Grail for Pancreatic Cancer?*. 17–20.
17. Flak RV, Stender MT, Jensen TM, Andersen KL, Henriksen SD, Mortensen PB, *et al* (2019). *Treatment of Locally Advanced Pancreatic Cancer with Irreversible Electroporation – a Danish Single Center Study of Safety and Feasibility*. *Scand J Gastroenterol*: 1–7. https://doi.org/10.1080/00365521.2019.1575465.
18. Zhao J, Wen XX, Tian L, Li T, Xu C, Wen XX, *et al* (2019). *Irreversible Electroporation Reverses Resistance to Immune Checkpoint Blockade in Pancreatic Cancer*. *Nat Commun* 10: 1–14. https://doi.org/10.1038/s41467-019-08782-1.
19. Neal RE, Rossmeisl JH, Robertson JL, Arena CB, Davis EM, Singh RN, *et al* (2013). *Improved Local and Systemic Anti-Tumor Efficacy for Irreversible Electroporation in Immunocompetent versus Immunodeficient Mice*. *PLoS One* 8: 1–10. https://doi.org/10.1371/journal.pone.0064559.
20. Bulvik BE, Ahmed M, Andriyanov A V, Goldberg SN (2016). *Irreversible Electroporation versus Radiofrequency Ablation : A Comparison of Local and Systemic*. 280. https://doi.org/10.1148/radiol.2015151166.
21. Shao Q, O'Flanagan S, Lam T, Roy P, Pelaez F, Burbach BJ, *et al* (2019). *Engineering T Cell Response to Cancer Antigens by Choice of Focal Therapeutic Conditions*. *Int J Hyperth* 36: 130–8. https://doi.org/10.1080/02656736.2018.1539253.
22. Lin M, Alnaggar M, Liang S, Wang X, Liang Y, Zhang M, *et al* (2017). *An Important Discovery on Combination of Irreversible Electroporation and Allogeneic Natural Killer Cell Immunotherapy for Unresectable Pancreatic Cancer. Oncotarget* 8: 101795–807. https://doi.org/10.18632/oncotarget.21974.
23. Sutter O, Calvo J, N'Kontchou G, Nault J-C, Ourabia R, Nahon P, *et al* (2017). *Safety and Efficacy of Irreversible Electroporation for the Treatment of Hepatocellular Carcinoma Not Amenable to Thermal Ablation Techniques: A Retrospective Single-Center Case Series*. *Radiology* 284: 877–86. https://doi.org/10.1148/radiol.2017161413.
24. Bhutiani N, Philips P, Scoggins CR, McMasters KM, Potts MH, Martin RCG (2016). *Evaluation of Tolerability and Efficacy of Irreversible Electroporation (IRE) in Treatment of Child-Pugh B (7/8) Hepatocellular Carcinoma (HCC)*. *HPB* 18: 593–9. https://doi.org/10.1016/j.hpb.2016.03.609.
25. Verloh N, Jensch I, Lürken L, Haimerl M, Dollinger M, Renner P, *et al* (2019). *Similar Complication Rates for Irreversible Electroporation and Thermal Ablation in Patients with Hepatocellular Tumors*. https://doi.org/10.2478/raon-2019-0011.
26. Scheffer HJ, Nielsen K, van Tilborg AAJM, Vieveen JM, Bouwman RA, Kazemier G, *et al* (2014). *Ablation of Colorectal Liver Metastases by Irreversible Electroporation: Results of the COLDFIRE-I Ablate-and-Resect Study*. *Eur Radiol* 24: 2467–75. https://doi.org/10.1007/s00330-014-3259-x.
27. Hosein PJ, Echenique A, Loaiza-Bonilla A, Froud T, Barbery K, Rocha Lima CM, *et al* (2014). *Percutaneous Irreversible Electroporation for the Treatment of Colorectal Cancer Liver Metastases with a Proposal for a New Response Evaluation System*. *J Vasc Interv Radiol* 25: 1233-1239.e2. https://doi.org/10.1016/j.jvir.2014.04.007.
28. Langan RC, Goldman DA, D'Angelica MI, DeMatteo RP, Allen PJ, Balachandran VP, *et al* (2017). *Recurrence Patterns Following Irreversible Electroporation for Hepatic Malignancies*. *J Surg Oncol* 115: 704–10. https://doi.org/10.1002/jso.24570.
29. Frühling P, Nilsson A, Duraj F, Haglund U, Norén A (2017). *Single-Center Nonrandomized Clinical Trial to Assess the Safety and Efficacy of Irreversible Electroporation (IRE) Ablation of Liver Tumors in Humans: Short to Mid-Term Results*. *Eur J Surg Oncol* 43: 751–7.

https://doi.org/10.1016/j.ejso.2016.12.004.
30. Eisele RM, Chopra SS, Glanemann M, Gebauer B (2014). *Risk of Local Failure after Ultrasound Guided Irreversible Electroporation of Malignant Liver Tumors. Interv Med Appl Sci* 6: 147–53. https://doi.org/10.1556/IMAS.6.2014.4.2.
31. Stillström D, Beermann M, Engstrand J, Freedman J, Nilsson H (2019). *Initial Experience with Irreversible Electroporation of Liver Tumours*. *Eur J Radiol Open* 6: 62–7. https://doi.org/10.1016/j.ejro.2019.01.004.
32. Zeng J, Liu G, Li ZH, Yang Y, Fang G, Li RR, *et al* (2017). *The Safety and Efficacy of Irreversible Electroporation for Large Hepatocellular Carcinoma*. *Technol Cancer Res Treat* 16: 120–4. https://doi.org/10.1177/1533034616676445.
33. Distelmaier M, Barabasch A, Heil P, Kraemer NA, Isfort P, Keil S, *et al* (2017). *Midterm Safety and Efficacy of Irreversible Electroporation of Malignant Liver Tumors Located Close to Major Portal or Hepatic Veins*. *Radiology* 285: 1023–31. https://doi.org/10.1148/radiol.2017161561.
34. Beyer LP, Pregler B, Michalik K, Niessen C, Dollinger M, Müller M, *et al* (2017). *Evaluation of a Robotic System for Irreversible Electroporation (IRE) of Malignant Liver Tumors: Initial Results*. *Int J Comput Assist Radiol Surg* 12: 803–9. https://doi.org/10.1007/s11548-016-1485-1.
35. Dollinger M, Beyer LP, Haimerl M, Niessen C, Jung E-M, Zeman F, *et al* (2015). *Adverse Effects of Irreversible Electroporation of Malignant Liver Tumors under CT Fluoroscopic Guidance: A Single-Center Experience*. *Diagnostic Interv Radiol* 21: 471–5. https://doi.org/10.5152/dir.2015.14442.
36. Cannon R, Ellis S, Hayes D, Narayanan G, Martin RCG (2013). *Safety and Early Efficacy of Irreversible Electroporation for Hepatic Tumors in Proximity to Vital Structures*. *J Surg Oncol* 107: 544–9. https://doi.org/10.1002/jso.23280.
37. Eller A, Schmid A, Schmidt J, Uder M, Brand M, Saake M, *et al* (2014). *Local Control of Perivascular Malignant Liver Lesions Using Percutaneous Irreversible Electroporation: Initial Experiences*. *Cardiovasc Intervent Radiol* 38: 152–9. https://doi.org/10.1007/s00270-014-0898-x.
38. Giorgio A, Amendola F, Calvanese A, Ingenito E, Santoro B, Gatti P, *et al* (2019). *Ultrasound-Guided Percutaneous Irreversible Electroporation of Hepatic and Abdominal Tumors Not Eligible for Surgery or Thermal Ablation: A Western Report on Safety and Efficacy*. *J Ultrasound* 22: 53–8. https://doi.org/10.1007/s40477-019-00372-7.
39. Cheung W, Kavnoudias H, Roberts S, Szkandera B, Kemp W, Thomson KR (2013). *Irreversible Electroporation for Unresectable Hepatocellular Carcinoma: Initial Experience and Review of Safety and Outcomes*. 12: 233–41. https://doi.org/10.7785/tcrt.2012.500317.
40. Sugimoto K, Moriyasu F, Kobayashi Y, Saito K, Takeuchi H, Ogawa S, *et al* (2015). *Irreversible Electroporation for Nonthermal Tumor Ablation in Patients with Hepatocellular Carcinoma: Initial Clinical Experience in Japan*. *Jpn J Radiol* 33: 424–32. https://doi.org/10.1007/s11604-015-0442-1.
41. Beyer LP, Pregler B, Nießen C, Schicho A, Haimerl M, Jung EM, *et al* (2016). *Stereotactically-Navigated Percutaneous Irreversible Electroporation (IRE) Compared to Conventional IRE: A Prospective Trial*. *PeerJ* 4: e2277. https://doi.org/10.7717/peerj.2277.
42. Duffy AG, Ulahannan S V, Makorova-Rusher O, Rahma O, Wedemeyer H, Pratt D, *et al* (2017). *Tremelimumab in Combination with Ablation in Patients with Advanced Hepatocellular Carcinoma*. *J Hepatol* 66: 545–51. https://doi.org/10.1016/j.jhep.2016.10.029.
43. Scheffer HJ, Stam AGM, Geboers B, Vroomen LGPH, Ruarus A, de Bruijn B, *et al* (2019). *Irreversible Electroporation of Locally Advanced Pancreatic Cancer Transiently Alleviates Immune Suppression and Creates a Window for Antitumor T Cell Activation*. *Oncoimmunology* 8. https://doi.org/10.1080/2162402X.2019.1652532.
44. Beitel-White N, Martin RCG, Li Y, Brock RM, Allen IC, Davalos R V (2019). *Real-Time Prediction of Patient Immune Cell Modulation during Irreversible Electroporation Therapy*. *Sci Rep* 9: 17739.

https://doi.org/10.1038/s41598-019-53974-w.
45. Carus A (2013). *Chronic Inflammation and Cancer*. Aarhus University.
46. Ino Y, Yamazaki-Itoh R, Shimada K, Iwasaki M, Kosuge T, Kanai Y, *et al* (2013). *Immune Cell Infiltration as an Indicator of the Immune Microenvironment of Pancreatic Cancer*. *Br J Cancer* 108: 914–23. https://doi.org/10.1038/bjc.2013.32.
47. De Monte L, Reni M, Tassi E, Clavenna D, Papa I, Recalde H, *et al* (2011). *Intratumor T Helper Type 2 Cell Infiltrate Correlates with Cancer-Associated Fibroblast Thymic Stromal Lymphopoietin Production and Reduced Survival in Pancreatic Cancer*. *J Exp Med* 208: 469–78. https://doi.org/10.1084/jem.20101876.
48. Fukunaga A, Miyamoto M, Cho Y, Murakami S, Kawarada Y, Oshikiri T, *et al* (2004). *CD8 + Tumor-Infiltrating Lymphocytes Together with CD4 +*. *Abacus A J Account Financ Bus Stud* 28: 26–31.
49. Kurahara H, Shinchi H, Mataki Y, Maemura K, Noma H, Kubo F, *et al* (2011). *Significance of M2-Polarized Tumor-Associated Macrophage in Pancreatic Cancer*. *J Surg Res* 167: e211–9. https://doi.org/10.1016/j.jss.2009.05.026.
50. Garcea G, Ladwa N, Neal CP, Metcalfe MS, Dennison AR, Berry DP (2011). *Preoperative Neutrophil-to-Lymphocyte Ratio (NLR) Is Associated with Reduced Disease-Free Survival Following Curative Resection of Pancreatic Adenocarcinoma*. *World J Surg* 35: 868–72. https://doi.org/10.1007/s00268-011-0984-z.
51. Bhatti I, Peacock O, Lloyd G, Larvin M, Hall RI (2010). *Preoperative Hematologic Markers as Independent Predictors of Prognosis in Resected Pancreatic Ductal Adenocarcinoma: Neutrophil-Lymphocyte versus Platelet-Lymphocyte Ratio*. *Am J Surg* 200: 197–203. https://doi.org/10.1016/j.amjsurg.2009.08.041.
52. An X, Ding PR, Li YH, Wang FH, Shi YX, Wang ZQ, *et al* (2010). *Elevated Neutrophil to Lymphocyte Ratio Predicts Survival in Advanced Pancreatic Cancer*. *Biomarkers* 15: 516–22. https://doi.org/10.3109/1354750X.2010.491557.
53. Ong SL, Garcea G, Thomasset SC, Mann CD, Neal CP, Abu Amara M, *et al* (2008). *Surrogate Markers of Resectability in Patients Undergoing Exploration of Potentially Resectable Pancreatic Adenocarcinoma*. *J Gastrointest Surg* 12: 1068–73. https://doi.org/10.1007/s11605-007-0422-6.
54. Hua J, Shi S, Liang D, Liang C, Meng Q, Zhang B, *et al* (2018). *Current Status and Dilemma of Second-Line Treatment in Advanced Pancreatic Cancer: Is There a Silver Lining?*. *Onco Targets Ther* 11: 4591–608. https://doi.org/10.2147/OTT.S166405.
55. Martin RCG, Durham AN, Besselink MG, Iannitti D, Weiss MJ, Wolfgang CL, *et al* (2016). *Irreversible Electroporation in Locally Advanced Pancreatic Cancer: A Call for Standardization of Energy Delivery*. *J Surg Oncol* 114: 865–71. https://doi.org/10.1002/jso.24404.
56. Eisenhauer EA, Therasse P, Bogaerts J, Schwartz LH, Sargent D, Ford R, *et al* (2009). *New Response Evaluation Criteria in Solid Tumours: Revised RECIST Guideline (Version 1.1)*. *Eur J Cancer* 45: 228–47. https://doi.org/10.1016/j.ejca.2008.10.026.
57. Common Terminology Criteria for Adverse Events (CTCAE). Available at: https://ctep.cancer.gov/protocolDevelopment/electronic_applications/ctc.htm [Accessed June 10, 2020].
58. PanCancer IO 360 Gene Expression Panel | NanoString Technologies. Available at: https://www.nanostring.com/products/gene-expression-panels/gene-expression-panels-overview/360-series-panel-collection/pancancer-io360-gene-expression-panel [Accessed August 6, 2020].
59. Golberg A, Sheviryov J, Solomon O, Anavy L, Yakhini Z (2019). *Molecular Harvesting with Electroporation for Tissue Profiling*. *Sci Rep* 9: 1–13. https://doi.org/10.1038/s41598-019-51634-7.
60. Harris PA, Taylor R, Thielke R, Payne J, Gonzalez N, Conde JG (2009). *Research Electronic Data*

Capture (REDCap)-A Metadata-Driven Methodology and Workflow Process for Providing Translational Research Informatics Support. *J Biomed Inform* 42: 377–81. https://doi.org/10.1016/j.jbi.2008.08.010.

61. International committee of medical journal editors. *ICMJE Recommendations*. Available at: http://www.icmje.org/recommendations/browse/roles-and-responsibilities/defining-the-role-of-authors-and-contributors.html [Accessed December 21, 2018].

APPENDIX A: PRECLINICAL STUDIES

New preclinical research has shown that IRE elicits a response to CPIs. In a study by Zhao et al. the combination of anti-PD1 and IRE was tested in a murine orthotopic PC model[18]. The result was that antiPD1 alone did not significantly impact the overall survival (8 vs 6 days). The same result was found for IRE alone (11.5 vs 6 days). However, when the treatments were applied in combination the survival was significantly prolonged (mOS 31.5 days, p<.0001), and 4 (36%) mice survived until the end of the trial at day 60 and where free of palpable tumors. The results where retested in additionally 7 mice, of which 3 (43%) survived for 60 days without palpable tumors. The surviving mice from both experiments where rechallenged with inoculation of new tumor cells, but all rejected the inoculation, thus showing long-term T-cell memory. The researchers did not find a significantly longer survival in mice receiving both antiPD1 and antiCTLA4 compared to antiPD1 alone. Furthermore, the experiment compared IRE + antiPD1 with radiation therapy (10Gy) + antiPD1. In this experiment the mOS was similar, however, all the radiation treated mice had progression and where dead by day 55. In contrast 4 of 11 mice in the IRE group survived for 120 days.

Neal et al. performed IRE ablations in immunocompetent and immunodeficient mice models of renal carcinoma[19]. Immunocompetent mice displayed longer survival compared to immunodeficient mice and sham controls from both mouse strains. Tissue samples subjected to CD3+ staining showed an increased T-cell presence in tumors after IRE in the immunocompetent group when compared to pretreatment samples. However, this was not the case in all animals. Additionally, rechallenged immunocompetent mice showed a significant reduction of tumor growth or complete rejection of injected tumor cells. Thus, the results suggest that IRE evokes adaptive immune system memory.

An additional animal experimental study by Bulvik et al. examined and compared the immunogenicity of IRE and radiofrequency ablation (RFA) in two mouse models of hepatocellular carcinoma[20]. In both cases macrophage infiltration was highest between day 7 and 14. RFA ablated tumors showed a significantly higher macrophage accumulation compared to IRE ablated tumors. RFA ablation was associated with a marked decrease in vessel patency, however this was not affected after IRE ablation, which showed ample penetration during bead injection studies. This indicates that therapeutic drugs would easily penetrate the tumors after IRE treatment. Among other proteins and cytokines, a marked increase in IL-6 secretion was measured on the day of treatment in both groups. IRE-treated mice showed IL-6 levels 10 times higher than untreated mice. In the RFA group levels were 3 times higher than in untreated mice. However, for both groups, IL-6 levels returned to normal one day after treatment. Furthermore, the authors showed that IRE in comparison with RFA increased the abscopal effect. However, IRE also displayed an increased tumorigenic effect on a cirrhotic mouse model. The authors conclude: *"Accordingly, our results may suggest that the choice of ablation device may have substantial effects on patient outcomes in some cases on the basis of as of yet incompletely elucidated pathways. Clearly, some tumors will likely have the potential for increased tumorigenesis, rendering RF ablation a better choice, whereas others are likely to be more immunogenic (abscopic), suggesting that even better outcomes could be achieved by using IRE.".*

Shao et al. examined the protein release and immune response after different focal therapies (heat, cryo and IRE) in vitro in B16 melanoma cells[21]. The study found that protein release was highest in the IRE treated cells. However, IRE yielded a lower concentration of native (not denaturated) protein when compared to cryotherapy. Both modalities vastly outperformed heat treatment. Subsequently, lysates where assessed in a T-cell assay to estimate the T-cell proliferation. In this sub experiment, IRE resulted in

higher T-cell proliferation compared to the other modalities. Cryotherapy only modestly outperformed heat treatment regarding T-cell proliferation.

APPENDIX B: PRODUCT RESUMÉ (DANISH)

BILAG I

PRODUKTRESUMÉ

1. LÆGEMIDLETS NAVN

KEYTRUDA 50 mg pulver til koncentrat til infusionsvæske, opløsning.

2. KVALITATIV OG KVANTITATIV SAMMENSÆTNING

Et hætteglas med pulver indeholder 50 mg pembrolizumab.

Efter rekonstitution indeholder 1 ml koncentrat 25 mg pembrolizumab.

Pembrolizumab er et humaniseret monoklonalt anti-*programmed cell death*-1 (PD-1)-antistof (IgG4/kappa-isotype med en stabiliserende forandring af sekvensen i Fc-regionen), som er fremstillet ved rekombinant dna-teknologi i ovarieceller fra kinesiske hamstre.

Alle hjælpestoffer er anført under pkt. 6.1.

3. LÆGEMIDDELFORM

Pulver til koncentrat til infusionsvæske, opløsning.

Hvidt til offwhite frysetørret pulver.

4. KLINISKE OPLYSNINGER

4.1 Terapeutiske indikationer

Melanom
KEYTRUDA som monoterapi er indiceret til behandling af fremskredent (inoperabelt eller metastatisk) melanom hos voksne.

KEYTRUDA som monoterapi er indiceret til adjuverende behandling af voksne med stadie III-melanom og lymfeknudeinvolvering, som har fået foretaget komplet resektion (se pkt. 5.1).

Ikke-småcellet lungecancer (NSCLC)
KEYTRUDA som monoterapi er indiceret til førstelinjebehandling af metastatisk ikke-småcellet lungecancer hos voksne, hvis tumorer udtrykker PD-L1 med *tumour proportion score* (TPS) ≥ 50% uden EGFR- eller ALK-positive mutationer i tumor.

KEYTRUDA, i kombination med pemetrexed og platinbaseret kemoterapi, er indiceret til førstelinjebehandling af metastatisk ikke-planocellulær ikke-småcellet lungecancer hos voksne uden EGFR- eller ALK-positive mutationer i tumorer.

KEYTRUDA, i kombination med carboplatin og enten paclitaxel eller nab-paclitaxel, er indiceret til førstelinjebehandling af metastatisk planocellulær ikke-småcellet lungecancer hos voksne.

KEYTRUDA som monoterapi er indiceret til behandling af lokalt fremskredent eller metastatisk ikke-småcellet lungecancer hos voksne efter tidligere behandling med minimum én kemoterapi, og hvis tumorer udtrykker PD-L1 med TPS ≥ 1%. Patienter med EGFR- eller ALK-positive mutationer i tumor bør også have været i targeteret behandling inden behandling med KEYTRUDA.

Klassisk Hodgkins lymfom (cHL)
KEYTRUDA som monoterapi er indiceret til behandling af recidiverende eller refraktært klassisk Hodgkins lymfom hos voksne, som har oplevet svigt af autolog stamcelletransplantation (ASCT) og svigt

af behandling med brentuximab vedotin (BV), eller som er uegnede til transplantation og har oplevet svigt af behandling med BV.

Urotelialt karcinom
KEYTRUDA som monoterapi er indiceret til behandling af lokalt fremskredent eller metastatisk urotelialt karcinom hos voksne, som tidligere har fået platinbaseret kemoterapi (se pkt. 5.1).

KEYTRUDA som monoterapi er indiceret til behandling af lokalt fremskredent eller metastatisk urotelialt karcinom hos voksne, som er uegnede til cisplatinbaseret kemoterapi, og hvis tumorer udtrykker PD-L1 med en kombineret positiv score (CPS) ≥ 10 (se pkt. 5.1).

Planocellulært hoved-hals karcinom (HNSCC)
KEYTRUDA som monoterapi eller i kombination med platinbaseret kemoterapi og 5-fluorouracil (5-FU) er indiceret til førstelinjebehandling af metastatisk eller inoperabelt recidiverende planocellulært hoved-hals karcinom hos voksne, hvis tumorer udtrykker PD-L1 med CPS ≥ 1 (se pkt. 5.1).

KEYTRUDA som monoterapi er indiceret til behandling af recidiverende eller metastatisk planocellulært hoved-hals karcinom hos voksne, hvis tumorer udtrykker PD-L1 med TPS ≥ 50% og med sygdomsprogression under eller efter platinbaseret kemoterapi (se pkt. 5.1).

Renalcellekarcinom (RCC)
KEYTRUDA, i kombination med axitinib, er indiceret til førstelinjebehandling af fremskredent renalcellekarcinom hos voksne (se pkt. 5.1).

4.2 Dosering og administration

Behandlingen skal initieres og superviseres af læger med erfaring i behandling af cancer.

PD-L1-test af patienter med NSCLC, urotelialt karcinom eller HNSCC
Ved behandling med KEYTRUDA som monoterapi anbefales det at teste for PD-L1-tumorekspression ved hjælp af en valideret test til at udvælge patienter med NSCLC eller tidligere ubehandlet urotelialt karcinom (se pkt. 4.1, 4.4, 4.8 og 5.1).

Patienter med HNSCC skal udvælges til behandling med KEYTRUDA som monoterapi eller i kombination med platinbaseret kemoterapi og 5-fluorouracil (5-FU) på basis af PD-L1-tumorekspression, som er bekræftet af en valideret test (se pkt. 4.1, 4.4, 4.8 og 5.1).

Dosering
Den anbefalede dosis af KEYTRUDA som monoterapi er enten 200 mg hver 3. uge eller 400 mg hver 6. uge administreret som intravenøs infusion over 30 minutter.

Den anbefalede dosis af KEYTRUDA som en del af kombinationsbehandling er 200 mg hver 3. uge administreret som intravenøs infusion over 30 minutter.

Patienterne skal behandles med KEYTRUDA indtil sygdomsprogression eller uacceptabel toksicitet. Atypisk respons (pseudoprogression) er observeret (initial forbigående stigning i tumorstørrelse eller små nye læsioner inden for de første få måneder efterfulgt af tumorregression). Det anbefales at fortsætte behandlingen hos klinisk stabile patienter med initiale tegn på sygdomsprogression, indtil sygdomsprogression er bekræftet.

Ved adjuverende behandling af melanom skal KEYTRUDA administreres indtil sygdomsrecidiv, uacceptabel toksicitet eller i en periode på op til 1 år.

Pausering eller seponering af behandling (se også pkt. 4.4)
Dosisreduktion af KEYTRUDA anbefales ikke. Behandlingen med KEYTRUDA skal pauseres eller seponeres for at håndtere bivirkninger som beskrevet i tabel 1.

Tabel 1: Anbefalede ændringer i KEYTRUDA-behandling

Immunrelaterede bivirkninger	Sværhedsgrad	Ændring af behandling
Pneumonitis	Grad 2	Pauser behandlingen indtil bivirkningerne bedres til grad 0-1*
	Grad 3 eller 4 eller tilbagevendende grad 2	Seponer behandlingen permanent
Colitis	Grad 2 eller 3	Pauser behandlingen indtil bivirkningerne bedres til grad 0-1*
	Grad 4 eller tilbagevendende grad 3	Seponer behandlingen permanent
Nefritis	Grad 2 med kreatinin > 1,5 til ≤ 3 gange øvre normale grænseværdi (ULN)	Pauser behandlingen indtil bivirkningerne bedres til grad 0-1*
	Grad ≥ 3 med kreatinin > 3 gange ULN	Seponer behandlingen permanent
Endokrinopatier	Grad 2 binyrebarkinsufficiens og hypofysitis	Pauser behandlingen indtil bivirkningen er kontrolleret med hormonsubstitutionsbehandling
	Grad 3 eller 4 binyrebarkinsufficiens eller symptomatisk hypofysitis<br><br>Type 1-diabetes associeret med hyperglykæmi grad ≥ 3 (glucose > 250 mg/dl eller > 13,9 mmol/l) eller associeret med ketoacidose<br><br>Hypertyroidisme grad ≥ 3	Pauser behandlingen indtil bivirkningerne bedres til grad 0-1*<br><br>Det kan overvejes at fortsætte behandlingen med pembrolizumab efter langsom udtrapning af eventuel nødvendig kortikosteroidbehandling hos patienter med endokrinopatier grad 3 eller grad 4, som bedres til grad 2 eller lavere med hormonsubstitutionsbehandling, hvis det er indiceret. Ellers skal behandlingen seponeres.
	Hypotyroidisme	Hypotyroidisme kan behandles med substitution uden afbrydelse af behandlingen.

4

Hepatitis<br><br>**BEMÆRK:** se doseringsvejledningen i hht. denne tabel for RCC-patienter i behandling med **pembrolizumab i kombination med axitinib** og med forhøjede leverenzymer.	Grad 2 med aspartataminotransferase (ASAT) eller alaninaminotransferase (ALAT) > 3-5 gange ULN eller total-bilirubin > 1,5-3 gange ULN	Pauser behandlingen indtil bivirkningerne bedres til grad 0-1*
	Grad ≥ 3 med ASAT eller ALAT > 5 gange ULN eller total-bilirubin > 3 gange ULN	Seponer behandlingen permanent
	Levermetastaser med grad 2 forhøjelse af ASAT eller ALAT, hepatitis med ASAT- eller ALAT-stigning på ≥ 50% i forhold til *baseline*, og som varer ≥ 1 uge	Seponer behandlingen permanent
Hudreaktioner	Grad 3 eller mistanke om Stevens-Johnsons syndrom (SJS) eller toksisk epidermal nekrolyse (TEN)	Pauser behandlingen indtil bivirkningerne bedres til grad 0-1*
	Grad 4 eller bekræftet SJS eller TEN	Seponer behandlingen permanent
**Andre immunrelaterede bivirkninger**	Baseret på sværhedsgrad og type af bivirkning (grad 2 eller grad 3)<br><br>Grad 3 eller 4 myokarditis<br>Grad 3 eller 4 encephalitis<br>Grad 3 eller 4 Guillain-Barrés syndrom<br><br>Grad 4 eller tilbagevendende grad 3	Pauser behandlingen indtil bivirkningerne bedres til grad 0-1*<br><br>Seponer behandlingen permanent<br><br>Seponer behandlingen permanent
**Infusionsreaktioner**	Grad 3 eller 4	Seponer behandlingen permanent

Bemærk: Toksicitetsgrader er angivet i overensstemmelse med *National Cancer Institute Common Terminology Criteria for Adverse Events Version 4.0* (NCI-CTCAE v.4).

* Hvis behandlingsrelateret toksicitet ikke bedres til grad 0-1 inden for 12 uger efter den sidste dosis KEYTRUDA, eller hvis kortikosteroiddoseringen ikke kan reduceres til ≤ 10 mg prednison eller tilsvarende pr. dag inden for 12 uger, skal KEYTRUDA seponeres permanent.

Sikkerheden ved re-initiering af behandling med pembrolizumab hos patienter, der tidligere har oplevet immunrelateret myokarditis, er ikke kendt.

KEYTRUDA, som monoterapi eller som kombinationsbehandling, skal seponeres permanent ved grad 4 eller tilbagevendende grad 3 immunrelaterede bivirkninger, medmindre det er specificeret anderledes i tabel 1.

Ved grad 4 hæmatologisk toksicitet, kun hos patienter med cHL, skal KEYTRUDA pauseres, indtil bivirkningerne bedres til grad 0-1.

KEYTRUDA i kombination med axitinib ved RCC
Se produktresuméet for axitinib for oplysninger vedrørende dosering af axitinib for RCC-patienter, der behandles med KEYTRUDA i kombination med axitinib. Når axitinib anvendes i kombination med pembrolizumab kan øgning af axitinib-dosis til over initialdosis på 5 mg overvejes i intervaller på seks uger eller længere (se pkt. 5.1).

Ved forhøjede leverenzymer hos RCC-patienter, der behandles med KEYTRUDA i kombination med axitinib:

- Hvis ALAT eller ASAT er ≥ 3 gange ULN, men < 10 gange ULN uden samtidig total-bilirubin-værdi ≥ 2 gange ULN, skal både KEYTRUDA og axitinib pauseres, indtil disse bivirkninger bedres til grad 0-1. Behandling med kortikosteroider kan overvejes. Genoptagelse af behandling (*rechallenge*) med et enkelt lægemiddel eller sekventiel genoptagelse af behandlingen (*sequential rechallenge*) med begge lægemidler efter bedring kan overvejes. Hvis behandlingen med axitinib genoptages, kan dosisreduktion i henhold til produktresuméet for axitinib overvejes.
- Hvis ALAT eller ASAT er ≥ 10 gange ULN eller > 3 gange ULN med samtidig total-bilirubin-værdi ≥ 2 gange ULN, skal både KEYTRUDA og axitinib seponeres permanent, og behandling med kortikosteroider kan overvejes.

Patienter, som bliver behandlet med KEYTRUDA, skal have udleveret et patientinformationskort og informeres om risiciene ved KEYTRUDA (se også indlægssedlen).

Særlige populationer
Ældre
Dosisjustering er ikke nødvendig hos patienter ≥ 65 år (se pkt. 5.1). Data fra patienter ≥ 65 år med cHL er for begrænsede til at kunne drage konklusioner (se pkt. 5.1). Data fra pembrolizumab som monoterapi hos patienter med resekteret stadie III-melanom, fra pembrolizumab i kombination med axitinib hos patienter med fremskredent RCC, fra kombinationsbehandling med kemoterapi hos patienter med metastatisk NSCLC, og fra pembrolizumab (med eller uden kemoterapi) hos patienter i førstelinjebehandling for metastatisk eller inoperabelt recidiverende HNSCC ≥ 75 år er begrænsede (se pkt. 4.4 og 5.1).

Nedsat nyrefunktion
Dosisjustering er ikke nødvendig hos patienter med let eller moderat nedsat nyrefunktion. KEYTRUDA er ikke undersøgt hos patienter med svært nedsat nyrefunktion (se pkt. 4.4 og 5.2).

Nedsat leverfunktion
Dosisjustering er ikke nødvendig hos patienter med let nedsat leverfunktion. KEYTRUDA er ikke undersøgt hos patienter med moderat eller svært nedsat leverfunktion (se pkt. 4.4 og 5.2).

Pædiatrisk population
KEYTRUDAs sikkerhed og virkning hos børn under 18 år er endnu ikke klarlagt. De foreliggende data er beskrevet i pkt. 4.8, 5.1 og 5.2.

Administration
KEYTRUDA er til intravenøs anvendelse. Det skal administreres som en infusion over 30 minutter. KEYTRUDA må ikke administreres som intravenøs push- eller bolus-injektion.

Se produktresuméet for de samtidigt administrerede lægemidler ved anvendelse i kombinationsbehandling. Når KEYTRUDA administreres som del af en kombinationsbehandling med intravenøs kemoterapi, skal KEYTRUDA administreres først.

For instruktioner om rekonstitution og fortynding af lægemidlet før administration, se pkt. 6.6.

4.3 Kontraindikationer

Overfølsomhed over for det aktive stof eller over for et eller flere af hjælpestofferne anført i pkt. 6.1.

4.4 Særlige advarsler og forsigtighedsregler vedrørende brugen

For at forbedre sporbarheden af biologiske lægemidler skal det administrerede produkts navn og batchnummer tydeligt registreres.

Vurdering af PD-L1-status
Ved vurdering af PD-L1-status af tumoren er det vigtigt, at der vælges en valideret og robust metode for at minimere forekomsten af falsk negative eller falsk positive resultater.

Immunrelaterede bivirkninger
Immunrelaterede bivirkninger, inklusive svære og dødelige tilfælde, er forekommet hos patienter, der fik pembrolizumab. De fleste immunrelaterede bivirkninger, der forekom under behandlingen med pembrolizumab, var reversible og blev håndteret ved hjælp af afbrydelse af behandlingen med pembrolizumab, behandling med kortikosteroider og/eller understøttende pleje. Immunrelaterede bivirkninger er også observeret efter den sidste dosis af pembrolizumab. Immunrelaterede bivirkninger, der påvirker mere end et organsystem i kroppen, kan forekomme på samme tid.

Ved mistanke om immunrelaterede bivirkninger skal det sikres, at der foretages tilstrækkelig udredning for at bekræfte ætiologi eller udelukke andre årsager. Baseret på bivirkningens sværhedsgrad skal behandlingen med pembrolizumab pauseres og kortikosteroider administreres. Ved bedring til grad ≤ 1 påbegyndes langsom udtrapning af kortikosteroider over en periode på mindst 1 måned. På basis af begrænsede data fra kliniske studier med patienter, hvis immunrelaterede bivirkninger ikke kunne kontrolleres med kortikosteroider, kan det overvejes at administrere andre systemiske immunsuppressiva.

Behandlingen med pembrolizumab kan genoptages inden for 12 uger efter den sidste dosis af KEYTRUDA, hvis bivirkningen bedres til grad ≤ 1, og kortikosteroiddosis er reduceret til ≤ 10 mg prednison dagligt eller en dosis ækvivalent hermed.

Pembrolizumab skal seponeres permanent ved en hvilken som helst immunrelateret bivirkning af grad 3, som recidiverer, og ved alle immunrelaterede bivirkninger af toksicitetsgrad 4, bortset fra endokrinopatier, som kontrolleres med hormonsubstitutionsbehandling (se pkt. 4.2 og 4.8).

Immunrelateret pneumonitis
Pneumonitis er rapporteret hos patienter, der fik pembrolizumab (se pkt. 4.8). Patienterne skal monitoreres for tegn og symptomer på pneumonitis. Mistanke om pneumonitis skal bekræftes ved en billeddiagnostisk udredning af patienterne, og andre årsager skal udelukkes. Kortikosteroider skal administreres ved bivirkninger grad ≥ 2 (initialdosis 1-2 mg/kg/dag prednison eller ækvivalent hermed efterfulgt af langsom udtrapning); pembrolizumab skal pauseres ved grad 2-pneumonitis og seponeres permanent ved grad 3-, grad 4- eller tilbagevendende grad 2-pneumonitis (se pkt. 4.2).

Immunrelateret colitis
Colitis er rapporteret hos patienter, der fik pembrolizumab (se pkt. 4.8). Patienterne skal monitoreres for tegn og symptomer på colitis, og andre årsager skal udelukkes. Kortikosteroider skal administreres ved bivirkninger grad ≥ 2 (initialdosis 1-2 mg/kg/dag prednison eller ækvivalent hermed efterfulgt af langsom udtrapning); pembrolizumab skal pauseres ved grad 2- eller grad 3-colitis og seponeres permanent ved grad 4- eller tilbagevendende grad 3-colitis (se pkt. 4.2). Den potentielle risiko for gastrointestinal perforation skal tages i betragning.

Immunrelateret hepatitis
Hepatitis er rapporteret hos patienter, der fik pembrolizumab (se pkt. 4.8). Patienterne skal monitoreres for ændringer i leverfunktionen (ved behandlingsstart, periodevist i løbet af behandlingen og som indiceret på basis af den kliniske vurdering) og for symptomer på hepatitis, og andre årsager skal udelukkes. Kortikosteroider skal administreres (initialdosis 0,5-1 mg/kg/dag [ved bivirkninger af grad 2] eller 1-2 mg/kg/dag [ved bivirkninger grad ≥ 3] prednison eller ækvivalent hermed efterfulgt af langsom udtrapning), og på basis af sværhedsgraden af leverenzymstigningen skal behandlingen med pembrolizumab pauseres eller seponeres (se pkt. 4.2).

Immunrelateret nefritis
Nefritis er rapporteret hos patienter, der fik pembrolizumab (se pkt. 4.8). Patienterne skal monitoreres for ændringer i nyrefunktionen, og andre årsager til nedsat nyrefunktion skal udelukkes. Kortikosteroider skal administreres ved bivirkninger grad ≥ 2 (initialdosis 1-2 mg/kg/dag prednison eller ækvivalent hermed efterfulgt af langsom udtrapning), og på basis af sværhedsgraden af kreatininstigningen skal behandlingen med pembrolizumab pauseres ved grad 2-nefritis og seponeres permanent ved grad 3- eller 4-nefritis (se pkt. 4.2).

Immunrelaterede endokrinopatier
Svære endokrinopatier, herunder binyrebarkinsufficiens, hypofysitis, type 1-diabetes mellitus, diabetisk ketoacidose, hypotyroidisme og hypertyroidisme er observeret i forbindelse med pembrolizumab-behandling.

Langtidsbehandling med hormonsubstitution kan være nødvendig ved immunrelaterede endokrinopatier.

Binyrebarkinsufficiens (primær og sekundær) er rapporteret hos patienter, der fik pembrolizumab. Hypofysitis er også rapporteret hos patienter, der fik pembrolizumab (se pkt. 4.8). Patienterne skal monitoreres for tegn og symptomer på binyrebarkinsufficiens og hypofysitis (inklusive hypofyseinsufficiens (hypopituitarisme), og andre årsager skal udelukkes. Kortikosteroider skal administreres for at behandle binyrebarkinsufficiens og anden hormonsubstitutionsbehandling som klinisk indiceret. Behandlingen med pembrolizumab skal pauseres ved grad 2-binyrebarkinsufficiens eller symptomatisk hypofysitis, indtil bivirkningen er kontrolleret med hormonsubstitutionsbehandling. Pembrolizumab skal pauseres eller seponeres ved grad 3- eller 4-binyrebarkinsufficiens eller -hypofysitis. Det kan overvejes at fortsætte behandlingen med pembrolizumab efter langsom udtrapning af kortikosterioder, hvis det er nødvendigt (se pkt. 4.2). Hypofysefunktion og hormonniveauer skal monitoreres for at sikre passende hormonsubstitutionsbehandling.

Type 1-diabetes mellitus, herunder diabetisk ketoacidose, er rapporteret hos patienter, der fik pembrolizumab (se pkt. 4.8). Patienterne skal monitoreres for hyperglykæmi eller andre tegn og symptomer på diabetes. Insulin skal administreres ved type 1-diabetes, og behandlingen med pembrolizumab skal pauseres i tilfælde af type 1-diabetes associeret med grad > 3-hyperglykæmi eller ketoacidose, indtil der er opnået metabolisk kontrol (se pkt. 4.2).

Thyreoideasygdomme, herunder hypotyroidisme, hypertyroidisme og tyroiditis, er rapporteret hos patienter, der fik pembrolizumab, og kan forekomme i hele behandlingsforløbet. Hypotyroidisme er rapporteret oftere hos patienter med HNSCC, som tidligere havde fået strålebehandling. Patienterne skal derfor monitoreres for ændringer i thyreoideafunktionen (ved behandlingsstart, periodevist i løbet af behandlingen og som indiceret på basis af den kliniske vurdering) samt for kliniske tegn og symptomer på thyreoideasygdomme. Hypotyroidisme kan substitutionsbehandles uden afbrydelse af behandlingen og uden kortikosteroider. Hypertyroidisme kan behandles symptomatisk. Pembrolizumab skal pauseres ved grad ≥3-hypertyroidisme indtil bedring til grad ≤ 1. Thyroideafunktion og hormonniveauer skal monitoreres for at sikre passende hormonsubstitutionsbehandling.

Hos patienter med grad 3- eller grad 4-endokrinopatier, som bedres til grad 2 eller derunder, og som er kontrolleret med hormonsubstitutionsbehandling, hvis det er indiceret, kan detovervejes at fortsætte behandlingen med pembrolizumab efter langsom udtrapning af kortikosteroider, hvis det er nødvendigt. Ellers skal behandlingen seponeres (se pkt. 4.2 og 4.8).

Immunrelaterede hudreaktioner
Alvorlige immunrelaterede hudreaktioner er rapporteret hos patienter, der fik pembrolizumab (se pkt. 4.8). Patienterne skal monitoreres ved mistanke om alvorlige hudreaktioner, og andre årsager skal udelukkes. På basis af bivirkningens sværhedsgrad skal pembrolizumab pauseres ved grad 3-hudreaktioner indtil bedring til grad ≤ 1 eller seponeres permanent ved grad 4-hudreaktioner, og kortikosteroider skal administreres (se pkt. 4.2).

Tilfælde af Stevens-Johnsons syndrom (SJS) og toksisk epidermal nekrolyse (TEN) er rapporteret hos patienter, der fik pembrolizumab (se pkt. 4.8). Ved mistanke om SJS eller TEN, skal behandling med

pembrolizumab pauseres, og patienten henvises til en specialafdeling til vurdering og behandling. Hvis mistanken om SJS eller TEN bekræftes, skal pembrolizumab seponeres permanent (se pkt. 4.2).

Der skal udvises forsigtighed, hvis pembrolizumab-behandling overvejes hos en patient, der har oplevet en alvorlig eller livstruende hudreaktion under tidligere behandling med andre immunstimulerende cancerlægemidler.

Andre immunrelaterede bivirkninger
Følgende yderligere klinisk signifikante, immunrelaterede bivirkninger er rapporteret i kliniske studier eller efter markedsføring: uveitis, artritis, myositis, myokarditis, pankreatitis, Guillain-Barrés syndrom, myastenisk syndrom, hæmolytisk anæmi, sarkoidose, encephalitis og myelitis (se pkt. 4.2 og 4.8).

Behandlingen med pembrolizumab skal pauseres ved grad 2- eller grad 3-bivirkninger og kortikosteroider administreres på basis af bivirkningens sværhedsgrad og type.

Behandlingen med pembrolizumab kan genoptages inden for 12 uger efter den sidste dosis af KEYTRUDA, hvis bivirkningen bedres til grad ≤ 1, og kortikosteroiddosis er blevet reduceret til ≤ 10 mg prednison dagligt eller ækvivalent hermed.

Behandlingen med pembrolizumab skal seponeres permanent ved en hvilken som helst immunrelateret bivirkning af grad 3, som recidiverer, og ved alle immunrelaterede bivirkninger af grad 4.

Pembrolizumab skal seponeres permanent ved grad 3- eller 4-myokarditis, encephalitis eller Guillain-Barrés syndrom (se pkt. 4.2 og 4.8).

Transplantationsrelaterede bivirkninger

Afstødning af transplanterede solide organer
Der er efter markedsføringen rapporteret om afstødning af transplanterede solide organer hos patienter, der er behandlet med PD-1-hæmmere. Behandling med pembrolizumab kan øge risikoen for afstødning hos modtagere af transplanterede solide organer. Fordelene ved behandling med pembrolizumab skal afvejes mod risikoen for mulig organafstødning hos disse patienter.

Komplikationer ved allogen hæmatopoietisk stamcelletransplantation (HSCT)

Allogen HSCT efter behandling med pembrolizumab
Tilfælde af *graft versus host*-sygdom (GVHD) og hepatisk veno-okklusiv sygdom (VOD) er observeret hos patienter med cHL, der gennemgår allogen HSCT efter tidligere eksponering for pembrolizumab. Indtil yderligere data bliver tilgængelige, skal de potentielle fordele ved HSCT og den potentielt øgede risiko for transplantationsrelaterede komplikationer vurderes nøje for hver enkelt patient (se pkt. 4.8).

Allogen HSCT før behandling med pembrolizumab
Hos patienter, der tidligere har gennemgået allogen HSCT, er der rapporteret akut GVHD, inklusive GVHD med dødelig udgang, efter behandling med pembrolizumab. Patienter, der udviklede GVHD efter deres transplantation, kan have øget risiko for GVHD efter behandling med pembrolizumab. Fordelene ved behandling med pembrolizumab skal afvejes mod risikoen for mulig GVHD hos patienter, der tidligere har gennemgået allogen HSCT.

Infusionsreaktioner
Alvorlige infusionsreaktioner, herunder overfølsomhed og anafylaksi, er rapporteret hos patienter, der fik pembrolizumab (se pkt. 4.8). Ved grad 3- eller 4-infusionsreaktioner skal infusionen stoppes og pembrolizumab seponeres permanent (se pkt. 4.2). Patienter med grad 1- eller 2-infusionsreaktioner kan fortsætte med at få pembrolizumab under tæt monitorering. Præmedicinering med antipyretikum og antihistamin kan overvejes.

Sygdomsspecifikke forholdsregler

Brug af pembrolizumab hos patienter med urotelialt karcinom, som tidligere har fået platinbaseret kemoterapi
Lægen bør overveje pembrolizumabs forsinkede indsættelse af virkning, før behandling initieres hos patienter med dårligere prognoser og/eller aggressiv sygdom. Ved urotelialt karcinom sås et højere antal dødsfald indenfor 2 måneder med pembrolizumab sammenlignet med kemoterapi (se pkt. 5.1). Faktorer associeret med tidlig død var hurtig progressiv sygdom efter tidligere platinbehandling og levermetastaser.

Brug af pembrolizumab ved urotelialt karcinom til patienter, som er uegnede til behandling med cisplatinbaseret kemoterapi, og hvis tumorer udtrykker PD-L1 med CPS ≥ 10
Prognostiske sygdomskarakteristika ved *baseline* hos studiepopulationen i KEYNOTE-052 inkluderede en procentdel patienter, der var egnet til carboplatinbaseret kombinationsbehandling, hvor fordelen bliver vurderet i et komparativt studie, og patienter, der var egnede til mono-kemoterapi, hvor der ikke foreligger randomiserede data. Derudover findes der ingen data vedrørende sikkerhed og virkning hos svagere patienter (f.eks. ECOG-performance-status 3), der ikke anses for egnede til behandling med kemoterapi. I mangel af disse data bør pembrolizumab anvendes med forsigtighed hos denne population efter nøje overvejelse af det potentielle benefit/risk-forhold på individuel basis.

Brug af pembrolizumab til førstelinjebehandling af patienter med NSCLC
Generelt forekommer bivirkninger hyppigere ved kombinationsbehandling med pembrolizumab end ved pembrolizumab som monoterapi eller kemoterapi alene, hvilket afspejler bidrag fra hver af de individuelle behandlinger (se pkt. 4.2 og 4.8). Der foreligger ikke en direkte sammenligning af pembrolizumab ved anvendelse i kombination med kemoterapi i forhold til pembrolizumab som monoterapi.

Lægen bør overveje benefit/risk-forholdet for de tilgængelige behandlingsmuligheder (pembrolizumab som monoterapi eller pembrolizumab i kombination med kemoterapi), før behandling initieres hos tidligere ubehandlede patienter med NSCLC, hvis tumorer udtrykker PD-L1.

Data vedrørende sikkerhed og virkning hos patienter ≥ 75 år er begrænsede. Kombinationsbehandling med pembrolizumab skal anvendes med forsigtighed hos patienter ≥ 75 år efter grundig overvejelse af benefit/risk-forholdet på individuel basis (se pkt. 5.1).

Brug af pembrolizumab til førstelinjebehandling af patienter med HNSCC
Generelt forekommer bivirkninger hyppigere ved kombinationsbehandling med pembrolizumab end ved pembrolizumab som monoterapi eller kemoterapi alene, hvilket afspejler bidrag fra hver af de individuelle behandlinger (se pkt. 4.8).

Lægen bør overveje benefit/risk-forholdet for de tilgængelige behandlingsmuligheder (pembrolizumab som monoterapi eller pembrolizumab i kombination med kemoterapi), før behandling initieres hos patienter med HNSCC, hvis tumorer udtrykker PD-L1 (se pkt. 5.1).

Brug af pembrolizumab til adjuverende behandling af melanom-patienter
En tendens til øget hyppighed af svære og alvorlige bivirkninger hos patienter ≥ 75 år blev observeret. Der er begrænsede data vedrørende sikkerhed fra patienter ≥ 75 år, som har fået adjuverende behandling for melanom.

Brug af pembrolizumab i kombination med axitinib til førstelinjebehandling af RCC-patienter
Ved administration af pembrolizumab i kombination med axitinib er der hos patienter med fremskredent RCC blevet rapporteret en højere end forventet forekomst af grad 3 og 4 forhøjelser af ALAT og ASAT (se pkt. 4.8). Leverenzymer skal monitoreres før initiering af behandlingen og med jævne mellemrum under behandlingen. Det kan overvejes at udføre hyppigere monitorering af leverenzymer i forhold til, når lægemidlerne anvendes som monoterapi. Retningslinjerne for medicinsk behandling for begge lægemidler skal følges (se pkt. 4.2 og produktresuméet for axitinib).

Patienter, der blev ekskluderet fra kliniske studier
Patienter med følgende tilstande blev ekskluderet fra kliniske studier: patienter med aktive metastaser i centralnervesystemet; patienter med ECOG-performance-status ≥ 2 (med undtagelse af urotelialt karcinom og RCC); patienter med hiv-infektion, hepatitis B- eller hepatitis C-infektion; patienter med aktiv systemisk autoimmun sygdom; patienter med interstitiel lungesygdom; patienter med pneumonitis i anamnesen, der krævede behandling med systemiske kortikosteroider; patienter, der tidligere har haft svær overfølsomhed over for andre monoklonale antistoffer; patienter i immunsuppressiv behandling samt patienter, der tidligere har haft alvorlige immunrelaterede bivirkninger ved behandling med ipilimumab, defineret som toksicitet af grad 4 eller af grad 3, hvor kortikosteroider (> 10 mg/dag af prednison eller ækvivalent hermed) i mere end 12 uger var nødvendig. Patienter med aktive infektioner blev ekskluderet fra kliniske studier, og det var en forudsætning, at infektionen blev behandlet, inden de kunne få pembrolizumab. Patienter, hvor aktive infektioner indtrådte under behandlingen med pembrolizumab, blev behandlet med passende medicinsk behandling. Patienter med klinisk signifikant nedsat nyrefunktion (kreatinin > 1,5 x ULN) eller leverfunktion (bilirubin > 1,5 x ULN, ALAT, ASAT > 2,5 x ULN med fravær af levermetastaser) ved *baseline* blev ekskluderet fra kliniske studier. Derfor foreligger der kun begrænset information om patienter med svært nedsat nyrefunktion og moderat til svært nedsat leverfunktion.

Der foreligger begrænsede data om KEYTRUDAs sikkerhed og virkning hos patienter med okulært melanom (se pkt. 5.1).

For så vidt angår recidiverende eller refraktært cHL foreligger der kun begrænsede kliniske data for anvendelse af pembrolizumab hos patienter, som er uegnede til ASCT af andre årsager end behandlingssvigt efter salvage-kemoterapi (se pkt. 5.1).

Efter nøje overvejelse af den potentielt øgede risiko kan pembrolizumab anvendes til disse patienter med passende klinisk håndtering.

Patientinformationskort
Alle læger, som påtænker at ordinere KEYTRUDA, skal sikre sig kendskab til indholdet i informationen til lægen og behandlingsvejledningen. Lægen skal drøfte risiciene ved KEYTRUDA-behandlingen med patienten. Patienten skal have udleveret patientinformationskortet ved hver ordination.

4.5 Interaktion med andre lægemidler og andre former for interaktion

Der er ikke udført formelle farmakokinetiske lægemiddelinteraktionsstudier med pembrolizumab. Eftersom pembrolizumab elimineres fra kredsløbet via nedbrydning (katabolisme) forventes ingen metaboliske lægemiddelinteraktioner.

Systemiske kortikosteroider og immunsuppressiva bør undgås før initiering af behandling med pembrolizumab på grund af deres potentielle interferens med pembrolizumabs farmakodynamiske aktivitet og virkning. Systemiske kortikosteroider og andre immunsuppressiva kan dog anvendes til at behandle immunrelaterede bivirkninger efter initiering af pembrolizumab (se pkt. 4.4). Kortikosteroider kan også anvendes som præmedicinering, når pembrolizumab anvendes i kombination med kemoterapi, som profylaktisk antiemetisk behandling og/eller til at mildne kemoterapi-relaterede bivirkninger.

4.6 Fertilitet, graviditet og amning

Kvinder i den fertile alder
Kvinder i den fertile alder skal anvende sikker kontraception under behandlingen med pembrolizumab og i mindst 4 måneder efter den sidste pembrolizumab-dosis.

Graviditet
Der er ingen data fra anvendelse af pembrolizumab til gravide kvinder. Der har ikke været udført reproduktionsstudier med pembrolizumab hos dyr. I dyremodeller med drægtige mus er det blevet påvist, at blokering af PD-L1-signalet kan påvirke tolerancen for fostret og medføre et stigende antal fostertab (se pkt. 5.3). Med udgangspunkt i pembrolizumabs virkningsmekanisme viser disse resultater en potentiel

risiko for, at administration af pembrolizumab under graviditet kan medføre fosterskader, herunder en øget forekomst af abort eller dødfødsel. Det er vist, at humant immunglobulin G4 (IgG4) kan passere placentabarrieren, og da pembrolizumab er et IgG4, kan pembrolizumab derfor potentielt overføres fra moderen til fosteret. Pembrolizumab bør ikke anvendes under graviditet, medmindre kvindens kliniske tilstand kræver behandling med pembrolizumab.

Amning
Det er ukendt, om pembrolizumab udskilles i human mælk. En risiko for nyfødte/spædbørn kan ikke udelukkes, da det er kendt, at antistoffer kan udskilles i human mælk. Det skal besluttes, om amning skal ophøre eller behandling med pembrolizumab skal seponeres, idet der tages højde for fordelene ved amning for barnet i forhold til de terapeutiske fordele ved pembrolizumab-behandlingen for moderen.

Fertilitet
Der foreligger ingen data om pembrolizumabs mulige indvirkning på fertilitet. Toksicitetsstudier af 1 måned og 6 måneders varighed med gentagne doser viste ingen væsentlig indvirkning på forplantningsorganerne hos han- og hunaber (se pkt. 5.3).

4.7 Virkning på evnen til at føre motorkøretøj og betjene maskiner

Pembrolizumab påvirker i mindre grad evnen til at føre motorkøretøj og betjene maskiner. Svimmelhed og træthed er rapporteret hos nogle patienter efter administration af pembrolizumab (se pkt. 4.8).

4.8 Bivirkninger

Oversigt over sikkerhedsprofilen
Pembrolizumab er sædvanligvis associeret med immunrelaterede bivirkninger. De fleste af disse, herunder alvorlige bivirkninger, gik over efter initiering af passende medicinsk behandling eller pausering af behandlingen af pembrolizumab (se "Beskrivelse af udvalgte bivirkninger" nedenfor).

Pembrolizumabs sikkerhed som monoterapi er blevet vurderet hos 5.884 patienter med fremskredent melanom, resekteret stadie III-melanom (adjuverende behandling), NSCLC, cHL, urotelialt karcinom eller HNSCC ved 4 doser (2 mg/kg hver 3. uge, 200 mg hver 3. uge eller 10 mg/kg hver 2. eller 3. uge) i kliniske studier. Hyppighederne, som er inkluderet nedenfor og i tabel 2, er baseret på alle rapporterede bivirkninger, uden hensyn til investigators vurdering af kausalitet. I denne patientpopulation var den mediane observationstid 7,3 måneder (interval: 1 dag til 31 måneder), og de hyppigste bivirkninger med pembrolizumab var: træthed (32%), kvalme (20%) og diarré (20%). Størstedelen af de rapporterede bivirkninger ved monoterapi var af sværhedsgrad 1 eller 2. De alvorligste bivirkninger var immunrelaterede bivirkninger og alvorlige infusionsreaktioner (se pkt. 4.4).

Pembrolizumabs sikkerhed i kombination med kemoterapi er blevet vurderet hos 1.067 patienter med NSCLC eller HNSCC, der fik 200 mg, 2 mg/kg eller 10 mg/kg pembrolizumab hver 3. uge, i kliniske studier. Hyppighederne, som er inkluderet nedenfor og i tabel 2, er baseret på alle rapporterede bivirkninger, uden hensyn til investigators vurdering af kausalitet. I denne patientpopulation var de hyppigste bivirkninger: anæmi (50%), kvalme (50%), træthed (37%), obstipation (35%), diarré (30%), neutropeni (30%), appetitløshed (28%) og opkastning (25%). Forekomsten af bivirkninger af grad 3-5 hos patienter med NSCLC var 67% for kombinationsbehandling med pembrolizumab og 66% for kemoterapi alene og forekomsten hos patienter med HNSCC var 85% for kombinationsbehandling med pembrolizumab og 84% for kemoterapi plus cetuximab.

Pembrolizumabs sikkerhed i kombination med axitinib er blevet vurderet i et klinisk studie med 429 patienter med fremskredent RCC, der fik 200 mg pembrolizumab hver 3. uge og 5 mg axitinib to gange dagligt. I denne patientpopulation var de hyppigste bivirkninger: diarré (54%), hypertension (45%), træthed (38%), hypotyroidisme (35%), appetitløshed (30%), palmoplantar erytrodysæstesisyndrom (hånd-fod-syndrom) (28%), kvalme (28%), forhøjet ALAT (27%), forhøjet ASAT (26%), dysfoni (25%), hoste (21%) og obstipation (21%). Forekomsten af bivirkninger af grad 3-5 var 76% for kombinationsbehandling med pembrolizumab og 71% for sunitinib alene.

Bivirkningstabel

De bivirkninger, der blev observeret i kliniske studier med pembrolizumab som monoterapi eller i kombination med kemoterapi eller andre anti-tumor lægemidler eller rapporteret i forbindelse med brug af pembrolizumab efter markedsføring, er opført i tabel 2. Bivirkninger, der vides at forekomme ved behandling med pembrolizumab eller ved kemoterapi givet alene, kan forekomme under behandlingen med disse lægemidler i kombination, selvom disse bivirkninger ikke blev rapporteret i kliniske studier med kombinationsbehandling. Disse bivirkninger er anført ud fra systemorganklasse og hyppighed. Hyppigheden er angivet som: Meget almindelig (≥ 1/10); Almindelig (≥ 1/100 til < 1/10); Ikke almindelig (≥ 1/1.000 til < 1/100); Sjælden (≥ 1/10.000 til < 1/1.000); Meget sjælden (< 1/10.000) og Ikke kendt (kan ikke estimeres ud fra forhåndenværende data). I hver hyppighedsgruppe er bivirkningerne opstillet efter faldende alvorlighed.

Tabel 2: Bivirkninger hos patienter, som blev behandlet med pembrolizumab*

	Monoterapi	Kombination med kemoterapi	Kombination med axitinib
**Infektioner og parasitære sygdomme**			
Almindelig	pneumoni	pneumoni	pneumoni
**Blod og lymfesystem**			
Meget almindelig	anæmi	anæmi, neutropeni, trombocytopeni	
Almindelig	trombocytopeni, lymfopeni	febril neutropeni, leukopeni, lymfopeni	anæmi, neutropeni, leukopeni, trombocytopeni
Ikke almindelig	neutropeni, leukopeni, eosinofili		lymfopeni, eosinofili
Sjælden	immun trombocytopenisk purpura, hæmolytisk anæmi, ren erytrocyt aplasi, hæmofagocytisk lymfohistiocytose	eosinofili	
**Immunsystemet**			
Almindelig	infusionsreaktioner[a]	infusionsreaktioner[a]	infusionsreaktioner[a]
Ikke almindelig	sarkoidose		
Ikke kendt	afstødning af transplanterede solide organer		
**Det endokrine system**			
Meget almindelig	hypotyroidisme[b]		hypertyroidisme, hypotyroidisme[b]
Almindelig	hypertyroidisme	hypotyroidisme, hypertyroidisme	hypofysitis[d], tyroiditis[e], binyrebarkinsufficiens[c]
Ikke almindelig	binyrebarkinsufficiens[c], hypofysitis[d], tyroiditis[e]	hypofysitis[d], tyroiditis[e], binyrebarkinsufficiens[c]	
**Metabolisme og ernæring**			
Meget almindelig	appetitløshed	hypokaliæmi, appetitløshed	appetitløshed
Almindelig	hyponatriæmi, hypokaliæmi, hypokalcæmi	hyponatriæmi, hypokalcæmi	hypokaliæmi, hyponatriæmi, hypokalcæmi
Ikke almindelig	type 1-diabetes mellitus[f]	type 1-diabetes mellitus	type 1-diabetes mellitus[f]
**Psykiske forstyrrelser**			
Almindelig	insomni	insomni	insomni
**Nervesystemet**			
Meget almindelig	hovedpine	svimmelhed, hovedpine, perifer neuropati, dysgeusi	hovedpine, dysgeusi

13

Almindelig	svimmelhed, perifer neuropati, letargi, dysgeusi	letargi	svimmelhed, letargi, perifer neuropati
Ikke almindelig	epilepsi	epilepsi	myastenisk syndrom[i]
Sjælden	encephalitis, Guillain-Barrés syndrom[g], myelitis[h], myastenisk syndrom[i], meningitis (aseptisk)[j]		
**Øjne**			
Almindelig	øjentørhed	øjentørhed	øjentørhed
Ikke almindelig	uveitis[k]		uveitis[k]
Sjælden	Vogt-Koyanagi-Harada-syndrom		
**Hjerte**			
Almindelig	hjertearytmi[f] (inklusive atrieflimren)	hjertearytmi[f] (inklusive atrieflimren)	hjertearytmi[f] (inklusive atrieflimren)
Ikke almindelig	perikardieeffusion, perikarditis	perikardieeffusion	myokarditis[l]
Sjælden	myokarditis[l]	myokarditis[l], perikarditis	
**Vaskulære sygdomme**			
Meget almindelig			hypertension
Almindelig	hypertension	hypertension	
**Luftveje, thorax og mediastinum**			
Meget almindelig	dyspnø, hoste	dyspnø, hoste	dyspnø, hoste, dysfoni
Almindelig	pneumonitis[m]	pneumonitis[m]	pneumonitis[m]
**Mave-tarm-kanalen**			
Meget almindelig	diarré, abdominalsmerter[n], kvalme, opkastning, obstipation	diarré, kvalme, opkastning, obstipation, abdominalsmerter[n]	diarré, abdominalsmerter[n], kvalme, opkastning, obstipation
Almindelig	colitis[o], mundtørhed	colitis[o], mundtørhed	colitis[o], mundtørhed
Ikke almindelig	pankreatitis[p], gastrointestinal ulceration[q]	pankreatitis[p], gastrointestinal ulceration[q]	pankreatitis[p], gastrointestinal ulceration[q]
Sjælden	perforation af tyndtarmen		
**Lever og galdeveje**			
Almindelig			hepatitis[r]
Ikke almindelig	hepatitis[r]	hepatitis[r]	
**Hud og subkutane væv**			
Meget almindelig	udslæt[s], pruritus[t]	udslæt[s], alopeci, pruritus[t]	palmoplantar erytrodysæstesisyndrom (hånd-fod-syndrom), udslæt[s], pruritus[t]
Almindelig	alvorlige hudreaktioner[u], erytem, tør hud, vitiligo[v], eksem, alopeci, akneiform dermatitis	alvorlige hudreaktioner[u], erytem, tør hud	alvorlige hudreaktioner[u], akneiform dermatitis, dermatitis, tør hud, alopeci, eksem, erytem
Ikke almindelig	likenoid keratose[w], psoriasis, dermatitis, papel, ændret hårfarve	psoriasis, akneiform dermatitis, dermatitis, vitiligo[v], eksem	ændret hårfarve, likenoid keratose, papel, psoriasis, vitiligo[v]
Sjælden	toksisk epidermal nekrolyse, Stevens-Johnsons syndrom, erythema nodosum	ændret hårfarve, likenoid keratose, papel	

14

**Knogler, led, muskler og bindevæv**			
Meget almindelig	muskuloskeletale smerter[x], artralgi	muskuloskeletale smerter[x], artralgi	muskuloskeletale smerter[x], artralgi, ekstremitetssmerter
Almindelig	ekstremitetssmerter, myositis[y], artritis[z]	myositis[y], ekstremitetssmerter, artritis[z]	myositis[y], artritis[z], tenosynovitis[aa]
Ikke almindelig	tenosynovitis[aa]	tenosynovitis[aa]	
**Nyrer og urinveje**			
Almindelig		nefritis[bb], akut nyreskade	akut nyreskade, nefritis[bb]
Ikke almindelig	nefritis[bb]		
**Almene symptomer og reaktioner på administrationsstedet**			
Meget almindelig	træthed, asteni, ødem[cc], pyreksi	træthed, asteni, pyreksi, ødem[cc]	træthed, asteni, pyreksi
Almindelig	influenzalignende sygdom, kuldegysninger	kuldegysninger, influenzalignende sygdom	ødem[cc], influenzalignende sygdom, kuldegysninger
**Undersøgelser**			
Meget almindelig		forhøjet kreatinin i blodet	forhøjet alaninaminotransferase, forhøjet aspartataminotransferase, forhøjet kreatinin i blodet
Almindelig	forhøjet aspartataminotransferase, forhøjet alaninaminotransferase, hyperkalcæmi, forhøjet alkalisk fosfatase i blodet, forhøjet bilirubin i blodet, forhøjet kreatinin i blodet	hyperkalcæmi, forhøjet alaninaminotransferase, forhøjet aspartataminotransferase, forhøjet alkalisk fosfatase i blodet	forhøjet alkalisk fosfatase i blodet, hyperkalcæmi, forhøjet bilirubin i blodet
Ikke almindelig	forhøjet amylase	forhøjet bilirubin i blodet, forhøjet amylase	forhøjet amylase

*Bivirkningshyppighederne, som er angivet i tabel 2, kan muligvis ikke udelukkende henføres til pembrolizumab alene, men kan måske også indeholde bidrag fra den underliggende sygdom eller fra andre lægemidler, som anvendes i kombination.

†Baseret på en standardforespørgsel inklusive bradyarytmier og takyarytmier.

Følgende termer repræsenterer en gruppe beslægtede bivirkninger, som snarere beskriver en medicinsk tilstand end en enkelt bivirkning.

a. infusionsreaktion (lægemiddeloverfølsomhed, anafylaktisk reaktion, anafylaktoid reaktion, overfølsomhed og cytokin-frigivelsessyndrom)
b. hypotyroidisme (myksødem)
c. binyrebarkinsufficiens (Addisons sygdom, akut binyrebarkinsufficiens, sekundær binyrebarkinsufficiens)
d. hypofysitis (hypopituitarisme)
e. tyroiditis (autoimmun tyroiditis og thyroideasygdom)
f. type 1-diabetes mellitus (diabetisk ketoacidose)
g. Guillain-Barrés syndrom (axonal neuropati og demyeliniserende polyneuropati)
h. myelitis (inklusive transversel myelitis)
i. myastenisk syndrom (myasthenia gravis, inklusive eksacerbation)
j. aseptisk meningitis (meningitis, non-infektiøs meningitis)
k. uveitis (iritis og iridocyklitis)
l. myokarditis (autoimmun myokarditis)
m. pneumonitis (interstitiel lungesygdom)
n. abdominalsmerter (abdominalgener, øvre abdominalsmerter og nedre abdominalsmerter)
o. colitis (mikroskopisk colitis, enterocolitis, hæmoragisk enterocolitis og autoimmun colitis)
p. pankreatitis (autoimmun pankreatitis og akut pankreatitis)
q. gastrointestinal ulceration (ulcus ventriculi og ulcus duodeni)
r. hepatitis (autoimmun hepatitis, immunmedieret hepatitis og lægemiddelinduceret leverskade)
s. udslæt (erytematøst udslæt, follikulært udslæt, generaliseret udslæt, makuløst udslæt, makulopapuløst udslæt, papuløst udslæt, pruritisk udslæt, vesikuløst udslæt og genitalt udslæt)
t. pruritus (urticaria, papuløs urticaria, generaliseret pruritus og genital pruritus)
u. alvorlige hudreaktioner (bulløs dermatit, eksfoliativ dermatitis, erythema multiforme, eksfoliativt udslæt, pemphigus, hudnekrose, toksisk hududslæt samt ≥ grad 3 af følgende: akut febril neutrofil dermatose, kontusion, liggesår,

psoriasiform dermatitis, lægemiddeludslæt, gulsot, pemfigoid, pruritus, generaliseret pruritus, udslæt, erytematøst udslæt, generaliseret udslæt, makulopapuløst udslæt, pruritisk udslæt, pustuløst udslæt og hudlæsion)

v. vitiligo (depigmentering af huden, hypopigmentering af huden og hypopigmentering af øjenlåget)
w. likenoid keratose (lichen planus og lichen sclerosus)
x. muskuloskeletale smerter (muskuloskeletale gener, rygsmerter, muskuloskeletal stivhed, muskuloskeletale brystsmerter og torticollis)
y. myositis (myalgi, myopati, polymyalgia rheumatica og rabdomyolyse)
z. artritis (hævede led, polyartritis og ledeffusion)
aa. tenosynovitis (tendinitis, synovitis og senesmerter)
bb. nefritis (autoimmun nefritis, tubulointerstitiel nefritis og nyresvigt, akut nyresvigt eller akut nyreskade med tegn på nefritis, nefrotisk syndrom og membranøs glomerulonefritis)
cc. ødem (perifert ødem, generaliseret ødem, overhydrering, væskeretention, øjenlågsødem og læbeødem, ansigtsødem, lokaliseret ødem og periorbitalt ødem)

Beskrivelse af udvalgte bivirkninger

Dataene for følgende immunrelaterede bivirkninger er baseret på patienter, som har fået pembrolizumab i kliniske studier (4 doseringsregimer: 2 mg/kg hver 3. uge, 10 mg/kg hver 2. eller 3. uge eller 200 mg hver 3. uge) (se pkt. 5.1). Vejledning til håndtering af disse bivirkninger findes i pkt. 4.4.

Immunrelaterede bivirkninger (se pkt. 4.4)

Immunrelateret pneumonitis

Pneumonitis forekom hos 253 (4,3%) patienter, som fik pembrolizumab, inklusive tilfælde af grad 2, 3, 4 eller 5 hos henholdsvis 106 (1,8%), 69 (1,2%), 13 (0,2%) og 9 (0,2%) patienter. Den mediane tid til indtræden af pneumonitis var 3,3 måneder (interval: 2 dage til 26,8 måneder). Medianvarigheden var 1,9 måneder (interval: 1 dag til 25,3+ måneder). Pneumonitis forekom hyppigere hos patienter, der tidligere havde fået torakal strålebehandling (8,1%) end hos patienter, der ikke tidligere havde fået torakal strålebehandling (3,4%). Pneumonitis medførte seponering af pembrolizumab hos 98 (1,7%) patienter. Pneumonitis gik over hos 138 patienter, 2 med sequelae.

Hos patienter med NSCLC forekom pneumonitis hos 107 (4,9%), inklusive tilfælde af grad 2, 3, 4 eller 5 hos henholdsvis 39 (1,8%), 30 (1,4%), 10 (0,5%) og 9 (0,4%) patienter. Hos patienter med NSCLC forekom pneumonitis hos 8,1% af patienterne, der tidligere havde fået torakal strålebehandling.

Immunrelateret colitis

Colitis forekom hos 107 (1,8%) patienter, som fik pembrolizumab, inklusive tilfælde af grad 2, 3 eller 4 hos henholdsvis 31 (0,5%), 62 (1,1%) og 3 (< 0,1%) patienter. Den mediane tid til indtræden af colitis var 4,3 måneder (interval: 7 dage til 24,3 måneder). Medianvarigheden var 0,9 måneder (interval: 1 dag til 8,7+ måneder). Colitis medførte seponering af pembrolizumab hos 29 (0,5%) patienter. Colitis gik over hos 84 patienter, 2 med sequelae.

Immunrelateret hepatitis

Hepatitis forekom hos 50 (0,8%) patienter, som fik pembrolizumab, inklusive tilfælde af grad 2, 3 eller 4 hos henholdsvis 8 (0,1%), 31 (0,5%) og 8 (0,1%) patienter. Den mediane tid til indtræden af hepatitis var 3,6 måneder (interval: 8 dage til 21,4 måneder). Medianvarigheden var 1,1 måneder (interval: 1 dag til 20,9+ måneder). Hepatitis medførte seponering af pembrolizumab hos 19 (0,3%) patienter. Hepatitis gik over hos 36 patienter.

Immunrelateret nefritis

Nefritis forekom hos 22 (0,4%) patienter, som fik pembrolizumab som monoterapi, inklusive tilfælde af grad 2, 3 eller 4 hos henholdsvis 5 (0,1%), 14 (0,2%) og 1 (< 0,1%) patienter. Den mediane tid til indtræden af nefritis var 5,0 måneder (interval: 12 dage til 21,4 måneder). Medianvarigheden var 2,6 måneder (interval: 6 dage til 12,0 måneder). Nefritis medførte seponering af pembrolizumab hos 10 (0,2%) patienter. Nefritis gik over hos 13 patienter, 3 med sequelae. Hos patienter med ikke-planocellulær NSCLC, som fik pembrolizumab i kombination med pemetrexed og platinbaseret kemoterapi (n=488), var forekomsten af nefritis 1,4% (alle grader) med 0,8% af grad 3 og 0,4% af grad 4.

Immunrelaterede endokrinopatier

Binyrebarkinsufficiens forekom hos 46 (0,8%) patienter, som fik pembrolizumab, inklusive tilfælde af grad 2, 3 eller 4 hos henholdsvis 19 (0,3%), 20 (0,3%) og 3 (0,1%) patienter. Den mediane tid til

indtræden af binyrebarkinsufficiens var 5,4 måneder (interval: 1 dag til 17,7 måneder). Medianvarigheden blev ikke nået (interval: 3 dage til 26,2+ måneder). Binyrebarkinsufficiens medførte seponering af pembrolizumab hos 4 (0,1%) patienter. Binyrebarkinsufficiens gik over hos 16 patienter, 4 med sequelae.

Hypofysitis forekom hos 36 (0,6%) patienter, som fik pembrolizumab, inklusive tilfælde af grad 2, 3 eller 4 hos henholdsvis 13 (0,2%), 19 (0,3%) og 1 (< 0,1%) patienter. Den mediane tid til indtræden af hypofysitis var 5,9 måneder (interval: 1 dag til 17,7 måneder). Medianvarigheden var 3,3 måneder (interval: 3 dage til 18,1+ måneder). Hypofysitis medførte seponering af pembrolizumab hos 8 (0,1%) patienter. Hypofysitis gik over hos 17 patienter, 8 med sequelae.

Hypertyroidisme forekom hos 244 (4,1%) patienter, som fik pembrolizumab, inklusive tilfælde af grad 2 eller 3 hos henholdsvis 64 (1,1%) og 7 (0,1%) patienter. Den mediane tid til indtræden af hypertyroidisme var 1,4 måneder (interval: 1 dag til 22,5 måneder). Medianvarigheden var 1,8 måneder (interval: 4 dage til 29,2+ måneder). Hypertyroidisme medførte seponering af pembrolizumab hos 3 (0,1%) patienter. Hypertyroidisme gik over hos 191 (78,3%) patienter, 5 med sequelae.

Hypotyroidisme forekom hos 645 (11,0%) patienter, som fik pembrolizumab, inklusive tilfælde af grad 2 eller 3 hos henholdsvis 475 (8,1%) og 8 (0,1%) patienter. Den mediane tid til indtræden af hypotyroidisme var 3,5 måneder (interval: 1 dag til 19,6 måneder). Medianvarigheden blev ikke nået (interval: 2 dage til 32,6+ måneder). 2 patienter (< 0,1%) seponerede pembrolizumab som følge af hypotyroidisme. Hypotyroidisme gik over hos 153 (23,7%) patienter, 10 med sequelae. Hos patienter med cHL (n=241) var forekomsten af hypotyroidisme 14,1% (alle grader) med 0,4% af grad 3. Hos patienter med HNSCC, som blev behandlet med pembrolizumab som monoterapi (n=909), var forekomsten af hypotyroidisme 16,1% (alle grader) med 0,3% af grad 3. Hos patienter med HNSCC, som blev behandlet med pembrolizumab i kombination med platinbaseret kemoterapi og 5-FU (n=276), var forekomsten af hypotyroidisme 15,2%, hvoraf alle var af grad 1 eller 2.

Immunrelaterede hudreaktioner
Alvorlige immunrelaterede hudreaktioner forekom hos 89 (1,5%) patienter, som fik pembrolizumab, inklusive tilfælde af grad 2, 3 eller 5 hos henholdsvis 10 (0,2%), 65 (1,1%) og 1 (< 0,1%) patienter. Den mediane tid til indtræden af alvorlige hudreaktioner var 3,3 måneder (interval: 3 dage til 19,4 måneder). Medianvarigheden var 1,6 måneder (interval: 1 dag til 27,3+ måneder). Alvorlige hudreaktioner medførte seponering af pembrolizumab hos 9 (0,2%) patienter. Alvorlige hudreaktioner gik over hos 64 patienter.

Der er observeret sjældne tilfælde af SJS og TEN, herunder tilfælde med dødelig udgang (se pkt. 4.2 og 4.4).

Komplikationer ved allogen HSCT ved cHL
Ud af 23 patienter med cHL, som gik videre til allogen HSCT efter behandling med pembrolizumab, udviklede 6 patienter (26%) GVHD, hvoraf en døde, og 2 patienter (9%) fik svær hepatisk VOD efter konditionering med reduceret intensitet, hvoraf en døde. De 23 patienter havde en median opfølgningstid fra efterfølgende allogen HSCT på 5,1 måneder (interval: 0-26,2 måneder).

Forhøjede leverenzymer, når pembrolizumab anvendes i kombination med axitinib ved RCC
I et klinisk studie af tidligere ubehandlede RCC-patienter, der fik pembrolizumab i kombination med axitinib, blev der observeret en højere end forventet forekomst af grad 3 og 4 forhøjelser af ALAT (20%) og ASAT (13%). Den mediane tid til indtræden af forhøjet ALAT var 2,3 måneder (interval: 7 dage til 19,8 måneder). Hos patienter med ALAT ≥ 3 gange ULN (grad 2-4, n=116) forbedredes ALAT til grad 0-1 hos 94%. 59% af patienterne med forhøjet ALAT fik systemiske kortikosteroider. 92 (84%) af de patienter, der bedredes, fik genoptaget behandlingen med enten pembrolizumab (3%) eller axitinib (31%) som monoterapi eller med begge (50%). 55% af disse patienter havde ingen recidiv af ALAT > 3 gange ULN, og bedring forekom hos alle patienter med recidiv af ALAT > 3 gange ULN. Der var ingen hepatiske hændelser af grad 5.

Unormale laboratorieværdier
Hos patienter, der blev behandlet med pembrolizumab som monoterapi, var andelen af patienter, som oplevede et skift fra *baseline* til en grad 3 eller 4 unormal laboratorieværdi, følgende: 10,9% med nedsat

lymfocyttal; 8,2% med nedsat natrium; 6,3% med nedsat hæmoglobin; 5,2% med nedsat fosfat; 4,8% med forhøjet glucose; 2,8% med forhøjet ASAT; 2,7% med forhøjet alkalisk fosfatase; 2,7% med forhøjet ALAT; 2,2% med nedsat kalium; 1,8% med forhøjet kalcium; 1,8% med nedsat neutrofiltal; 1,8% med forhøjet kalium; 1,7% med forhøjet bilirubin; 1,7% med nedsat trombocyttal; 1,6% med nedsat albumin; 1,5% med nedsat kalcium; 1,3% med forhøjet kreatinin; 0,8% med nedsat leukocyttal; 0,7% med forhøjet magnesium; 0,6% med nedsat glucose; 0,2% med nedsat magnesium og 0,2% med forhøjet natrium.

Hos patienter, der blev behandlet med pembrolizumab i kombination med kemoterapi, var andelen af patienter, som oplevede et skift fra *baseline* til unormal laboratorieværdi af grad 3 eller 4, følgende: 26,7% med nedsat neutrofiltal; 23,9% med nedsat lymfocyttal; 19,1% med nedsat hæmoglobin; 17,9% med nedsat leukocyttal; 12,2% med nedsat trombocyttal; 10,2% med nedsat natrium; 8,9% med nedsat fosfat; 7,4% med forhøjet glucose; 6,5% med nedsat kalium; 3,3% med forhøjet kreatinin; 3,1% med forhøjet ALAT; 3,1% med forhøjet ASAT; 3,1% med nedsat kalcium; 3,0% med forhøjet kalium; 2,9% med nedsat albumin; 2,3% med forhøjet kalcium; 1,2% med forhøjet alkalisk fosfatase; 0,8% med nedsat glucose; 0,7% med forhøjet bilirubin og 0,3% med forhøjet natrium.

Hos patienter, der blev behandlet med pembrolizumab i kombination med axitinib, var andelen af patienter, som oplevede et skift fra *baseline* til en grad 3 eller 4 unormal laboratorieværdi, følgende: 20,1% med forhøjet ALAT; 13,2% med forhøjet ASAT; 10,8% med nedsat lymfocyttal; 8,9% med forhøjet glucose; 7,8% med nedsat natrium; 6,4% med nedsat fosfat; 6,2% med forhøjet kalium; 4,3% med forhøjet kreatinin; 3,6% med nedsat kalium; 2,1% med forhøjet bilirubin; 2,1% med nedsat hæmoglobin; 1,7% med forhøjet alkalisk fosfatase; 1,5% med forhøjet INR (protrombintid); 1,4% med nedsat leukocyttal; 1,4% med nedsat trombocyttal; 1,2% med forlænget aktiveret partiel tromboplastintid (APTT); 1,2% med nedsat neutrofiltal; 1,2% med forhøjet natrium; 0,7% med nedsat kalcium; 0,7% med forhøjet kalcium; 0,5% med nedsat albumin og 0,2% med nedsat glucose.

Immunogenicitet
I kliniske studier med patienter, som blev behandlet med pembrolizumab 2 mg/kg hver 3. uge, 200 mg hver 3. uge eller 10 mg/kg hver 2. eller 3. uge som monoterapi, havde 36 (1,8%) ud af 2.034 evaluerbare patienter en positiv test for antistoffer mod pembrolizumab udviklet under behandlingen, hvoraf 9 (0,4%) patienter havde neutraliserende antistoffer mod pembrolizumab. Der sås ingen tegn på ændret farmakokinetisk profil eller sikkerhedsprofil i forbindelse med anti-pembrolizumab binding eller dannelse af neutraliserende antistoffer mod pembrolizumab.

Pædiatrisk population
Pembrolizumabs sikkerhed som monoterapi blev vurderet hos 154 pædiatriske patienter med fremskredent melanom, lymfom eller PD-L1-positive fremskredne, recidiverende eller refraktære solide tumorer ved en dosis på 2 mg/kg hver 3. uge i fase I/II-studiet KEYNOTE-051. Sikkerhedsprofilen hos disse pædiatriske patienter var generelt sammenlignelig med sikkerhedsprofilen hos voksne, der blev behandlet med pembrolizumab. De hyppigste bivirkninger (rapporteret hos mindst 20% af de pædiatriske patienter) var pyreksi (31%), opkastning (26%), hovedpine (22%), abdominalsmerter (21%), anæmi (21%) og obstipation (20%). Størstedelen af de rapporterede bivirkninger ved monoterapi var af sværhedsgrad 1 eller 2. 69 (44,8%) patienter havde 1 eller flere bivirkninger af grad 3 til 5, hvoraf 6 (3,9%) patienter havde 1 eller flere bivirkninger med dødelig udgang. Hyppighederne er baseret på alle rapporterede bivirkninger, uden hensyn til investigators vurdering af kausalitet.

Indberetning af formodede bivirkninger
Når lægemidlet er godkendt, er indberetning af formodede bivirkninger vigtig. Det muliggør løbende overvågning af benefit/risk-forholdet for lægemidlet. Sundhedspersoner anmodes om at indberette alle formodede bivirkninger via det nationale rapporteringssystem anført i Appendiks V.

4.9 Overdosering

Der findes ingen information vedrørende overdosering med pembrolizumab.

I tilfælde af overdosering skal patienten monitoreres tæt for tegn og symptomer på bivirkninger, og passende symptomatisk behandling iværksættes.

5. FARMAKOLOGISKE EGENSKABER

5.1 Farmakodynamiske egenskaber

Farmakoterapeutisk klassifikation: antineoplastiske stoffer, monoklonale antistoffer, ATC-kode: L01XC18

Virkningsmekanisme
KEYTRUDA er et humaniseret monoklonalt antistof, der binder til *programmed cell death*-1 (PD-1)-receptoren og blokerer dets interaktion med liganderne PD-L1 og PD-L2. PD-1-receptoren er en negativ regulator af T-celleaktivering, som er påvist at være involveret i kontrollen af T-cellemedieret immunrespons. KEYTRUDA aktiverer T-cellemedieret respons, herunder anti-tumorrespons, ved at blokere PD-1-bindingen til PD-L1 og PD-L2, som er udtrykt i antigenpræsenterende celler, og som kan udtrykkes af tumorer eller andre celler i tumorens mikromiljø.

Klinisk virkning og sikkerhed
Pembrolizumab-doser på 2 mg/kg hver 3. uge, 10 mg/kg hver 3. uge og 10 mg/kg hver 2. uge blev vurderet i kliniske studier af melanom-patienter eller NSCLC-patienter, som tidligere har været behandlet. Baseret på modellering og simulering af forholdet mellem dosis og eksponering med hensyn til pembrolizumabs virkning og sikkerhed er der ingen klinisk signifikant forskel i virkning eller sikkerhed blandt doserne på 200 mg hver 3. uge, 2 mg/kg hver 3. uge og 400 mg hver 6. uge som monoterapi (se pkt. 4.2).

Melanom
KEYNOTE-006: Kontrolleret studie med ipilimumab-naive melanom-patienter
Pembrolizumabs sikkerhed og virkning blev undersøgt i KEYNOTE-006, et åbent, kontrolleret fase III-multicenterstudie af behandling af fremskredent melanom hos patienter, der ikke tidligere havde været behandlet med ipilimumab. Patienterne blev randomiseret i forholdet 1:1:1 til at få pembrolizumab 10 mg/kg hver 2. (n=279) eller 3. uge (n=277) eller ipilimumab 3 mg/kg hver 3. uge (n=278). Det var ikke et krav, at patienter med BRAF V600E-muteret melanom skulle have fået forudgående behandling med en BRAF-hæmmer.

Patienterne blev behandlet med pembrolizumab indtil sygdomsprogression eller uacceptabel toksicitet. Klinisk stabile patienter med initial evidens på sygdomsprogression fik lov til at forblive i behandling, indtil sygdomsprogression var blevet bekræftet. Der blev foretaget vurdering af tumorstatus i uge 12, herefter hver 6. uge til og med uge 48 og derefter hver 12. uge.

Ud af 834 patienter var 60% mænd, 44% var ≥ 65 år (medianalder 62 år [interval: 18-89]), og 98% var kaukasere. 65% af patienterne havde melanom i M1c-stadiet, 9% havde hjernemetastaser i anamnesen, 66% havde ikke tidligere fået behandling, og 34% havde forudgående fået ét behandlingsregime. 31% havde ECOG-performance-status på 1, 69% havde ECOG-performance-status på 0 og 32% havde forhøjet LDH. BRAF-mutation blev rapporteret hos 302 (36%) patienter. Blandt patienterne med BRAF-muteret tumor havde 139 (46%) tidligere været i behandling med en BRAF-hæmmer.

De primære resultatmål var progressionsfri overlevelse (PFS, baseret på IRO-vurdering [*Integrated Radiology and Oncology Assessment*] i henhold til RECIST version 1.1 [*Response Evaluation Criteria in Solid Tumors*]) og samlet overlevelse (OS). De sekundære resultatmål var objektiv responsrate (ORR) og responsvarighed. I tabel 3 opsummeres de centrale effektresultater hos ipilimumab-naive patienter efter den endelige analyse, som er blevet udført efter en opfølgningstid på mindst 21 måneder. Kaplan-Meier-kurver for OS og PFS, som er baseret på den endelige analyse, er vist i figur 1 og 2.

Tabel 3: Effektresultater i KEYNOTE-006

Endepunkt	Pembrolizumab 10 mg/kg hver 3. uge n=277	Pembrolizumab 10 mg/kg hver 2. uge n=279	Ipilimumab 3 mg/kg hver 3. uge n=278
**OS**			
Antal (%) patienter med hændelse	119 (43%)	122 (44%)	142 (51%)
*Hazard ratio** (HR) (95% konfidensinterval, CI)	0,68 (0,53; 0,86)	0,68 (0,53; 0,87)	---
p-værdi†	< 0,001	< 0,001	---
Median i måneder (95% CI)	Ikke nået (24, NA)	Ikke nået (22, NA)	16 (14, 22)
**PFS**			
Antal (%) patienter med hændelse	183 (66%)	181 (65%)	202 (73%)
HR* (95% CI)	0,61 (0,50; 0,75)	0,61 (0,50; 0,75)	---
p-værdi†	< 0,001	< 0,001	---
Median i måneder (95% CI)	4,1 (2,9; 7,2)	5,6 (3,4; 8,2)	2,8 (2,8; 2,9)
**Bedste objektive respons**			
ORR % (95% CI)	36% (30, 42)	37% (31, 43)	13% (10, 18)
Komplet respons %	13%	12%	5%
Partielt respons %	23%	25%	8%
**Responsvarighed‡**			
Median i måneder (interval)	Ikke nået (2,0; 22,8+)	Ikke nået (1,8; 22,8+)	Ikke nået (1,1+; 23,8+)
% igangværende i måned 18	68%§	71%§	70%§

* *Hazard ratio* (pembrolizumab sammenlignet med ipilimumab) baseret på den stratificerede Cox *proportional hazard*-model
† Baseret på stratificeret log rank-test
‡ Baseret på patienter med bedste objektive respons, der er bekræftet som komplet eller partielt respons
§ Baseret på Kaplan-Meier-estimering
NA = Foreligger ikke

20

Figur 1: Kaplan-Meier-kurve for samlet overlevelse i hver behandlingsarm i KEYNOTE-006 (*intent to treat*-population)

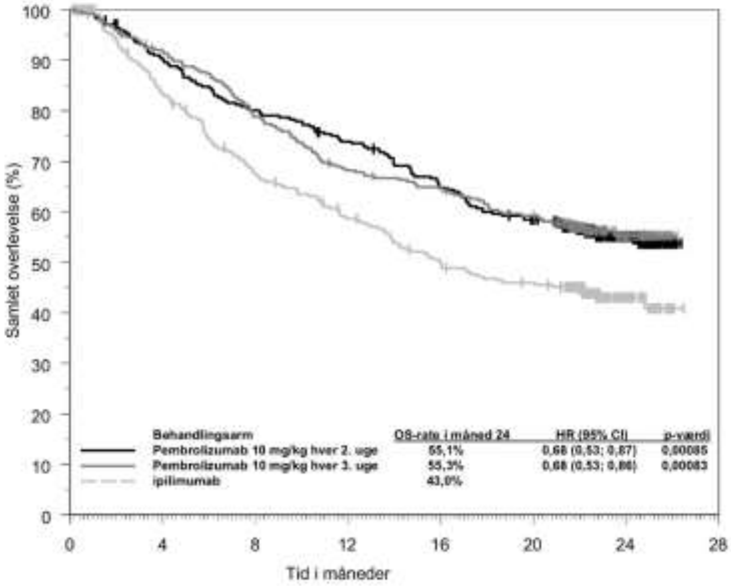

Antal i risikogruppe								
Pembrolizumab 10 mg/kg hver 2. uge:	279	249	221	202	176	156	44	0
Pembrolizumab 10 mg/kg hver 3. uge:	277	251	215	184	174	156	43	0
ipilimumab:	278	213	170	145	122	110	28	0

21

Figur 2: Kaplan-Meier-kurve for progressionsfri overlevelse i hver behandlingsarm i KEYNOTE-006 (*intent to treat*-population)

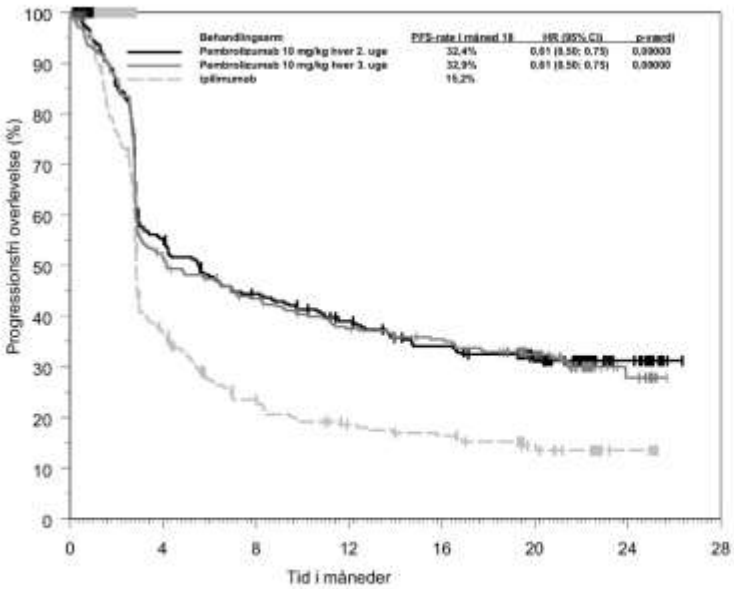

Antal i risikogruppe								
Pembrolizumab 10 mg/kg hver 2. uge:	279	148	116	98	82	52	16	0
Pembrolizumab 10 mg/kg hver 3. uge:	277	136	111	91	84	60	13	0
ipilimumab:	278	88	48	34	29	16	5	0

KEYNOTE-002: Kontrolleret studie med melanom-patienter, der tidligere har været behandlet med ipilimumab

Pembrolizumabs sikkerhed og virkning blev undersøgt i KEYNOTE-002, et dobbeltblindet, kontrolleret multicenterstudie af behandling af fremskredent melanom hos patienter, der tidligere har været behandlet med ipilimumab og med en BRAF- eller MEK-hæmmer, hvis der forekom en BRAF V600-mutation. Patienterne blev randomiseret i forholdet 1:1:1 til at få pembrolizumab i en dosis på 2 (n=180) eller 10 mg/kg (n=181) hver 3. uge eller kemoterapi (n=179; inkluderede dacarbazin, temozolomid, carboplatin, paclitaxel eller carboplatin+paclitaxel). Studiet ekskluderede patienter med autoimmun sygdom og patienter, som fik immunsuppressiv behandling. Yderligere eksklusionskriterier var en anamnese med svære eller livstruende immunrelaterede bivirkninger som følge af behandling med ipilimumab, defineret som en hvilken som helst grad 4-toksicitet eller en grad 3-toksicitet, som krævede behandling med kortikosteroider (> 10 mg prednison dagligt eller en dosis ækvivalent hermed) i mere end 12 uger; igangværende bivirkninger ≥ grad 2 fra tidligere behandling med ipilimumab; patienter, der tidligere har haft svær overfølsomhed over for andre monoklonale antistoffer; patienter med pneumonitis eller interstitiel lungesygdom i anamnesen; patienter med hiv-, hepatitis B- eller hepatitis C-infektion og ECOG-performance-status ≥ 2.

Patienterne blev behandlet med pembrolizumab indtil sygdomsprogression eller uacceptabel toksicitet. Klinisk stabile patienter med initial evidens på sygdomsprogression fik lov til at forblive i behandling, indtil sygdomsprogression var blevet bekræftet. Der blev foretaget vurdering af tumorstatus i uge 12, herefter hver 6. uge til og med uge 48 og derefter hver 12. uge. De patienter, som fik kemoterapi og oplevede uafhængigt verificeret sygdomsprogression efter den første planlagte sygdomsvurdering, kunne overkrydses til at få 2 mg/kg eller 10 mg/kg pembrolizumab hver 3. uge i et dobbeltblindet design.

Ud af 540 patienter var 61% mænd, 43% var ≥ 65 år (medianalderen var 62 år [interval: 15-89]), og 98% var kaukasere. 82% havde melanom i M1c-stadiet, 73% af patienterne havde forudgående fået mindst to, og 32% af patienterne havde fået tre eller flere systemiske behandlingsregimer for fremskredent

22

melanom. 45% havde en ECOG-performance-status på 1, 40% havde forhøjet LDH, og 23% havde en BRAF-muteret tumor.

De primære resultatmål var PFS, baseret på IRO-vurdering i henhold til RECIST version 1.1, og OS. De sekundære resultatmål var ORR og responsvarighed. I tabel 4 opsummeres de centrale effektresultater efter den endelige analyse hos patienter, der tidligere har været behandlet med ipilimumab, og Kaplan-Meier-kurven for PFS er vist i figur 3. Begge behandlingsarme med pembrolizumab havde bedre (superior) PFS i forhold til kemoterapi, og der var ingen forskel mellem pembrolizumab-doserne. Der var ingen statistisk signifikant forskel mellem pembrolizumab og kemoterapi i den endelige OS-analyse, som ikke var korrigeret for den potentielt konfunderende effekt af overkrydsningen. 55% af de patienter, som var randomiseret til kemoterapi-armen, krydsede over og fik efterfølgende behandling med pembrolizumab.

Tabel 4: Effektresultater i KEYNOTE-002

**Endepunkt**	**Pembrolizumab 2 mg/kg hver 3. uge n=180**	**Pembrolizumab 10 mg/kg hver 3. uge n=181**	**Kemoterapi n=179**
**PFS**			
Antal (%) patienter med hændelse	150 (83%)	144 (80%)	172 (96%)
HR* (95% CI)	0,58 (0,46; 0,73)	0,47 (0,37; 0,60)	---
p-værdi†	< 0,001	< 0,001	---
Median i måneder (95% CI)	2,9 (2,8; 3,8)	3,0 (2,8; 5,2)	2,8 (2,6; 2,8)
**OS**			
Antal (%) patienter med hændelse	123 (68%)	117 (65%)	128 (72%)
HR* (95% CI)	0,86 (0,67; 1,10)	0,74 (0,57; 0,96)	---
p-værdi†	0,1173	0,0106‡	---
Median i måneder (95% CI)	13,4 (11,0; 16,4)	14,7 (11,3; 19,5)	11,0 (8,9; 13,8)
**Bedste objektive respons**			
ORR % (95% CI)	22% (16; 29)	28% (21; 35)	5% (2; 9)
Komplet respons %	3%	7%	0%
Partielt respons %	19%	20%	5%
**Responsvarighed**§			
Median i måneder (interval)	22,8 (1,4+; 25,3+)	Ikke nået (1,1+; 28,3+)	6,8 (2,8; 11,3)
% igangværende i måned 12	73%¶	79%¶	0%¶

* *Hazard ratio* (pembrolizumab sammenlignet med kemoterapi) baseret på den stratificerede Cox *proportional hazard*-model
† Baseret på stratificeret log rank-test
‡ Ikke statistisk signifikant efter justering for multiplicitet
§ Baseret på patienter med bedste objektive respons, der er bekræftet som komplet eller partielt respons fra den endelige analyse
¶ Baseret på Kaplan-Meier-estimering

23

Figur 3: Kaplan-Meier-kurve for progressionsfri overlevelse i hver behandlingsarm i KEYNOTE-002 (*intent to treat*-population)

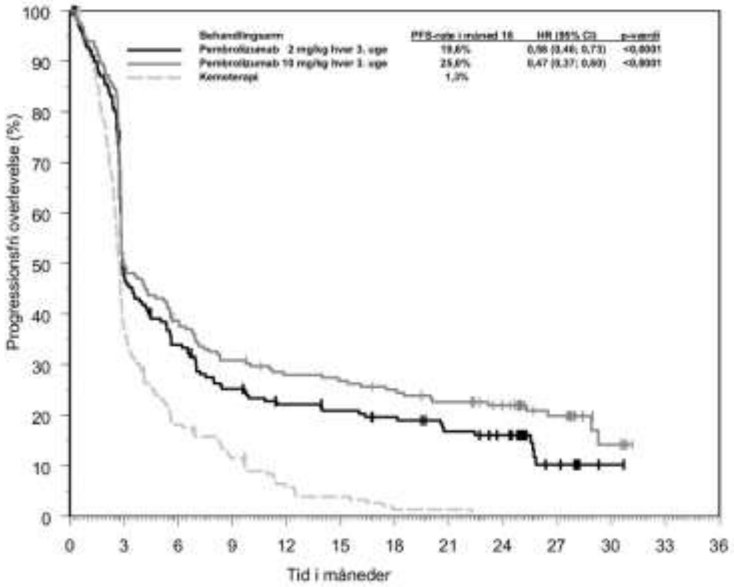

Antal i risikogruppe							
Pembrolizumab 2 mg/kg hver 3. uge:	180	59	36	29	19	1	0
Pembrolizumab 10 mg/kg hver 3. uge:	181	69	48	42	30	5	0
Kemoterapi:	179	31	9	2	1	0	0

KEYNOTE-001: Åbent studie med ipilimumab-naive og ipilimumab-erfarne melanom-patienter

Pembrolizumabs sikkerhed og virkning blev undersøgt hos patienter med fremskredent melanom i et ikke-kontrolleret, åbent studie, KEYNOTE-001. Virkningen blev vurderet hos 276 patienter fra to definerede kohorter, en kohorte, som inkluderede patienter, der tidligere har været behandlet med ipilimumab (og med en BRAF- eller MEK-hæmmer, hvis der forekom en BRAF V600-mutation), og den anden kohorte, som inkluderede patienter, der ikke tidligere har været behandlet med ipilimumab. Patienterne blev randomiseret til at få enten pembrolizumab i en dosis på 2 mg/kg hver 3. uge eller 10 mg/kg hver 3. uge. Patienterne blev behandlet med pembrolizumab indtil sygdomsprogression eller uacceptabel toksicitet. Klinisk stabile patienter med initial evidens på sygdomsprogression fik lov til at forblive i behandling, indtil sygdomsprogression var blevet bekræftet. Eksklusionskriterierne var de samme som i KEYNOTE-002.

Af de 89 patienter, som fik 2 mg/kg pembrolizumab, og som tidligere havde været behandlet med ipilimumab, var 53% mænd, 33% var ≥ 65 år, og medianalderen var 59 år (interval: 18-88). Alle på nær to patienter var kaukasere. 84% havde melanom i M1c-stadiet, og 8% af patienterne havde hjernemetastaser i anamnesen. 70% af patienterne havde forudgående fået mindst to, og 35% af patienterne havde fået tre eller flere systemiske behandlingsregimer for fremskredent melanom. BRAF-mutation blev rapporteret hos 13% af studiepopulationen. Alle patienter med BRAF-muteret tumor havde tidligere været i behandling med en BRAF-hæmmer.

Af de 51 patienter, som fik 2 mg/kg pembrolizumab, og som ikke tidligere havde været behandlet med ipilimumab, var 63% mænd, 35% var ≥ 65 år, og medianalderen var 60 år (interval: 35-80). Alle på nær en patient var kaukasere. 63% havde melanom i M1c-stadiet, og 2% af patienterne havde hjernemetastaser i anamnesen. 45% havde ikke tidligere fået behandling for fremskredent melanom. BRAF-mutation blev rapporteret hos 20 (39%) patienter. Blandt patienterne med BRAF-muteret tumor havde 10 (50%) tidligere været i behandling med en BRAF-hæmmer.

24

Det primære resultatmål var ORR, baseret på en vurdering ved IRO i henhold til RECIST 1.1. De sekundære resultatmål var sygdomskontrolrate (DCR, herunder komplet respons, partielt respons og stabil sygdom), responsvarighed, PFS og OS. Tumorrespons blev vurderet hver 12. uge. I tabel 5 opsummeres de centrale effektresultater hos patienter, der tidligere har været eller ikke tidligere har været behandlet med ipilimumab, som fik pembrolizumab ved en dosis på 2 mg/kg baseret på en opfølgningstid på mindst 30 måneder for alle patienter.

Tabel 5: Effektresultater i KEYNOTE-001

**Endepunkt**	**Pembrolizumab 2 mg/kg hver 3. uge hos ipilimumab-erfarne patienter n=89**	**Pembrolizumab 2 mg/kg hver 3. uge hos ipilimumab-naive patienter n=51**
**Bedste objektive respons* bedømt ved IRO**†		
ORR %, (95% CI)	26% (17; 36)	35% (22; 50)
Komplet respons	7%	12%
Partielt respons	19%	24%
Sygdomskontrolrate %‡	48%	49%
**Responsvarighed**§		
Median i måneder (interval)	30,5 (2,8+; 30,6+)	27,4 (1,6+; 31,8+)
% igangværende i måned 24¶	75%	71%
**PFS**		
Median i måneder (95% CI)	4,9 (2,8; 8,3)	4,7 (2,8; 13,8)
PFS-rate i måned 12	34%	38%
**OS**		
Median i måneder (95% CI)	18,9 (11, foreligger ikke)	28,0 (14, foreligger ikke)
OS-rate i måned 24	44%	56%

* Inkluderer patienter uden målbar sygdom ved *baseline* ved uafhængig radiologisk gennemgang
† IRO = *Integrated Radiology and Oncology* vurdering i henhold til RECIST 1.1
‡ Baseret på stabil sygdom eller bedre som bedste respons
§ Baseret på patienter med bekræftet respons ved uafhængig gennemgang, startende fra den dato, hvor respons blev registreret første gang; n=23 for ipilimumab-erfarne patienter; n=18 for ipilimumab-naive patienter
¶ Baseret på Kaplan-Meier-estimering

Resultaterne for ipilimumab-erfarne patienter (n=84) og for ipilimumab-naive patienter (n=52), som fik 10 mg/kg pembrolizumab hver 3. uge, var sammenlignelige med resultaterne for patienter, som fik 2 mg/kg pembrolizumab hver 3. uge.

Analyser af subgrupper

BRAF-mutationstatus ved melanom
Der blev udført en subgruppeanalyse som en del af den endelige analyse i KEYNOTE-002 hos patienter, som havde BRAF vildtype (n=414; 77%) eller BRAF-mutation med forudgående BRAF-behandling (n=126; 23%) som opsummeret i tabel 6.

25

Tabel 6: Effektresultater ud fra BRAF-mutationsstatus i KEYNOTE-002

	BRAF vildtype		BRAF-mutation med forudgående BRAF-behandling	
**Endepunkt**	**Pembrolizumab 2 mg/kg hver 3. uge (n=136)**	**Kemoterapi (n=137)**	**Pembrolizumab 2 mg/kg hver 3. uge (n=44)**	**Kemoterapi (n=42)**
HR* for PFS (95% CI)	0,50 (0,39; 0,66)	---	0,79 (0,50; 1,25)	---
HR* for OS (95% CI)	0,78 (0,58; 1,04)	---	1,07 (0,64; 1,78)	---
ORR %	26%	6%	9%	0%

* *Hazard ratio* (pembrolizumab sammenlignet med kemoterapi) baseret på den stratificerede Cox *proportional hazard*-model

Der blev udført en subgruppeanalyse som en del af den endelige analyse i KEYNOTE-006 hos patienter, som var enten BRAF vildtype (n=525; 63%), BRAF-mutation uden forudgående BRAF-behandling (n=163; 20%) eller BRAF-mutation med forudgående BRAF-behandling (n=139; 17%) som opsummeret i tabel 7.

Tabel 7: Effektresultater ud fra BRAF-mutationsstatus i KEYNOTE-006

	BRAF vildtype		BRAF-mutation uden forudgående BRAF-behandling		BRAF-mutation med forudgående BRAF-behandling	
**Endepunkt**	**Pembrolizumab 10 mg/kg hver 2. eller 3. uge (puljet)**	**Ipilimumab (n=170)**	**Pembrolizumab 10 mg/kg hver 2. eller 3. uge (puljet)**	**Ipilimumab (n=55)**	**Pembrolizumab 10 mg/kg hver 2. eller 3. uge (puljet)**	**Ipilimumab (n=52)**
HR* for PFS (95% CI)	0,61 (0,49; 0,76)	---	0,52 (0,35; 0,78)	---	0,76 (0,51; 1,14)	---
HR* for OS (95% CI)	0,68 (0,52; 0,88)	---	0,70 (0,40; 1,22)	---	0,66 (0,41; 1,04)	---
ORR %	38%	14%	41%	15%	24%	10%

* *Hazard ratio* (pembrolizumab sammenlignet med ipilimumab) baseret på den stratificerede Cox *proportional hazard*-model

PD-L1-status ved melanom

Der blev udført en subgruppeanalyse som en del af den endelige analyse i KEYNOTE-002 hos patienter, der var PD-L1-positive (PD-L1-ekspression i ≥ 1% af tumorcellerne og tumor-associerede immunceller i forhold til alle levedygtige tumorceller – MEL-score) *versus* PD-L1-negative. PD-L1-ekspression blev testet retrospektivt ved en immunhistokemisk analyse med 22C3 anti-PD-L1-antistof. Blandt de patienter, som var evaluerbare for PD-L1-ekspression (79%), var 69% (n=294) PD-L1-positive, og 31% (n=134) var PD-L1-negative. I tabel 8 opsummeres effektresultaterne ud fra PD-L1-ekspression.

Tabel 8: Effektresultater ud fra PD-L1-ekspression i KEYNOTE-002

**Endepunkt**	**Pembrolizumab 2 mg/kg hver 3. uge**	**Kemoterapi**	**Pembrolizumab 2 mg/kg hver 3. uge**	**Kemoterapi**
	**PD-L1-positiv**		**PD-L1-negativ**	
HR* for PFS (95% CI)	0,55 (0,40; 0,76)	---	0,81 (0,50; 1,31)	---
HR* for OS (95% CI)	0,90 (0,63; 1,28)	---	1,18 (0,70; 1,99)	---
ORR %	25%	4%	10%	8%

* *Hazard ratio* (pembrolizumab sammenlignet med kemoterapi) baseret på den stratificerede Cox *proportional hazard*-model

Der blev udført en subgruppeanalyse som en del af den endelige analyse i KEYNOTE-006 hos patienter, der var PD-L1-positive (n=671; 80%) *versus* PD-L1-negative (n=150; 18%). Hos de patienter, som var evaluerbare for PD-L1-ekspression (98%), var 82% PD-L1-positive, og 18% var PD-L1-negative. I tabel 9 opsummeres effektresultaterne ud fra PD-L1-ekspression.

Tabel 9: Effektresultater ud fra PD-L1-ekspression i KEYNOTE-006

Endepunkt	Pembrolizumab 10 mg/kg hver 2. eller 3. uge (puljet)	Ipilimumab	Pembrolizumab 10 mg/kg hver 2. eller 3. uge (puljet)	Ipilimumab
	PD-L1-positiv		PD-L1-negativ	
HR* for PFS (95% CI)	0,53 (0,44; 0,65)	---	0,87 (0,58; 1,30)	---
HR* for OS (95% CI)	0,63 (0,50; 0,80)	---	0,76 (0,48; 1,19)	---
ORR %	40%	14%	24%	13%

* *Hazard ratio* (pembrolizumab sammenlignet med ipilimumab) baseret på den stratificerede Cox *proportional hazard*-model

Okulært melanom
Hos 20 forsøgspersoner med okulært melanom, som var inkluderet i KEYNOTE-001, blev der ikke rapporteret objektivt respons. Stabil sygdom blev rapporteret hos 6 patienter.

KEYNOTE-054: Placebokontrolleret studie med adjuverende behandling af patienter med komplet resekteret melanom
Pembrolizumabs virkning blev vurderet i KEYNOTE-054, et randomiseret, dobbeltblindet, placebokontrolleret multicenterstudie hos patienter med komplet resekteret stadie IIIA- (> 1 mm lymfeknudemetastaser), IIIB- eller IIIC-melanom. I alt 1.019 voksne patienter blev randomiseret i forholdet 1:1 til at få pembrolizumab 200 mg hver 3. uge (n=514) eller placebo (n=505) i en periode på op til 1 år indtil sygdomsrecidiv eller uacceptabel toksicitet. Randomiseringen blev stratificeret ud fra stadie (IIIA *versus* IIIB *versus* IIIC 1-3 positive lymfeknuder *versus* IIIC ≥ 4 positive lymfeknuder) i henhold til *American Joint Committee on Cancer* (AJCC), 7. udgave, og geografisk område (Nordamerika, de europæiske lande, Australien og andre udvalgte lande). Patienterne skal have fået foretaget lymfeknude-dissektion, og hvis det er indiceret, strålebehandling inden for 13 uger før påbegyndelse af behandlingen. Patienter med aktiv autoimmun sygdom eller lidelser, som krævede behandling med immunsuppressiva, eller slimhindemelanom eller okulært melanom var uegnede til studiet. Patienter, som havde modtaget anden forudgående behandling for melanom end kirurgisk indgreb eller interferon for tykke primære melanomer uden tegn på lymfeknudeinvolvering, var uegnede til studiet. Patienterne fik foretaget billeddiagnostisk undersøgelse hver 12. uge efter den første dosis pembrolizumab i løbet af de første 2 år, herefter hver 6. måned fra år 3 til 5 og herefter årligt.

Hos de 1.019 patienter var *baseline*-karakteristika: medianalder 54 år (25% i alderen 65 år eller ældre); 62% mænd og ECOG-performance-status 0 (94%) og 1 (6%). 16% havde stadie IIIA; 46% havde stadie IIIB; 18% havde stadie IIIC (1-3 positive lymfeknuder), og 20% havde stadie IIIC (≥ 4 positive lymfeknuder); 50% var positive for BRAF V600-mutation og 44% var BRAF vildtype. PD-L1-ekspression blev testet retrospektivt ved en immunhistokemisk analyse med 22C3 anti-PD-L1-antistof; 84% af patienterne havde PD-L1-positivt melanom (PD-L1-ekspression hos ≥ 1% af tumor- og tumorassocierede immunceller i forhold til alle levedygtige tumorceller). Der blev anvendt det samme scoringssystem ved metastatisk melanom (MEL-score).

Det primære resultatmål var investigator-vurderet recidivfri overlevelse (RFS) i hele populationen og i populationen med PD-L1-positive tumorer, hvor RFS blev defineret som tiden mellem datoen for randomisering og datoen for første recidiv (lokale, regionale eller fjernmetastaser) eller død, afhængigt af, hvad der indtræffer først. Studiet viste en statistisk signifikant forbedring i RFS for patienter randomiseret til armen med pembrolizumab sammenlignet med placebo ved den præspecificerede interimanalyse. Effektresultater baseret på yderligere 7 måneders opfølgning er opsummeret i tabel 10 og i figur 4.

Tabel 10: Effektresultater i KEYNOTE-054

Endepunkt	KEYTRUDA 200 mg hver 3. uge n=514	Placebo n=505
Antal (%) patienter med hændelse	158 (31%)	246 (49%)
Median i måneder (95% CI)	NR	21,7 (17,1; NR)
HR* (98% CI)	0,56 (0,44; 0,72)	
p-værdi (stratificeret log-rank)	< 0,0001	
**RFS ved måned 6**		
RFS-rate	82%	73%
**RFS ved måned 12**		
RFS-rate	76%	61%
**RFS ved måned 18**		
RFS-rate	72%	54%

* Baseret på den stratificerede Cox *proportional hazard*-model
NR = Ikke nået

KEYNOTE-054 inkluderede patienter i henhold til AJCC, 7. udgave, og en subgruppeanalyse af RFS i henhold til AJCC, 8. udgave, blev udført efter at RFS-studieresultaterne blev rapporteret. Der blev påvist en statistisk signifikant forbedring i RFS for patienter randomiseret til armen med pembrolizumab sammenlignet med placebo i den samlede population på tværs af resekteret stadie III-melanom i henhold til AJCC, 7. udgave. Stadie IIIA-melanom i henhold til AJCC, 8. udgave, identificerer en patientpopulation med en bedre prognose sammenlignet med stadie IIIA i henhold til AJCC, 7. udgave. I henhold til AJCC-stadieklassificering, 8. udgave, blev i alt 82 forsøgspersoner klassificeret som stadie IIIA; 42 i armen med pembrolizumab og 40 i placebo-armen; med i alt 13 RFS-hændelser; 6 i armen med pembrolizumab og 7 i placebo-armen. Der er begrænsede data vedrørende forsøgspersoner med stadie IIIA i henhold til AJCC, 8. udgave, på tidspunktet for RFS-analysen.

28

Figur 4: Kaplan-Meier-kurve for recidivfri overlevelse i hver behandlingsarm i KEYNOTE-054 (*intent to treat*-population)

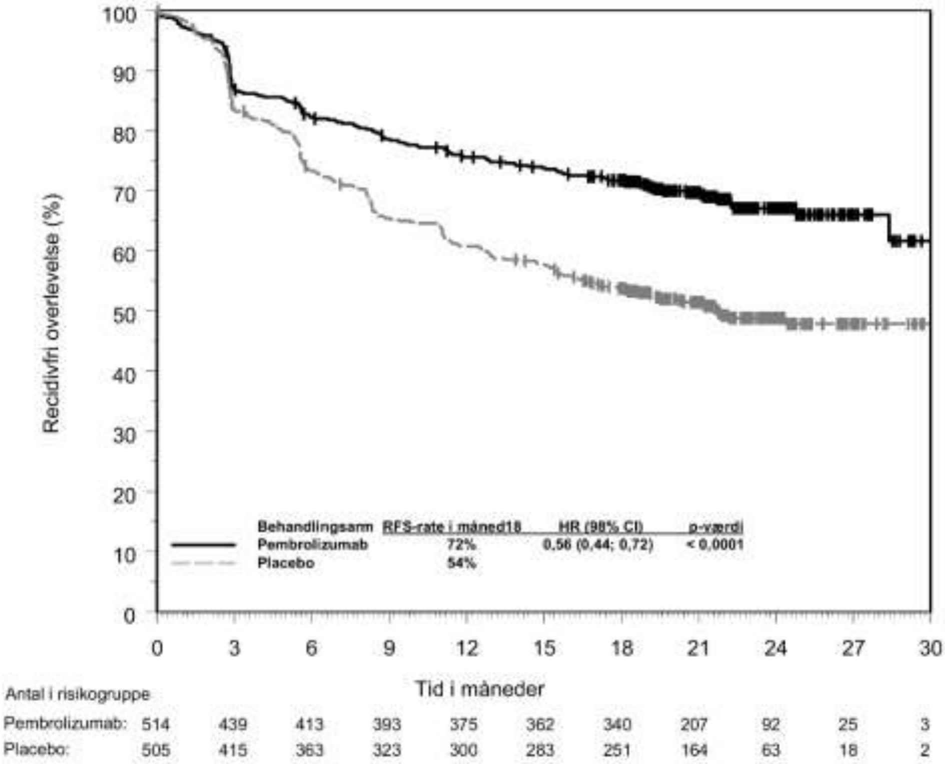

Selvom analysen hos patienter med PD-L1-positive tumorer var et co-primært endepunkt, blev der udført prædefinerede subgruppeanalyser hos patienter, hvis tumorer var PD-L1-negative, positive eller negative for BRAF-mutation. I tabel 11 opsummeres effektresultater ud fra PD-L1-ekspression og BRAF-mutationsstatus.

Tabel 11: Effektresultater ud fra PD-L1-ekspression og BRAF-mutationsstatus i KEYNOTE-054

Endepunkt	Pembrolizumab 200 mg hver 3. uge	Placebo	Pembrolizumab 200 mg hver 3. uge	Placebo
	PD-L1-positiv		PD-L1-negativ	
	n=428	n=425	n=59	n=57
HR* for RFS (95% CI)	0,54 (0,42; 0,69)	---	0,47 (0,26; 0,85)	---
RFS-rate ved måned 6	84%	75%	81%	64%
	Positiv for BRAF-mutation		Negativ for BRAF-mutation	
	n=245	n=262	n=233	n=214
HR* for RFS (95% CI)	0,49 (0,36; 0,67)	---	0,64 (0,47; 0,87)	---
RFS-rate ved måned 6	83%	73%	80%	72%

*Baseret på den stratificerede Cox *proportional hazard*-model

29

NSCLC

KEYNOTE-024: Kontrolleret studie med behandlingsnaive NSCLC-patienter

Pembrolizumabs sikkerhed og virkning blev undersøgt i KEYNOTE-024, et åbent, kontrolleret multicenterstudie hos tidligere ubehandlede patienter med metastatisk NSCLC. Patienterne havde PD-L1-ekspression med TPS ≥ 50% baseret på PD-L1 IHC 22C3 pharmDx™-analysen. Patienterne blev randomiseret i forholdet 1:1 til pembrolizumab i en dosis på 200 mg hver 3. uge (n=154) eller investigators valg af platinbaseret kemoterapi (inklusive pemetrexed+carboplatin, pemetrexed+cisplatin, gemcitabin+cisplatin, gemcitabin+carboplatin eller paclitaxel+carboplatin; n=151. Patienter med ikke-planocellulært karcinom kunne få vedligeholdelsesbehandling med pemetrexed). Patienterne blev behandlet med pembrolizumab indtil uacceptabel toksicitet eller sygdomsprogression. Behandlingen kunne fortsætte efter sygdomsprogression, hvis patienten var klinisk stabil, og investigator mente, at patienten havde en klinisk fordel. Patienter uden sygdomsprogression kunne behandles i op til 24 måneder. Patienter med EGFR- eller ALK-mutationer i tumor, autoimmun sygdom, der havde krævet systemisk behandling inden for de seneste 2 år, lidelser, som krævede behandling med immunsuppressiva, og patienter, som havde fået mere end 30 Gy torakal strålebehandling inden for de forudgående 26 uger, blev ekskluderet fra studiet. Tumorstatus blev vurderet hver 9. uge. Patienter i behandling med kemoterapi, som oplevede uafhængigt verificeret sygdomsprogression, kunne krydse over til pembrolizumab.

Hos de 305 patienter i KEYNOTE-024 var *baseline*-karakteristika: medianalder 65 år (54% ≥ 65 år), 61% mænd, 82% kaukasere, 15% asiater og ECOG-performance-status 0 og 1 hos henholdsvis 35% og 65%. Sygdomskarakteristika var planocellulær (18%) og ikke-planocellulær (82%), M1-stadie (99%) og hjernemetastaser (9%).

Det primære resultatmål var PFS baseret på BICR (*blinded independent central review*)-vurdering i henhold til RECIST 1.1. Sekundære resultatmål var OS og ORR (baseret på BICR-vurdering i henhold til RECIST 1.1). I tabel 12 opsummeres de vigtigste effektresultater for hele *intent to treat* (ITT)-populationen. PFS- og ORR-resultater er rapporteret fra en interimanalyse med en median opfølgningstid på 11 måneder. OS-resultater er rapporteret fra den endelige analyse med en median opfølgningstid på 25 måneder.

Tabel 12: Effektresultater i KEYNOTE-024

Endepunkt	Pembrolizumab 200 mg hver 3. uge n=154	Kemoterapi n=151
**PFS**		
Antal (%) patienter med hændelse	73 (47%)	116 (77%)
HR* (95% CI)	0,50 (0,37; 0,68)	
p-værdi[†]	< 0,001	
Median i måneder (95% CI)	10,3 (6,7; NA)	6,0 (4,2; 6,2)
**OS**		
Antal (%) patienter med hændelse	73 (47%)	96 (64%)
HR* (95% CI)	0,63 (0,47; 0,86)	
p-værdi[†]	0,002	
Median i måneder (95% CI)	30,0 (18,3; NA)	14,2 (9,8; 19,0)
**Objektiv responsrate**		
ORR % (95% CI)	45% (37; 53)	28% (21; 36)
Komplet respons %	4%	1%
Partielt respons %	41%	27%
**Responsvarighed**[‡]		
Median i måneder (interval)	Ikke nået (1,9+; 14,5+)	6,3 (2,1+; 12,6+)
% med varighed ≥ 6 måneder	88%[§]	59%[¶]

* *Hazard ratio* (pembrolizumab sammenlignet med kemoterapi) baseret på den stratificerede Cox *proportional hazard*-model

† Baseret på stratificeret log rank-test

‡ Baseret på patienter med bedste objektive respons, der er bekræftet komplet respons eller partielt respons

§ Baseret på Kaplan-Meier-estimater; inkluderer 43 patienter med respons på 6 måneder eller længere

¶ Baseret på Kaplan-Meier-estimater; inkluderer 16 patienter med respons på 6 måneder eller længere

NA = Foreligger ikke

31

Figur 5: Kaplan-Meier-kurve for progressionsfri overlevelse i hver behandlingsarm i KEYNOTE-024 (*intent to treat*-population)

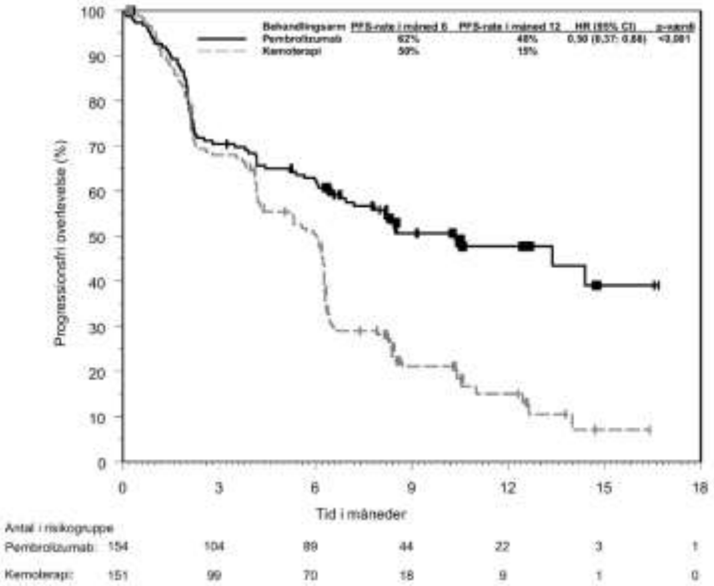

Figur 6: Kaplan-Meier-kurve for samlet overlevelse i hver behandlingsarm i KEYNOTE-024 (*intent to treat*-population)

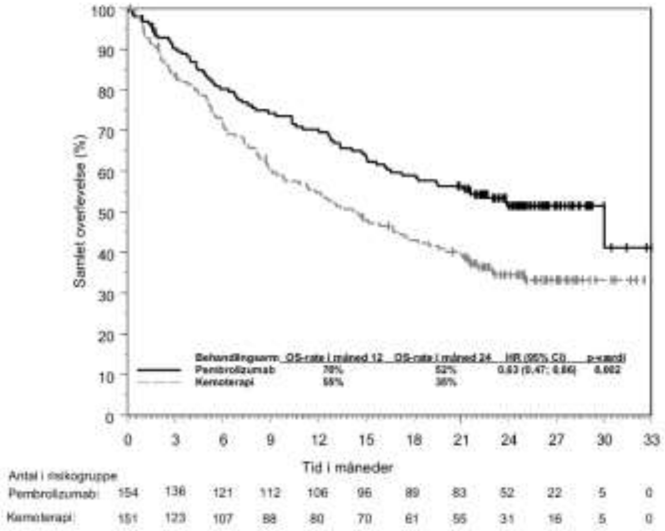

I en subgruppeanalyse viste pembrolizumab en reduceret fordel med hensyn til overlevelse sammenlignet med kemoterapi hos de få patienter, som aldrig havde røget. På grund af det lave antal patienter kan der dog ikke drages nogle definitive konklusioner på baggrund af disse data.

KEYNOTE-189: Kontrolleret studie af kombinationsbehandling hos behandlingsnaive patienter med ikke-planocellulær NSCLC

Pembrolizumabs virkning i kombination med pemetrexed og platinbaseret kemoterapi blev undersøgt i et randomiseret, aktivt kontrolleret, dobbeltblindet multicenterstudie, KEYNOTE-189. De centrale inklusionskriterier var metastatisk ikke-planocellulær NSCLC, ingen tidligere systemisk behandling for metastatisk NSCLC og ingen EGFR- eller ALK-mutationer i tumor. Patienter med autoimmun sygdom, der havde krævet systemisk behandling inden for de seneste 2 år; lidelser, som krævede behandling med immunsuppressiva; eller patienter, som havde fået mere end 30 Gy torakal strålebehandling inden for de forudgående 26 uger, blev ekskluderet fra studiet. Patienterne blev randomiseret i forholdet 2:1 til behandling med et af følgende regimer:

- Pembrolizumab 200 mg med pemetrexed 500 mg/m^2 og investigators valg af cisplatin 75 mg/m^2 eller carboplatin AUC 5 mg/ml/min intravenøst hver 3. uge i 4 serier efterfulgt af pembrolizumab 200 mg og pemetrexed 500 mg/m^2 intravenøst hver 3. uge (n=410).
- Placebo med pemetrexed 500 mg/m^2 og investigators valg af cisplatin 75 mg/m^2 eller carboplatin AUC 5 mg/ml/min intravenøst hver 3. uge i 4 serier efterfulgt af placebo og pemetrexed 500 mg/m^2 intravenøst hver 3. uge (n=206).

Behandling med pembrolizumab fortsatte indtil sygdomsprogression defineret i henhold til RECIST 1.1 efter investigators vurdering, uacceptabel toksicitet eller i højst 24 måneder. Administration af pembrolizumab var tilladt efter sygdomsprogression i henhold til RECIST baseret på BICR-vurdering eller efter seponering af pemetrexed, hvis patienten var klinisk stabil, og investigator vurderede, at patienten havde en klinisk fordel. Hos patienter, der gennemførte 24 måneders behandling eller som havde komplet respons, kunne behandlingen med pembrolizumab genoptages ved sygdomsprogression og blive administreret i op til yderligere 1 år. Vurdering af tumorstatus blev foretaget i uge 6 og uge 12 og efterfølgende hver 9. uge derefter. Patienter, der fik placebo plus kemoterapi, som oplevede uafhængigt verificeret sygdomsprogression, blev tilbudt pembrolizumab som monoterapi.

Hos de 616 patienter i KEYNOTE-189 var *baseline*-karakteristika: medianalder 64 år (49% i alderen 65 år eller ældre); 59% mænd; 94% kaukasere og 3% asiater; henholdsvis 43% og 56% med ECOG-performance-status 0 eller 1; 31% PD-L1-negative (TPS < 1%); og 18% med behandlede eller ikke behandlede hjernemetastaser ved *baseline*. I alt 67 patienter i placebo plus kemoterapiarmen krydsede over til pembrolizumab som monoterapi på tidspunktet for sygdomsprogression, og yderligere 18 patienter fik en *checkpoint*-hæmmer som efterfølgende behandling.

De primære resultatmål var OS og PFS (BICR-vurdering i henhold til RECIST 1.1). De sekundære resultatmål var ORR og responsvarighed baseret på BICR-vurdering i henhold til RECIST 1.1. Den mediane opfølgningstid var 10,5 måneder (interval: 0,2 til 20,4 måneder). I tabel 13 opsummeres de vigtigste effektresultater. Kaplan-Meier-kurver for OS og PFS er vist i figur 7 og 8.

Tabel 13: Effektresultater i KEYNOTE-189

Endepunkt	Pembrolizumab + pemetrexed + platinbaseret kemoterapi n=410	Placebo + pemetrexed + platinbaseret kemoterapi n=206
**OS**		
Antal (%) patienter med hændelse	127 (31%)	108 (52%)
HR* (95% CI)	0,49 (0,38; 0,64)	
p-værdi†	< 0,00001	
Median i måneder (95% CI)	Ikke nået (NA; NA)	11,3 (8,7; 15,1)
**PFS**		
Antal (%) patienter med hændelse	244 (60%)	166 (81%)
HR* (95% CI)	0,52 (0,43; 0,64)	
p-værdi†	< 0,00001	
Median i måneder (95% CI)	8,8 (7,6; 9,2)	4,9 (4,7; 5,5)
**Objektiv responsrate**		
ORR‡ % (95% CI)	48% (43; 53)	19% (14; 25)
Komplet respons %	0,5%	0,5%
Partielt respons %	47%	18%
p-værdi§	< 0,0001	
**Responsvarighed**		
Median i måneder (interval)	11,2 (1,1+; 18,0+)	7,8 (2,1+; 16,4+)
% med varighed ≥ 6 måneder¶	81%	63%
% med varighed ≥ 9 måneder¶	60%	44%

* Baseret på den stratificerede Cox *proportional hazard*-model
† Baseret på stratificeret log rank-test
‡ Baseret på patienter med bedste objektive respons, der er bekræftet komplet respons eller partielt respons
§ Baseret på Miettinen- og Nurminen-metoden stratificeret ud fra PD-L1-status, platinbaseret kemoterapi og rygestatus
¶ Baseret på Kaplan-Meier-estimering
NA = Foreligger ikke

34

Figur 7: Kaplan-Meier-kurve for samlet overlevelse i hver behandlingsarm i KEYNOTE-189 (*intent to treat*-population)

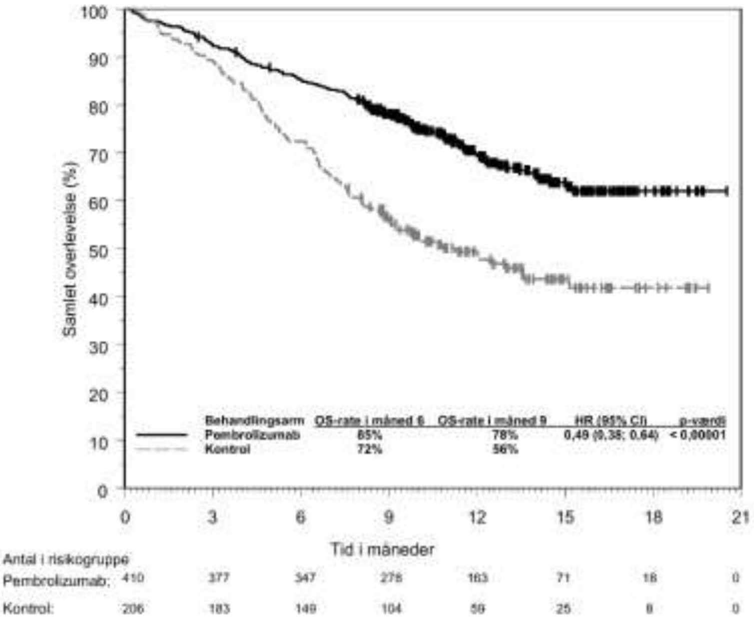

Figur 8: Kaplan-Meier-kurve for progressionsfri overlevelse i hver behandlingsarm i KEYNOTE-189 (*intent to treat*-population)

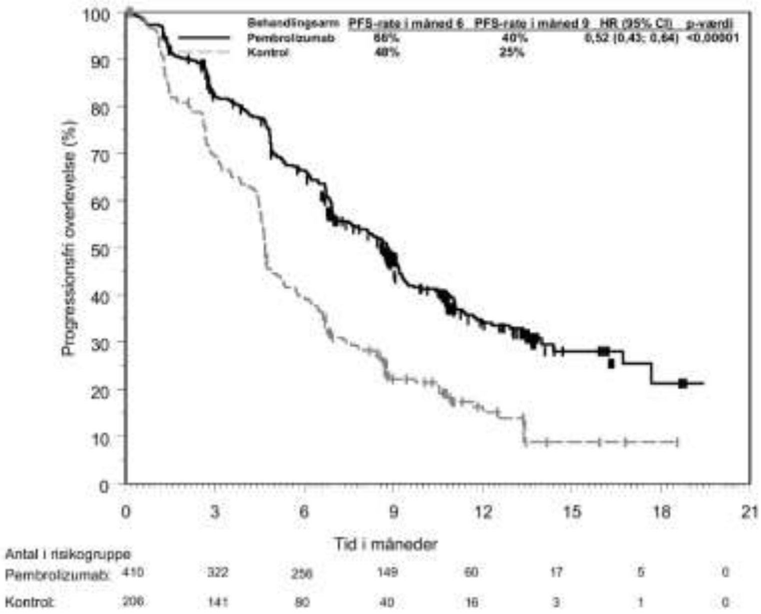

35

Der blev udført en analyse i KEYNOTE-189 hos patienter, som havde PD-L1 TPS < 1% [pembrolizumab-kombination: n=127 (31%) *versus* kemoterapi: n=63 (31%)], TPS 1-49% [pembrolizumab-kombination: n=128 (31%) *versus* kemoterapi: n=58 (28%)] eller TPS ≥ 50% [pembrolizumab-kombination: n=132 (32%) *versus* kemoterapi: n=70 (34%)] (se tabel 14).

Tabel 14: Effektresultater ud fra PD-L1-ekspression i KEYNOTE-189

Endepunkt	Pembrolizumab kombinations-behandling	Kemoterapi	Pembrolizumab kombinations-behandling	Kemoterapi	Pembrolizumab kombinations-behandling	Kemoterapi
	TPS < 1%		TPS 1 til 49%		TPS ≥ 50%	
HR* for OS (95% CI)	0,59 (0,38; 0,92)		0,55 (0,34; 0,90)		0,42 (0,26; 0,68)	
HR* for PFS (95% CI)	0,75 (0,53; 1,05)		0,55 (0,37; 0,81)		0,36 (0,25; 0,52)	
ORR %	32%	14%	48%	21%	61%	23%

* *Hazard ratio* (pembrolizumab kombinationsbehandling sammenlignet med kemoterapi) baseret på den stratificerede Cox *proportional hazard*-model

I alt 57 NSCLC-patienter ≥ 75 år var inkluderet i KEYNOTE-189-studiet (35 i kombinationsbehandlingen med pembrolizumab og 22 i kontrolgruppen). En HR = 2,09 [95% CI 0,84; 5,23] for OS og HR = 1,73 [95% CI 0,77; 3,90] for PFS for kombinationsbehandlingen med pembrolizumab *versus* kemoterapi blev rapporteret i denne studiesubgruppe. Data vedrørende pembrolizumabs sikkerhed og virkning i kombination med platinbaseret kemoterapi er begrænset i denne patientpopulation.

KEYNOTE-407: Kontrolleret studie af kombinationsbehandling hos behandlingsnaive patienter med planocellulær NSCLC

Pembrolizumabs virkning i kombination med carboplatin og enten paclitaxel eller nab-paclitaxel blev undersøgt i KEYNOTE-407, et randomiseret, dobbeltblindet, placebokontrolleret multicenterstudie. De centrale inklusionskriterier var metastatisk planocellulær NSCLC, uanset PD-L1-tumorekspressionsstatus, og ingen tidligere systemisk behandling for metastatisk sygdom. Patienter med autoimmun sygdom, der havde krævet systemisk behandling inden for de seneste 2 år; lidelser, som krævede behandling med immunsuppressiva; eller patienter, som havde fået mere end 30 Gy torakal strålebehandling inden for de forudgående 26 uger, blev ekskluderet fra studiet. Randomiseringen blev stratificeret ud fra PD-L1-tumorekspression (TPS < 1% [negativ] *versus* TPS ≥ 1%), investigators valg af paclitaxel eller nab-paclitaxel og geografisk område (Østasien *versus* ikke Østasien). Patienterne blev randomiseret i forholdet 1:1 til en af følgende behandlingsarme med intravenøs infusion:

- Pembrolizumab 200 mg og carboplatin AUC 6 mg/ml/min på dag 1 i hver 21-dages serie i 4 serier, og paclitaxel 200 mg/m^2 på dag 1 i hver 21-dages serie i 4 serier eller nab-paclitaxel 100 mg/m^2 på dag 1, 8 og 15 i hver 21-dages serie i 4 serier, efterfulgt af pembrolizumab 200 mg hver 3. uge. Pembrolizumab blev administreret før kemoterapi på dag 1.
- Placebo og carboplatin AUC 6 mg/ml/min på dag 1 i hver 21-dages serie i 4 serier og paclitaxel 200 mg/m^2 på dag 1 i hver 21-dages serie i 4 serier eller nab-paclitaxel 100 mg/m^2 på dag 1, 8 og 15 i hver 21-dages serie i 4 serier, efterfulgt af placebo hver 3. uge.

Behandling med pembrolizumab eller placebo fortsatte indtil sygdomsprogression i henhold til RECIST 1.1 baseret på BICR-vurdering, uacceptabel toksicitet eller i højst 24 måneder. Administration af pembrolizumab var tilladt efter sygdomsprogression i henhold til RECIST, hvis patienten var klinisk stabil, og investigator vurderede, at patienten havde en klinisk fordel.

Patienterne i placeboarmen blev tilbudt pembrolizumab som monoterapi på tidspunktet for sygdomsprogression.

Vurdering af tumorstatus blev foretaget hver 6. uge til og med uge 18, hver 9. uge til og med uge 45 og hver 12. uge derefter.

I alt 559 patienter blev randomiseret. Karakteristika for studiepopulationen var: medianalder på 65 år (interval: 29 til 88); 55% i alderen 65 år eller ældre; 81% mænd; 77% kaukasere; ECOG-performance-

status 0 (29%) og 1 (71%); og 8% med behandlede hjernemetastaser ved *baseline*. 35% havde PD-L1-tumorekspression TPS < 1% [negativ]; 19% var østasiatere og 60% fik paclitaxel.

De primære resultatmål var OS og PFS (BICR-vurdering i henhold til RECIST 1.1). De sekundære resultatmål var ORR og responsvarighed baseret på BICR-vurdering i henhold til RECIST 1.1. Den mediane opfølgningstid var 7,8 måneder (interval: 0,1 til 19,1 måneder). I tabel 15 opsummeres de vigtigste effektresultater. Kaplan-Meier-kurver for OS og PFS er vist i figur 9 og 10.

Tabel 15: Effektresultater i KEYNOTE-407

Endepunkt	Pembrolizumab Carboplatin Paclitaxel/nab-paclitaxel n=278	Placebo Carboplatin Paclitaxel/nab-paclitaxel n=281
**OS**		
Antal hændelser (%)	85 (31%)	120 (43%)
Median i måneder (95% CI)	15,9 (13,2; NA)	11,3 (9,5; 14,8)
HR* (95% CI)	0,64 (0,49; 0,85)	
p-værdi†	0,0008	
**PFS**		
Antal hændelser (%)	152 (55%)	197 (70%)
Median i måneder (95% CI)	6,4 (6,2; 8,3)	4,8 (4,3; 5,7)
HR* (95% CI)	0,56 (0,45; 0,70)	
p-værdi†	< 0,0001	
**Objektiv responsrate**		
ORR (95% CI)	58% (52; 64)	38% (33; 44)
Komplet respons %	1,4%	2,1%
Partielt respons %	57%	36%
p-værdi‡	< 0,0001	
**Responsvarighed**		
Median responsvarighed i måneder (interval)	7,7 (1,1+; 14,7+)	4,8 (1,3+; 15,8+)
% med varighed ≥ 6 måneder§	62%	40%

* Baseret på den stratificerede Cox *proportional hazard*-model
† Baseret på stratificeret log rank-test
‡ Baseret på Miettinen- og Nurminen-metoden
§ Baseret på Kaplan-Meier-estimering
NA = Foreligger ikke

37

Figur 9: Kaplan-Meier-kurve for samlet overlevelse i KEYNOTE-407

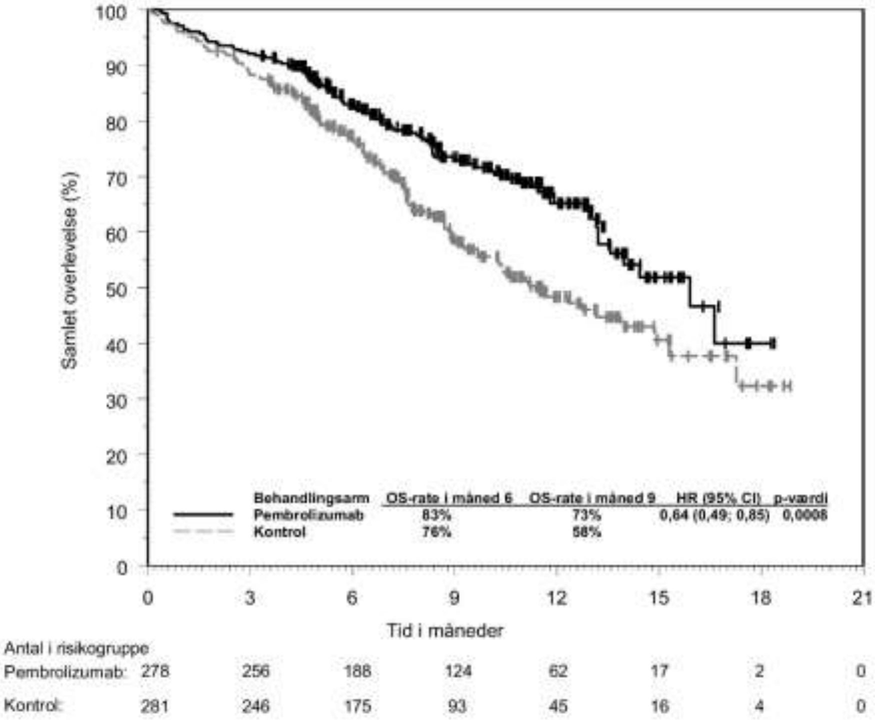

38

Figur 10: Kaplan-Meier-kurve for progressionsfri overlevelse i KEYNOTE-407

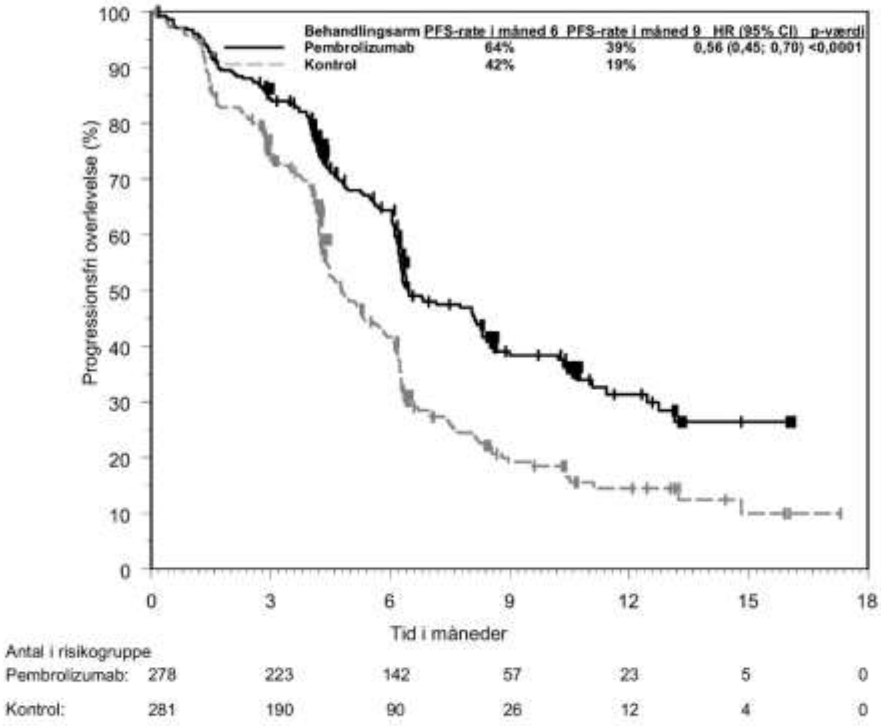

Der blev udført en analyse i KEYNOTE-407 hos patienter, som havde PD-L1 TPS < 1% [pembrolizumab plus kemoterapiarmen: n=95 (34%) *versus* placebo plus kemoterapiarmen: n=99 (35%)], TPS 1% til 49% [pembrolizumab plus kemoterapiarmen: n=103 (37%) *versus* placebo plus kemoterapiarmen: n=104 (37%)] eller TPS ≥ 50% [pembrolizumab plus kemoterapiarmen: n=73 (26%) *versus* placebo plus kemoterapiarmen: n=73 (26%)] (se tabel 16).

Tabel 16: Effektresultater ud fra PD-L1-ekspression i KEYNOTE-407

Endepunkt	Pembrolizumab kombinations-behandling	Kemoterapi	Pembrolizumab kombinations-behandling	Kemoterapi	Pembrolizumab kombinations-behandling	Kemoterapi
	TPS < 1%		TPS 1 til 49%		TPS ≥ 50%	
HR* for OS (95% CI)	0,61 (0,38; 0,98)		0,57 (0,36; 0,90)		0,64 (0,37; 1,10)	
HR* for PFS (95% CI)	0,68 (0,47; 0,98)		0,56 (0,39; 0,80)		0,37 (0,24; 0,58)	
ORR %	63%	40%	50%	41%	60%	33%

* *Hazard ratio* (pembrolizumab kombinationsbehandling sammenlignet med kemoterapi) baseret på den stratificerede Cox *proportional hazard*-model

I alt 65 NSCLC-patienter i alderen ≥ 75 år blev inkluderet i KEYNOTE-407-studiet (34 i kombinationsbehandlingen med pembrolizumab og 31 i kontrolgruppen). En HR=0,96 [95% CI 0,37; 2,52] for OS, en HR=0,60 [95% CI 0,29; 1,21] for PFS, og en ORR på 47% og 42% for kombinationsbehandlingen med pembrolizumab *versus* kemoterapi blev rapporteret i denne studiesubgruppe. Data vedrørende pembrolizumabs virkning og sikkerhed i kombination med platinbaseret kemoterapi er begrænset i denne patientpopulation.

39

KEYNOTE-010: Kontrolleret studie med NSCLC-patienter, der tidligere har været i behandling med kemoterapi

Pembrolizumabs sikkerhed og virkning blev undersøgt i KEYNOTE-010, et åbent, kontrolleret multicenterstudie til behandling af fremskredent NSCLC hos patienter, der tidligere har fået platinbaseret kemoterapi. Patienterne havde PD-L1-ekspression med TPS ≥ 1% baseret på PD-L1 IHC 22C3 pharmDx^{TM}-analysen. Patienter med EGFR-aktiverende mutationer eller ALK-translokation havde også sygdomsprogression, mens de var i godkendt behandling for disse mutationer inden behandling med pembrolizumab. Patienterne blev randomiseret i forholdet 1:1:1 til at få pembrolizumab i en dosis på 2 mg/kg (n=344) eller 10 mg/kg (n=346) hver 3. uge eller docetaxel i en dosis på 75 mg/m^2 hver 3. uge (n=343) indtil sygdomsprogression eller uacceptabel toksicitet. Patienter med autoimmun sygdom, en lidelse, som kræver behandling med immunsuppressiva, og patienter, som havde fået mere end 30 Gy torakal bestråling inden for de forudgående 26 uger, blev ekskluderet fra studiet. Tumorstatus blev vurderet hver 9. uge.

Baseline-karakteristika for denne population inkluderede: medianalder 63 år (42% i alderen 65 år eller ældre); 61% mænd; 72% kaukasere og 21% asiater og henholdsvis 34% og 66% med ECOG-performance-status 0 og 1. Sygdomskarakteristika var planocellulær (21%) og ikke-planocellulær (70%); M1-stadie (91%); stabile hjernemetastaser (15%), og forekomsten af mutationer var EGFR (8%) og ALK (1%). Forudgående behandling inkluderede platinbaseret dublet-regime (100%); patienterne havde fået en (69%) eller to eller flere (29%) behandlingslinjer.

De primære resultatmål var OS og PFS baseret på BICR-vurdering i henhold til RECIST 1.1. Sekundære resultatmål var ORR og responsvarighed. I tabel 17 opsummeres de centrale effektresultater for hele populationen (TPS ≥ 1%) og for patienter med TPS ≥ 50%, og figur 11 viser Kaplan-Meier-kurverne for OS (TPS ≥ 1%) baseret på en endelig analyse med en median opfølgningstid på 42,6 måneder.

Tabel 17: Respons på pembrolizumab 2 eller 10 mg/kg hver 3. uge hos tidligere behandlede patienter med NSCLC i KEYNOTE-010

Endepunkt	Pembrolizumab 2 mg/kg hver 3. uge	Pembrolizumab 10 mg/kg hver 3. uge	Docetaxel 75 mg/m² hver 3. uge
**TPS ≥ 1%**			
Antal patienter	344	346	343
**OS**			
Antal (%) patienter med hændelse	284 (83%)	264 (76%)	295 (86%)
HR* (95% CI)	0,77 (0,66; 0,91)	0,61 (0,52; 0,73)	---
p-værdi†	0,00128	< 0,001	---
Median i måneder (95% CI)	10,4 (9,5; 11,9)	13,2 (11,2; 16,7)	8,4 (7,6; 9,5)
**PFS‡**			
Antal (%) patienter med hændelse	305 (89%)	292 (84%)	314 (92%)
HR* (95% CI)	0,88 (0,75; 1,04)	0,75 (0,63; 0,89)	---
p-værdi†	0,065	< 0,001	---
Median i måneder (95% CI)	3,9 (3,1; 4,1)	4,0 (2,7; 4,5)	4,1 (3,8; 4,5)
**Objektiv responsrate‡**			
ORR % (95% CI)	20% (16; 25)	21% (17; 26)	9% (6; 13)
Komplet respons %	2%	3%	0%
Partielt respons %	18%	18%	9%
**Responsvarighed‡,§**			
Median i måneder (interval)	Ikke nået (2,8; 46,2+)	37,8 (2,0+; 49,3+)	7,1 (1,4+; 16,8)
% igangværende respons¶	42%	43%	6%
**TPS ≥ 50%**			
Antal patienter	139	151	152
**OS**			
Antal (%) patienter med hændelse	97 (70%)	102 (68%)	127 (84%)
HR* (95% CI)	0,56 (0,43; 0,74)	0,50 (0,38; 0,65)	---
p-værdi†	< 0,001	< 0,001	---
Median i måneder (95% CI)	15,8 (10,8; 22,5)	18,7 (12,1; 25,3)	8,2 (6,4; 9,8)
**PFS‡**			
Antal (%) patienter med hændelse	107 (77%)	115 (76%)	138 (91%)
HR* (95% CI)	0,59 (0,45; 0,77)	0,53 (0,41; 0,70)	---
p-værdi†	< 0,001	< 0,001	---
Median i måneder (95% CI)	5,3 (4,1; 7,9)	5,2 (4,1; 8,1)	4,2 (3,8; 4,7)
**Objektiv responsrate‡**			
ORR % (95% CI)	32% (24; 40)	32% (25; 41)	9% (5; 14)
Komplet respons %	4%	4%	0%
Partielt respons %	27%	28%	9%
**Responsvarighed‡,§**			
Median i måneder (interval)	Ikke nået (2,8; 44,0+)	37,5 (2,0+; 49,3+)	8,1 (2,6; 16,8)
% igangværende respons¶	55%	47%	8%

* *Hazard ratio* (pembrolizumab sammenlignet med docetaxel) baseret på den stratificerede Cox *proportional hazard*-model

† Baseret på stratificeret log rank-test

‡ BICR-vurdering i henhold til RECIST 1.1

§ Baseret på patienter med bedste objektive respons, der er bekræftet komplet respons eller partielt respons

¶ Igangværende respons inkluderer alle patienter med respons, som på tidspunktet for analysen, var i live, progressionsfri, ikke initierede ny behandling mod cancer og ikke blev anset for være *lost to follow-up*

41

Figur 11: Kaplan-Meier-kurve for samlet overlevelse i hver behandlingsarm i KEYNOTE-010 (patienter med PD-L1-ekspression TPS ≥ 1%, *intent to treat*-population)

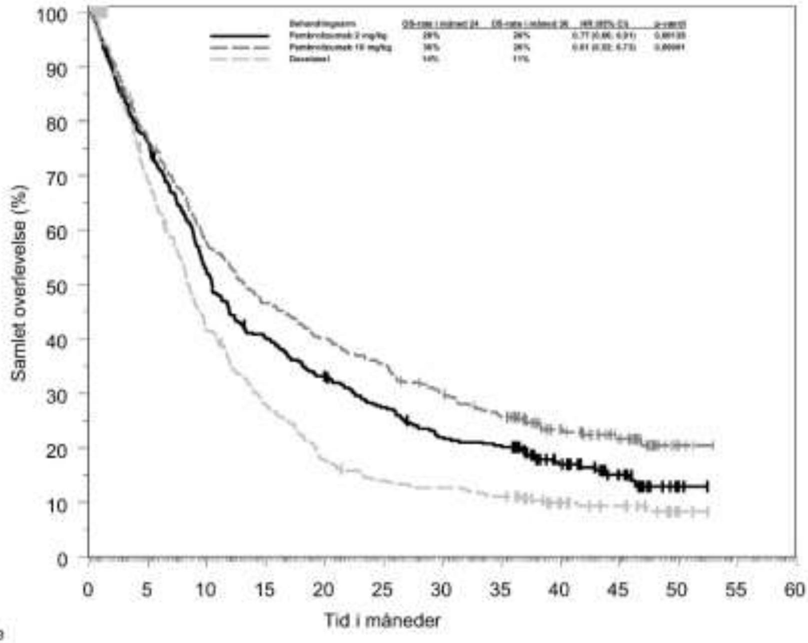

Antal i risikogruppe													
Pembrolizumab 2 mg/kg:	344	261	177	136	111	91	72	67	36	17	2	0	0
Pembrolizumab 10 mg/kg:	346	262	197	159	137	120	99	84	50	28	3	0	0
Docetaxel:	343	226	135	90	57	44	40	35	20	13	2	0	0

Effektresultaterne var sammenlignelige i armene med 2 mg/kg og 10 mg/kg pembrolizumab. Effektresultaterne for OS var konsistente uanset tumorprøvens alder (nyt væv *versus* arkivvæv) baseret på en sammenligning mellem grupperne.

I analyser af subgrupper viste pembrolizumab en reduceret fordel med hensyn til overlevelse sammenlignet med docetaxel hos patienter, som aldrig havde røget, og hos patienter med EGFR-aktiverende mutationer, der som minimum havde fået platinbaseret kemoterapi og en tyrosinkinasehæmmer. På grund af det lave antal patienter kan der dog ikke drages nogle definitive konklusioner på baggrund af disse data.

Pembrolizumabs virkning og sikkerhed hos patienter, hvis tumorer ikke udtrykker PD-L1, er ikke klarlagt.

Klassisk Hodgkins lymfom

KEYNOTE-087 og KEYNOTE-013: Åbne studier med patienter med recidiverende eller refraktært cHL

Pembrolizumabs virkning blev undersøgt i KEYNOTE-087 og KEYNOTE-013, to åbne multicenterstudier hos 241 patienter med cHL. Disse studier indrullerede patienter, som havde oplevet svigt af ASCT og svigt af behandling med BV; som var uegnede til ASCT, da de ikke var i stand til at opnå komplet eller partiel remission efter salvage-kemoterapi og havde oplevet svigt af behandling med BV; eller som havde oplevet svigt af ASCT og ikke havde fået BV. 5 forsøgspersoner var uegnede til ASCT af andre årsager end behandlingssvigt efter salvage-kemoterapi. Begge studier inkluderede patienter uanset PD-L1-ekspression. Patienter med aktiv, ikke-infektiøs pneumonitis, allogen transplantation inden for de seneste 5 år (eller > 5 år, men med GVHD), aktiv autoimmun sygdom eller en lidelse, som nødvendiggjorde behandling med immunsuppressiva, var uegnede til at deltage i begge

studier. Patienterne fik pembrolizumab 200 mg hver 3. uge (n=210; KEYNOTE-087) eller 10 mg/kg hver 2. uge (n=31; KEYNOTE-013) indtil uacceptabel toksicitet eller dokumenteret sygdomsprogression.

Hos patienterne i KEYNOTE-087 var *baseline*-karakteristika: medianalder 35 år (9% ≥ 65 år); 54% mænd; 88% kaukasere; og henholdsvis 49% og 51% havde ECOG-performance-status 0 og 1. Det mediane antal forudgående behandlingslinjer for cHL var 4 (1-12). 81% var refraktære over for mindst en forudgående behandling, inklusive 35%, som var refraktære over for førstelinjebehandling. 61% af patienterne havde gennemgået ASCT, 38% var uegnede til transplantation; 17% havde ikke tidligere fået brentuximab vedotin; og 36% af patienterne havde tidligere fået strålebehandling. Sygdomsundergrupperne var 80% nodulær sklerose, 11% blandet cellularitet, 4% lymfocytrig og 2% lymfocytfattig.

Hos patienterne i KEYNOTE-013 var *baseline*-karakteristika: medianalder 32 år (7% ≥ 65 år), 58% mænd, 94% kaukasere; og 45% og 55% havde henholdsvis ECOG-performance-status 0 og 1. Det mediane antal forudgående behandlingslinjer for cHL var 5 (2-15). 84% var refraktære over for mindst en forudgående behandling, inklusive 35%, som var refraktære over for førstelinjebehandling. 74% af patienterne havde gennemgået ASCT, 26% var uegnede til transplantation, og 45% af patienterne havde tidligere fået strålebehandling. Sygdomsundergrupperne var 97% nodulær sklerose og 3% blandet cellularitet.

De vigtigste resultatmål objektiv responsrate (ORR) og komplet remissionsrate (CRR) blev baseret på BICR-vurdering i henhold til IWG (*International Working Group*)-kriterierne revideret i 2007. Sekundære resultatmål var responsvarighed, PFS og OS. Respons blev vurderet i henholdsvis hver 12. (KEYNOTE-087) og hver 8. uge (KEYNOTE-013) med den første planlagte vurdering efter *baseline* i uge 12. Effektresultaterne opsummeres i tabel 18.

Tabel 18: Effektresultater i KEYNOTE-087 og KEYNOTE-013

Endepunkt	KEYNOTE-087[a] Pembrolizumab 200 mg hver 3. uge n=210	KEYNOTE-013[b] Pembrolizumab 10 mg/kg hver 2. uge n=31
**Objektiv responsrate**[c]		
ORR % (95% CI)	69% (62,3; 75,2)	58% (39,1; 75,5)
Komplet remission	22%	19%
Partiel remission	47%	39%
**Responssvarighed**[c]		
Median i måneder (interval)	11,1 (0,0+; 11,1)[d]	Ikke nået (0,0+; 45,6+)[e]
% med varighed ≥ 6 måneder	76%[f]	80%[g]
% med varighed ≥ 12 måneder	---	70%[h]
**Tid til respons**		
Median i måneder (interval)	2,8 (2,1; 8,8)[d]	2,8 (2,4; 8,6)[e]
**PFS**[c]		
Antal (%) patienter med hændelse	70 (33%)	19 (61%)
Median i måneder (95% CI)	11,3 (10,8; Ikke nået)	11,4 (4,9; 27,8)
6-måneders PFS-rate	72%	66%
9-måneders PFS-rate	62%	---
12-måneders PFS-rate	---	48%
**OS**		
Antal (%) patienter med hændelse	4 (2%)	6 (19%)
6-måneders OS-rate	99,5%	100%
12-måneders OS-rate	97,6%	87,1%

[a] Median opfølgningstid på 10,1 måneder
[b] Median opfølgningstid på 52,8 måneder
[c] BICR-vurderingi henhold til IWG (*International Working Group*)-kriterierne, revideret i 2007, ud fra PET/CT-scanninger
[d] Baseret på patienter (n=145) med et respons ud fra uafhængig vurdering
[e] Baseret på patienter (n=18) med et respons ud fra uafhængig vurdering
[f] Baseret på Kaplan-Meier-estimering; inkluderer 31 patienter med respons på 6 måneder eller længere
[g] Baseret på Kaplan-Meier-estimering; inkluderer 9 patienter med respons på 6 måneder eller længere
[h] Baseret på Kaplan-Meier-estimering; inkluderer 7 patienter med respons på 12 måneder eller længere

Sikkerhed og virkning hos ældre patienter
Alt i alt blev 20 cHL-patienter ≥ 65 år behandlet med pembrolizumab i studierne KEYNOTE-087 og KEYNOTE-013. Data fra disse patienter er for begrænsede til at kunne drage konklusioner om sikkerhed eller virkning for denne population.

Urotelialt karcinom
KEYNOTE-045: Kontrolleret studie med patienter med urotelialt karcinom, som tidligere har fået platin-baseret kemoterapi
Pembrolizumabs sikkerhed og virkning blev vurderet i KEYNOTE-045, et åbent, randomiseret (1:1), kontrolleret multicenterstudie til behandling af lokalt fremskredent eller metastatisk urotelialt karcinom hos patienter med sygdomsprogression ved eller efter platinbaseret kemoterapi. Patienterne skal have fået et *first line* platinbaseret regime for lokalt fremskredent/metastatisk sygdom eller som neoadjuvant/adjuvant behandling med tilbagevenden/progression ≤ 12 måneder efter afsluttet behandling. Patienterne blev randomiseret (1:1) til at få pembrolizumab 200 mg hver 3. uge (n=270) eller investigators valg af en hvilken som helst af følgende kemoterapi-regimer, der alle gives intravenøst hver 3. uge (n=272): paclitaxel 175 mg/m^2 (n=84), docetaxel 75 mg/m^2 (n=84) eller vinflunin 320 mg/m^2 (n=87). Patienterne blev behandlet med pembrolizumab indtil uacceptabel toksicitet eller sygdomsprogression. Behandlingen kunne fortsætte efter sygdomsprogression, hvis patienten var klinisk stabil, og investigator mente, at patienten havde en klinisk fordel. Patienter uden sygdomsprogression kunne behandles i op til 24 måneder. Patienter med autoimmun sygdom, en medicinsk tilstand som krævede behandling med immunsuppressiva, og patienter som tidligere havde fået mere end 2 omgange

systemisk kemoterapi mod metastatisk urotelialt karcinom blev ekskluderet fra studiet. Patienter med ECOG-performance-status på 2 skulle have et hæmoglobinniveau på ≥ 10 g/dl, måtte ikke have levermetastaser og måtte have fået den sidste dosis af det seneste tidligere kemoterapiregime ≥ 3 måneder før inklusion. Vurdering af tumorstatus blev foretaget 9 uger efter første dosis, derefter hver 6. uge i det første år, og efterfølgende hver 12. uge.

Blandt de 542 randomiserede patienter i KEYNOTE-045 var *baseline*-karakteristika følgende: median alder 66 år (interval: 26 til 88), 58% i alderen 65 år eller ældre; 74% mænd; 72% hvide og 23% asiater; 56% havde ECOG-performance-status på 1, og 1% havde ECOG-performance-status på 2; og 96% havde M1 sygdom, og 4% havde M0 sygdom. 87% af patienterne havde viscerale metastaser, herunder 34% med levermetastaser. 86% havde en primær tumor i de nedre urinveje, og 14% havde en primær tumor i de øvre urinveje. 15% af patienterne havde sygdomsprogression efter tidligere platinbaseret neoadjuvant eller adjuvant kemoterapi. 21% havde tidligere fået 2 systemiske regimer til behandling af metastaser. 76% af patienterne havde tidligere fået cisplatin, 23% havde tidligere fået carboplatin, og 1% var blevet behandlet med andre platinbaserede regimer.

De primære resultatmål var OS og PFS baseret på BICR i henhold til RECIST version 1.1. De sekundære resultatmål var ORR (baseret på BICR i henhold til RECIST version 1.1) og responsvarighed. I tabel 19 opsummeres de centrale effektresultater for ITT-populationen ved den endelige analyse. Kaplan-Meier-kurven baseret på den endelige analyse for OS er vist i figur 12. Studiet viste statistisk signifikante forbedringer i OS og ORR for patienter, der var randomiseret til pembrolizumab sammenlignet med kemoterapi. Der var ingen statistisk signifikant forskel mellem pembrolizumab og kemoterapi med hensyn til PFS.

Tabel 19: Respons på pembrolizumab 200 mg hver 3. uge hos patienter med urotelialt karcinom, der tidligere var blevet behandlet med kemoterapi i KEYNOTE-045

Endepunkt	Pembrolizumab 200 mg hver 3. uge n=270	Kemoterapi n=272
**OS**		
Antal (%) patienter med hændelse	200 (74%)	219 (81%)
HR* (95% CI)	0,70 (0,57; 0,85)	
p-værdi†	< 0,001	
Median i måneder (95% CI)	10,1 (8,0; 12,3)	7,3 (6,1; 8,1)
**PFS‡**		
Antal (%) patienter med hændelse	233 (86%)	237 (87%)
HR* (95% CI)	0,96 (0,79; 1,16)	
p-værdi†	0,313	
Median i måneder (95% CI)	2,1 (2,0; 2,2)	3,3 (2,4; 3,6)
**Objektiv responsrate‡**		
ORR % (95% CI)	21% (16; 27)	11% (8; 15)
p-værdi§	< 0,001	
Komplet respons	9%	3%
Partielt respons	12%	8%
Stabil sygdom	17%	34%
**Responsvarighed‡,¶**		
Median i måneder (interval)	Ikke nået (1,6+; 30,0+)	4,4 (1,4+; 29,9+)
Antal (%#) patienter med varighed ≥ 6 måneder	46 (84%)	8 (47%)
Antal (%#) patienter med varighed ≥ 12 måneder	35 (68%)	5 (35%)

* *Hazard ratio* (pembrolizumab sammenlignet med kemoterapi) baseret på den stratificerede Cox *proportional hazard-model*
† Baseret på stratificeret log rank-test
‡ BICR-vurdering i henhold til RECIST 1.1
§ Baseret på Miettinen-og Nurminen-metoden
¶ Baseret på patienter med bedste objektive respons, der er bekræftet komplet eller partielt respons
\# Baseret på Kaplan-Meier-estimering

46

Figur 12: Kaplan-Meier-kurve for samlet overlevelse i hver behandlingsarm i KEYNOTE-045 (*intent to treat*-population)

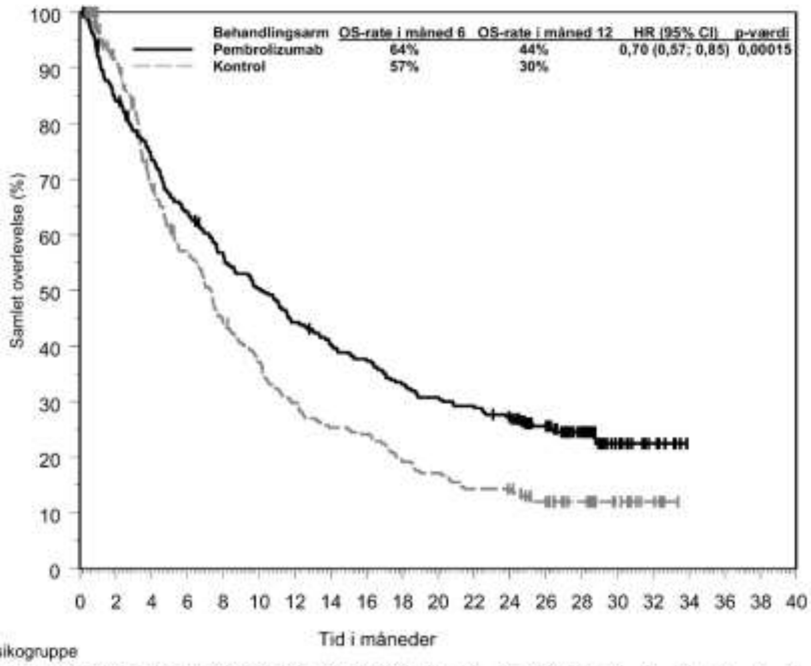

Antal i risikogruppe

Pembrolizumab:	270	226	195	170	148	132	116	105	98	86	80	76	67	52	33	14	7	0	0	0	0
Kontrol:	272	234	173	140	109	91	73	62	59	47	42	35	34	24	18	10	4	0	0	0	0

Der blev udført en analyse i KEYNOTE-045 hos patienter, der havde PD-L1 CPS < 10 [pembrolizumab: n=186 (69%) *versus* kemoterapi: n=176 (65%)] eller ≥ 10 [pembrolizumab: n=74 (27%) *versus* kemoterapi: n=90 (33%)] i både pembrolizumab- og kemoterapi-armene (se tabel 20).

Tabel 20: OS ved PD-L1-ekspression

PD-L1-ekspression	Pembrolizumab	Kemoterapi	
	OS ved PD-L1-ekspression Antal (%) patienter med hændelse*		HR[†] (95% CI)
CPS < 10	140 (75%)	144 (82%)	0,75 (0,59; 0,95)
CPS ≥ 10	53 (72%)	72 (80%)	0,55 (0,37; 0,81)

* Baseret på den endelige analyse
† *Hazard ratio* (pembrolizumab sammenlignet med kemoterapi) baseret på stratificeret Cox *proportional hazard*-model

Patient-rapporterede oplysninger (PRO) blev vurderet i henhold til EORTC QLQ-C30. I EORTC QLQ-C30 global helbredstilstand/QoL sås en forlængelse af tiden til forværring hos patienter, der var blevet behandlet med pembrolizumab sammenlignet med investigators valg af kemoterapi (HR 0,70; 95% CI 0,55-0,90). I løbet af 15 ugers opfølgning opnåede patienter, der var blevet behandlet med pembrolizumab, en stabil global helbredstilstand/QoL, hvorimod de der var blevet behandlet med investigators valg af kemoterapi fik en nedgang i global helbredstilstand/QoL. Disse resultater skal fortolkes i konteksten af det åbne studiedesign og skal derfor fortolkes med forsigtighed.

KEYNOTE-052: Åbent studie med patienter med urotelialt karcinom, der er uegnede til cisplatinbaseret kemoterapi

Pembrolizumabs sikkerhed og virkning blev undersøgt i KEYNOTE-052, et åbent, multicenterstudie til behandling af lokalt fremskredent eller metastatisk urotelialt karcinom hos patienter, som var uegnede til behandling med cisplatinbaseret kemoterapi. Patienterne fik pembrolizumab i doser på 200 mg hver 3. uge, indtil uacceptabel toksicitet eller sygdomsprogression. Behandlingen kunne fortsætte efter progression, hvis patienten var klinisk stabil, og investigator mente, at patienten havde en klinisk fordel. Patienter uden sygdomsprogression kunne behandles i op til 24 måneder. Patienter med autoimmun sygdom eller en medicinsk tilstand, som krævede behandling med immunsuppressiva blev ekskluderet fra studiet. Vurdering af tumorstatus blev foretaget 9 uger efter første dosis, derefter hver 6. uge i det første år, og efterfølgende hver 12. uge.

Blandt de 370 patienter med urotelialt karcinom, som var uegnede til cisplatinbaseret kemoterapi var *baseline*-karakteristika følgende: median alder 74 år (82% i alderen 65 år eller ældre); 77% mænd; og 89% hvide og 7% asiater. 88% havde M1-sygdom, og 12% havde M0-sygdom. 85% af patienterne havde viscerale metastaser, herunder 21% med levermetastaser. Årsager til cisplatin-uegnethed var: creatinin-clearance på < 60 ml/min (50%) ved *baseline*, ECOG-performance-status på 2 (32%), ECOG-performance-status på 2 og creatinin-clearance på < 60 ml/min (9%) ved *baseline* og andre (hjertesvigt klasse III, perifer neuropati grad 2 eller højere og høretab grad 2 eller højere; 9%). 90% af patienterne var behandlingsnaive, og 10% havde tidligere fået adjuvant eller neoadjuvant platinbaseret kemoterapi. 81% havde en primær tumor i den nederste del af urinvejene, og 19% havde en primær tumor i de øvre urinveje.

Det primære resultatmål var ORR baseret på BICR i henhold til RECIST version 1.1. De sekundære resultatmål var responsvarighed, PFS og OS. I tabel 21 opsummeres de centrale effektresultater for studiepopulationen ved den endelige analyse på basis af en median opfølgningstid på 11,4 måneder (interval: 0,1; 41,2 måneder) for alle patienter.

Tabel 21: Respons på pembrolizumab 200 mg hver 3. uge hos patienter med urotelialt karcinom, der var uegnede til behandling med cisplatinbaseret kemoterapi i KEYNOTE-052

Endepunkt	n=370
**Objektiv responsrate***	
ORR %, (95% CI)	29% (24; 34)
Sygdomskontrolrate†	47%
Komplet respons	9%
Partielt respons	20%
Stabil sygdom	18%
**Responsvarighed**	
Median i måneder (interval)	30,1 (1,4+; 35,9+)
% med varighed ≥ 6 måneder	81%‡
**Tid til respons**	
Median i måneder (interval)	2,1 (1,3; 9,0)
**PFS***	
Median i måneder (95% CI)	2,2 (2,1; 3,4)
6-måneders PFS-rate	33%
12-måneders PFS-rate	22%
**OS**	
Median i måneder (95% CI)	11,3 (9,7; 13,1)
6-måneders OS-rate	67%
12-måneders OS-rate	47%

* BICR-vurdering i henhold til RECIST 1.1
† Baseret på bedst respons for stabil sygdom eller bedre
‡ Baseret på Kaplan-Meier-estimering; omfatter 84 patienter med 6 måneders respons eller længere

Der blev udført en analyse i KEYNOTE-052 hos patienter, som havde tumorer, der udtrykte PD-L1 med CPS < 10 (n=251; 68%) eller ≥ 10 (n=110; 30%) baseret på PD-L1 IHC 22C3 pharmDx™-analysen (se tabel 22).

Tabel 22: ORR og OS ved PD-L1-ekspression

Endepunkt	CPS < 10<br>N=251	CPS ≥ 10<br>N=110
**Objektiv responsrate***		
ORR %, (95% CI)	20% (16; 26)	47% (38; 57)
**OS**		
Median i måneder (95% CI)	10 (8; 12)	19 (12; 29)
12-måneders OS-rate	41%	61%

* BICR i henhold til RECIST 1.1

KEYNOTE-361 er et igangværende randomiseret, kontrolleret, åbent klinisk fase III-studie af pembrolizumab med eller uden platinbaseret kombineret kemoterapi *versus* kemoterapi som førstelinjebehandling hos forsøgspersoner med fremskredent eller metastatisk urotelialt karcinom. Foreløbige data fra en tidlig gennemgang viste en nedsat overlevelse med pembrolizumab som monoterapi hos patienter, hvis tumorer udtrykker PD-L1 med CPS < 10 sammenlignet med standardkemoterapi.

På basis af en anbefaling fra en ekstern datamonitoreringskomité blev tilgangen af patienter i armen med pembrolizumab som monoterapi stoppet for patienter, hvis tumorer udtrykker PD-L1 med CPS < 10.

Armen med pembrolizumab som monoterapi forbliver kun åben for patienter, hvis tumorer udtrykker PD-L1 med CPS ≥ 10. Forsøgspersoner, hvis tumorer udtrykker PD-L1 CPS < 10 og som allerede er indrulleret i armen med pembrolizumab som monoterapi, kan fortsætte behandlingen. Randomisering til kemoterapi- og kemoterapi-pembrolizumab-armene forbliver åben.

Planocellulært hoved-hals karcinom
KEYNOTE-048: Kontrolleret studie af monoterapi og kombinationsbehandling hos behandlingsnaive HNSCC-patienter i behandling for recidiverende eller metastatisk sygdom
Pembrolizumabs virkning blev undersøgt i KEYNOTE-048, et randomiseret, åbent, aktivt kontrolleret multicenterstudie hos patienter med histologisk bekræftet metastatisk eller recidiverende HNSCC i mundhulen, svælget (farynx) eller struben (larynx), som ikke tidligere havde fået systemisk behandling for recidiverende eller metastatisk sygdom, og som blev anset for at være uhelbredeligt med lokal behandling. Patienter med nasofaryngealt karcinom, aktiv autoimmun sygdom, der havde krævet systemisk behandling inden for 2 år efter behandling, eller lidelser, som krævede behandling med immunsuppressiva, var uegnede til studiet. Randomiseringen blev stratificeret ud fra PD-L1-tumorekspression (TPS ≥ 50% eller < 50%), HPV-status (positiv eller negativ) og ECOG-PS (0 *versus* 1). Patienterne blev randomiseret i forholdet 1:1:1 til en af følgende behandlingsarme:

- Pembrolizumab 200 mg hver 3. uge
- Pembrolizumab 200 mg hver 3. uge, carboplatin AUC 5 mg/ml/min hver 3. uge eller cisplatin 100 mg/m^2 hver 3. uge, og 5-FU 1.000 mg/m^2/dag kontinuerligt i 4 dage hver 3. uge (maksimalt 6 serier med platinbaseret behandling og 5-FU)
- Cetuximab 400 mg/m^2 støddosis og herefter 250 mg/m^2 en gang ugentligt, carboplatin AUC 5 mg/ml/min hver 3. uge eller cisplatin 100 mg/m^2 hver 3. uge, og 5-FU 1.000 mg/m^2/dag kontinuerligt i 4 dage hver 3. uge (maksimalt 6 serier med platinbaseret behandling og 5-FU)

Behandling med pembrolizumab fortsatte indtil sygdomsprogression defineret i henhold til RECIST 1.1 efter investigators vurdering, uacceptabel toksicitet eller i højst 24 måneder. Administration af pembrolizumab var tilladt efter sygdomsprogression defineret i henhold til RECIST, hvis patienten var klinisk stabil, og investigator vurderede, at patienten havde en klinisk fordel. Vurdering af tumorstatus blev foretaget i uge 9 og herefter hver 6. uge i løbet af det første år, og efterfølgende hver 9. uge til og med måned 24.

Blandt de 882 patienter i KEYNOTE-048, havde 754 (85%) tumorer, som udtrykte PD-L1 med CPS ≥ 1 baseret på PD-L1 IHC 22C3 pharmDx™-analysen. Karakteristika ved *baseline* for disse 754 patienter inkluderede: medianalder på 61 år (interval: 20 til 94); 36% i alderen 65 eller ældre; 82% mænd; 74% kaukasere og 19% asiatere; 61% med ECOG-performance-status 1; og 77% tidligere/nuværende rygere. Sygdomskarakteristika var: 21% HPV-positive og 95% havde sygdom i stadie IV (stadie IVa 21%, stadie IVb 6% og stadie IVc 69%).

De primære resultatmål var OS og PFS (BICR-vurdering i henhold til RECIST 1.1). Studiet viste en statistisk signifikant forbedring i OS for alle patienter, som var randomiseret til pembrolizumab i kombination med kemoterapi sammenlignet med standardbehandling (HR: 0,72; 95% CI 0,60-0,87), og hos patienter, hvis tumorer udtrykte PD-L1 CPS ≥ 1, som var randomiseret til pembrolizumab som monoterapi sammenlignet med standardbehandling. I tabel 23 og 24 opsummeres de centrale effektresultater for pembrolizumab hos patienter, hvis tumorer udtrykte PD-L1 med CPS ≥ 1 i KEYNOTE-048 ved den endelige analyse udført med en median opfølgningstid på 13 måneder for pembrolizumab i kombination med kemoterapi og med en median opfølgningstid på 11,5 måneder for pembrolizumab som monoterapi. Kaplan-Meier-kurver for OS baseret på den endelige analyse er vist i figur 13 og 14.

Tabel 23: Effektresultater for pembrolizumab plus kemoterapi i KEYNOTE-048 med PD-L1-ekspression (CPS ≥ 1)

Endepunkt	Pembrolizumab + platinbaseret kemoterapi + 5-FU n=242	Standardbehandling* n=235
**OS**		
Antal (%) patienter med hændelse	177 (73%)	213 (91%)
Median i måneder (95% CI)	13,6 (10,7; 15,5)	10,4 (9,1; 11,7)
HR† (95% CI)	0,65 (0,53; 0,80)	
p-værdi‡	0,00002	
**PFS**		
Antal (%) patienter med hændelse	212 (88%)	221 (94%)
Median i måneder (95% CI)	5,1 (4,7; 6,2)	5,0 (4,8; 6,0)
HR† (95% CI)	0,84 (0,69; 1,02)	
p-værdi‡	0,03697	
**Objektiv responsrate**		
ORR§ (95% CI)	36% (30,3; 42,8)	36% (29,6; 42,2)
Komplet respons	7%	3%
Partielt respons	30%	33%
p-værdi¶	0,4586	
**Responsvarighed**		
Median i måneder (interval)	6,7 (1,6+; 39,0+)	4,3 (1,2+; 31,5+)
% med varighed ≥ 6 måneder	54%	34%

* Cetuximab, platinbaseret behandling og 5-FU
† Baseret på den stratificerede Cox *proportional hazard*-model
‡ Baseret på stratificeret log rank-test
§ Respons: Bedste objektive respons, der er bekræftet komplet eller partielt respons
¶ Baseret på Miettinen- og Nurminen-metoden stratificeret ud fra ECOG (0 *versus* 1), HPV-status (positiv *versus* negativ) og PD-L1-status (markant positiv *versus* ikke markant positiv)

Figur 13: Kaplan-Meier-kurve for samlet overlevelse for pembrolizumab plus kemoterapi i KEYNOTE-048 med PD-L1-ekspression (CPS ≥ 1)

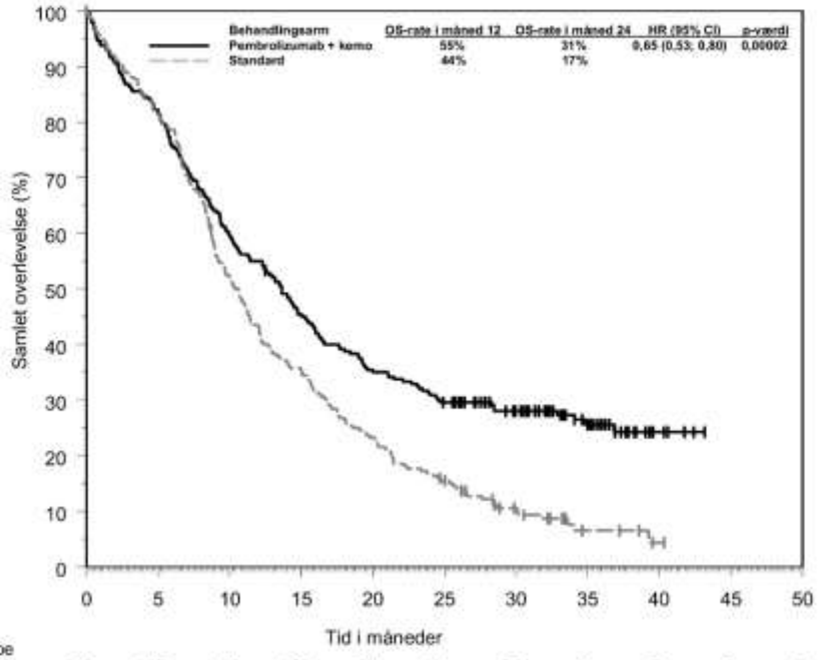

Antal i risikogruppe											
Pembrolizumab + kemo:	242	197	144	109	84	70	52	29	5	0	0
Standard:	235	191	122	83	54	35	17	5	1	0	0

52

Tabel 24: Effektresultater for pembrolizumab som monoterapi i KEYNOTE-048 med PD-L1-ekspression (CPS ≥ 1)

Endepunkt	Pembrolizumab n=257	Standardbehandling* n=255
**OS**		
Antal (%) patienter med hændelse	197 (77%)	229 (90%)
Median i måneder (95% CI)	12,3 (10,8; 14,3)	10,3 (9,0; 11,5)
HR[†] (95% CI)	0,74 (0,61; 0,90)	
p-værdi[‡]	0,00133	
**PFS**		
Antal (%) patienter med hændelse	228 (89%)	237 (93%)
Median i måneder (95% CI)	3,2 (2,2; 3,4)	5,0 (4,8; 6,0)
HR[†] (95% CI)	1,13 (0,94; 1,36)	
p-værdi[‡]	0,89580	
**Objektiv responsrate**		
ORR[§] (95% CI)	19,1% (14,5; 24,4)	35% (29,1; 41,1)
Komplet respons	5%	3%
Partielt respons	14%	32%
p-værdi[¶]	1,0000	
**Responsvarighed**		
Median i måneder (interval)	23,4 (1,5+; 43,0+)	4,5 (1,2+; 38,7+)
% med varighed ≥ 6 måneder	81%	36%

* Cetuximab, platinbaseret behandling og 5-FU
† Baseret på den stratificerede Cox *proportional hazard*-model
‡ Baseret på stratificeret log rank-test
§ Respons: Bedste objektive respons, der er bekræftet komplet eller partielt respons
¶ Baseret på Miettinen- og Nurminen-metoden stratificeret ud fra ECOG (0 *versus* 1), HPV-status (positiv *versus* negativ) og PD-L1-status (markant positiv *versus* ikke markant positiv)

Figur 14: Kaplan-Meier-kurve for samlet overlevelse for pembrolizumab som monoterapi i KEYNOTE-048 med PD-L1-ekspression (CPS ≥ 1)

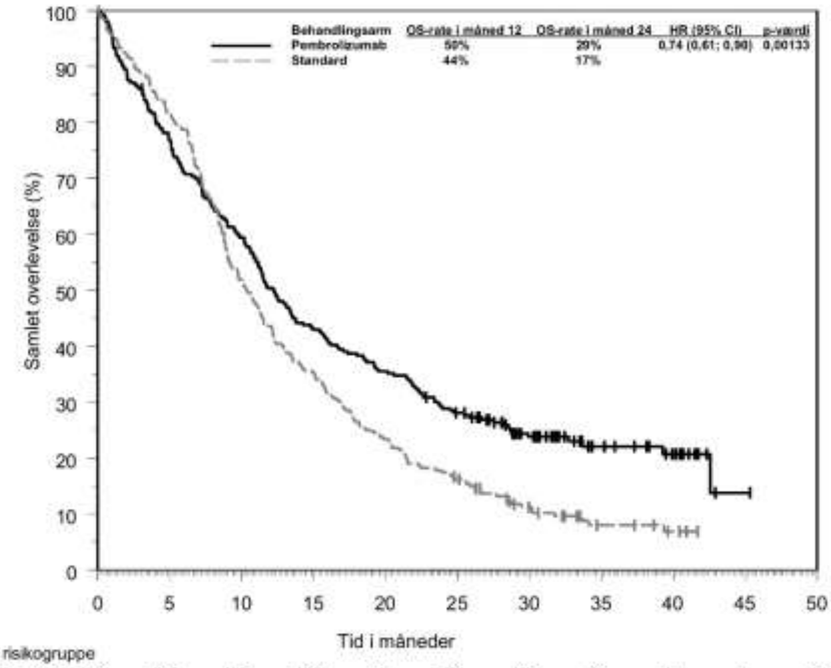

Antal i risikogruppe										
Pembrolizumab:	257	197	152	110	91	70	43	21	13	1	0
Standard:	255	207	131	89	59	40	21	9	5	0	0

Der blev udført en analyse i KEYNOTE-048 hos patienter, hvis tumorer udtrykte PD-L1 CPS ≥ 20 [pembrolizumab plus kemoterapi: n=126 (49%) *versus* standardbehandling: n=110 (43%) og pembrolizumab som monoterapi: n=133 (52%) *versus* standardbehandling: n=122 (48%)] (se tabel 25).

54

Tabel 25: Effektresultater for pembrolizumab plus kemoterapi og pembrolizumab som monoterapi ud fra PD-L1-ekspression i KEYNOTE-048 (CPS ≥ 20)

Endepunkt	Pembrolizumab + platinbaseret kemoterapi + 5-FU n=126	Standard-behandling* n=110	Pembrolizumab-monoterapi n=133	Standard-behandling* n=122
**OS**				
Antal (%) patienter med hændelse	84 (66,7)	98 (89,1)	94 (70,7)	108 (88,5)
Median i måneder (95% CI)	14,7 (10,3; 19,3)	11,0 (9,2; 13,0)	14,8 (11,5; 20,6)	10,7 (8,8; 12,8)
HR† (95% CI)	0,60 (0,45; 0,82)		0,58 (0,44; 0,78)	
p-værdi‡	0,00044		0,00010	
OS-rate i måned 6 (95% CI)	74,6 (66,0; 81,3)	80,0 (71,2; 86,3)	74,4 (66,1; 81,0)	79,5 (71,2; 85,7)
OS-rate i måned 12 (95% CI)	57,1 (48,0; 65,2)	46,1 (36,6; 55,1)	56,4 (47,5; 64,3)	44,9 (35,9; 53,4)
OS-rate i måned 24 (95% CI)	35,4 (27,2; 43,8)	19,4 (12,6; 27,3)	35,3 (27,3; 43,4)	19,1 (12,7; 26,6)
**PFS**				
Antal (%) patienter med hændelse	106 (84,1)	104 (94,5)	115 (86,5)	114 (93,4)
Median i måneder (95% CI)	5,8 (4,7; 7,6)	5,3 (4,9; 6,3)	3,4 (3,2; 3,8)	5,3 (4,8; 6,3)
HR† (95% CI)	0,76 (0,58; 1,01)		0,99 (0,76; 1,29)	
p-værdi+	0,02951		0,46791	
PFS-rate i måned 6 (95% CI)	49,4 (40,3; 57,9)	47,2 (37,5; 56,2)	33,0 (25,2; 41,0)	46,6 (37,5; 55,2)
PFS-rate i måned 12 (95% CI)	23,9 (16,7; 31,7)	14,0 (8,2; 21,3)	23,5 (16,6; 31,1)	15,1 (9,3; 22,2)
PFS-rate i måned 24 (95% CI)	14,6 (8,9; 21,5)	5,0 (1,9; 10,5)	16,8 (10,9; 23,8)	6,1 (2,7; 11,6)
**Objektiv responsrate**				
ORR§ (95% CI)	42,9 (34,1; 52,0)	38,2 (29,1; 47,9)	23,3 (16,4; 31,4)	36,1 (27,6; 45,3)
**Responsvarighed**				
Antal patienter med respons	54	42	31	44
Median i måneder (interval)	7,1 (2,1+; 39,0+)	4,2 (1,2+; 31,5+)	22,6 (2,7+; 43,0+)	4,2 (1,2+; 31,5+)

* Cetuximab, platinbaseret behandling og 5-FU
† Baseret på den stratificerede Cox *proportional hazard*-model
‡ Baseret på stratificeret log rank-test
§ Respons: Bedste objektive respons, der er bekræftet komplet eller partielt respons

Der blev udført en eksploratorisk subgruppeanalyse i KEYNOTE-048 hos patienter, hvis tumorer udtrykte PD-L1 CPS ≥ 1 til < 20 [pembrolizumab plus kemoterapi: n=116 (45%) *versus* standardbehandling: n=125 (49%) og pembrolizumab som monoterapi: n=124 (48%) *versus* standardbehandling: n=133 (52%)] (se tabel 26).

55

Tabel 26: Effektresultater for pembrolizumab plus kemoterapi og pembrolizumab som monoterapi ud fra PD-L1-ekspression i KEYNOTE-048 (CPS ≥ 1 til < 20)

Endepunkt	Pembrolizumab + platinbaseret kemoterapi + 5-FU n=116	Standard-behandling* n=125	Pembrolizumab-monoterapi n=124	Standard-behandling* n=133
**OS**				
Antal (%) patienter med hændelse	93 (80,2)	115 (92,0)	103 (83,1)	121 (91,0)
Median i måneder (95% CI)	12,7 (9,4; 15,3)	9,9 (8,6; 11,5)	10,8 (9,0; 12,6)	10,1 (8,7; 12,1)
HR† (95% CI)	0,71 (0,54; 0,94)		0,86 (0,66; 1,12)	
OS-rate i måned 6 (95% CI)	76,7 (67,9; 83,4)	77,4 (69,0; 83,8)	67,6 (58,6; 75,1)	78,0 (70,0; 84,2)
OS-rate i måned 12 (95% CI)	52,6 (43,1; 61,2)	41,1 (32,4; 49,6)	44,0 (35,1; 52,5)	42,4 (33,9; 50,7)
OS-rate i måned 24 (95% CI)	25,9 (18,3; 34,1)	14,5 (9,0; 21,3)	22,0 (15,1; 29,6)	15,9 (10,3; 22,6)
**PFS**				
Antal (%) patienter med hændelse	106 (91,4)	117 (93,6)	113 (91,1)	123 (92,5)
Median i måneder (95% CI)	4,9 (4,2; 5,3)	4,9 (3,7; 6,0)	2,2 (2,1; 2,9)	4,9 (3,8; 6,0)
HR† (95% CI)	0,93 (0,71; 1,21)		1,25 (0,96; 1,61)	
PFS-rate i måned 6 (95% CI)	40,1 (31,0; 49,0)	40,0 (31,2; 48,5)	24,2 (17,1; 32,0)	41,4 (32,8; 49,7)
PFS-rate i måned 12 (95% CI)	15,1 (9,1; 22,4)	11,3 (6,4; 17,7)	17,5 (11,4; 24,7)	12,1 (7,2; 18,5)
PFS-rate i måned 24 (95% CI)	8,5 (4,2; 14,7)	5,0 (1,9; 10,1)	8,3 (4,3; 14,1)	6,3 (2,9; 11,5)
**Objektiv responsrate**				
ORR‡ (95% CI)	29,3 (21,2; 38,5)	33,6 (25,4; 42,6)	14,5 (8,8; 22,0)	33,8 (25,9; 42,5)
**Responsvarighed**				
Antal patienter med respons	34	42	18	45
Median i måneder (interval)	5,6 (1,6+; 25,6+)	4,6 (1,4+; 31,4+)	NR (1,5+; 38,9+)	5,0 (1,4+; 38,7+)

* Cetuximab, platinbaseret behandling og 5-FU
† Baseret på den stratificerede Cox *proportional hazard*-model
‡ Respons: Bedste objektive respons, der er bekræftet komplet eller partielt respons

KEYNOTE-040: Kontrolleret studie med HNSCC-patienter, som tidligere har været behandlet med platinbaseret kemoterapi

Pembrolizumabs sikkerhed og virkning blev undersøgt i KEYNOTE-040, et åbent, randomiseret, kontrolleret multicenterstudie til behandling af histologisk bekræftet recidiverende eller metastatisk HNSCC i mundhulen, svælget (farynx) eller struben (larynx) hos patienter, som havde sygdomsprogression under eller efter platinbaseret kemoterapi administreret til behandling af recidiverende eller metastatisk HNSCC eller efter platinbaseret kemoterapi administreret som en del af induktionsbehandling, samtidig behandling eller adjuverende behandling, og som ikke var modtagelige for lokal kurativt intenderet behandling. Patienterne blev stratificeret ud fra PD-L1-tumorekspression (TPS ≥ 50%), HPV-status og ECOG-performance-status og blev herefter randomiseret i forholdet 1:1 til at få enten pembrolizumab 200 mg hver 3. uge (n=247) eller en af tre standardbehandlinger (n=248): methotrexat 40 mg/m^2 en gang ugentligt (n=64), docetaxel 75 mg/m^2 en gang hver 3. uge (n=99) eller cetuximab 400 mg/m^2 støddosis og herefter 250 mg/m^2 en gang ugentligt (n=71). Behandlingen kunne fortsætte efter sygdomsprogression, hvis patienten var klinisk stabil, og investigator vurderede, at patienten havde en klinisk fordel. Patienter med nasofaryngealt karcinom, aktiv autoimmun sygdom, der

56

havde krævet systemisk behandling inden for de seneste 2 år efter behandling, lidelser, som krævede behandling med immunsuppressiva, eller som tidligere havde fået 3 eller flere systemiske regimer til behandling af recidiverende og/eller metastatisk HNSCC, blev ekskluderet fra studiet. Vurdering af tumorstatus blev foretaget i uge 9, herefter hver 6. uge til og med uge 52, og efterfølgende hver 9. uge til og med måned 24.

Blandt de 495 patienter i KEYNOTE-040 havde 129 (26%) tumorer, der udtrykte PD-L1 med TPS ≥ 50% baseret på PD-L1 IHC 22C3 pharmDx™-analysen. *Baseline*-karakteristika for disse 129 patienter inkluderede: medianalder 62 år (40% i alderen 65 år eller ældre); 81% mænd; 78% kaukasere, 11% asiater og 2% negroide; henholdsvis 23% og 77% havde ECOG-performance-status på 0 eller 1; og 19% havde HPV-positive tumorer. 67% af patienterne havde M1-sygdom og størstedelen havde sygdom i stadie IV (stadie IV 32%, stadie IVa 14%, stadie IVb 4% og stadie IVc 44%). 16% havde sygdomsprogression efter platinbaseret neoadjuverende eller adjuverende kemoterapi, og 84% havde tidligere fået 1-2 systemiske regimer for metastatisk sygdom.

Det primære resultatmål var OS i ITT-populationen. Den initiale analyse resulterede i en HR for OS på 0,82 (95% CI: 0,67; 1,01) med ensidet p-værdi på 0,0316. Den mediane OS var 8,4 måneder for pembrolizumab sammenlignet med 7,1 måneder for standardbehandling. I tabel 27 opsummeres de centrale effektresultater for populationen med TPS ≥ 50%. Kaplan-Meier-kurver for OS for populationen med TPS ≥ 50% er vist i figur 15.

Tabel 27: Effekt af pembrolizumab 200 mg hver 3. uge hos HNSCC-patienter med TPS ≥ 50%, som tidligere har været behandlet med platinbaseret kemoterapi i KEYNOTE-040

Endepunkt	Pembrolizumab 200 mg hver 3. uge n=64	Standardbehandling* n=65
**OS**		
Antal (%) patienter med hændelse	41 (64)	56 (86)
HR[†] (95% CI)	0,53 (0,35; 0,81)	
p-værdi[‡]	0,001	
Median i måneder (95% CI)	11,6 (8,3; 19,5)	6,6 (4,8; 9,2)
**PFS[§]**		
Antal (%) patienter med hændelse	52 (81)	58 (89)
HR[†] (95% CI)	0,58 (0,39; 0,86)	
p-værdi[‡]	0,003	
Median i måneder (95% CI)	3,5 (2,1; 6,3)	2,1 (2,0; 2,4)
6-måneders rate (%) (95% CI)	40,1 (28,1; 51,9)	17,1 (8,8; 27,7)
**Objektiv responsrate[§]**		
ORR% (95% CI)	26,6 (16,3; 39,1)	9,2 (3,5; 19,0)
p-værdi[¶]	0,0009	
Komplet respons	5%	2%
Partielt respons	22%	8%
Stabil sygdom	23%	23%
**Responsvarighed[§,#]**		
Median i måneder (interval)	Ikke nået (2,7; 13,8+)	6,9 (4,2; 18,8)
Antal (%[Þ]) patienter med varighed ≥ 6 måneder	9 (66)	2 (50)

* Methotrexat, docetaxel eller cetuximab
† *Hazard ratio* (pembrolizumab sammenlignet med standardbehandling) baseret på stratificeret Cox *proportional hazard*-model
‡ Ensidet p-værdi baseret på log-rank test
§ BICR-vurdering i henhold til RECIST 1.1
¶ Baseret på Miettinen- og Nurminen-metoden
\# Baseret på patienter med bedste objektive respons, der er bekræftet komplet eller partielt respons
Þ Baseret på Kaplan-Meier-estimering

Figur 15: Kaplan-Meier-kurve for samlet overlevelse for hver behandlingsarm hos patienter med PD-L1-ekspression (TPS ≥ 50%) i KEYNOTE-040

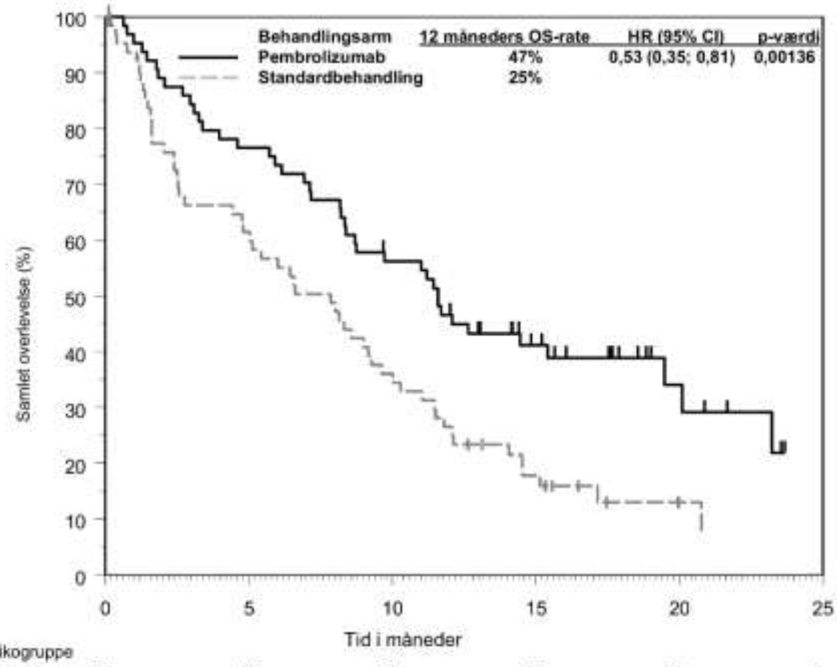

Antal i risikogruppe						
Pembrolizumab:	64	49	35	19	7	1
Standardbehandling:	65	38	22	9	2	0

Renalcellekarcinom

KEYNOTE-426: Kontrolleret studie af kombinationsbehandling hos behandlingsnaive RCC-patienter

Pembrolizumabs virkning i kombination med axitinib blev undersøgt i KEYNOTE-426, et randomiseret, åbent, aktivt kontrolleret multicenterstudie hos patienter med fremskredent RCC med en *clear cell*-komponent, uanset PD-L1-tumorekspressionsstatus og risikogruppekategorier i henhold til IMDC (*International Metastatic RCC Database Consortium*). Patienter med autoimmun sygdom eller lidelser, som krævede immunsuppression, blev ekskluderet fra studiet. Randomiseringen blev stratificeret ud fra risikokategorier (lav risiko *versus* middel risiko *versus* høj risiko) og geografisk område (Nordamerika *versus* Vesteuropa *versus* "resten af verden"). Patienterne blev randomiseret i forholdet 1:1 til en af følgende behandlingsarme:

- pembrolizumab 200 mg intravenøst hver 3. uge i kombination med axitinib 5 mg oralt, to gange dagligt. Hos patienter, som tålte axitinib 5 mg to gange dagligt i 2 konsekutive behandlingsserier (dvs. 6 uger) uden > grad 2 behandlingsrelaterede bivirkninger ved axitinib og som havde et velkontrolleret blodtryk på ≤ 150/90 mm Hg, var det tilladt at øge dosis af axitinib til 7 mg to gange dagligt. Det var tilladt at øge dosis af axitinib til 10 mg to gange dagligt ud fra de samme kriterier. Axitinib kunne pauseres eller reduceres til 3 mg to gange dagligt og efterfølgende til 2 mg to gange dagligt med henblik på at behandle toksicitet.
- sunitinib 50 mg oralt, en gang dagligt i 4 uger og herefter stop af behandling i 2 uger.

Behandlingen med pembrolizumab og axitinib fortsatte indtil sygdomsprogression defineret i henhold til RECIST v1.1 baseret på BICR-vurdering eller bekræftet af investigator, uacceptabel toksicitet, eller for for pembrolizumab, i højst 24 måneder. Administration af pembrolizumab og axitinib var tilladt efter sygdomsprogression i henhold til RECIST, hvis patienten var klinisk stabil, og investigator mente, at patienten havde en klinisk fordel. Der blev foretaget vurdering af tumorstatus ved *baseline*, efter

58

randomiseringen i uge 12, herefter hver 6. uge indtil uge 54, og herefter hver 12. uge. Kemiske og hæmatologiske laboratorietests blev udført ved hver behandlingsserie.

Der blev randomiseret i alt 861 patienter. Karakteristika for studiepopulationen var: medianalder på 62 år (interval: 26 til 90); 38% i alderen 65 år eller ældre; 73% mænd; 79% kaukasere og 16% asiatere; 80% havde en Karnofsky Performance-Score (KPS) på 90-100, og 20% havde en KPS på 70-80; patientfordelingen ifølge IMDC-risikokategorier var 31% lav risiko, 56% middel risiko og 13% høj risiko.

De primære resultatmål var OS og PFS (baseret på BICR-vurdering i henhold til RECIST 1.1). De sekundære resultatmål var ORR og responsvarighed baseret på BICR-vurdering i henhold til RECIST 1.1. Den mediane opfølgningstid var 12,8 måneder (interval: 0,1 til 21,5 måneder). I tabel 28 opsummeres de vigtigste effektresultater fra den præspecificerede interimanalyse. Kaplan-Meier-kurverne for OS og PFS baseret på yderligere fire måneders opfølgning er vist i figur 16 og 17.

Tabel 28: Effektresultater i KEYNOTE-426

Endepunkt	Pembrolizumab Axitinib n=432	Sunitinib n=429
**OS**		
Antal hændelser (%)	59 (14%)	97 (23%)
Median i måneder (95% CI)	Ikke nået (NA; NA)	Ikke nået (NA; NA)
HR[*] (95% CI)	0,53 (0,38; 0,74)	
p-værdi[†]	0,00005	
**PFS[‡]**		
Antal hændelser (%)	183 (42%)	213 (50%)
Median i måneder (95% CI)	15,1 (12,6; 17,7)	11,0 (8,7; 12,5)
HR[*] (95% CI)	0,69 (0,56; 0,84)	
p-værdi[†]	0,00012	
**Objektiv responsrate**		
ORR[§]% (95% CI)	59 (54; 64)	36 (31; 40)
Komplet respons	6%	2%
Partielt respons	53%	34%
p-værdi[¶]	< 0,0001	
**Responsvarighed**		
Median i måneder (interval)	Ikke nået (1,4+; 18,2+)	15,2 (1,1+; 15,4+)
Antal (%[#]) patienter med varighed ≥ 6 måneder	161 (88%)	84 (81%)
Antal (%[#]) patienter med varighed ≥ 12 måneder	58 (71%)	26 (62%)

* Baseret på den stratificerede Cox *proportional hazard*-model
† Baseret på stratificeret log rank-test
‡ BICR-vurdering i henhold til RECIST 1.1
§ Baseret på patienter med bedste objektive respons, der er bekræftet komplet eller partielt respons
¶ Baseret på Miettinen- og Nurminen-metoden stratificeret ud fra IMDC-risikogruppe og geografisk område
\# Baseret på Kaplan-Meier-estimering
NA = Foreligger ikke

Figur 16: Kaplan-Meier-kurve for samlet overlevelse i hver behandlingsarm i KEYNOTE-426 (*intent to treat*-population)*

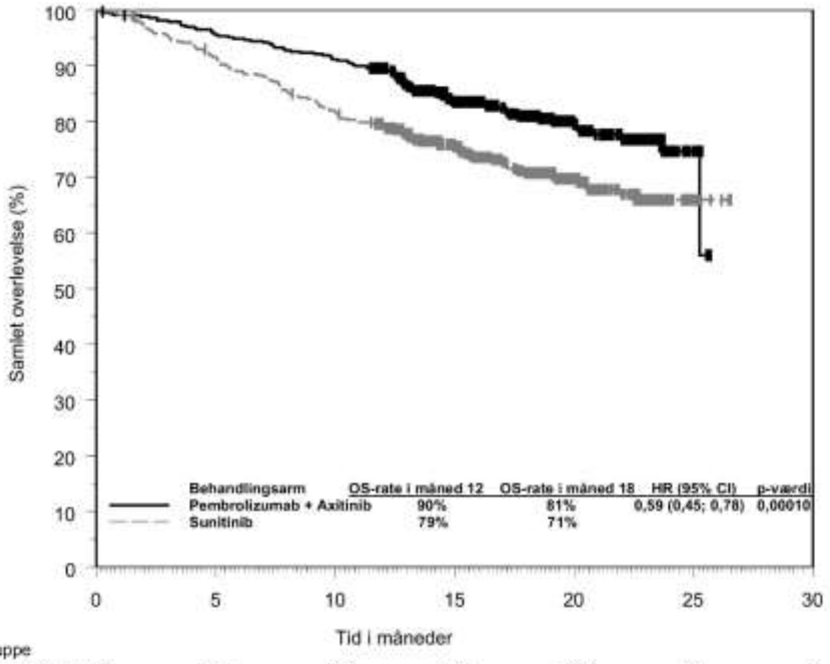

Antal i risikogruppe

Pembrolizumab + Axitinib:	432	411	392	275	133	9	0
Sunitinib:	429	389	346	230	111	6	0

*p-værdien er nominel. Se tabel 28 for p-værdien for inferentiel test af OS baseret på den præspecificerede interimanalyse, hvor der er nået statistisk signifikans.

60

Figur 17: Kaplan-Meier-kurve for progressionsfri overlevelse i hver behandlingsarm i KEYNOTE-426 (*intent to treat*-population)*

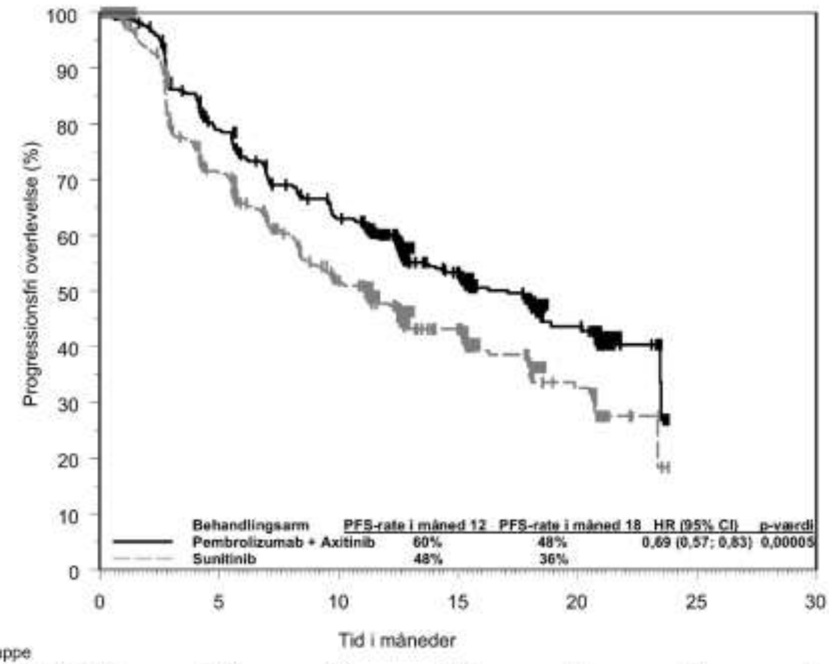

Antal i risikogruppe							
Pembrolizumab + Axitinib:	432	324	247	145	51	0	0
Sunitinib:	429	277	175	90	32	0	0

*p-værdien er nominel. Se tabel 28 for p-værdien for inferentiel test af PFS baseret på den præspecificerede interimanalyse, hvor der er nået statistisk signifikans.

Der blev udført subgruppeanalyser ud fra inklusion i KEYNOTE-426 hos patienter med PD-L1 CPS ≥ 1 [pembrolizumab/axitinib-kombinationen: n=243 (56%) *versus* sunitinib: n=254 (59%)]; CPS < 1 [pembrolizumab/axitinib-kombinationen: n=167 (39%) *versus* sunitinib: n=158 (37%)], og hos patienter med IMDC-risikokategorierne lav risiko [pembrolizumab/axitinib-kombinationen: n=138 (32%) *versus* sunitinib: n=131 (31%)]; middel risiko [pembrolizumab/axitinib-kombinationen: n=238 (55%) *versus* sunitinib: n=246 (57%)]; og høj risiko [pembrolizumab/axitinib-kombinationen: n=56 (13%) *versus* sunitinib: n=52 (12%)]. OS- og PFS-fordel sås uanset PD-L1-ekspressionsniveau. KEYNOTE-426-studiet var ikke designet til at vurdere virkningen i individuelle subgrupper. I tabel 29 opsummeres effektresultater ud fra IMDC-risikokategori fra den præspecificerede interimanalyse.

Tabel 29: Effektresultater i KEYNOTE-426 ud fra IMDC-risikokategori

Endepunkt	Pembrolizumab + Axitinib N = 432	Sunitinib N = 429	Pembrolizumab + Axitinib *versus* Sunitinib
**OS**	**OS-rate i måned 12, % (95% CI)**		**HR for OS (95% CI)**
Lav risiko	95,2 (89,6; 97,9)	93,8 (87,4; 97,0)	0,64 (0,24; 1,68)
Middel risiko	92,1 (84,7; 96,0)	76,7 (70,6; 81,8)	0,53 (0,35; 0,82)
Høj risiko	70,3 (56,1; 80,7)	45,2 (30,0; 59,3)	0,43 (0,23; 0,81)

61

PFS	Median (95% CI), måneder		HR for PFS (95% CI)
Lav risiko	17,7 (15,2; NA)	12,7 (11,5; NA)	0,81 (0,53; 1,24)
Middel risiko	14,5 (12,4; 18,0)	9,5 (8,0; 12,5)	0,69 (0,53; 0,90)
Høj risiko	4,9 (2,9; 12,4)	2,9 (2,7; 4,2)	0,58 (0,35; 0,94)
**Bekræftet ORR**	**% (95% CI)**		**ORR-forskel, % (95% CI)**
Lav risiko	66,7 (58,1; 74,5)	49,6 (40,8; 58,5)	17,0 (5,3; 28,4)
Middel risiko	59,2 (52,7; 65,5)	33,7 (27,9; 40,0)	25,5 (16,7; 33,9)
Høj risiko	41,1 (28,1; 55,0)	9,6 (3,2; 21,0)	31,5 (15,7; 46,2)

NA = Foreligger ikke

En opdateret OS-analyse blev udført, når patienterne havde en median opfølgingstid på 16,6 måneder (interval: 0,1 til 26,3 måneder). På tidspunktet for denne analyse var HR i den samlede population (95% CI): 0,59 (0,45; 0,78) med 84/432 (19,4%) hændelser i armen med kombinationsbehandling og 122/429 (28,4%) hændelser i sunitinib-armen. OS-raten i måned 12 var 89,5% (95% CI: 86,2; 92,1) for pembrolizumab i kombination med axitinib og 78,8% (95% CI: 74,7; 82,4) for sunitinib. OS-raten i måned 18 var 81,0% (95% CI: 76,7; 84,6) for pembrolizumab i kombination med axitinib og 70,7% (95% CI: 65,8; 75,1) for sunitinib. For IMDC-risikokategorien: HR for OS for risikogruppen med lav risiko var 0,94 (95% CI: 0,43; 2,07), HR for OS for risikogruppen med middel risiko var 0,52 (95% CI: 0,36; 0,75) og HR for OS for risikogruppen med høj risiko var 0,50 (95% CI: 0,29; 0,87).

Pædiatrisk population
I KEYNOTE-051 blev pembrolizumab 2 mg/kg hver 3. uge administreret til 154 pædiatriske patienter (60 børn i alderen 6 måneder til under 12 år og 94 unge i alderen 12 år til 18 år) med fremskredent melanom eller PD-L1-positive fremskredne, recidiverende eller refraktære solide tumorer eller lymfomer. Alle patienter fik pembrolizumab med en median på 3 doser (interval: 1-35 doser), hvor 130 patienter (84,4%) fik 2 eller flere doser pembrolizumab. Deltagerne blev inkluderet på tværs af 28 tumortyper ud fra primær diagnose. De hyppigste tumortyper ud fra histologi var Hodgkins lymfom (11,7%), glioblastom multiforme (9,1%), neuroblastom (6,5%), osteosarkom (6,5%) og melanom (5,2%). Ud af 154 patienter blev 134 inkluderet med solide tumorer, 18 med Hodgkins lymfom og 2 med andre lymfomer. Hos patienter med solide tumorer og andre lymfomer var ORR 5,9%, ingen patienter havde komplet respons og 8 patienter (5,9%) havde partielt respons. I populationen med Hodgkins lymfom var ORR 50,0%, 2 patienter (11,1%) havde komplet respons og 7 patienter (38,9%) havde partielt respons.

Det Europæiske Lægemiddelagentur har udsat forpligtelsen til at fremlægge resultaterne af studier med pembrolizumab i en eller flere undergrupper af den pædiatriske population ved behandling af Hodgkins lymfom (se pkt. 4.2 for oplysninger om pædiatrisk anvendelse).

5.2 Farmakokinetiske egenskaber

Pembrolizumabs farmakokinetik blev undersøgt hos 2.993 patienter med metastatisk eller inoperabelt melanom, NSCLC eller karcinom, som har fået doser i intervallet 1 til 10 mg/kg hver 2. uge, 2 til 10 mg/kg hver 3. uge eller 200 mg hver 3. uge.

Absorption
Pembrolizumab administreres intravenøst, og biotilgængeligheden er derfor øjeblikkelig og fuldstændig.

Fordeling
I overensstemmelse med en begrænset ekstravaskulær fordeling er det gennemsnitlige fordelingsvolumen for pembrolizumab ved *steady state* lille (~6,0 liter; variationskoefficient (CV) 20%). Som forventet for et antistof binder pembrolizumab ikke specifikt til plasmaproteiner.

Biotransformation
Pembrolizumab nedbrydes via non-specifikke metaboliseringsveje; metaboliseringen bidrager ikke til pembrolizumabs *clearance*.

Elimination
Pembrolizumabs *clearance* er cirka 23% lavere (geometrisk gennemsnit, 195 ml/dag [CV%: 40%]) efter at have opnået maksimal ændring ved *steady state* sammenlignet med den første dosis (252 ml/dag [CV%: 37%]). Dette fald i *clearance* over tid anses ikke for at være klinisk betydningsfuldt. Den geometriske gennemsnitsværdi (CV%) for den terminale halveringstid er 22 dage (32%) ved *steady state*.

Linearitet/non-linearitet
Eksponeringen for pembrolizumab udtrykt ved *peak*-koncentration (C_{max}) eller areal under kurven for plasmakoncentration over tid (AUC) steg dosisproportionalt inden for det terapeutiske dosisinterval. *Steady state*-koncentrationer af pembrolizumab blev nået efter 16 uger med gentagen dosering med et regime med administration hver 3. uge, og den systemiske akkumulering var cirka 2,1 gange. Den mediane dalkoncentration (C_{min}) ved *steady state* var ca. 22 µg/ml ved en dosis på 2 mg/kg hver 3. uge og 29 µg/ml ved en dosis på 200 mg hver 3. uge. Det mediane areal under kurven for koncentration over tid ved *steady state* over 3 uger ($AUC_{0\text{-}3uger}$) var 794 µg·dag/ml ved en dosis på 2 mg/kg hver 3. uge og 1.053 µg·dag/ml ved en dosis på 200 mg hver 3. uge.

Efter administration af pembrolizumab 200 mg hver 3. uge til patienter med cHL var den observerede mediane C_{min} ved *steady state* op til 40% højere end den, som blev observeret ved andre tumortyper, der blev behandlet med samme dosis. Intervallerne for dalkoncentrationerne er imidlertid sammenlignelige. Der er ingen væsentlig forskel i median C_{max} mellem cHL og andre tumortyper. Baseret på tilgængelige sikkerhedsdata for cHL og andre tumortyper er disse forskelle ikke klinisk relevante.

Særlige populationer
Indvirkningen af forskellige kovariater på pembrolizumabs farmakokinetik blev vurderet i farmakokinetiske populationsanalyser. Følgende faktorer havde ingen klinisk betydningsfuld indvirkning på pembrolizumabs *clearance*: alder (interval: 15-94 år), køn, race, let eller moderat nedsat nyrefunktion, let nedsat leverfunktion og tumorbyrde. Forholdet mellem kropsvægt og *clearance* understøtter dosering med enten fast dosis eller dosis baseret på kropsvægt til at give tilstrækkelig og sammenlignelig eksponeringskontrol. Ved vægtbaseret dosering på 2 mg/kg hver 3. uge hos pædiatriske patienter (2 til 17 år) er koncentrationen af pembrolizumab sammenlignelig med koncentrationen hos voksne ved samme dosis.

Nedsat nyrefunktion
Effekten af nedsat nyrefunktion på pembrolizumabs *clearance* blev vurderet ud fra farmakokinetiske populationsanalyser, hvor patienter med let eller moderat nedsat nyrefunktion blev sammenlignet med patienter med normal nyrefunktion. Der blev ikke påvist klinisk betydningsfulde forskelle i pembrolizumabs *clearance* mellem patienter med let eller moderat nedsat nyrefunktion og patienter med normal nyrefunktion. Pembrolizumab er ikke undersøgt hos patienter med svært nedsat nyrefunktion.

Nedsat leverfunktion
Effekten af nedsat leverfunktion på pembrolizumabs *clearance* blev vurderet ud fra farmakokinetiske populationsanalyser, hvor patienter med let nedsat leverfunktion (defineret i henhold til *US National Cancer Institute* (NCI) kriterier for leverinsufficiens) blev sammenlignet med patienter med normal leverfunktion. Der blev ikke påvist klinisk betydningsfulde forskelle i pembrolizumabs *clearance* mellem patienter med let nedsat leverfunktion og patienter med normal leverfunktion. Pembrolizumab er ikke undersøgt hos patienter med moderat eller svært nedsat leverfunktion (se pkt. 4.2).

5.3 Non-kliniske sikkerhedsdata

Pembrolizumabs sikkerhed blev vurderet i toksicitetsstudier med gentagne doser af 1 måned og 6 måneders varighed, hvor cynomolgusaber fik administreret intravenøse doser på 6, 40 eller 200 mg/kg en gang om ugen i studiet af 1 måneds varighed og en gang hver anden uge i studiet af 6 måneders varighed, efterfulgt af en behandlingsfri periode på 4 måneder. Der blev ikke observeret fund af

toksikologisk betydning, og NOAEL-værdien (*No Observed Adverse Effect Level*) i begge studier var ≥ 200 mg/kg, hvilket gav multiple eksponeringer på 19 og 94 gange eksponeringen hos mennesker ved doser på henholdsvis 10 og 2 mg/kg. Den multiple eksponering mellem NOAEL og en human dosis på 200 mg var 74.

Der er ikke udført reproduktionsstudier med pembrolizumab hos dyr. Det menes, at PD-1/PD-L1-signalvejen er involveret i at opretholde tolerancen over for fostret under graviditeten. I dyremodeller med drægtige mus er det blevet påvist, at blokering af PD-L1-signalet kan påvirke tolerancen for fostret og medføre et stigende antal fostertab.

Der er ikke udført fertilitetsstudier med pembrolizumab hos dyr. Der var ingen betydningsfuld indvirkning på forplantningsorganerne hos han- og hunaber i toksicitetsstudier af 1 måned og 6 måneders varighed med gentagne doser. Mange dyr i disse studier var imidlertid ikke kønsmodne.

6. FARMACEUTISKE OPLYSNINGER

6.1 Hjælpestoffer

L-histidin
L-histidin-hydrochloridmonohydrat
Saccharose
Polysorbat 80

6.2 Uforligeligheder

Da der ikke foreligger studier af eventuelle uforligeligheder, må dette lægemiddel ikke blandes med andre lægemidler end dem, der er anført under pkt. 6.6.

6.3 Opbevaringstid

Uåbnet hætteglas
3 år.

Efter rekonstitution
Ud fra en mikrobiologisk synsvinkel skal det rekonstituerede koncentrat eller den fortyndede infusionsvæske anvendes straks. Den rekonstituerede eller fortyndede opløsning må ikke nedfryses. Hvis KEYTRUDA ikke anvendes straks, er der påvist kemisk og fysisk stabilitet i 96 timer ved 2 °C-8 °C. Den samlede opbevaringstid på 96 timer efter rekonstitution kan inkludere opbevaring i op til 6 timer ved stuetemperatur (ved eller under 25 °C). Hætteglassene og/eller de intravenøse infusionsposer skal opnå stuetemperatur inden brug, hvis de har været opbevaret i køleskab.

6.4 Særlige opbevaringsforhold

Opbevares i køleskab (2 °C – 8 °C).

Opbevaringsforhold efter rekonstitution eller fortynding af lægemidlet, se pkt. 6.3.

6.5 Emballagetype og pakningsstørrelser

15 ml hætteglas (type I-glas) med en grå brombutylprop og en aluminiumshætte med et avocadofarvet flip-off-låg, der indeholder 50 mg pembrolizumab.

Hver karton indeholder et hætteglas.

6.6 Regler for bortskaffelse og anden håndtering

Klargøring og administration

- Før rekonstitution kan hætteglasset med frysetørret pulver (lyofilisat) opbevares uden for køleskab (ved temperaturer på eller under 25 °C) i op til 24 timer.
- Tilføj 2,3 ml vand til injektionsvæsker aseptisk for at få en opløsning af KEYTRUDA på 25 mg/ml (pH: 5,2-5,8). Hvert hætteglas indeholder et overskud på 10 mg (0,4 ml) for at sikre, at der kan udtrækkes 50 mg KEYTRUDA pr. hætteglas. Efter rekonstitution indeholder 1 ml koncentrat 25 mg pembrolizumab.
- Tilsæt vandet ned langs siden af hætteglasset og ikke direkte ned i det frysetørrede pulver for at undgå skumdannelse.
- Hvirvl langsomt hætteglasset rundt således, at det frysetørrede pulver bliver opløst. Lad der gå 5 minutter, så eventuelle bobler forsvinder. Hætteglasset må ikke rystes.
- Parenterale lægemidler skal inspiceres visuelt for partikler og misfarvning inden administration. Det rekonstituerede KEYTRUDA-koncentrat er en klar til let opaliserende og farveløs til lysegul opløsning. Hætteglasset skal kasseres, hvis der observeres synlige partikler.
- Træk det påkrævede volumen (op til 2 ml (50 mg)) KEYTRUDA op og overfør det til en intravenøs infusionspose, der indeholder natriumchlorid 9 mg/ml (0,9%) eller glucose 50 mg/ml (5%) for at fremstille den færdige infusionsvæske med en endelig koncentration på 1 til 10 mg/ml. Bland den færdige infusionsvæske ved forsigtigt at vende infusionsposen.
- Ud fra en mikrobiologisk synsvinkel skal det rekonstituerede koncentrat eller den fortyndede infusionsvæske anvendes straks. Den rekonstituerede eller fortyndede opløsning må ikke nedfryses. Hvis KEYTRUDA ikke anvendes straks, er der påvist kemisk og fysisk stabilitet i 96 timer ved 2 °C-8 °C. Den samlede opbevaringstid på 96 timer efter rekonstitution kan inkludere opbevaring i op til 6 timer ved stuetemperatur (ved eller under 25 °C). Hætteglassene og/eller de intravenøse infusionsposer skal opnå stuetemperatur inden brug, hvis de har været opbevaret i køleskab. Gennemsigtige til hvide proteinholdige partikler kan forekomme i den fortyndede infusionsvæske. Administrer infusionsvæsken intravenøst over en periode på 30 minutter med et infusionssæt med et *in-line* eller *add-on* sterilt, ikke-pyrogent, minimalt proteinbindende filter (porestørrelse 0,2 til 5 µm).
- KEYTRUDA må ikke administreres sammen med andre lægemidler i samme infusionsslange.
- KEYTRUDA er kun til engangsbrug. Den ubrugte rest i hætteglasset bortskaffes.

Ikke anvendt lægemiddel samt affald heraf skal bortskaffes i henhold til lokale retningslinjer.

7. INDEHAVER AF MARKEDSFØRINGSTILLADELSEN

Merck Sharp & Dohme B.V.
Waarderweg 39
2031 BN Haarlem
Holland

8. MARKEDSFØRINGSTILLADELSESNUMMER (-NUMRE)

EU/1/15/1024/001

9. DATO FOR FØRSTE MARKEDSFØRINGSTILLADELSE/FORNYELSE AF TILLADELSEN

Dato for første markedsføringstilladelse: 17. juli 2015
Dato for seneste fornyelse: 24. marts 2020

10. DATO FOR ÆNDRING AF TEKSTEN

Yderligere oplysninger om dette lægemiddel findes på Det Europæiske Lægemiddelagenturs hjemmeside http://www.ema.europa.eu.

1. LÆGEMIDLETS NAVN

KEYTRUDA 25 mg/ml koncentrat til infusionsvæske, opløsning.

2. KVALITATIV OG KVANTITATIV SAMMENSÆTNING

Et hætteglas med 4 ml koncentrat indeholder 100 mg pembrolizumab.
Hver ml koncentrat indeholder 25 mg pembrolizumab.

Pembrolizumab er et humaniseret monoklonalt anti-*programmed cell death*-1 (PD-1)-antistof (IgG4/kappa-isotype med en stabiliserende forandring af sekvensen i Fc-regionen), som er fremstillet ved rekombinant dna-teknologi i ovarieceller fra kinesiske hamstre.

Alle hjælpestoffer er anført under pkt. 6.1.

3. LÆGEMIDDELFORM

Koncentrat til infusionsvæske, opløsning.

Klar til let opaliserende, farveløs til lysegul opløsning med pH 5,2 - 5,8.

4. KLINISKE OPLYSNINGER

4.1 Terapeutiske indikationer

Melanom
KEYTRUDA som monoterapi er indiceret til behandling af fremskredent (inoperabelt eller metastatisk) melanom hos voksne.

KEYTRUDA som monoterapi er indiceret til adjuverende behandling af voksne med stadie III-melanom og lymfeknudeinvolvering, som har fået foretaget komplet resektion (se pkt. 5.1).

Ikke-småcellet lungecancer (NSCLC)
KEYTRUDA som monoterapi er indiceret til førstelinjebehandling af metastatisk ikke-småcellet lungecancer hos voksne, hvis tumorer udtrykker PD-L1 med *tumour proportion score* (TPS) ≥ 50% uden EGFR- eller ALK-positive mutationer i tumor.

KEYTRUDA, i kombination med pemetrexed og platinbaseret kemoterapi, er indiceret til førstelinjebehandling af metastatisk ikke-planocellulær ikke-småcellet lungecancer hos voksne uden EGFR- eller ALK-positive mutationer i tumorer.

KEYTRUDA, i kombination med carboplatin og enten paclitaxel eller nab-paclitaxel, er indiceret til førstelinjebehandling af metastatisk planocellulær ikke-småcellet lungecancer hos voksne.

KEYTRUDA som monoterapi er indiceret til behandling af lokalt fremskredent eller metastatisk ikke-småcellet lungecancer hos voksne efter tidligere behandling med minimum én kemoterapi, og hvis tumorer udtrykker PD-L1 med TPS ≥ 1%. Patienter med EGFR- eller ALK-positive mutationer i tumor bør også have været i targeteret behandling inden behandling med KEYTRUDA.

Klassisk Hodgkins lymfom (cHL)
KEYTRUDA som monoterapi er indiceret til behandling af recidiverende eller refraktært klassisk Hodgkins lymfom hos voksne, som har oplevet svigt af autolog stamcelletransplantation (ASCT) og svigt af behandling med brentuximab vedotin (BV), eller som er uegnede til transplantation og har oplevet svigt af behandling med BV.

Urotelialt karcinom
KEYTRUDA som monoterapi er indiceret til behandling af lokalt fremskredent eller metastatisk urotelialt karcinom hos voksne, som tidligere har fået platinbaseret kemoterapi (se pkt. 5.1).

KEYTRUDA som monoterapi er indiceret til behandling af lokalt fremskredent eller metastatisk urotelialt karcinom hos voksne, som er uegnede til cisplatinbaseret kemoterapi, og hvis tumorer udtrykker PD-L1 med en kombineret positiv score (CPS) ≥ 10 (se pkt. 5.1).

Planocellulært hoved-hals karcinom (HNSCC)
KEYTRUDA som monoterapi eller i kombination med platinbaseret kemoterapi og 5-fluorouracil (5-FU) er indiceret til førstelinjebehandling af metastatisk eller inoperabelt recidiverende planocellulært hoved-hals karcinom hos voksne, hvis tumorer udtrykker PD-L1 med CPS ≥ 1 (se pkt. 5.1).

KEYTRUDA som monoterapi er indiceret til behandling af recidiverende eller metastatisk planocellulært hoved-hals karcinom hos voksne, hvis tumorer udtrykker PD-L1 med TPS ≥ 50% og med sygdomsprogression under eller efter platinbaseret kemoterapi (se pkt. 5.1).

Renalcellekarcinom (RCC)
KEYTRUDA, i kombination med axitinib, er indiceret til førstelinjebehandling af fremskredent renalcellekarcinom hos voksne (se pkt. 5.1).

4.2 Dosering og administration

Behandlingen skal initieres og superviseres af læger med erfaring i behandling af cancer.

PD-L1-test af patienter med NSCLC, urotelialt karcinom eller HNSCC
Ved behandling med KEYTRUDA som monoterapi anbefales det at teste for PD-L1-tumorekspression ved hjælp af en valideret test til at udvælge patienter med NSCLC eller tidligere ubehandlet urotelialt karcinom (se pkt. 4.1, 4.4, 4.8 og 5.1).

Patienter med HNSCC skal udvælges til behandling med KEYTRUDA som monoterapi eller i kombination med platinbaseret kemoterapi og 5-fluorouracil (5-FU) på basis af PD-L1-tumorekspression, som er bekræftet af en valideret test (se pkt. 4.1, 4.4, 4.8 og 5.1).

Dosering
Den anbefalede dosis af KEYTRUDA som monoterapi er enten 200 mg hver 3. uge eller 400 mg hver 6. uge administreret som intravenøs infusion over 30 minutter.

Den anbefalede dosis af KEYTRUDA som en del af kombinationsbehandling er 200 mg hver 3. uge administreret som intravenøs infusion over 30 minutter.

Patienterne skal behandles med KEYTRUDA indtil sygdomsprogression eller uacceptabel toksicitet. Atypisk respons (pseudoprogression) er observeret (initial forbigående stigning i tumorstørrelse eller små nye læsioner inden for de første få måneder efterfulgt af tumorregression). Det anbefales at fortsætte behandlingen hos klinisk stabile patienter med initiale tegn på sygdomsprogression, indtil sygdomsprogression er bekræftet.

Ved adjuverende behandling af melanom skal KEYTRUDA administreres indtil sygdomsrecidiv, uacceptabel toksicitet eller i en periode på op til 1 år.

Pausering eller seponering af behandling (se også pkt. 4.4)
Dosisreduktion af KEYTRUDA anbefales ikke. Behandlingen med KEYTRUDA skal pauseres eller seponeres for at håndtere bivirkninger som beskrevet i tabel 1.

Tabel 1: Anbefalede ændringer i KEYTRUDA-behandling

Immunrelaterede bivirkninger	Sværhedsgrad	Ændring af behandling
Pneumonitis	Grad 2	Pauser behandlingen indtil bivirkningerne bedres til grad 0-1*
	Grad 3 eller 4 eller tilbagevendende grad 2	Seponer behandlingen permanent
Colitis	Grad 2 eller 3	Pauser behandlingen indtil bivirkningerne bedres til grad 0-1*
	Grad 4 eller tilbagevendende grad 3	Seponer behandlingen permanent
Nefritis	Grad 2 med kreatinin > 1,5 til ≤ 3 gange øvre normale grænseværdi (ULN)	Pauser behandlingen indtil bivirkningerne bedres til grad 0-1*
	Grad ≥ 3 med kreatinin > 3 gange ULN	Seponer behandlingen permanent
Endokrinopatier	Grad 2 binyrebarkinsufficiens og hypofysitis	Pauser behandlingen indtil bivirkningen er kontrolleret med hormonsubstitutionsbehandling
	Grad 3 eller 4 binyrebarkinsufficiens eller symptomatisk hypofysitis<br><br>Type 1-diabetes associeret med hyperglykæmi grad ≥ 3 (glucose > 250 mg/dl eller > 13,9 mmol/l) eller associeret med ketoacidose<br><br>Hypertyroidisme grad ≥ 3	Pauser behandlingen indtil bivirkningerne bedres til grad 0-1*<br><br>Det kan overvejes at fortsætte behandlingen med pembrolizumab efter langsom udtrapning af eventuel nødvendig kortikosteroidbehandling hos patienter med endokrinopatier grad 3 eller grad 4, som bedres til grad 2 eller lavere med hormonsubstitutionsbehandling, hvis det er indiceret. Ellers skal behandlingen seponeres.
	Hypotyroidisme	Hypotyroidisme kan behandles med substitution uden afbrydelse af behandlingen.

Hepatitis<br><br>**BEMÆRK:** se doseringsvejledningen i hht. denne tabel for RCC-patienter i behandling med **pembrolizumab i kombination med axitinib** og med forhøjede leverenzymer.	Grad 2 med aspartataminotransferase (ASAT) eller alaninaminotransferase (ALAT) > 3-5 gange ULN eller total-bilirubin > 1,5-3 gange ULN	Pauser behandlingen indtil bivirkningerne bedres til grad 0-1*
	Grad ≥ 3 med ASAT eller ALAT > 5 gange ULN eller total-bilirubin > 3 gange ULN	Seponer behandlingen permanent
	Levermetastaser med grad 2 forhøjelse af ASAT eller ALAT, hepatitis med ASAT- eller ALAT-stigning på ≥ 50% i forhold til *baseline*, og som varer ≥ 1 uge	Seponer behandlingen permanent
Hudreaktioner	Grad 3 eller mistanke om Stevens-Johnsons syndrom (SJS) eller toksisk epidermal nekrolyse (TEN)	Pauser behandlingen indtil bivirkningerne bedres til grad 0-1*
	Grad 4 eller bekræftet SJS eller TEN	Seponer behandlingen permanent
**Andre immunrelaterede bivirkninger**	Baseret på sværhedsgrad og type af bivirkning (grad 2 eller grad 3)<br><br>Grad 3 eller 4 myokarditis<br>Grad 3 eller 4 encephalitis<br>Grad 3 eller 4 Guillain-Barrés syndrom<br><br>Grad 4 eller tilbagevendende grad 3	Pauser behandlingen indtil bivirkningerne bedres til grad 0-1*<br><br>Seponer behandlingen permanent<br><br>Seponer behandlingen permanent
**Infusionsreaktioner**	Grad 3 eller 4	Seponer behandlingen permanent

Bemærk: Toksicitetsgrader er angivet i overensstemmelse med *National Cancer Institute Common Terminology Criteria for Adverse Events Version 4.0* (NCI-CTCAE v.4).

* Hvis behandlingsrelateret toksicitet ikke bedres til grad 0-1 inden for 12 uger efter den sidste dosis KEYTRUDA, eller hvis kortikosteroiddoseringen ikke kan reduceres til ≤ 10 mg prednison eller tilsvarende pr. dag inden for 12 uger, skal KEYTRUDA seponeres permanent.

Sikkerheden ved re-initiering af behandling med pembrolizumab hos patienter, der tidligere har oplevet immunrelateret myokarditis, er ikke kendt.

KEYTRUDA, som monoterapi eller som kombinationsbehandling, skal seponeres permanent ved grad 4 eller tilbagevendende grad 3 immunrelaterede bivirkninger, medmindre det er specificeret anderledes i tabel 1.

Ved grad 4 hæmatologisk toksicitet, kun hos patienter med cHL, skal KEYTRUDA pauseres indtil bivirkningerne bedres til grad 0-1.

KEYTRUDA i kombination med axitinib ved RCC
Se produktresuméet for axitinib for oplysninger vedrørende dosering af axitinib for RCC-patienter, der behandles med KEYTRUDA i kombination med axitinib. Når axitinib anvendes i kombination med pembrolizumab kan øgning af axitinib-dosis til over initialdosis på 5 mg overvejes i intervaller på seks uger eller længere (se pkt. 5.1).

Ved forhøjede leverenzymer hos RCC-patienter, der behandles med KEYTRUDA i kombination med axitinib:

- Hvis ALAT eller ASAT er ≥ 3 gange ULN, men < 10 gange ULN uden samtidig total-bilirubin-værdi ≥ 2 gange ULN, skal både KEYTRUDA og axitinib pauseres, indtil disse bivirkninger bedres til grad 0-1. Behandling med kortikosteroider kan overvejes. Genoptagelse af behandling (*rechallenge*) med et enkelt lægemiddel eller sekventiel genoptagelse af behandlingen (*sequential rechallenge*) med begge lægemidler efter bedring kan overvejes. Hvis behandlingen med axitinib genoptages, kan dosisreduktion i henhold til produktresuméet for axitinib overvejes.
- Hvis ALAT eller ASAT er ≥ 10 gange ULN eller > 3 gange ULN med samtidig total-bilirubin-værdi ≥ 2 gange ULN, skal både KEYTRUDA og axitinib seponeres permanent, og behandling med kortikosteroider kan overvejes.

Patienter, som bliver behandlet med KEYTRUDA, skal have udleveret et patientinformationskort og informeres om risiciene ved KEYTRUDA (se også indlægssedlen).

Særlige populationer
Ældre
Dosisjustering er ikke nødvendig hos patienter ≥ 65 år (se pkt. 5.1). Data fra patienter ≥ 65 år med cHL er for begrænsede til at kunne drage konklusioner (se pkt. 5.1). Data fra pembrolizumab som monoterapi hos patienter med resekteret stadie III-melanom, fra pembrolizumab i kombination med axitinib hos patienter med fremskredent RCC, fra kombinationsbehandling med kemoterapi hos patienter med metastatisk NSCLC, og fra pembrolizumab (med eller uden kemoterapi) hos patienter i førstelinjebehandling for metastatisk eller inoperabelt recidiverende HNSCC ≥ 75 år er begrænsede (se pkt. 4.4 og 5.1).

Nedsat nyrefunktion
Dosisjustering er ikke nødvendig hos patienter med let eller moderat nedsat nyrefunktion. KEYTRUDA er ikke undersøgt hos patienter med svært nedsat nyrefunktion (se pkt. 4.4 og 5.2).

Nedsat leverfunktion
Dosisjustering er ikke nødvendig hos patienter med let nedsat leverfunktion. KEYTRUDA er ikke undersøgt hos patienter med moderat eller svært nedsat leverfunktion (se pkt. 4.4 og 5.2).

Pædiatrisk population
KEYTRUDAs sikkerhed og virkning hos børn under 18 år er endnu ikke klarlagt. De foreliggende data er beskrevet i pkt. 4.8, 5.1 og 5.2.

Administration
KEYTRUDA er til intravenøs anvendelse. Det skal administreres som en infusion over 30 minutter. KEYTRUDA må ikke administreres som intravenøs push- eller bolus-injektion.

Se produktresuméet for de samtidigt administrerede lægemidler ved anvendelse i kombinationsbehandling. Når KEYTRUDA administreres som del af en kombinationsbehandling med intravenøs kemoterapi, skal KEYTRUDA administreres først.

For instruktioner om fortynding af lægemidlet før administration, se pkt. 6.6.

4.3 Kontraindikationer

Overfølsomhed over for det aktive stof eller over for et eller flere af hjælpestofferne anført i pkt. 6.1.

4.4 Særlige advarsler og forsigtighedsregler vedrørende brugen

For at forbedre sporbarheden af biologiske lægemidler skal det administrerede produkts navn og batchnummer tydeligt registreres.

Vurdering af PD-L1-status
Ved vurdering af PD-L1-status af tumoren er det vigtigt, at der vælges en valideret og robust metode for at minimere forekomsten af falsk negative eller falsk positive resultater.

Immunrelaterede bivirkninger
Immunrelaterede bivirkninger, inklusive svære og dødelige tilfælde, er forekommet hos patienter, der fik pembrolizumab. De fleste immunrelaterede bivirkninger, der forekom under behandlingen med pembrolizumab, var reversible og blev håndteret ved hjælp af afbrydelse af behandlingen med pembrolizumab, behandling med kortikosteroider og/eller understøttende pleje. Immunrelaterede bivirkninger er også observeret efter den sidste dosis af pembrolizumab. Immunrelaterede bivirkninger, der påvirker mere end et organsystem i kroppen, kan forekomme på samme tid.

Ved mistanke om immunrelaterede bivirkninger skal det sikres, at der foretages tilstrækkelig udredning for at bekræfte ætiologi eller udelukke andre årsager. Baseret på bivirkningens sværhedsgrad skal behandlingen med pembrolizumab pauseres og kortikosteroider administreres. Ved bedring til grad ≤ 1 påbegyndes langsom udtrapning af kortikosteroider over en periode på mindst 1 måned. På basis af begrænsede data fra kliniske studier med patienter, hvis immunrelaterede bivirkninger ikke kunne kontrolleres med kortikosteroider, kan det overvejes at administrere andre systemiske immunsuppressiva.

Behandlingen med pembrolizumab kan genoptages inden for 12 uger efter den sidste dosis af KEYTRUDA, hvis bivirkningen bedres til grad ≤ 1, og kortikosteroiddosis er reduceret til ≤ 10 mg prednison dagligt eller en dosis ækvivalent hermed.

Pembrolizumab skal seponeres permanent ved en hvilken som helst immunrelateret bivirkning af grad 3, som recidiverer, og ved alle immunrelaterede bivirkninger af toksicitetsgrad 4, bortset fra endokrinopatier, som kontrolleres med hormonsubstitutionsbehandling (se pkt. 4.2 og 4.8).

Immunrelateret pneumonitis
Pneumonitis er rapporteret hos patienter, der fik pembrolizumab (se pkt. 4.8). Patienterne skal monitoreres for tegn og symptomer på pneumonitis. Mistanke om pneumonitis skal bekræftes ved en billeddiagnostisk udredning af patienterne, og andre årsager skal udelukkes. Kortikosteroider skal administreres ved bivirkninger grad ≥ 2 (initialdosis 1-2 mg/kg/dag prednison eller ækvivalent hermed efterfulgt af langsom udtrapning); pembrolizumab skal pauseres ved grad 2-pneumonitis og seponeres permanent ved grad 3-, grad 4- eller tilbagevendende grad 2-pneumonitis (se pkt. 4.2).

Immunrelateret colitis
Colitis er rapporteret hos patienter, der fik pembrolizumab (se pkt. 4.8). Patienterne skal monitoreres for tegn og symptomer på colitis, og andre årsager skal udelukkes. Kortikosteroider skal administreres ved bivirkninger grad ≥ 2 (initialdosis 1-2 mg/kg/dag prednison eller ækvivalent hermed efterfulgt af langsom udtrapning); pembrolizumab skal pauseres ved grad 2- eller grad 3-colitis og seponeres permanent ved grad 4- eller tilbagevendende grad 3-colitis (se pkt. 4.2). Den potentielle risiko for gastrointestinal perforation skal tages i betragning.

Immunrelateret hepatitis
Hepatitis er rapporteret hos patienter, der fik pembrolizumab (se pkt. 4.8). Patienterne skal monitoreres for ændringer i leverfunktionen (ved behandlingsstart, periodevist i løbet af behandlingen og som indiceret på basis af den kliniske vurdering) og for symptomer på hepatitis, og andre årsager skal udelukkes. Kortikosteroider skal administreres (initialdosis 0,5-1 mg/kg/dag [ved bivirkninger af grad 2] eller 1-2 mg/kg/dag [ved bivirkninger grad ≥ 3] prednison eller ækvivalent hermed efterfulgt af langsom udtrapning), og på basis af sværhedsgraden af leverenzymstigningen skal behandlingen med pembrolizumab pauseres eller seponeres (se pkt. 4.2).

Immunrelateret nefritis
Nefritis er rapporteret hos patienter, der fik pembrolizumab (se pkt. 4.8). Patienterne skal monitoreres for ændringer i nyrefunktionen, og andre årsager til nedsat nyrefunktion skal udelukkes. Kortikosteroider skal administreres ved bivirkninger grad ≥ 2 (initialdosis 1-2 mg/kg/dag prednison eller ækvivalent hermed efterfulgt af langsom udtrapning), og på basis af sværhedsgraden af kreatininstigningen skal behandlingen med pembrolizumab pauseres ved grad 2-nefritis og seponeres permanent ved grad 3- eller 4-nefritis (se pkt. 4.2).

Immunrelaterede endokrinopatier
Svære endokrinopatier, herunder binyrebarkinsufficiens, hypofysitis, type 1-diabetes mellitus, diabetisk ketoacidose, hypotyroidisme og hypertyroidisme er observeret i forbindelse med pembrolizumab-behandling.

Langtidsbehandling med hormonsubstitution kan være nødvendig ved immunrelaterede endokrinopatier.

Binyrebarkinsufficiens (primær og sekundær) er rapporteret hos patienter, der fik pembrolizumab. Hypofysitis er også rapporteret hos patienter, der fik pembrolizumab (se pkt. 4.8). Patienterne skal monitoreres for tegn og symptomer på binyrebarkinsufficiens og hypofysitis (inklusive hypofyseinsufficiens (hypopituitarisme), og andre årsager skal udelukkes. Kortikosteroider skal administreres for at behandle binyrebarkinsufficiens og anden hormonsubstitutionsbehandling som klinisk indiceret. Behandlingen med pembrolizumab skal pauseres ved grad 2-binyrebarkinsufficiens eller symptomatisk hypofysitis, indtil bivirkningen er kontrolleret med hormonsubstitutionsbehandling. Pembrolizumab skal pauseres eller seponeres ved grad 3- eller 4-binyrebarkinsufficiens eller -hypofysitis. Det kan overvejes at fortsætte behandlingen med pembrolizumab efter langsom udtrapning af kortikosterioder, hvis det er nødvendigt (se pkt. 4.2). Hypofysefunktion og hormonniveauer skal monitoreres for at sikre passende hormonsubstitutionsbehandling.

Type 1-diabetes mellitus, herunder diabetisk ketoacidose, er rapporteret hos patienter, der fik pembrolizumab (se pkt. 4.8). Patienterne skal monitoreres for hyperglykæmi eller andre tegn og symptomer på diabetes. Insulin skal administreres ved type 1-diabetes, og behandlingen med pembrolizumab skal pauseres i tilfælde af type 1-diabetes associeret med grad > 3-hyperglykæmi eller ketoacidose, indtil der er opnået metabolisk kontrol (se pkt. 4.2).

Thyreoideasygdomme, herunder hypotyroidisme, hypertyroidisme og tyroiditis, er rapporteret hos patienter, der fik pembrolizumab, og kan forekomme i hele behandlingsforløbet. Hypotyroidisme er rapporteret oftere hos patienter med HNSCC, som tidligere havde fået strålebehandling. Patienterne skal derfor monitoreres for ændringer i thyreoideafunktionen (ved behandlingsstart, periodevist i løbet af behandlingen og som indiceret på basis af den kliniske vurdering) samt for kliniske tegn og symptomer på thyreoideasygdomme. Hypotyroidisme kan substitutionsbehandles uden afbrydelse af behandlingen og uden kortikosteroider. Hypertyroidisme kan behandles symptomatisk. Pembrolizumab skal pauseres ved grad ≥3-hypertyroidisme indtil bedring til grad ≤ 1. Thyroideafunktion og hormonniveauer skal monitoreres for at sikre passende hormonsubstitutionsbehandling.

Hos patienter med grad 3- eller grad 4-endokrinopatier, som bedres til grad 2 eller derunder, og som er kontrolleret med hormonsubstitutionsbehandling, hvis det er indiceret, kan detovervejes at fortsætte behandlingen med pembrolizumab efter langsom udtrapning af kortikosteroider, hvis det er nødvendigt. Ellers skal behandlingen seponeres (se pkt. 4.2 og 4.8).

Immunrelaterede hudreaktioner
Alvorlige immunrelaterede hudreaktioner er rapporteret hos patienter, der fik pembrolizumab (se pkt. 4.8). Patienterne skal monitoreres ved mistanke om alvorlige hudreaktioner, og andre årsager skal udelukkes. På basis af bivirkningens sværhedsgrad skal pembrolizumab pauseres ved grad 3-hudreaktioner indtil bedring til grad ≤ 1 eller seponeres permanent ved grad 4-hudreaktioner, og kortikosteroider skal administreres (se pkt. 4.2).

Tilfælde af Stevens-Johnsons syndrom (SJS) og toksisk epidermal nekrolyse (TEN) er rapporteret hos patienter, der fik pembrolizumab (se pkt. 4.8). Ved mistanke om SJS eller TEN, skal behandling med

pembrolizumab pauseres, og patienten henvises til en specialafdeling til vurdering og behandling. Hvis mistanken om SJS eller TEN bekræftes, skal pembrolizumab seponeres permanent (se pkt. 4.2).

Der skal udvises forsigtighed, hvis pembrolizumab-behandling overvejes hos en patient, der har oplevet en alvorlig eller livstruende hudreaktion under tidligere behandling med andre immunstimulerende cancerlægemidler.

Andre immunrelaterede bivirkninger
Følgende yderligere klinisk signifikante, immunrelaterede bivirkninger er rapporteret i kliniske studier eller efter markedsføring: uveitis, artritis, myositis, myokarditis, pankreatitis, Guillain-Barrés syndrom, myastenisk syndrom, hæmolytisk anæmi, sarkoidose, encephalitis og myelitis (se pkt. 4.2 og 4.8).

Behandlingen med pembrolizumab skal pauseres ved grad 2- eller grad 3-bivirkninger og kortikosteroider administreres på basis af bivirkningens sværhedsgrad og type.

Behandlingen med pembrolizumab kan genoptages inden for 12 uger efter den sidste dosis af KEYTRUDA, hvis bivirkningen bedres til grad ≤ 1, og kortikosteroiddosis er blevet reduceret til ≤ 10 mg prednison dagligt eller ækvivalent hermed.

Behandlingen med pembrolizumab skal seponeres permanent ved en hvilken som helst immunrelateret bivirkning af grad 3, som recidiverer, og ved alle immunrelaterede bivirkninger af grad 4.

Pembrolizumab skal seponeres permanent ved grad 3- eller 4-myokarditis, encephalitis eller Guillain-Barrés syndrom (se pkt. 4.2 og 4.8).

Transplantationsrelaterede bivirkninger

Afstødning af transplanterede solide organer
Der er efter markedsføringen rapporteret om afstødning af transplanterede solide organer hos patienter, der er behandlet med PD-1-hæmmere. Behandling med pembrolizumab kan øge risikoen for afstødning hos modtagere af transplanterede solide organer. Fordelene ved behandling med pembrolizumab skal afvejes mod risikoen for mulig organafstødning hos disse patienter.

Komplikationer ved allogen hæmatopoietisk stamcelletransplantation (HSCT)

Allogen HSCT efter behandling med pembrolizumab
Tilfælde af *graft versus host*-sygdom (GVHD) og hepatisk veno-okklusiv sygdom (VOD) er observeret hos patienter med cHL, der gennemgår allogen HSCT efter tidligere eksponering for pembrolizumab. Indtil yderligere data bliver tilgængelige, skal de potentielle fordele ved HSCT og den potentielt øgede risiko for transplantationsrelaterede komplikationer vurderes nøje for hver enkelt patient (se pkt. 4.8).

Allogen HSCT før behandling med pembrolizumab
Hos patienter, der tidligere har gennemgået allogen HSCT, er der rapporteret akut GVHD, inklusive GVHD med dødelig udgang, efter behandling med pembrolizumab. Patienter, der udviklede GVHD efter deres transplantation, kan have øget risiko for GVHD efter behandling med pembrolizumab. Fordelene ved behandling med pembrolizumab skal afvejes mod risikoen for mulig GVHD hos patienter, der tidligere har gennemgået allogen HSCT.

Infusionsreaktioner
Alvorlige infusionsreaktioner, herunder overfølsomhed og anafylaksi, er rapporteret hos patienter, der fik pembrolizumab (se pkt. 4.8). Ved grad 3- eller 4-infusionsreaktioner skal infusionen stoppes og pembrolizumab seponeres permanent (se pkt. 4.2). Patienter med grad 1- eller 2-infusionsreaktioner kan fortsætte med at få pembrolizumab under tæt monitorering. Præmedicinering med antipyretikum og antihistamin kan overvejes.

Sygdomsspecifikke forholdsregler

Brug af pembrolizumab hos patienter med urotelialt karcinom, som tidligere har fået platinbaseret kemoterapi
Lægen bør overveje pembrolizumabs forsinkede indsættelse af virkning, før behandling initieres hos patienter med dårligere prognoser og/eller aggressiv sygdom. Ved urotelialt karcinom sås et højere antal dødsfald indenfor 2 måneder med pembrolizumab sammenlignet med kemoterapi (se pkt. 5.1). Faktorer associeret med tidlig død var hurtig progressiv sygdom efter tidligere platinbehandling og levermetastaser.

Brug af pembrolizumab ved urotelialt karcinom til patienter, som er uegnede til behandling med cisplatinbaseret kemoterapi, og hvis tumorer udtrykker PD-L1 med CPS ≥ 10
Prognostiske sygdomskarakteristika ved *baseline* hos studiepopulationen i KEYNOTE-052 inkluderede en procentdel patienter, der var egnet til carboplatinbaseret kombinationsbehandling, hvor fordelen bliver vurderet i et komparativt studie, og patienter, der var egnede til mono-kemoterapi, hvor der ikke foreligger randomiserede data. Derudover findes der ingen data vedrørende sikkerhed og virkning hos svagere patienter (f.eks. ECOG-performance-status 3), der ikke anses for egnede til behandling med kemoterapi. I mangel af disse data bør pembrolizumab anvendes med forsigtighed hos denne population efter nøje overvejelse af det potentielle benefit/risk-forhold på individuel basis.

Brug af pembrolizumab til førstelinjebehandling af patienter med NSCLC
Generelt forekommer bivirkninger hyppigere ved kombinationsbehandling med pembrolizumab end ved pembrolizumab som monoterapi eller kemoterapi alene, hvilket afspejler bidrag fra hver af de individuelle behandlinger (se pkt. 4.2 og 4.8). Der foreligger ikke en direkte sammenligning af pembrolizumab ved anvendelse i kombination med kemoterapi i forhold til pembrolizumab som monoterapi.

Lægen bør overveje benefit/risk-forholdet for de tilgængelige behandlingsmuligheder (pembrolizumab som monoterapi eller pembrolizumab i kombination med kemoterapi), før behandling initieres hos tidligere ubehandlede patienter med NSCLC, hvis tumorer udtrykker PD-L1.

Data vedrørende sikkerhed og virkning hos patienter ≥ 75 år er begrænsede. Kombinationsbehandling med pembrolizumab skal anvendes med forsigtighed hos patienter ≥ 75 år efter grundig overvejelse af benefit/risk-forholdet på individuel basis (se pkt. 5.1).

Brug af pembrolizumab til førstelinjebehandling af patienter med HNSCC
Generelt forekommer bivirkninger hyppigere ved kombinationsbehandling med pembrolizumab end ved pembrolizumab som monoterapi eller kemoterapi alene, hvilket afspejler bidrag fra hver af de individuelle behandlinger (se pkt. 4.8).

Lægen bør overveje benefit/risk-forholdet for de tilgængelige behandlingsmuligheder (pembrolizumab som monoterapi eller pembrolizumab i kombination med kemoterapi), før behandling initieres hos patienter med HNSCC, hvis tumorer udtrykker PD-L1 (se pkt. 5.1).

Brug af pembrolizumab til adjuverende behandling af melanom-patienter
En tendens til øget hyppighed af svære og alvorlige bivirkninger hos patienter ≥ 75 år blev observeret. Der er begrænsede data vedrørende sikkerhed fra patienter ≥ 75 år, som har fået adjuverende behandling for melanom.

Brug af pembrolizumab i kombination med axitinib til førstelinjebehandling af RCC-patienter
Ved administration af pembrolizumab i kombination med axitinib er der hos patienter med fremskredent RCC blevet rapporteret en højere forekomst end forventet af grad 3 og 4 forhøjelser af ALAT og ASAT (se pkt. 4.8). Leverenzymer skal monitoreres før initiering af behandlingen og med jævne mellemrum under behandlingen. Det kan overvejes at udføre hyppigere monitorering af leverenzymer i forhold til, når lægemidlerne anvendes som monoterapi. Retningslinjerne for medicinsk behandling for begge lægemidler skal følges (se pkt. 4.2 og produktresuméet for axitinib).

Patienter, der blev ekskluderet fra kliniske studier
Patienter med følgende tilstande blev ekskluderet fra kliniske studier: patienter med aktive metastaser i centralnervesystemet; patienter med ECOG-performance-status ≥ 2 (med undtagelse af urotelialt karcinom og RCC); patienter med hiv-infektion, hepatitis B- eller hepatitis C-infektion; patienter med aktiv systemisk autoimmun sygdom; patienter med interstitiel lungesygdom; patienter med pneumonitis i anamnesen, der krævede behandling med systemiske kortikosteroider; patienter, der tidligere har haft svær overfølsomhed over for andre monoklonale antistoffer; patienter i immunsuppressiv behandling samt patienter, der tidligere har haft alvorlige immunrelaterede bivirkninger ved behandling med ipilimumab, defineret som toksicitet af grad 4 eller af grad 3, hvor kortikosteroider (> 10 mg/dag af prednison eller ækvivalent hermed) i mere end 12 uger var nødvendig. Patienter med aktive infektioner blev ekskluderet fra kliniske studier, og det var en forudsætning, at infektionen blev behandlet, inden de kunne få pembrolizumab. Patienter, hvor aktive infektioner indtrådte under behandlingen med pembrolizumab, blev behandlet med passende medicinsk behandling. Patienter med klinisk signifikant nedsat nyrefunktion (kreatinin > 1,5 x ULN) eller leverfunktion (bilirubin > 1,5 x ULN, ALAT, ASAT > 2,5 x ULN med fravær af levermetastaser) ved *baseline* blev ekskluderet fra kliniske studier. Derfor foreligger der kun begrænset information om patienter med svært nedsat nyrefunktion og moderat til svært nedsat leverfunktion.

Der foreligger begrænsede data om KEYTRUDAs sikkerhed og virkning hos patienter med okulært melanom (se pkt. 5.1).

For så vidt angår recidiverende eller refraktært cHL foreligger der kun begrænsede kliniske data for anvendelse af pembrolizumab hos patienter, som er uegnede til ASCT af andre årsager end behandlingssvigt efter salvage-kemoterapi (se pkt. 5.1).

Efter nøje overvejelse af den potentielt øgede risiko kan pembrolizumab anvendes til disse patienter med passende klinisk håndtering.

Patientinformationskort
Alle læger, som påtænker at ordinere KEYTRUDA, skal sikre sig kendskab til indholdet i informationen til lægen og behandlingsvejledningen. Lægen skal drøfte risiciene ved KEYTRUDA-behandlingen med patienten. Patienten skal have udleveret patientinformationskortet ved hver ordination.

4.5 Interaktion med andre lægemidler og andre former for interaktion

Der er ikke udført formelle farmakokinetiske lægemiddelinteraktionsstudier med pembrolizumab. Eftersom pembrolizumab elimineres fra kredsløbet via nedbrydning (katabolisme) forventes ingen metaboliske lægemiddelinteraktioner.

Systemiske kortikosteroider og immunsuppressiva bør undgås før initiering af behandling med pembrolizumab på grund af deres potentielle interferens med pembrolizumabs farmakodynamiske aktivitet og virkning. Systemiske kortikosteroider og andre immunsuppressiva kan dog anvendes til at behandle immunrelaterede bivirkninger efter initiering af pembrolizumab (se pkt. 4.4). Kortikosteroider kan også anvendes som præmedicinering, når pembrolizumab anvendes i kombination med kemoterapi, som profylaktisk antiemetisk behandling og/eller til at mildne kemoterapi-relaterede bivirkninger.

4.6 Fertilitet, graviditet og amning

Kvinder i den fertile alder
Kvinder i den fertile alder skal anvende sikker kontraception under behandlingen med pembrolizumab og i mindst 4 måneder efter den sidste pembrolizumab-dosis.

Graviditet
Der er ingen data fra anvendelse af pembrolizumab til gravide kvinder. Der har ikke været udført reproduktionsstudier med pembrolizumab hos dyr. I dyremodeller med drægtige mus er det blevet påvist, at blokering af PD-L1-signalet kan påvirke tolerancen for fostret og medføre et stigende antal fostertab (se pkt. 5.3). Med udgangspunkt i pembrolizumabs virkningsmekanisme viser disse resultater en potentiel

risiko for, at administration af pembrolizumab under graviditet kan medføre fosterskader, herunder en øget forekomst af abort eller dødfødsel. Det er vist, at humant immunglobulin G4 (IgG4) kan passere placentabarrieren, og da pembrolizumab er et IgG4, kan pembrolizumab derfor potentielt overføres fra moderen til fosteret. Pembrolizumab bør ikke anvendes under graviditet, medmindre kvindens kliniske tilstand kræver behandling med pembrolizumab.

Amning
Det er ukendt, om pembrolizumab udskilles i human mælk. En risiko for nyfødte/spædbørn kan ikke udelukkes, da det er kendt, at antistoffer kan udskilles i human mælk. Det skal besluttes, om amning skal ophøre eller behandling med pembrolizumab skal seponeres, idet der tages højde for fordelene ved amning for barnet i forhold til de terapeutiske fordele ved pembrolizumab-behandlingen for moderen.

Fertilitet
Der foreligger ingen data om pembrolizumabs mulige indvirkning på fertilitet. Toksicitetsstudier af 1 måned og 6 måneders varighed med gentagne doser viste ingen væsentlig indvirkning på forplantningsorganerne hos han- og hunaber (se pkt. 5.3).

4.7 Virkning på evnen til at føre motorkøretøj og betjene maskiner

Pembrolizumab påvirker i mindre grad evnen til at føre motorkøretøj og betjene maskiner. Svimmelhed og træthed er rapporteret hos nogle patienter efter administration af pembrolizumab (se pkt. 4.8).

4.8 Bivirkninger

Oversigt over sikkerhedsprofilen
Pembrolizumab er sædvanligvis associeret med immunrelaterede bivirkninger. De fleste af disse, herunder alvorlige bivirkninger, gik over efter initiering af passende medicinsk behandling eller pausering af behandlingen af pembrolizumab (se "Beskrivelse af udvalgte bivirkninger" nedenfor).

Pembrolizumabs sikkerhed som monoterapi er blevet vurderet hos 5.884 patienter med fremskredent melanom, resekteret stadie III-melanom (adjuverende behandling), NSCLC, cHL, urotelialt karcinom eller HNSCC ved 4 doser (2 mg/kg hver 3. uge, 200 mg hver 3. uge eller 10 mg/kg hver 2. eller 3. uge) i kliniske studier. Hyppighederne, som er inkluderet nedenfor og i tabel 2, er baseret på alle rapporterede bivirkninger, uden hensyn til investigators vurdering af kausalitet. I denne patientpopulation var den mediane observationstid 7,3 måneder (interval: 1 dag til 31 måneder), og de hyppigste bivirkninger med pembrolizumab var: træthed (32%), kvalme (20%) og diarré (20%). Størstedelen af de rapporterede bivirkninger ved monoterapi var af sværhedsgrad 1 eller 2. De alvorligste bivirkninger var immunrelaterede bivirkninger og alvorlige infusionsreaktioner (se pkt. 4.4).

Pembrolizumabs sikkerhed i kombination med kemoterapi er blevet vurderet hos 1.067 patienter med NSCLC eller HNSCC, der fik 200 mg, 2 mg/kg eller 10 mg/kg pembrolizumab hver 3. uge, i kliniske studier. Hyppighederne, som er inkluderet nedenfor og i tabel 2, er baseret på alle rapporterede bivirkninger, uden hensyn til investigators vurdering af kausalitet. I denne patientpopulation var de hyppigste bivirkninger: anæmi (50%), kvalme (50%), træthed (37%), obstipation (35%), diarré (30%), neutropeni (30%), appetitløshed (28%) og opkastning (25%). Forekomsten af bivirkninger af grad 3-5 hos patienter med NSCLC var 67% for kombinationsbehandling med pembrolizumab og 66% for kemoterapi alene og forekomsten hos patienter med HNSCC var 85% for kombinationsbehandling med pembrolizumab og 84% for kemoterapi plus cetuximab.

Pembrolizumabs sikkerhed i kombination med axitinib er blevet vurderet i et klinisk studie med 429 patienter med fremskredent RCC, der fik 200 mg pembrolizumab hver 3. uge og 5 mg axitinib to gange dagligt. I denne patientpopulation var de hyppigste bivirkninger: diarré (54%), hypertension (45%), træthed (38%), hypotyroidisme (35%), appetitløshed (30%), palmoplantar erytrodysæstesisyndrom (hånd-fod-syndrom) (28%), kvalme (28%), forhøjet ALAT (27%), forhøjet ASAT (26%), dysfoni (25%), hoste (21%) og obstipation (21%). Forekomsten af bivirkninger af grad 3-5 var 76% for kombinationsbehandling med pembrolizumab og 71% for sunitinib alene.

Bivirkningstabel

De bivirkninger, der blev observeret i kliniske studier med pembrolizumab som monoterapi eller i kombination med kemoterapi eller andre anti-tumor lægemidler eller rapporteret i forbindelse med brug af pembrolizumab efter markedsføring, er opført i tabel 2. Bivirkninger, der vides at forekomme ved behandling med pembrolizumab eller ved kemoterapi givet alene, kan forekomme under behandlingen med disse lægemidler i kombination, selvom disse bivirkninger ikke blev rapporteret i kliniske studier med kombinationsbehandling. Disse bivirkninger er anført ud fra systemorganklasse og hyppighed. Hyppigheden er angivet som: Meget almindelig (≥ 1/10); Almindelig (≥ 1/100 til < 1/10); Ikke almindelig (≥ 1/1.000 til < 1/100); Sjælden (≥ 1/10.000 til < 1/1.000); Meget sjælden (< 1/10.000) og Ikke kendt (kan ikke estimeres ud fra forhåndenværende data). I hver hyppighedsgruppe er bivirkningerne opstillet efter faldende alvorlighed.

Tabel 2: Bivirkninger hos patienter, som blev behandlet med pembrolizumab*

	**Monoterapi**	**Kombination med kemoterapi**	**Kombination med axitinib**
**Infektioner og parasitære sygdomme**			
Almindelig	pneumoni	pneumoni	pneumoni
**Blod og lymfesystem**			
Meget almindelig	anæmi	anæmi, neutropeni, trombocytopeni	
Almindelig	trombocytopeni, lymfopeni	febril neutropeni, leukopeni, lymfopeni	anæmi, neutropeni, leukopeni, trombocytopeni
Ikke almindelig	neutropeni, leukopeni, eosinofili		lymfopeni, eosinofili
Sjælden	immun trombocytopenisk purpura, hæmolytisk anæmi, ren erytrocyt aplasi, hæmofagocytisk lymfohistiocytose	eosinofili	
**Immunsystemet**			
Almindelig	infusionsreaktioner[a]	infusionsreaktioner[a]	infusionsreaktioner[a]
Ikke almindelig	sarkoidose		
Ikke kendt	afstødning af transplanterede solide organer		
**Det endokrine system**			
Meget almindelig	hypotyroidisme[b]		hypertyroidisme, hypotyroidisme[b]
Almindelig	hypertyroidisme	hypotyroidisme, hypertyroidisme	hypofysitis[d], tyroiditis[e], binyrebarkinsufficiens[c]
Ikke almindelig	binyrebarkinsufficiens[c], hypofysitis[d], tyroiditis[e]	hypofysitis[d], tyroiditis[e], binyrebarkinsufficiens[c]	
**Metabolisme og ernæring**			
Meget almindelig	appetitløshed	hypokaliæmi, appetitløshed	appetitløshed
Almindelig	hyponatriæmi, hypokaliæmi, hypokalcæmi	hyponatriæmi, hypokalcæmi	hypokaliæmi, hyponatriæmi, hypokalcæmi
Ikke almindelig	type 1-diabetes mellitus[f]	type 1-diabetes mellitus	type 1-diabetes mellitus[f]
**Psykiske forstyrrelser**			
Almindelig	insomni	insomni	insomni
**Nervesystemet**			
Meget almindelig	hovedpine	svimmelhed, hovedpine, perifer neuropati, dysgeusi	hovedpine, dysgeusi

Almindelig	svimmelhed, perifer neuropati, letargi, dysgeusi	letargi	svimmelhed, letargi, perifer neuropati
Ikke almindelig	epilepsi	epilepsi	myastenisk syndrom[i]
Sjælden	encephalitis, Guillain-Barrés syndrom[g], myelitis[h], myastenisk syndrom[i], meningitis (aseptisk)[j]		
**Øjne**			
Almindelig	øjentørhed	øjentørhed	øjentørhed
Ikke almindelig	uveitis[k]		uveitis[k]
Sjælden	Vogt-Koyanagi-Harada-syndrom		
**Hjerte**			
Almindelig	hjertearytmi[†] (inklusive atrieflimren)	hjertearytmi[†] (inklusive atrieflimren)	hjertearytmi[†] (inklusive atrieflimren)
Ikke almindelig	perikardieeffusion, perikarditis	perikardieeffusion	myokarditis[l]
Sjælden	myokarditis[l]	myokarditis[l], perikarditis	
**Vaskulære sygdomme**			
Meget almindelig			hypertension
Almindelig	hypertension	hypertension	
**Luftveje, thorax og mediastinum**			
Meget almindelig	dyspnø, hoste	dyspnø, hoste	dyspnø, hoste, dysfoni
Almindelig	pneumonitis[m]	pneumonitis[m]	pneumonitis[m]
**Mave-tarm-kanalen**			
Meget almindelig	diarré, abdominalsmerter[n], kvalme, opkastning, obstipation	diarré, kvalme, opkastning, obstipation, abdominalsmerter[n]	diarré, abdominalsmerter[n], kvalme, opkastning, obstipation
Almindelig	colitis[o], mundtørhed	colitis[o], mundtørhed	colitis[o], mundtørhed
Ikke almindelig	pankreatitis[p], gastrointestinal ulceration[q]	pankreatitis[p], gastrointestinal ulceration[q]	pankreatitis[p], gastrointestinal ulceration[q]
Sjælden	perforation af tyndtarmen		
**Lever og galdeveje**			
Almindelig			hepatitis[r]
Ikke almindelig	hepatitis[r]	hepatitis[r]	
**Hud og subkutane væv**			
Meget almindelig	udslæt[s], pruritus[t]	udslæt[s], alopeci, pruritus[t]	palmoplantar erytrodysæstesisyndrom (hånd-fod-syndrom), udslæt[s], pruritus[t]
Almindelig	alvorlige hudreaktioner[u], erytem, tør hud, vitiligo[v], eksem, alopeci, akneiform dermatitis	alvorlige hudreaktioner[u], erytem, tør hud	alvorlige hudreaktioner[u], akneiform dermatitis, dermatitis, tør hud, alopeci, eksem, erytem
Ikke almindelig	likenoid keratose[w], psoriasis, dermatitis, papel, ændret hårfarve	psoriasis, akneiform dermatitis, dermatitis, vitiligo[v], eksem	ændret hårfarve, likenoid keratose, papel, psoriasis, vitiligo[v]
Sjælden	toksisk epidermal nekrolyse, Stevens-Johnsons syndrom, erythema nodosum	ændret hårfarve, likenoid keratose, papel	

**Knogler, led, muskler og bindevæv**			
Meget almindelig	muskuloskeletale smerter[x], artralgi	muskuloskeletale smerter[x], artralgi	muskuloskeletale smerter[x], artralgi, ekstremitetssmerter
Almindelig	ekstremitetssmerter, myositis[y], artritis[z]	myositis[y], ekstremitetssmerter, artritis[z]	myositis[y], artritis[z], tenosynovitis[aa]
Ikke almindelig	tenosynovitis[aa]	tenosynovitis[aa]	
**Nyrer og urinveje**			
Almindelig		nefritis[bb], akut nyreskade	akut nyreskade, nefritis[bb]
Ikke almindelig	nefritis[bb]		
**Almene symptomer og reaktioner på administrationsstedet**			
Meget almindelig	træthed, asteni, ødem[cc], pyreksi	træthed, asteni, pyreksi, ødem[cc]	træthed, asteni, pyreksi
Almindelig	influenzalignende sygdom, kuldegysninger	kuldegysninger, influenzalignende sygdom	ødem[cc], influenzalignende sygdom, kuldegysninger
**Undersøgelser**			
Meget almindelig		forhøjet kreatinin i blodet	forhøjet alaninaminotransferase, forhøjet aspartataminotransferase, forhøjet kreatinin i blodet
Almindelig	forhøjet aspartataminotransferase, forhøjet alaninaminotransferase, hyperkalcæmi, forhøjet alkalisk fosfatase i blodet, forhøjet bilirubin i blodet, forhøjet kreatinin i blodet	hyperkalcæmi, forhøjet alaninaminotransferase, forhøjet aspartataminotransferase, forhøjet alkalisk fosfatase i blodet	forhøjet alkalisk fosfatase i blodet, hyperkalcæmi, forhøjet bilirubin i blodet
Ikke almindelig	forhøjet amylase	forhøjet bilirubin i blodet, forhøjet amylase	forhøjet amylase

*Bivirkningshyppighederne, som er angivet i tabel 2, kan muligvis ikke udelukkende henføres til pembrolizumab alene, men kan måske også indeholde bidrag fra den underliggende sygdom eller fra andre lægemidler, som anvendes i kombination.
†Baseret på en standardforespørgsel inklusive bradyarytmier og takyarytmier.
Følgende termer repræsenterer en gruppe beslægtede bivirkninger, som snarere beskriver en medicinsk tilstand end en enkelt bivirkning.

a. infusionsreaktion (lægemiddeloverfølsomhed, anafylaktisk reaktion, anafylaktoid reaktion, overfølsomhed og cytokin-frigivelsessyndrom)
b. hypotyroidisme (myksødem)
c. binyrebarkinsufficiens (Addisons sygdom, akut binyrebarkinsufficiens, sekundær binyrebarkinsufficiens)
d. hypofysitis (hypopituitarisme)
e. tyroiditis (autoimmun tyroiditis og thyroideasygdom)
f. type 1-diabetes mellitus (diabetisk ketoacidose)
g. Guillain-Barrés syndrom (axonal neuropati og demyeliniserende polyneuropati)
h. myelitis (inklusive transversel myelitis)
i. myastenisk syndrom (myasthenia gravis, inklusive eksacerbation)
j. aseptisk meningitis (meningitis, non-infektiøs meningitis)
k. uveitis (iritis og iridocyklitis)
l. myokarditis (autoimmun myokarditis)
m. pneumonitis (interstitiel lungesygdom)
n. abdominalsmerter (abdominalgener, øvre abdominalsmerter og nedre abdominalsmerter)
o. colitis (mikroskopisk colitis, enterocolitis, hæmoragisk enterocolitis og autoimmun colitis)
p. pankreatitis (autoimmun pankreatitis og akut pankreatitis)
q. gastrointestinal ulceration (ulcus ventriculi og ulcus duodeni)
r. hepatitis (autoimmun hepatitis, immunmedieret hepatitis og lægemiddelinduceret leverskade)
s. udslæt (erytematøst udslæt, follikulært udslæt, generaliseret udslæt, makuløst udslæt, makulopapuløst udslæt, papuløst udslæt, pruritisk udslæt, vesikuløst udslæt og genitalt udslæt)
t. pruritus (urticaria, papuløs urticaria, generaliseret pruritus og genital pruritus)
u. alvorlige hudreaktioner (bulløs dermatit, eksfoliativ dermatitis, erythema multiforme, eksfoliativt udslæt, pemphigus, hudnekrose, toksisk hududslæt samt ≥ grad 3 af følgende: akut febril neutrofil dermatose, kontusion, liggesår,

psoriasiform dermatitis, lægemiddeludslæt, gulsot, pemfigoid, pruritus, generaliseret pruritus, udslæt, erytematøst udslæt, generaliseret udslæt, makulopapuløst udslæt, pruritisk udslæt, pustuløst udslæt og hudlæsion)

v. vitiligo (depigmentering af huden, hypopigmentering af huden og hypopigmentering af øjenlåget)
w. likenoid keratose (lichen planus og lichen sclerosus)
x. muskuloskeletale smerter (muskuloskeletale gener, rygsmerter, muskuloskeletal stivhed, muskuloskeletale brystsmerter og torticollis)
y. myositis (myalgi, myopati, polymyalgia rheumatica og rabdomyolyse)
z. artritis (hævede led, polyartritis og ledeffusion)
aa. tenosynovitis (tendinitis, synovitis og senesmerter)
bb. nefritis (autoimmun nefritis, tubulointerstitiel nefritis og nyresvigt, akut nyresvigt eller akut nyreskade med tegn på nefritis, nefrotisk syndrom og membranøs glomerulonefritis)
cc. ødem (perifert ødem, generaliseret ødem, overhydrering, væskeretention, øjenlågsødem og læbeødem, ansigtsødem, lokaliseret ødem og periorbitalt ødem)

Beskrivelse af udvalgte bivirkninger

Dataene for følgende immunrelaterede bivirkninger er baseret på patienter, som har fået pembrolizumab i kliniske studier (4 doseringsregimer: 2 mg/kg hver 3. uge, 10 mg/kg hver 2. eller 3. uge eller 200 mg hver 3. uge) (se pkt. 5.1). Vejledning til håndtering af disse bivirkninger findes i pkt. 4.4.

Immunrelaterede bivirkninger (se pkt. 4.4)

Immunrelateret pneumonitis

Pneumonitis forekom hos 253 (4,3%) patienter, som fik pembrolizumab, inklusive tilfælde af grad 2, 3, 4 eller 5 hos henholdsvis 106 (1,8%), 69 (1,2%), 13 (0,2%) og 9 (0,2%) patienter. Den mediane tid til indtræden af pneumonitis var 3,3 måneder (interval: 2 dage til 26,8 måneder). Medianvarigheden var 1,9 måneder (interval: 1 dag til 25,3+ måneder). Pneumonitis forekom hyppigere hos patienter, der tidligere havde fået torakal strålebehandling (8,1%) end hos patienter, der ikke tidligere havde fået torakal strålebehandling (3,4%). Pneumonitis medførte seponering af pembrolizumab hos 98 (1,7%) patienter. Pneumonitis gik over hos 138 patienter, 2 med sequelae.

Hos patienter med NSCLC forekom pneumonitis hos 107 (4,9%), inklusive tilfælde af grad 2, 3, 4 eller 5 hos henholdsvis 39 (1,8%), 30 (1,4%), 10 (0,5%) og 9 (0,4%) patienter. Hos patienter med NSCLC forekom pneumonitis hos 8,1% af patienterne, der tidligere havde fået torakal strålebehandling.

Immunrelateret colitis

Colitis forekom hos 107 (1,8%) patienter, som fik pembrolizumab, inklusive tilfælde af grad 2, 3 eller 4 hos henholdsvis 31 (0,5%), 62 (1,1%) og 3 (< 0,1%) patienter. Den mediane tid til indtræden af colitis var 4,3 måneder (interval: 7 dage til 24,3 måneder). Medianvarigheden var 0,9 måneder (interval: 1 dag til 8,7+ måneder). Colitis medførte seponering af pembrolizumab hos 29 (0,5%) patienter. Colitis gik over hos 84 patienter, 2 med sequelae.

Immunrelateret hepatitis

Hepatitis forekom hos 50 (0,8%) patienter, som fik pembrolizumab, inklusive tilfælde af grad 2, 3 eller 4 hos henholdsvis 8 (0,1%), 31 (0,5%) og 8 (0,1%) patienter. Den mediane tid til indtræden af hepatitis var 3,6 måneder (interval: 8 dage til 21,4 måneder). Medianvarigheden var 1,1 måneder (interval: 1 dag til 20,9+ måneder). Hepatitis medførte seponering af pembrolizumab hos 19 (0,3%) patienter. Hepatitis gik over hos 36 patienter.

Immunrelateret nefritis

Nefritis forekom hos 22 (0,4%) patienter, som fik pembrolizumab som monoterapi, inklusive tilfælde af grad 2, 3 eller 4 hos henholdsvis 5 (0,1%), 14 (0,2%) og 1 (< 0,1%) patienter. Den mediane tid til indtræden af nefritis var 5,0 måneder (interval: 12 dage til 21,4 måneder). Medianvarigheden var 2,6 måneder (interval: 6 dage til 12,0 måneder). Nefritis medførte seponering af pembrolizumab hos 10 (0,2%) patienter. Nefritis gik over hos 13 patienter, 3 med sequelae. Hos patienter med ikke-planocellulær NSCLC, som fik pembrolizumab i kombination med pemetrexed og platinbaseret kemoterapi (n=488), var forekomsten af nefritis 1,4% (alle grader) med 0,8% af grad 3 og 0,4% af grad 4.

Immunrelaterede endokrinopatier

Binyrebarkinsufficiens forekom hos 46 (0,8%) patienter, som fik pembrolizumab, inklusive tilfælde af grad 2, 3 eller 4 hos henholdsvis 19 (0,3%), 20 (0,3%) og 3 (0,1%) patienter. Den mediane tid til

indtræden af binyrebarkinsufficiens var 5,4 måneder (interval: 1 dag til 17,7 måneder). Medianvarigheden blev ikke nået (interval: 3 dage til 26,2+ måneder). Binyrebarkinsufficiens medførte seponering af pembrolizumab hos 4 (0,1%) patienter. Binyrebarkinsufficiens gik over hos 16 patienter, 4 med sequelae.

Hypofysitis forekom hos 36 (0,6%) patienter, som fik pembrolizumab, inklusive tilfælde af grad 2, 3 eller 4 hos henholdsvis 13 (0,2%), 19 (0,3%) og 1 (< 0,1%) patienter. Den mediane tid til indtræden af hypofysitis var 5,9 måneder (interval: 1 dag til 17,7 måneder). Medianvarigheden var 3,3 måneder (interval: 3 dage til 18,1+ måneder). Hypofysitis medførte seponering af pembrolizumab hos 8 (0,1%) patienter. Hypofysitis gik over hos 17 patienter, 8 med sequelae.

Hypertyroidisme forekom hos 244 (4,1%) patienter, som fik pembrolizumab, inklusive tilfælde af grad 2 eller 3 hos henholdsvis 64 (1,1%) og 7 (0,1%) patienter. Den mediane tid til indtræden af hypertyroidisme var 1,4 måneder (interval: 1 dag til 22,5 måneder). Medianvarigheden var 1,8 måneder (interval: 4 dage til 29,2+ måneder). Hypertyroidisme medførte seponering af pembrolizumab hos 3 (0,1%) patienter. Hypertyroidisme gik over hos 191 (78,3%) patienter, 5 med sequelae.

Hypotyroidisme forekom hos 645 (11,0%) patienter, som fik pembrolizumab, inklusive tilfælde af grad 2 eller 3 hos henholdsvis 475 (8,1%) og 8 (0,1%) patienter. Den mediane tid til indtræden af hypotyroidisme var 3,5 måneder (interval: 1 dag til 19,6 måneder). Medianvarigheden blev ikke nået (interval: 2 dage til 32,6+ måneder). 2 patienter (< 0,1%) seponerede pembrolizumab som følge af hypotyroidisme. Hypotyroidisme gik over hos 153 (23,7%) patienter, 10 med sequelae. Hos patienter med cHL (n=241) var forekomsten af hypotyroidisme 14,1% (alle grader) med 0,4% af grad 3. Hos patienter med HNSCC, som blev behandlet med pembrolizumab som monoterapi (n=909), var forekomsten af hypotyroidisme 16,1% (alle grader) med 0,3% af grad 3. Hos patienter med HNSCC, som blev behandlet med pembrolizumab i kombination med platinbaseret kemoterapi og 5-FU (n=276), var forekomsten af hypotyroidisme 15,2%, hvoraf alle var af grad 1 eller 2.

Immunrelaterede hudreaktioner
Alvorlige immunrelaterede hudreaktioner forekom hos 89 (1,5%) patienter, som fik pembrolizumab, inklusive tilfælde af grad 2, 3 eller 5 hos henholdsvis 10 (0,2%), 65 (1,1%) og 1 (< 0,1%) patienter. Den mediane tid til indtræden af alvorlige hudreaktioner var 3,3 måneder (interval: 3 dage til 19,4 måneder). Medianvarigheden var 1,6 måneder (interval: 1 dag til 27,3+ måneder). Alvorlige hudreaktioner medførte seponering af pembrolizumab hos 9 (0,2%) patienter. Alvorlige hudreaktioner gik over hos 64 patienter.

Der er observeret sjældne tilfælde af SJS og TEN, herunder tilfælde med dødelig udgang (se pkt. 4.2 og 4.4).

Komplikationer ved allogen HSCT ved cHL
Ud af 23 patienter med cHL, som gik videre til allogen HSCT efter behandling med pembrolizumab, udviklede 6 patienter (26%) GVHD, hvoraf en døde, og 2 patienter (9%) fik svær hepatisk VOD efter konditionering med reduceret intensitet, hvoraf en døde. De 23 patienter havde en median opfølgningstid fra efterfølgende allogen HSCT på 5,1 måneder (interval: 0-26,2 måneder).

Forhøjede leverenzymer, når pembrolizumab anvendes i kombination med axitinib ved RCC
I et klinisk studie af tidligere ubehandlede RCC-patienter, der fik pembrolizumab i kombination med axitinib, blev der observeret en højere end forventet forekomst af grad 3 og 4 forhøjelser af ALAT (20%) og ASAT (13%). Den mediane tid til indtræden af forhøjet ALAT var 2,3 måneder (interval: 7 dage til 19,8 måneder). Hos patienter med ALAT ≥ 3 gange ULN (grad 2-4, n=116) forbedredes ALAT til grad 0-1 hos 94%. 59% af patienterne med forhøjet ALAT fik systemiske kortikosteroider. 92 (84%) af de patienter, der bedredes, fik genoptaget behandlingen med enten pembrolizumab (3%) eller axitinib (31%) som monoterapi eller med begge (50%). 55% af disse patienter havde ingen recidiv af ALAT > 3 gange ULN, og bedring forekom hos alle patienter med recidiv af ALAT > 3 gange ULN. Der var ingen hepatiske hændelser af grad 5.

Unormale laboratorieværdier
Hos patienter, der blev behandlet med pembrolizumab som monoterapi, var andelen af patienter, som oplevede et skift fra *baseline* til en grad 3 eller 4 unormal laboratorieværdi, følgende: 10,9% med nedsat

lymfocyttal; 8,2% med nedsat natrium; 6,3% med nedsat hæmoglobin; 5,2% med nedsat fosfat; 4,8% med forhøjet glucose; 2,8% med forhøjet ASAT; 2,7% med forhøjet alkalisk fosfatase; 2,7% med forhøjet ALAT; 2,2% med nedsat kalium; 1,8% med forhøjet kalcium; 1,8% med nedsat neutrofiltal; 1,8% med forhøjet kalium; 1,7% med forhøjet bilirubin; 1,7% med nedsat trombocyttal; 1,6% med nedsat albumin; 1,5% med nedsat kalcium; 1,3% med forhøjet kreatinin; 0,8% med nedsat leukocyttal; 0,7% med forhøjet magnesium; 0,6% med nedsat glucose; 0,2% med nedsat magnesium og 0,2% med forhøjet natrium.

Hos patienter, der blev behandlet med pembrolizumab i kombination med kemoterapi, var andelen af patienter, som oplevede et skift fra *baseline* til unormal laboratorieværdi af grad 3 eller 4, følgende: 26,7% med nedsat neutrofiltal; 23,9% med nedsat lymfocyttal; 19,1% med nedsat hæmoglobin; 17,9% med nedsat leukocyttal; 12,2% med nedsat trombocyttal; 10,2% med nedsat natrium; 8,9% med nedsat fosfat; 7,4% med forhøjet glucose; 6,5% med nedsat kalium; 3,3% med forhøjet kreatinin; 3,1% med forhøjet ALAT; 3,1% med forhøjet ASAT; 3,1% med nedsat kalcium; 3,0% med forhøjet kalium; 2,9% med nedsat albumin; 2,3% med forhøjet kalcium; 1,2% med forhøjet alkalisk fosfatase; 0,8% med nedsat glucose; 0,7% med forhøjet bilirubin og 0,3% med forhøjet natrium.

Hos patienter, der blev behandlet med pembrolizumab i kombination med axitinib, var andelen af patienter, som oplevede et skift fra *baseline* til en grad 3 eller 4 unormal laboratorieværdi, følgende: 20,1% med forhøjet ALAT; 13,2% med forhøjet ASAT; 10,8% med nedsat lymfocyttal; 8,9% med forhøjet glucose; 7,8% med nedsat natrium; 6,4% med nedsat fosfat; 6,2% med forhøjet kalium; 4,3% med forhøjet kreatinin; 3,6% med nedsat kalium; 2,1% med forhøjet bilirubin; 2,1% med nedsat hæmoglobin; 1,7% med forhøjet alkalisk fosfatase; 1,5% med forhøjet INR (protrombintid); 1,4% med nedsat leukocyttal; 1,4% med nedsat trombocyttal; 1,2% med forlænget aktiveret partiel tromboplastintid (APTT); 1,2% med nedsat neutrofiltal; 1,2% med forhøjet natrium; 0,7% med nedsat kalcium; 0,7% med forhøjet kalcium; 0,5% med nedsat albumin og 0,2% med nedsat glucose.

Immunogenicitet

I kliniske studier med patienter, som blev behandlet med pembrolizumab 2 mg/kg hver 3. uge, 200 mg hver 3. uge eller 10 mg/kg hver 2. eller 3. uge som monoterapi, havde 36 (1,8%) ud af 2.034 evaluerbare patienter en positiv test for antistoffer mod pembrolizumab udviklet under behandlingen, hvoraf 9 (0,4%) patienter havde neutraliserende antistoffer mod pembrolizumab. Der sås ingen tegn på ændret farmakokinetisk profil eller sikkerhedsprofil i forbindelse med anti-pembrolizumab binding eller dannelse af neutraliserende antistoffer mod pembrolizumab.

Pædiatrisk population

Pembrolizumabs sikkerhed som monoterapi blev vurderet hos 154 pædiatriske patienter med fremskredent melanom, lymfom eller PD-L1-positive fremskredne, recidiverende eller refraktære solide tumorer ved en dosis på 2 mg/kg hver 3. uge i fase I/II-studiet KEYNOTE-051. Sikkerhedsprofilen hos disse pædiatriske patienter var generelt sammenlignelig med sikkerhedsprofilen hos voksne, der blev behandlet med pembrolizumab. De hyppigste bivirkninger (rapporteret hos mindst 20% af de pædiatriske patienter) var pyreksi (31%), opkastning (26%), hovedpine (22%), abdominalsmerter (21%), anæmi (21%) og obstipation (20%). Størstedelen af de rapporterede bivirkninger ved monoterapi var af sværhedsgrad 1 eller 2. 69 (44,8%) patienter havde 1 eller flere bivirkninger af grad 3 til 5, hvoraf 6 (3,9%) patienter havde 1 eller flere bivirkninger med dødelig udgang. Hyppighederne er baseret på alle rapporterede bivirkninger, uden hensyn til investigators vurdering af kausalitet.

Indberetning af formodede bivirkninger

Når lægemidlet er godkendt, er indberetning af formodede bivirkninger vigtig. Det muliggør løbende overvågning af benefit/risk-forholdet for lægemidlet. Sundhedspersoner anmodes om at indberette alle formodede bivirkninger via det nationale rapporteringssystem anført i Appendiks V.

4.9 Overdosering

Der findes ingen information vedrørende overdosering med pembrolizumab.

I tilfælde af overdosering skal patienten monitoreres tæt for tegn og symptomer på bivirkninger, og passende symptomatisk behandling iværksættes.

5. FARMAKOLOGISKE EGENSKABER

5.1 Farmakodynamiske egenskaber

Farmakoterapeutisk klassifikation: antineoplastiske stoffer, monoklonale antistoffer, ATC-kode: L01XC18

Virkningsmekanisme
KEYTRUDA er et humaniseret monoklonalt antistof, der binder til *programmed cell death*-1 (PD-1)-receptoren og blokerer dets interaktion med liganderne PD-L1 og PD-L2. PD-1-receptoren er en negativ regulator af T-celleaktivering, som er påvist at være involveret i kontrollen af T-cellemedieret immunrespons. KEYTRUDA aktiverer T-cellemedieret respons, herunder anti-tumorrespons, ved at blokere PD-1-bindingen til PD-L1 og PD-L2, som er udtrykt i antigenpræsenterende celler, og som kan udtrykkes af tumorer eller andre celler i tumorens mikromiljø.

Klinisk virkning og sikkerhed
Pembrolizumab-doser på 2 mg/kg hver 3. uge, 10 mg/kg hver 3. uge og 10 mg/kg hver 2. uge blev vurderet i kliniske studier af melanom-patienter eller NSCLC-patienter, som tidligere har været behandlet. Baseret på modellering og simulering af forholdet mellem dosis og eksponering med hensyn til pembrolizumabs virkning og sikkerhed er der ingen klinisk signifikant forskel i virkning eller sikkerhed blandt doserne på 200 mg hver 3. uge, 2 mg/kg hver 3. uge og 400 mg hver 6. uge som monoterapi (se pkt. 4.2).

Melanom
KEYNOTE-006: Kontrolleret studie med ipilimumab-naive melanom-patienter
Pembrolizumabs sikkerhed og virkning blev undersøgt i KEYNOTE-006, et åbent, kontrolleret fase III-multicenterstudie af behandling af fremskredent melanom hos patienter, der ikke tidligere havde været behandlet med ipilimumab. Patienterne blev randomiseret i forholdet 1:1:1 til at få pembrolizumab 10 mg/kg hver 2. (n=279) eller 3. uge (n=277) eller ipilimumab 3 mg/kg hver 3. uge (n=278). Det var ikke et krav, at patienter med BRAF V600E-muteret melanom skulle have fået forudgående behandling med en BRAF-hæmmer.

Patienterne blev behandlet med pembrolizumab indtil sygdomsprogression eller uacceptabel toksicitet. Klinisk stabile patienter med initial evidens på sygdomsprogression fik lov til at forblive i behandling, indtil sygdomsprogression var blevet bekræftet. Der blev foretaget vurdering af tumorstatus i uge 12, herefter hver 6. uge til og med uge 48 og derefter hver 12. uge.

Ud af 834 patienter var 60% mænd, 44% var ≥ 65 år (medianalder 62 år [interval: 18-89]), og 98% var kaukasere. 65% af patienterne havde melanom i M1c-stadiet, 9% havde hjernemetastaser i anamnesen, 66% havde ikke tidligere fået behandling, og 34% havde forudgående fået ét behandlingsregime. 31% havde ECOG-performance-status på 1, 69% havde ECOG-performance-status på 0 og 32% havde forhøjet LDH. BRAF-mutation blev rapporteret hos 302 (36%) patienter. Blandt patienterne med BRAF-muteret tumor havde 139 (46%) tidligere været i behandling med en BRAF-hæmmer.

De primære resultatmål var progressionsfri overlevelse (PFS, baseret på IRO-vurdering [*Integrated Radiology and Oncology Assessment*] i henhold til RECIST version 1.1 [*Response Evaluation Criteria in Solid Tumors*]) og samlet overlevelse (OS). De sekundære resultatmål var objektiv responsrate (ORR) og responsvarighed. I tabel 3 opsummeres de centrale effektresultater hos ipilimumab-naive patienter efter den endelige analyse, som er blevet udført efter en opfølgningstid på mindst 21 måneder. Kaplan-Meier-kurver for OS og PFS, som er baseret på den endelige analyse, er vist i figur 1 og 2.

Tabel 3: Effektresultater i KEYNOTE-006

**Endepunkt**	**Pembrolizumab 10 mg/kg hver 3. uge n=277**	**Pembrolizumab 10 mg/kg hver 2. uge n=279**	**Ipilimumab 3 mg/kg hver 3. uge n=278**
**OS**			
Antal (%) patienter med hændelse	119 (43%)	122 (44%)	142 (51%)
*Hazard ratio** (HR) (95% konfidensinterval, CI)	0,68 (0,53; 0,86)	0,68 (0,53; 0,87)	---
p-værdi†	< 0,001	< 0,001	---
Median i måneder (95% CI)	Ikke nået (24, NA)	Ikke nået (22, NA)	16 (14, 22)
**PFS**			
Antal (%) patienter med hændelse	183 (66%)	181 (65%)	202 (73%)
HR* (95% CI)	0,61 (0,50; 0,75)	0,61 (0,50; 0,75)	---
p-værdi†	< 0,001	< 0,001	---
Median i måneder (95% CI)	4,1 (2,9; 7,2)	5,6 (3,4; 8,2)	2,8 (2,8; 2,9)
**Bedste objektive respons**			
ORR % (95% CI)	36% (30, 42)	37% (31, 43)	13% (10, 18)
Komplet respons %	13%	12%	5%
Partielt respons %	23%	25%	8%
**Responsvarighed**‡			
Median i måneder (interval)	Ikke nået (2,0; 22,8+)	Ikke nået (1,8; 22,8+)	Ikke nået (1,1+; 23,8+)
% igangværende i måned 18	68%§	71%§	70%§

* *Hazard ratio* (pembrolizumab sammenlignet med ipilimumab) baseret på den stratificerede Cox *proportional hazard*-model
† Baseret på stratificeret log rank-test
‡ Baseret på patienter med bedste objektive respons, der er bekræftet som komplet eller partielt respons
§ Baseret på Kaplan-Meier-estimering
NA = Foreligger ikke

85

Figur 1: Kaplan-Meier-kurve for samlet overlevelse i hver behandlingsarm i KEYNOTE-006 (*intent to treat*-population)

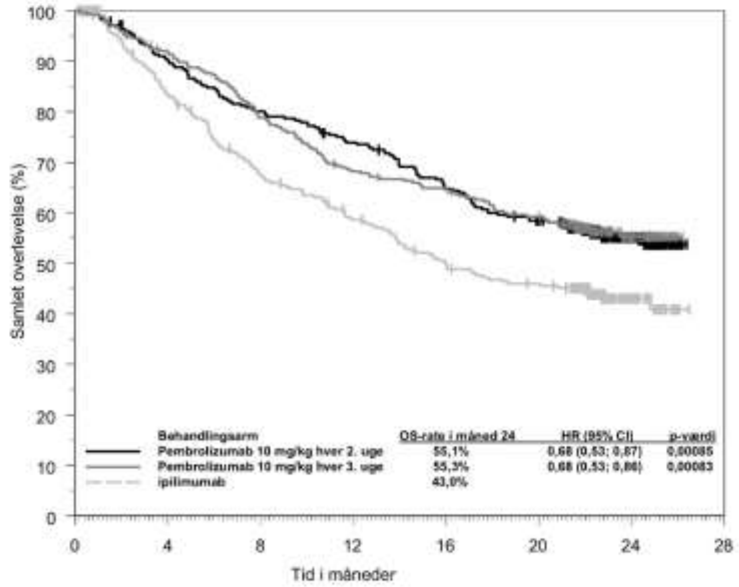

Antal i risikogruppe								
Pembrolizumab 10 mg/kg hver 2. uge:	279	249	221	202	176	156	44	0
Pembrolizumab 10 mg/kg hver 3. uge:	277	251	215	184	174	156	43	0
ipilimumab:	278	213	170	145	122	110	28	0

Figur 2: Kaplan-Meier-kurve for progressionsfri overlevelse i hver behandlingsarm i KEYNOTE-006 (*intent to treat*-population)

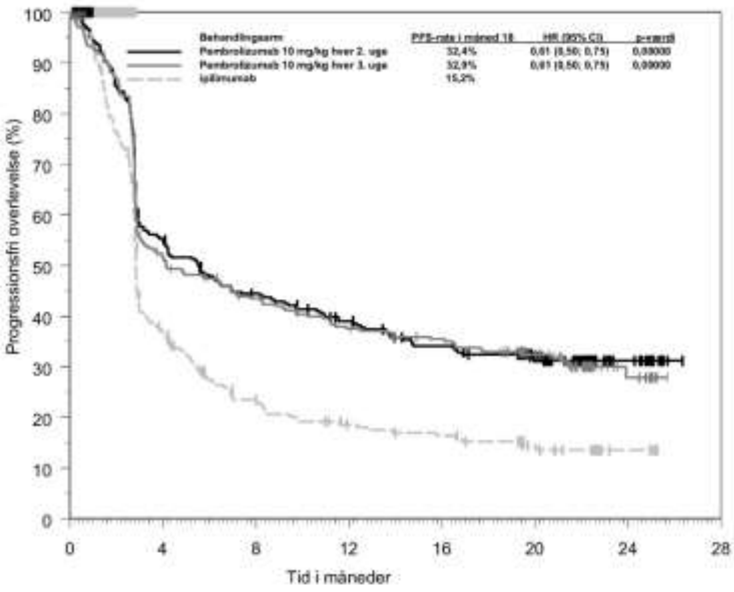

Antal i risikogruppe								
Pembrolizumab 10 mg/kg hver 2. uge:	279	148	116	98	82	52	16	0
Pembrolizumab 10 mg/kg hver 3. uge:	277	136	111	91	84	60	13	0
ipilimumab:	278	88	48	34	29	16	5	0

86

KEYNOTE-002: Kontrolleret studie med melanom-patienter, der tidligere har været behandlet med ipilimumab

Pembrolizumabs sikkerhed og virkning blev undersøgt i KEYNOTE-002, et dobbeltblindet, kontrolleret multicenterstudie af behandling af fremskredent melanom hos patienter, der tidligere har været behandlet med ipilimumab og med en BRAF- eller MEK-hæmmer, hvis der forekom en BRAF V600-mutation. Patienterne blev randomiseret i forholdet 1:1:1 til at få pembrolizumab i en dosis på 2 (n=180) eller 10 mg/kg (n=181) hver 3. uge eller kemoterapi (n=179; inkluderede dacarbazin, temozolomid, carboplatin, paclitaxel eller carboplatin+paclitaxel). Studiet ekskluderede patienter med autoimmun sygdom og patienter, som fik immunsuppressiv behandling. Yderligere eksklusionskriterier var en anamnese med svære eller livstruende immunrelaterede bivirkninger som følge af behandling med ipilimumab, defineret som en hvilken som helst grad 4-toksicitet eller en grad 3-toksicitet, som krævede behandling med kortikosteroider (> 10 mg prednison dagligt eller en dosis ækvivalent hermed) i mere end 12 uger; igangværende bivirkninger ≥ grad 2 fra tidligere behandling med ipilimumab; patienter, der tidligere har haft svær overfølsomhed over for andre monoklonale antistoffer; patienter med pneumonitis eller interstitiel lungesygdom i anamnesen; patienter med hiv-, hepatitis B- eller hepatitis C-infektion og ECOG-performance-status ≥ 2.

Patienterne blev behandlet med pembrolizumab indtil sygdomsprogression eller uacceptabel toksicitet. Klinisk stabile patienter med initial evidens på sygdomsprogression fik lov til at forblive i behandling, indtil sygdomsprogression var blevet bekræftet. Der blev foretaget vurdering af tumorstatus i uge 12, herefter hver 6. uge til og med uge 48 og derefter hver 12. uge. De patienter, som fik kemoterapi og oplevede uafhængigt verificeret sygdomsprogression efter den første planlagte sygdomsvurdering, kunne overkrydses til at få 2 mg/kg eller 10 mg/kg pembrolizumab hver 3. uge i et dobbeltblindet design.

Ud af 540 patienter var 61% mænd, 43% var ≥ 65 år (medianalderen var 62 år [interval: 15-89]), og 98% var kaukasere. 82% havde melanom i M1c-stadiet, 73% af patienterne havde forudgående fået mindst to, og 32% af patienterne havde fået tre eller flere systemiske behandlingsregimer for fremskredent melanom. 45% havde en ECOG-performance-status på 1, 40% havde forhøjet LDH, og 23% havde en BRAF-muteret tumor.

De primære resultatmål var PFS, baseret på IRO-vurdering i henhold til RECIST version 1.1, og OS. De sekundære resultatmål var ORR og responsvarighed. I tabel 4 opsummeres de centrale effektresultater efter den endelige analyse hos patienter, der tidligere har været behandlet med ipilimumab, og Kaplan-Meier-kurven for PFS er vist i figur 3. Begge behandlingsarme med pembrolizumab havde bedre (superior) PFS i forhold til kemoterapi, og der var ingen forskel mellem pembrolizumab-doserne. Der var ingen statistisk signifikant forskel mellem pembrolizumab og kemoterapi i den endelige OS-analyse, som ikke var korrigeret for den potentielt konfunderende effekt af overkrydsningen. 55% af de patienter, som var randomiseret til kemoterapi-armen, krydsede over og fik efterfølgende behandling med pembrolizumab.

Tabel 4: Effektresultater i KEYNOTE-002

Endepunkt	Pembrolizumab 2 mg/kg hver 3. uge n=180	Pembrolizumab 10 mg/kg hver 3. uge n=181	Kemoterapi n=179
**PFS**			
Antal (%) patienter med hændelse	150 (83%)	144 (80%)	172 (96%)
HR* (95% CI)	0,58 (0,46; 0,73)	0,47 (0,37; 0,60)	---
p-værdi†	< 0,001	< 0,001	---
Median i måneder (95% CI)	2,9 (2,8; 3,8)	3,0 (2,8; 5,2)	2,8 (2,6; 2,8)
**OS**			
Antal (%) patienter med hændelse	123 (68%)	117 (65%)	128 (72%)
HR* (95% CI)	0,86 (0,67; 1,10)	0,74 (0,57; 0,96)	---
p-værdi†	0,1173	0,0106‡	---
Median i måneder (95% CI)	13,4 (11,0; 16,4)	14,7 (11,3; 19,5)	11,0 (8,9; 13,8)
**Bedste objektive respons**			
ORR % (95% CI)	22% (16; 29)	28% (21; 35)	5% (2; 9)
Komplet respons %	3%	7%	0%
Partielt respons %	19%	20%	5%
**Responsvarighed**§			
Median i måneder (interval)	22,8 (1,4+; 25,3+)	Ikke nået (1,1+; 28,3+)	6,8 (2,8; 11,3)
% igangværende i måned 12	73%¶	79%¶	0%¶

* *Hazard ratio* (pembrolizumab sammenlignet med kemoterapi) baseret på den stratificerede Cox *proportional hazard*-model
† Baseret på stratificeret log rank-test
‡ Ikke statistisk signifikant efter justering for multiplicitet
§ Baseret på patienter med bedste objektive respons, der er bekræftet som komplet eller partielt respons fra den endelige analyse
¶ Baseret på Kaplan-Meier-estimering

88

Figur 3: Kaplan-Meier-kurve for progressionsfri overlevelse i hver behandlingsarm i KEYNOTE-002 (*intent to treat*-population)

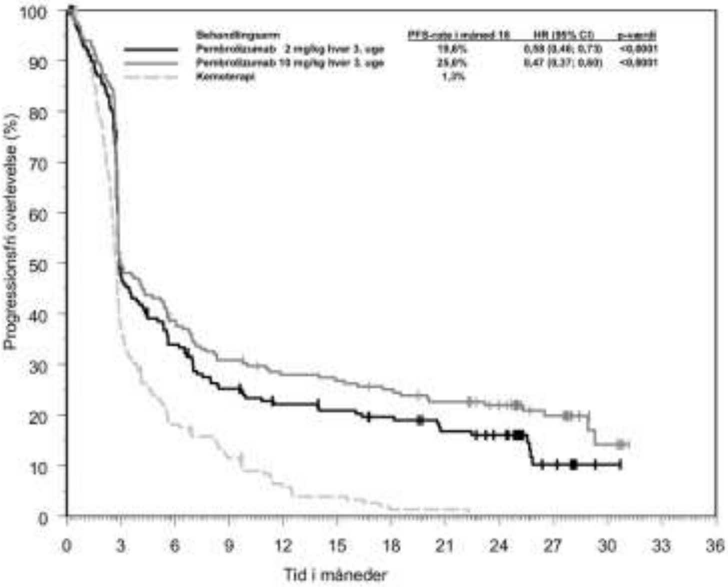

Antal i risikogruppe							
Pembrolizumab 2 mg/kg hver 3. uge:	180	59	36	29	19	1	0
Pembrolizumab 10 mg/kg hver 3. uge:	181	69	48	42	30	5	0
Kemoterapi:	179	31	9	2	1	0	0

KEYNOTE-001: Åbent studie med ipilimumab-naive og ipilimumab-erfarne melanom-patienter

Pembrolizumabs sikkerhed og virkning blev undersøgt hos patienter med fremskredent melanom i et ikke-kontrolleret, åbent studie, KEYNOTE-001. Virkningen blev vurderet hos 276 patienter fra to definerede kohorter, en kohorte, som inkluderede patienter, der tidligere har været behandlet med ipilimumab (og med en BRAF- eller MEK-hæmmer, hvis der forekom en BRAF V600-mutation), og den anden kohorte, som inkluderede patienter, der ikke tidligere har været behandlet med ipilimumab. Patienterne blev randomiseret til at få enten pembrolizumab i en dosis på 2 mg/kg hver 3. uge eller 10 mg/kg hver 3. uge. Patienterne blev behandlet med pembrolizumab indtil sygdomsprogression eller uacceptabel toksicitet. Klinisk stabile patienter med initial evidens på sygdomsprogression fik lov til at forblive i behandling, indtil sygdomsprogression var blevet bekræftet. Eksklusionskriterierne var de samme som i KEYNOTE-002.

Af de 89 patienter, som fik 2 mg/kg pembrolizumab, og som tidligere havde været behandlet med ipilimumab, var 53% mænd, 33% var ≥ 65 år, og medianalderen var 59 år (interval: 18-88). Alle på nær to patienter var kaukasere. 84% havde melanom i M1c-stadiet, og 8% af patienterne havde hjernemetastaser i anamnesen. 70% af patienterne havde forudgående fået mindst to, og 35% af patienterne havde fået tre eller flere systemiske behandlingsregimer for fremskredent melanom. BRAF-mutation blev rapporteret hos 13% af studiepopulationen. Alle patienter med BRAF-muteret tumor havde tidligere været i behandling med en BRAF-hæmmer.

Af de 51 patienter, som fik 2 mg/kg pembrolizumab, og som ikke tidligere havde været behandlet med ipilimumab, var 63% mænd, 35% var ≥ 65 år, og medianalderen var 60 år (interval: 35-80). Alle på nær en patient var kaukasere. 63% havde melanom i M1c-stadiet, og 2% af patienterne havde hjernemetastaser i anamnesen. 45% havde ikke tidligere fået behandling for fremskredent melanom. BRAF-mutation blev rapporteret hos 20 (39%) patienter. Blandt patienterne med BRAF-muteret tumor havde 10 (50%) tidligere været i behandling med en BRAF-hæmmer.

Det primære resultatmål var ORR, baseret på en vurdering ved IRO i henhold til RECIST 1.1. De sekundære resultatmål var sygdomskontrolrate (DCR, herunder komplet respons, partielt respons og stabil sygdom), responsvarighed, PFS og OS. Tumorrespons blev vurderet hver 12. uge. I tabel 5 opsummeres de centrale effektresultater hos patienter, der tidligere har været eller ikke tidligere har været behandlet med ipilimumab, som fik pembrolizumab ved en dosis på 2 mg/kg baseret på en opfølgningstid på mindst 30 måneder for alle patienter.

Tabel 5: Effektresultater i KEYNOTE-001

**Endepunkt**	**Pembrolizumab 2 mg/kg hver 3. uge hos ipilimumab-erfarne patienter n=89**	**Pembrolizumab 2 mg/kg hver 3. uge hos ipilimumab-naive patienter n=51**
**Bedste objektive respons* bedømt ved IRO†**		
ORR %, (95% CI)	26% (17; 36)	35% (22; 50)
Komplet respons	7%	12%
Partielt respons	19%	24%
Sygdomskontrolrate %‡	48%	49%
**Responsvarighed§**		
Median i måneder (interval)	30,5 (2,8+; 30,6+)	27,4 (1,6+; 31,8+)
% igangværende i måned 24¶	75%	71%
**PFS**		
Median i måneder (95% CI)	4,9 (2,8; 8,3)	4,7 (2,8; 13,8)
PFS-rate i måned 12	34%	38%
**OS**		
Median i måneder (95% CI)	18,9 (11, foreligger ikke)	28,0 (14, foreligger ikke)
OS-rate i måned 24	44%	56%

* Inkluderer patienter uden målbar sygdom ved *baseline* ved uafhængig radiologisk gennemgang
† IRO = *Integrated Radiology and Oncology* vurdering i henhold til RECIST 1.1
‡ Baseret på stabil sygdom eller bedre som bedste respons
§ Baseret på patienter med bekræftet respons ved uafhængig gennemgang, startende fra den dato, hvor respons blev registreret første gang; n=23 for ipilimumab-erfarne patienter; n=18 for ipilimumab-naive patienter
¶ Baseret på Kaplan-Meier-estimering

Resultaterne for ipilimumab-erfarne patienter (n=84) og for ipilimumab-naive patienter (n=52), som fik 10 mg/kg pembrolizumab hver 3. uge, var sammenlignelige med resultaterne for patienter, som fik 2 mg/kg pembrolizumab hver 3. uge.

Analyser af subgrupper

BRAF-mutationstatus ved melanom
Der blev udført en subgruppeanalyse som en del af den endelige analyse i KEYNOTE-002 hos patienter, som havde BRAF vildtype (n=414; 77%) eller BRAF-mutation med forudgående BRAF-behandling (n=126; 23%) som opsummeret i tabel 6.

Tabel 6: Effektresultater ud fra BRAF-mutationsstatus i KEYNOTE-002

	BRAF vildtype		BRAF-mutation med forudgående BRAF-behandling	
**Endepunkt**	**Pembrolizumab 2 mg/kg hver 3. uge (n=136)**	**Kemoterapi (n=137)**	**Pembrolizumab 2 mg/kg hver 3. uge (n=44)**	**Kemoterapi (n=42)**
HR* for PFS (95% CI)	0,50 (0,39; 0,66)	---	0,79 (0,50; 1,25)	---
HR* for OS (95% CI)	0,78 (0,58; 1,04)	---	1,07 (0,64; 1,78)	---
ORR %	26%	6%	9%	0%

* *Hazard ratio* (pembrolizumab sammenlignet med kemoterapi) baseret på den stratificerede Cox *proportional hazard*-model

Der blev udført en subgruppeanalyse som en del af den endelige analyse i KEYNOTE-006 hos patienter, som var enten BRAF vildtype (n=525; 63%), BRAF-mutation uden forudgående BRAF-behandling (n=163; 20%) eller BRAF-mutation med forudgående BRAF-behandling (n=139; 17%) som opsummeret i tabel 7.

Tabel 7: Effektresultater ud fra BRAF-mutationsstatus i KEYNOTE-006

	BRAF vildtype		BRAF-mutation uden forudgående BRAF-behandling		BRAF-mutation med forudgående BRAF-behandling	
**Endepunkt**	**Pembrolizumab 10 mg/kg hver 2. eller 3. uge (puljet)**	**Ipilimumab (n=170)**	**Pembrolizumab 10 mg/kg hver 2. eller 3. uge (puljet)**	**Ipilimumab (n=55)**	**Pembrolizumab 10 mg/kg hver 2. eller 3. uge (puljet)**	**Ipilimumab (n=52)**
HR* for PFS (95% CI)	0,61 (0,49; 0,76)	---	0,52 (0,35; 0,78)	---	0,76 (0,51; 1,14)	---
HR* for OS (95% CI)	0,68 (0,52; 0,88)	---	0,70 (0,40; 1,22)	---	0,66 (0,41; 1,04)	---
ORR %	38%	14%	41%	15%	24%	10%

* *Hazard ratio* (pembrolizumab sammenlignet med ipilimumab) baseret på den stratificerede Cox *proportional hazard*-model

PD-L1-status ved melanom

Der blev udført en subgruppeanalyse som en del af den endelige analyse i KEYNOTE-002 hos patienter, der var PD-L1-positive (PD-L1-ekspression i ≥ 1% af tumorcellerne og tumor-associerede immunceller i forhold til alle levedygtige tumorceller – MEL-score) *versus* PD-L1-negative. PD-L1-ekspression blev testet retrospektivt ved en immunhistokemisk analyse med 22C3 anti-PD-L1-antistof. Blandt de patienter, som var evaluerbare for PD-L1-ekspression (79%), var 69% (n=294) PD-L1-positive, og 31% (n=134) var PD-L1-negative. I tabel 8 opsummeres effektresultaterne ud fra PD-L1-ekspression.

Tabel 8: Effektresultater ud fra PD-L1-ekspression i KEYNOTE-002

**Endepunkt**	**Pembrolizumab 2 mg/kg hver 3. uge**	**Kemoterapi**	**Pembrolizumab 2 mg/kg hver 3. uge**	**Kemoterapi**
	**PD-L1-positiv**		**PD-L1-negativ**	
HR* for PFS (95% CI)	0,55 (0,40; 0,76)	---	0,81 (0,50; 1,31)	---
HR* for OS (95% CI)	0,90 (0,63; 1,28)	---	1,18 (0,70; 1,99)	---
ORR %	25%	4%	10%	8%

* *Hazard ratio* (pembrolizumab sammenlignet med kemoterapi) baseret på den stratificerede Cox *proportional hazard*-model

Der blev udført en subgruppeanalyse som en del af den endelige analyse i KEYNOTE-006 hos patienter, der var PD-L1-positive (n=671; 80%) *versus* PD-L1-negative (n=150; 18%). Hos de patienter, som var evaluerbare for PD-L1-ekspression (98%), var 82% PD-L1-positive, og 18% var PD-L1-negative. I tabel 9 opsummeres effektresultaterne ud fra PD-L1-ekspression.

Tabel 9: Effektresultater ud fra PD-L1-ekspression i KEYNOTE-006

Endepunkt	Pembrolizumab 10 mg/kg hver 2. eller 3. uge (puljet)	Ipilimumab	Pembrolizumab 10 mg/kg hver 2. eller 3. uge (puljet)	Ipilimumab
	PD-L1-positiv		PD-L1-negativ	
HR* for PFS (95% CI)	0,53 (0,44; 0,65)	---	0,87 (0,58; 1,30)	---
HR* for OS (95% CI)	0,63 (0,50; 0,80)	---	0,76 (0,48; 1,19)	---
ORR %	40%	14%	24%	13%

* *Hazard ratio* (pembrolizumab sammenlignet med ipilimumab) baseret på den stratificerede Cox *proportional hazard*-model

Okulært melanom

Hos 20 forsøgspersoner med okulært melanom, som var inkluderet i KEYNOTE-001, blev der ikke rapporteret objektivt respons. Stabil sygdom blev rapporteret hos 6 patienter.

KEYNOTE-054: Placebokontrolleret studie med adjuverende behandling af patienter med komplet resekteret melanom

Pembrolizumabs virkning blev vurderet i KEYNOTE-054, et randomiseret, dobbeltblindet, placebokontrolleret multicenterstudie hos patienter med komplet resekteret stadie IIIA- (> 1 mm lymfeknudemetastaser), IIIB- eller IIIC-melanom. I alt 1.019 voksne patienter blev randomiseret i forholdet 1:1 til at få pembrolizumab 200 mg hver 3. uge (n=514) eller placebo (n=505) i en periode på op til 1 år indtil sygdomsrecidiv eller uacceptabel toksicitet. Randomiseringen blev stratificeret ud fra stadie (IIIA *versus* IIIB *versus* IIIC 1-3 positive lymfeknuder *versus* IIIC ≥ 4 positive lymfeknuder) i henhold til *American Joint Committee on Cancer* (AJCC), 7. udgave, og geografisk område (Nordamerika, de europæiske lande, Australien og andre udvalgte lande). Patienterne skal have fået foretaget lymfeknude-dissektion, og hvis det er indiceret, strålebehandling inden for 13 uger før påbegyndelse af behandlingen. Patienter med aktiv autoimmun sygdom eller lidelser, som krævede behandling med immunsuppressiva, eller slimhindemelanom eller okulært melanom var uegnede til studiet. Patienter, som havde modtaget anden forudgående behandling for melanom end kirurgisk indgreb eller interferon for tykke primære melanomer uden tegn på lymfeknudeinvolvering, var uegnede til studiet. Patienterne fik foretaget billeddiagnostisk undersøgelse hver 12. uge efter den første dosis pembrolizumab i løbet af de første 2 år, herefter hver 6. måned fra år 3 til 5 og herefter årligt.

Hos de 1.019 patienter var *baseline*-karakteristika: medianalder 54 år (25% i alderen 65 år eller ældre); 62% mænd og ECOG-performance-status 0 (94%) og 1 (6%). 16% havde stadie IIIA; 46% havde stadie IIIB; 18% havde stadie IIIC (1-3 positive lymfeknuder), og 20% havde stadie IIIC (≥ 4 positive lymfeknuder); 50% var positive for BRAF V600-mutation og 44% var BRAF vildtype. PD-L1-ekspression blev testet retrospektivt ved en immunhistokemisk analyse med 22C3 anti-PD-L1-antistof; 84% af patienterne havde PD-L1-positivt melanom (PD-L1-ekspression hos ≥ 1% af tumor- og tumorassocierede immunceller i forhold til alle levedygtige tumorceller). Der blev anvendt det samme scoringssystem ved metastatisk melanom (MEL-score).

Det primære resultatmål var investigator-vurderet recidivfri overlevelse (RFS) i hele populationen og i populationen med PD-L1-positive tumorer, hvor RFS blev defineret som tiden mellem datoen for randomisering og datoen for første recidiv (lokale, regionale eller fjernmetastaser) eller død, afhængigt af, hvad der indtræffer først. Studiet viste en statistisk signifikant forbedring i RFS for patienter randomiseret til armen med pembrolizumab sammenlignet med placebo ved den præspecificerede interimanalyse. Effektresultater baseret på yderligere 7 måneders opfølgning er opsummeret i tabel 10 og i figur 4.

Tabel 10: Effektresultater i KEYNOTE-054

**Endepunkt**	**KEYTRUDA 200 mg hver 3. uge n=514**	**Placebo n=505**
Antal (%) patienter med hændelse	158 (31%)	246 (49%)
Median i måneder (95% CI)	NR	21,7 (17,1; NR)
HR* (98% CI)	0,56 (0,44; 0,72)	
p-værdi (stratificeret log-rank)	< 0,0001	
**RFS ved måned 6**		
RFS-rate	82%	73%
**RFS ved måned 12**		
RFS-rate	76%	61%
**RFS ved måned 18**		
RFS-rate	72%	54%

* Baseret på den stratificerede Cox *proportional hazard*-model
NR = Ikke nået

KEYNOTE-054 inkluderede patienter i henhold til AJCC, 7. udgave, og en subgruppeanalyse af RFS i henhold til AJCC, 8. udgave, blev udført efter at RFS-studieresultaterne blev rapporteret. Der blev påvist en statistisk signifikant forbedring i RFS for patienter randomiseret til armen med pembrolizumab sammenlignet med placebo i den samlede population på tværs af resekteret stadie III-melanom i henhold til AJCC, 7. udgave. Stadie IIIA-melanom i henhold til AJCC, 8. udgave, identificerer en patientpopulation med en bedre prognose sammenlignet med stadie IIIA i henhold til AJCC, 7. udgave. I henhold til AJCC-stadieklassificering, 8. udgave, blev i alt 82 forsøgspersoner klassificeret som stadie IIIA; 42 i armen med pembrolizumab og 40 i placebo-armen; med i alt 13 RFS-hændelser; 6 i armen med pembrolizumab og 7 i placebo-armen. Der er begrænsede data vedrørende forsøgspersoner med stadie IIIA i henhold til AJCC, 8. udgave, på tidspunktet for RFS-analysen.

Figur 4: Kaplan-Meier-kurve for recidivfri overlevelse i hver behandlingsarm i KEYNOTE-054 (*intent to treat*-population)

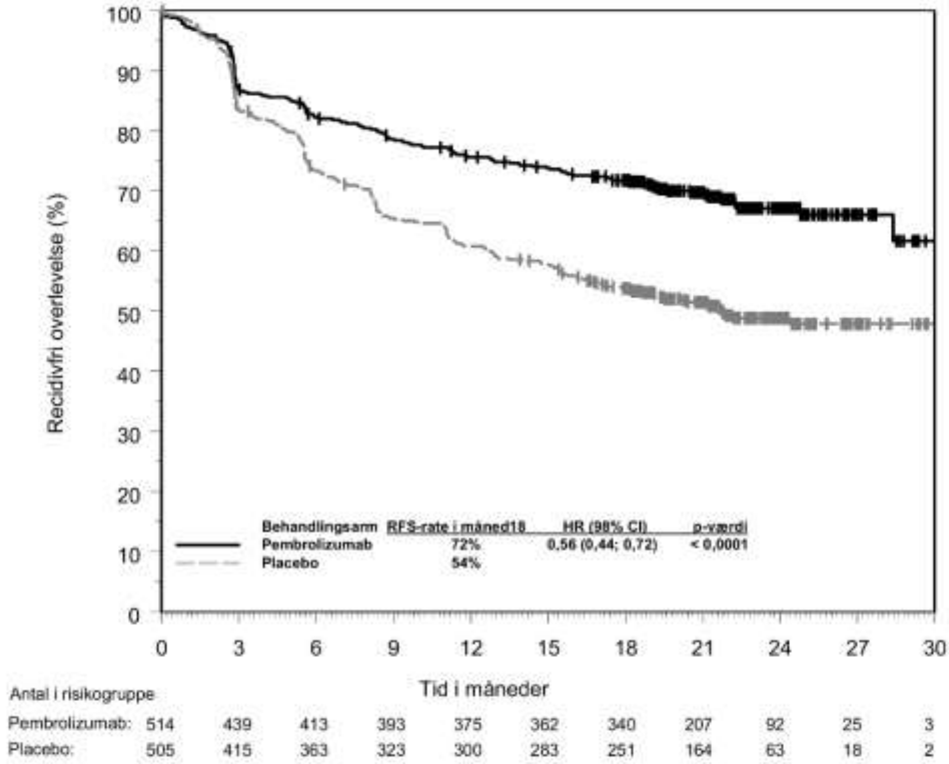

Selvom analysen hos patienter med PD-L1-positive tumorer var et co-primært endepunkt, blev der udført prædefinerede subgruppeanalyser hos patienter, hvis tumorer var PD-L1-negative, positive eller negative for BRAF-mutation. I tabel 11 opsummeres effektresultater ud fra PD-L1-ekspression og BRAF-mutationsstatus.

Tabel 11: Effektresultater ud fra PD-L1-ekspression og BRAF-mutationsstatus i KEYNOTE-054

Endepunkt	Pembrolizumab 200 mg hver 3. uge	Placebo	Pembrolizumab 200 mg hver 3. uge	Placebo
	PD-L1-positiv		PD-L1-negativ	
	n=428	n=425	n=59	n=57
HR* for RFS (95% CI)	0,54 (0,42; 0,69)	---	0,47 (0,26; 0,85)	---
RFS-rate ved måned 6	84%	75%	81%	64%
	Positiv for BRAF-mutation		Negativ for BRAF-mutation	
	n=245	n=262	n=233	n=214
HR* for RFS (95% CI)	0,49 (0,36; 0,67)	---	0,64 (0,47; 0,87)	---
RFS-rate ved måned 6	83%	73%	80%	72%

*Baseret på den stratificerede Cox *proportional hazard*-model

NSCLC

KEYNOTE-024: Kontrolleret studie med behandlingsnaive NSCLC-patienter

Pembrolizumabs sikkerhed og virkning blev undersøgt i KEYNOTE-024, et åbent, kontrolleret multicenterstudie hos tidligere ubehandlede patienter med metastatisk NSCLC. Patienterne havde PD-L1-ekspression med TPS ≥ 50% baseret på PD-L1 IHC 22C3 pharmDx™-analysen. Patienterne blev randomiseret i forholdet 1:1 til pembrolizumab i en dosis på 200 mg hver 3. uge (n=154) eller investigators valg af platinbaseret kemoterapi (inklusive pemetrexed+carboplatin, pemetrexed+cisplatin, gemcitabin+cisplatin, gemcitabin+carboplatin eller paclitaxel+carboplatin; n=151. Patienter med ikke-planocellulært karcinom kunne få vedligeholdelsesbehandling med pemetrexed). Patienterne blev behandlet med pembrolizumab indtil uacceptabel toksicitet eller sygdomsprogression. Behandlingen kunne fortsætte efter sygdomsprogression, hvis patienten var klinisk stabil, og investigator mente, at patienten havde en klinisk fordel. Patienter uden sygdomsprogression kunne behandles i op til 24 måneder. Patienter med EGFR- eller ALK-mutationer i tumor, autoimmun sygdom, der havde krævet systemisk behandling inden for de seneste 2 år, lidelser, som krævede behandling med immunsuppressiva, og patienter, som havde fået mere end 30 Gy torakal strålebehandling inden for de forudgående 26 uger, blev ekskluderet fra studiet. Tumorstatus blev vurderet hver 9. uge. Patienter i behandling med kemoterapi, som oplevede uafhængigt verificeret sygdomsprogression, kunne krydse over til pembrolizumab.

Hos de 305 patienter i KEYNOTE-024 var *baseline*-karakteristika: medianalder 65 år (54% ≥ 65 år), 61% mænd, 82% kaukasere, 15% asiater og ECOG-performance-status 0 og 1 hos henholdsvis 35% og 65%. Sygdomskarakteristika var planocellulær (18%) og ikke-planocellulær (82%), M1-stadie (99%) og hjernemetastaser (9%).

Det primære resultatmål var PFS baseret på BICR (*blinded independent central review*)-vurdering i henhold til RECIST 1.1. Sekundære resultatmål var OS og ORR (baseret på BICR-vurdering i henhold til RECIST 1.1). I tabel 12 opsummeres de vigtigste effektresultater for hele *intent to treat* (ITT)-populationen. PFS- og ORR-resultater er rapporteret fra en interimanalyse med en median opfølgningstid på 11 måneder. OS-resultater er rapporteret fra den endelige analyse med en median opfølgningstid på 25 måneder.

Tabel 12: Effektresultater i KEYNOTE-024

Endepunkt	Pembrolizumab 200 mg hver 3. uge n=154	Kemoterapi n=151
**PFS**		
Antal (%) patienter med hændelse	73 (47%)	116 (77%)
HR* (95% CI)	0,50 (0,37; 0,68)	
p-værdi†	< 0,001	
Median i måneder (95% CI)	10,3 (6,7; NA)	6,0 (4,2; 6,2)
**OS**		
Antal (%) patienter med hændelse	73 (47%)	96 (64%)
HR* (95% CI)	0,63 (0,47; 0,86)	
p-værdi†	0,002	
Median i måneder (95% CI)	30,0 (18,3; NA)	14,2 (9,8; 19,0)
**Objektiv responsrate**		
ORR % (95% CI)	45% (37; 53)	28% (21; 36)
Komplet respons %	4%	1%
Partielt respons %	41%	27%
**Responsvarighed‡**		
Median i måneder (interval)	Ikke nået (1,9+; 14,5+)	6,3 (2,1+; 12,6+)
% med varighed ≥ 6 måneder	88%§	59%¶

* *Hazard ratio* (pembrolizumab sammenlignet med kemoterapi) baseret på den stratificerede Cox *proportional hazard*-model
† Baseret på stratificeret log rank-test
‡ Baseret på patienter med bedste objektive respons, der er bekræftet komplet respons eller partielt respons
§ Baseret på Kaplan-Meier-estimater; inkluderer 43 patienter med respons på 6 måneder eller længere
¶ Baseret på Kaplan-Meier-estimater; inkluderer 16 patienter med respons på 6 måneder eller længere
NA = Foreligger ikke

96

Figur 5: Kaplan-Meier-kurve for progressionsfri overlevelse i hver behandlingsarm i KEYNOTE-024 (*intent to treat*-population)

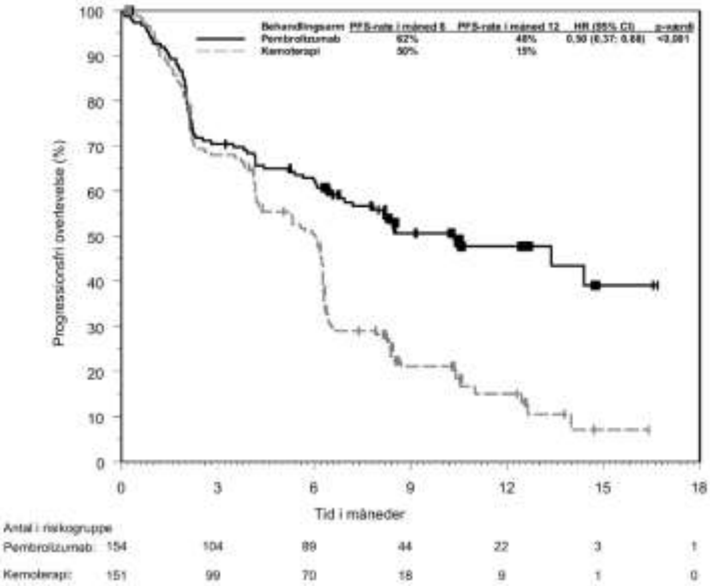

Figur 6: Kaplan-Meier-kurve for samlet overlevelse i hver behandlingsarm i KEYNOTE-024 (*intent to treat*-population)

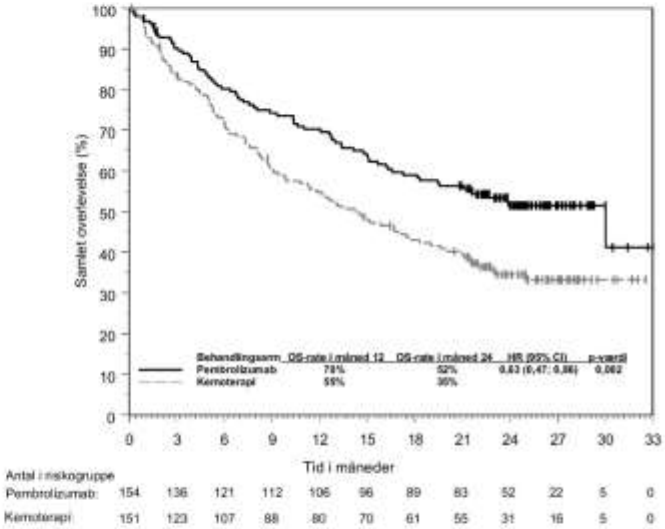

I en subgruppeanalyse viste pembrolizumab en reduceret fordel med hensyn til overlevelse sammenlignet med kemoterapi hos de få patienter, som aldrig havde røget. På grund af det lave antal patienter kan der dog ikke drages nogle definitive konklusioner på baggrund af disse data.

97

KEYNOTE-189: Kontrolleret studie af kombinationsbehandling hos behandlingsnaive patienter med ikke-planocellulær NSCLC

Pembrolizumabs virkning i kombination med pemetrexed og platinbaseret kemoterapi blev undersøgt i et randomiseret, aktivt kontrolleret, dobbeltblindet multicenterstudie, KEYNOTE-189. De centrale inklusionskriterier var metastatisk ikke-planocellulær NSCLC, ingen tidligere systemisk behandling for metastatisk NSCLC og ingen EGFR- eller ALK-mutationer i tumor. Patienter med autoimmun sygdom, der havde krævet systemisk behandling inden for de seneste 2 år; lidelser, som krævede behandling med immunsuppressiva; eller patienter, som havde fået mere end 30 Gy torakal strålebehandling inden for de forudgående 26 uger, blev ekskluderet fra studiet. Patienterne blev randomiseret i forholdet 2:1 til behandling med et af følgende regimer:

- Pembrolizumab 200 mg med pemetrexed 500 mg/m^2 og investigators valg af cisplatin 75 mg/m^2 eller carboplatin AUC 5 mg/ml/min intravenøst hver 3. uge i 4 serier efterfulgt af pembrolizumab 200 mg og pemetrexed 500 mg/m^2 intravenøst hver 3. uge (n=410).
- Placebo med pemetrexed 500 mg/m^2 og investigators valg af cisplatin 75 mg/m^2 eller carboplatin AUC 5 mg/ml/min intravenøst hver 3. uge i 4 serier efterfulgt af placebo og pemetrexed 500 mg/m^2 intravenøst hver 3. uge (n=206).

Behandling med pembrolizumab fortsatte indtil sygdomsprogression defineret i henhold til RECIST 1.1 efter investigators vurdering, uacceptabel toksicitet eller i højst 24 måneder. Administration af pembrolizumab var tilladt efter sygdomsprogression i henhold til RECIST baseret på BICR-vurdering eller efter seponering af pemetrexed, hvis patienten var klinisk stabil, og investigator vurderede, at patienten havde en klinisk fordel. Hos patienter, der gennemførte 24 måneders behandling eller som havde komplet respons, kunne behandlingen med pembrolizumab genoptages ved sygdomsprogression og blive administreret i op til yderligere 1 år. Vurdering af tumorstatus blev foretaget i uge 6 og uge 12 og efterfølgende hver 9. uge derefter. Patienter, der fik placebo plus kemoterapi, som oplevede uafhængigt verificeret sygdomsprogression, blev tilbudt pembrolizumab som monoterapi.

Hos de 616 patienter i KEYNOTE-189 var *baseline*-karakteristika: medianalder 64 år (49% i alderen 65 år eller ældre); 59% mænd; 94% kaukasere og 3% asiater; henholdsvis 43% og 56% med ECOG-performance-status 0 eller 1; 31% PD-L1-negative (TPS < 1%); og 18% med behandlede eller ikke behandlede hjernemetastaser ved *baseline*. I alt 67 patienter i placebo plus kemoterapiarmen krydsede over til pembrolizumab som monoterapi på tidspunktet for sygdomsprogression, og yderligere 18 patienter fik en *checkpoint*-hæmmer som efterfølgende behandling.

De primære resultatmål var OS og PFS (BICR-vurdering i henhold til RECIST 1.1). De sekundære resultatmål var ORR og responsvarighed baseret på BICR-vurdering i henhold til RECIST 1.1. Den mediane opfølgningstid var 10,5 måneder (interval: 0,2 til 20,4 måneder). I tabel 13 opsummeres de vigtigste effektresultater. Kaplan-Meier-kurver for OS og PFS er vist i figur 7 og 8.

Tabel 13: Effektresultater i KEYNOTE-189

Endepunkt	Pembrolizumab + pemetrexed + platinbaseret kemoterapi n=410	Placebo + pemetrexed + platinbaseret kemoterapi n=206
**OS**		
Antal (%) patienter med hændelse	127 (31%)	108 (52%)
HR* (95% CI)	0,49 (0,38; 0,64)	
p-værdi†	< 0,00001	
Median i måneder (95% CI)	Ikke nået (NA; NA)	11,3 (8,7; 15,1)
**PFS**		
Antal (%) patienter med hændelse	244 (60%)	166 (81%)
HR* (95% CI)	0,52 (0,43; 0,64)	
p-værdi†	< 0,00001	
Median i måneder (95% CI)	8,8 (7,6; 9,2)	4,9 (4,7; 5,5)
**Objektiv responsrate**		
ORR‡ % (95% CI)	48% (43; 53)	19% (14; 25)
Komplet respons %	0,5%	0,5%
Partielt respons %	47%	18%
p-værdi§	< 0,0001	
**Responsvarighed**		
Median i måneder (interval)	11,2 (1,1+; 18,0+)	7,8 (2,1+; 16,4+)
% med varighed ≥ 6 måneder¶	81%	63%
% med varighed ≥ 9 måneder¶	60%	44%

* Baseret på den stratificerede Cox *proportional hazard*-model
† Baseret på stratificeret log rank-test
‡ Baseret på patienter med bedste objektive respons, der er bekræftet komplet respons eller partielt respons
§ Baseret på Miettinen- og Nurminen-metoden stratificeret ud fra PD-L1-status, platinbaseret kemoterapi og rygestatus
¶ Baseret på Kaplan-Meier-estimering
NA = Foreligger ikke

99

Figur 7: Kaplan-Meier-kurve for samlet overlevelse i hver behandlingsarm i KEYNOTE-189 (*intent to treat*-population)

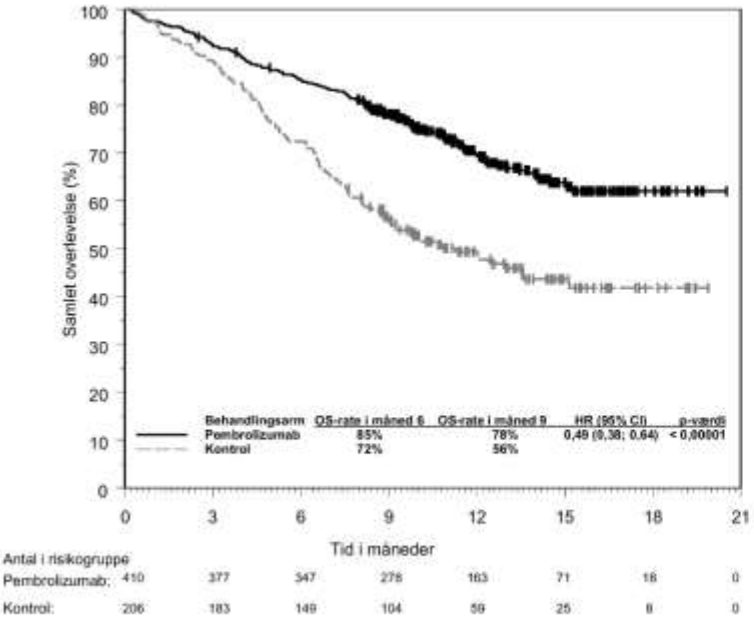

Figur 8: Kaplan-Meier-kurve for progressionsfri overlevelse i hver behandlingsarm i KEYNOTE-189 (*intent to treat*-population)

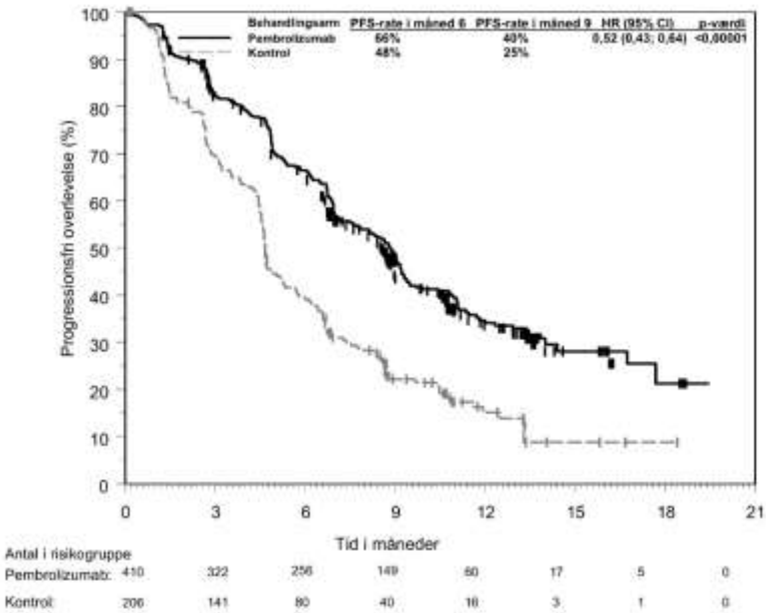

100

Der blev udført en analyse i KEYNOTE-189 hos patienter, som havde PD-L1 TPS < 1% [pembrolizumab-kombination: n=127 (31%) *versus* kemoterapi: n=63 (31%)], TPS 1-49% [pembrolizumab-kombination: n=128 (31%) *versus* kemoterapi: n=58 (28%)] eller TPS ≥ 50% [pembrolizumab-kombination: n=132 (32%) *versus* kemoterapi: n=70 (34%)] (se tabel 14).

Tabel 14: Effektresultater ud fra PD-L1-ekspression i KEYNOTE-189

Endepunkt	Pembrolizumab kombinations-behandling	Kemoterapi	Pembrolizumab kombinations-behandling	Kemoterapi	Pembrolizumab kombinations-behandling	Kemoterapi
	TPS < 1%		TPS 1 til 49%		TPS ≥ 50%	
HR* for OS (95% CI)	0,59 (0,38; 0,92)		0,55 (0,34; 0,90)		0,42 (0,26; 0,68)	
HR* for PFS (95% CI)	0,75 (0,53; 1,05)		0,55 (0,37; 0,81)		0,36 (0,25; 0,52)	
ORR %	32%	14%	48%	21%	61%	23%

* *Hazard ratio* (pembrolizumab kombinationsbehandling sammenlignet med kemoterapi) baseret på den stratificerede Cox *proportional hazard*-model

I alt 57 NSCLC-patienter ≥ 75 år var inkluderet i KEYNOTE-189-studiet (35 i kombinationsbehandlingen med pembrolizumab og 22 i kontrolgruppen). En HR = 2,09 [95% CI 0,84; 5,23] for OS og HR = 1,73 [95% CI 0,77; 3,90] for PFS for kombinationsbehandlingen med pembrolizumab *versus* kemoterapi blev rapporteret i denne studiesubgruppe. Data vedrørende pembrolizumabs sikkerhed og virkning i kombination med platinbaseret kemoterapi er begrænset i denne patientpopulation.

KEYNOTE-407: Kontrolleret studie af kombinationsbehandling hos behandlingsnaive patienter med planocellulær NSCLC

Pembrolizumabs virkning i kombination med carboplatin og enten paclitaxel eller nab-paclitaxel blev undersøgt i KEYNOTE-407, et randomiseret, dobbeltblindet, placebokontrolleret multicenterstudie. De centrale inklusionskriterier var metastatisk planocellulær NSCLC, uanset PD-L1-tumorekspressionsstatus, og ingen tidligere systemisk behandling for metastatisk sygdom. Patienter med autoimmun sygdom, der havde krævet systemisk behandling inden for de seneste 2 år; lidelser, som krævede behandling med immunsuppressiva; eller patienter, som havde fået mere end 30 Gy torakal strålebehandling inden for de forudgående 26 uger, blev ekskluderet fra studiet. Randomiseringen blev stratificeret ud fra PD-L1-tumorekspression (TPS < 1% [negativ] *versus* TPS ≥ 1%), investigators valg af paclitaxel eller nab-paclitaxel og geografisk område (Østasien *versus* ikke Østasien). Patienterne blev randomiseret i forholdet 1:1 til en af følgende behandlingsarme med intravenøs infusion:

- Pembrolizumab 200 mg og carboplatin AUC 6 mg/ml/min på dag 1 i hver 21-dages serie i 4 serier, og paclitaxel 200 mg/m^2 på dag 1 i hver 21-dages serie i 4 serier eller nab-paclitaxel 100 mg/m^2 på dag 1, 8 og 15 i hver 21-dages serie i 4 serier, efterfulgt af pembrolizumab 200 mg hver 3. uge. Pembrolizumab blev administreret før kemoterapi på dag 1.
- Placebo og carboplatin AUC 6 mg/ml/min på dag 1 i hver 21-dages serie i 4 serier og paclitaxel 200 mg/m^2 på dag 1 i hver 21-dages serie i 4 serier eller nab-paclitaxel 100 mg/m^2 på dag 1, 8 og 15 i hver 21-dages serie i 4 serier, efterfulgt af placebo hver 3. uge.

Behandling med pembrolizumab eller placebo fortsatte indtil sygdomsprogression i henhold til RECIST 1.1 baseret på BICR-vurdering, uacceptabel toksicitet eller i højst 24 måneder. Administration af pembrolizumab var tilladt efter sygdomsprogression i henhold til RECIST, hvis patienten var klinisk stabil, og investigator vurderede, at patienten havde en klinisk fordel.

Patienterne i placeboarmen blev tilbudt pembrolizumab som monoterapi på tidspunktet for sygdomsprogression.

Vurdering af tumorstatus blev foretaget hver 6. uge til og med uge 18, hver 9. uge til og med uge 45 og hver 12. uge derefter.

I alt 559 patienter blev randomiseret. Karakteristika for studiepopulationen var: medianalder på 65 år (interval: 29 til 88); 55% i alderen 65 år eller ældre; 81% mænd; 77% kaukasere; ECOG-performance-

status 0 (29%) og 1 (71%); og 8% med behandlede hjernemetastaser ved *baseline*. 35% havde PD-L1-tumorekspression TPS < 1% [negativ]; 19% var østasiatere og 60% fik paclitaxel.

De primære resultatmål var OS og PFS (BICR-vurdering i henhold til RECIST 1.1). De sekundære resultatmål var ORR og responsvarighed baseret på BICR-vurdering i henhold til RECIST 1.1. Den mediane opfølgningstid var 7,8 måneder (interval: 0,1 til 19,1 måneder). I tabel 15 opsummeres de vigtigste effektresultater. Kaplan-Meier-kurver for OS og PFS er vist i figur 9 og 10.

Tabel 15: Effektresultater i KEYNOTE-407

**Endepunkt**	**Pembrolizumab Carboplatin Paclitaxel/nab-paclitaxel n=278**	**Placebo Carboplatin Paclitaxel/nab-paclitaxel n=281**
**OS**		
Antal hændelser (%)	85 (31%)	120 (43%)
Median i måneder (95% CI)	15,9 (13,2; NA)	11,3 (9,5; 14,8)
HR* (95% CI)	0,64 (0,49; 0,85)	
p-værdi†	0,0008	
**PFS**		
Antal hændelser (%)	152 (55%)	197 (70%)
Median i måneder (95% CI)	6,4 (6,2; 8,3)	4,8 (4,3; 5,7)
HR* (95% CI)	0,56 (0,45; 0,70)	
p-værdi†	< 0,0001	
**Objektiv responsrate**		
ORR (95% CI)	58% (52; 64)	38% (33; 44)
Komplet respons %	1,4%	2,1%
Partielt respons %	57%	36%
p-værdi‡	< 0,0001	
**Responsvarighed**		
Median responsvarighed i måneder (interval)	7,7 (1,1+; 14,7+)	4,8 (1,3+; 15,8+)
% med varighed ≥ 6 måneder§	62%	40%

* Baseret på den stratificerede Cox *proportional hazard*-model
† Baseret på stratificeret log rank-test
‡ Baseret på Miettinen- og Nurminen-metoden
§ Baseret på Kaplan-Meier-estimering
NA = Foreligger ikke

Figur 9: Kaplan-Meier-kurve for samlet overlevelse i KEYNOTE-407

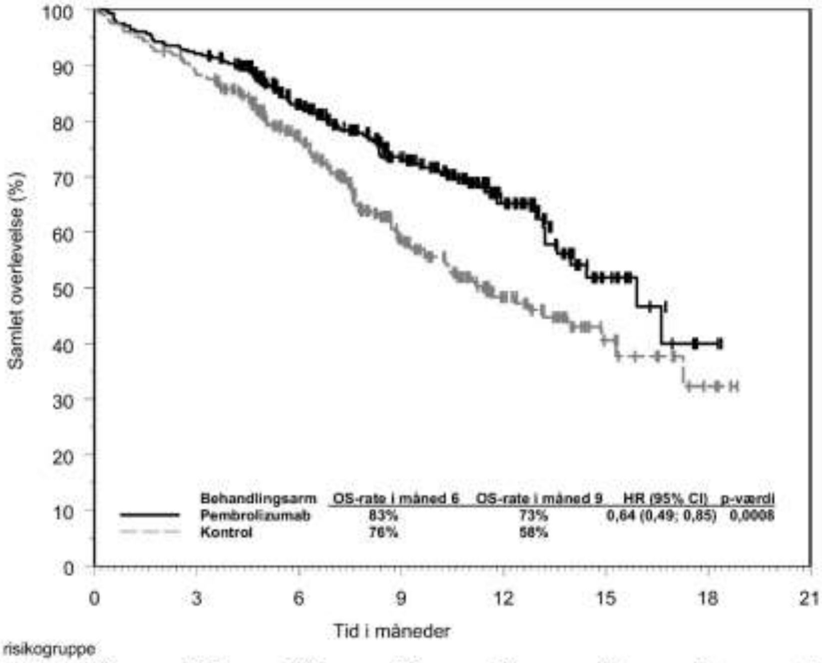

Antal i risikogruppe

Pembrolizumab:	278	256	188	124	62	17	2	0
Kontrol:	281	246	175	93	45	16	4	0

Figur 10: Kaplan-Meier-kurve for progressionsfri overlevelse i KEYNOTE-407

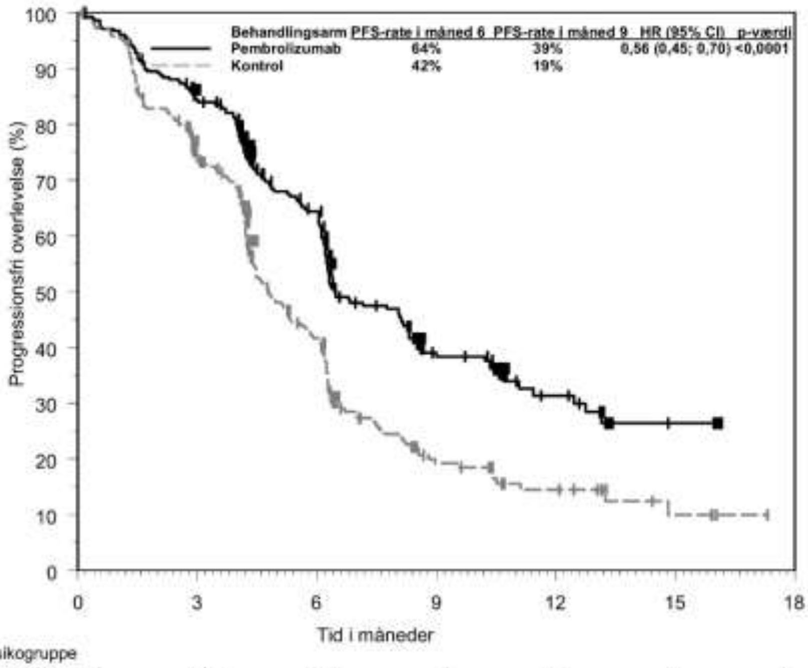

Antal i risikogruppe

Pembrolizumab:	278	223	142	57	23	5	0
Kontrol:	281	190	90	26	12	4	0

Der blev udført en analyse i KEYNOTE-407 hos patienter, som havde PD-L1 TPS < 1% [pembrolizumab plus kemoterapiarmen: n=95 (34%) *versus* placebo plus kemoterapiarmen: n=99 (35%)], TPS 1% til 49% [pembrolizumab plus kemoterapiarmen: n=103 (37%) *versus* placebo plus kemoterapiarmen: n=104 (37%)] eller TPS ≥ 50% [pembrolizumab plus kemoterapiarmen: n=73 (26%) *versus* placebo plus kemoterapiarmen: n=73 (26%)] (se tabel 16).

Tabel 16: Effektresultater ud fra PD-L1-ekspression i KEYNOTE-407

Endepunkt	Pembrolizumab kombinations-behandling	Kemoterapi	Pembrolizumab kombinations-behandling	Kemoterapi	Pembrolizumab kombinations-behandling	Kemoterapi
	TPS < 1%		TPS 1 til 49%		TPS ≥ 50%	
HR* for OS (95% CI)	0,61 (0,38; 0,98)		0,57 (0,36; 0,90)		0,64 (0,37; 1,10)	
HR* for PFS (95% CI)	0,68 (0,47; 0,98)		0,56 (0,39; 0,80)		0,37 (0,24; 0,58)	
ORR %	63%	40%	50%	41%	60%	33%

* *Hazard ratio* (pembrolizumab kombinationsbehandling sammenlignet med kemoterapi) baseret på den stratificerede Cox *proportional hazard*-model

I alt 65 NSCLC-patienter i alderen ≥ 75 år blev inkluderet i KEYNOTE-407-studiet (34 i kombinationsbehandlingen med pembrolizumab og 31 i kontrolgruppen). En HR=0,96 [95% CI 0,37; 2,52] for OS, en HR=0,60 [95% CI 0,29; 1,21] for PFS, og en ORR på 47% og 42% for kombinationsbehandlingen med pembrolizumab *versus* kemoterapi blev rapporteret i denne studiesubgruppe. Data vedrørende pembrolizumabs virkning og sikkerhed i kombination med platinbaseret kemoterapi er begrænset i denne patientpopulation.

104

KEYNOTE-010: Kontrolleret studie med NSCLC-patienter, der tidligere har været i behandling med kemoterapi

Pembrolizumabs sikkerhed og virkning blev undersøgt i KEYNOTE-010, et åbent, kontrolleret multicenterstudie til behandling af fremskredent NSCLC hos patienter, der tidligere har fået platinbaseret kemoterapi. Patienterne havde PD-L1-ekspression med TPS ≥ 1% baseret på PD-L1 IHC 22C3 pharmDx™-analysen. Patienter med EGFR-aktiverende mutationer eller ALK-translokation havde også sygdomsprogression, mens de var i godkendt behandling for disse mutationer inden behandling med pembrolizumab. Patienterne blev randomiseret i forholdet 1:1:1 til at få pembrolizumab i en dosis på 2 mg/kg (n=344) eller 10 mg/kg (n=346) hver 3. uge eller docetaxel i en dosis på 75 mg/m^2 hver 3. uge (n=343) indtil sygdomsprogression eller uacceptabel toksicitet. Patienter med autoimmun sygdom, en lidelse, som kræver behandling med immunsuppressiva, og patienter, som havde fået mere end 30 Gy torakal bestråling inden for de forudgående 26 uger, blev ekskluderet fra studiet. Tumorstatus blev vurderet hver 9. uge.

Baseline-karakteristika for denne population inkluderede: medianalder 63 år (42% i alderen 65 år eller ældre); 61% mænd; 72% kaukasere og 21% asiater og henholdsvis 34% og 66% med ECOG-performance-status 0 og 1. Sygdomskarakteristika var planocellulær (21%) og ikke-planocellulær (70%); M1-stadie (91%); stabile hjernemetastaser (15%), og forekomsten af mutationer var EGFR (8%) og ALK (1%). Forudgående behandling inkluderede platinbaseret dublet-regime (100%); patienterne havde fået en (69%) eller to eller flere (29%) behandlingslinjer.

De primære resultatmål var OS og PFS baseret på BICR-vurdering i henhold til RECIST 1.1. Sekundære resultatmål var ORR og responsvarighed. I tabel 17 opsummeres de centrale effektresultater for hele populationen (TPS ≥ 1%) og for patienter med TPS ≥ 50%, og figur 11 viser Kaplan-Meier-kurverne for OS (TPS ≥ 1%) baseret på en endelig analyse med en median opfølgningstid på 42,6 måneder.

Tabel 17: Respons på pembrolizumab 2 eller 10 mg/kg hver 3. uge hos tidligere behandlede patienter med NSCLC i KEYNOTE-010

Endepunkt	Pembrolizumab 2 mg/kg hver 3. uge	Pembrolizumab 10 mg/kg hver 3. uge	Docetaxel 75 mg/m² hver 3. uge
**TPS ≥ 1%**			
Antal patienter	344	346	343
**OS**			
Antal (%) patienter med hændelse	284 (83%)	264 (76%)	295 (86%)
HR* (95% CI)	0,77 (0,66; 0,91)	0,61 (0,52; 0,73)	---
p-værdi[†]	0,00128	< 0,001	---
Median i måneder (95% CI)	10,4 (9,5; 11,9)	13,2 (11,2; 16,7)	8,4 (7,6; 9,5)
**PFS**[‡]			
Antal (%) patienter med hændelse	305 (89%)	292 (84%)	314 (92%)
HR* (95% CI)	0,88 (0,75; 1,04)	0,75 (0,63; 0,89)	---
p-værdi[†]	0,065	< 0,001	---
Median i måneder (95% CI)	3,9 (3,1; 4,1)	4,0 (2,7; 4,5)	4,1 (3,8; 4,5)
**Objektiv responsrate**[‡]			
ORR % (95% CI)	20% (16; 25)	21% (17; 26)	9% (6; 13)
Komplet respons %	2%	3%	0%
Partielt respons %	18%	18%	9%
**Responsvarighed**[‡,§]			
Median i måneder (interval)	Ikke nået (2,8; 46,2+)	37,8 (2,0+; 49,3+)	7,1 (1,4+; 16,8)
% igangværende respons[¶]	42%	43%	6%
**TPS ≥ 50%**			
Antal patienter	139	151	152
**OS**			
Antal (%) patienter med hændelse	97 (70%)	102 (68%)	127 (84%)
HR* (95% CI)	0,56 (0,43; 0,74)	0,50 (0,38; 0,65)	---
p-værdi[†]	< 0,001	< 0,001	---
Median i måneder (95% CI)	15,8 (10,8; 22,5)	18,7 (12,1; 25,3)	8,2 (6,4; 9,8)
**PFS**[‡]			
Antal (%) patienter med hændelse	107 (77%)	115 (76%)	138 (91%)
HR* (95% CI)	0,59 (0,45; 0,77)	0,53 (0,41; 0,70)	---
p-værdi[†]	< 0,001	< 0,001	---
Median i måneder (95% CI)	5,3 (4,1; 7,9)	5,2 (4,1; 8,1)	4,2 (3,8; 4,7)
**Objektiv responsrate**[‡]			
ORR % (95% CI)	32% (24; 40)	32% (25; 41)	9% (5; 14)
Komplet respons %	4%	4%	0%
Partielt respons %	27%	28%	9%
**Responsvarighed**[‡,§]			
Median i måneder (interval)	Ikke nået (2,8; 44,0+)	37,5 (2,0+; 49,3+)	8,1 (2,6; 16,8)
% igangværende respons[¶]	55%	47%	8%

* *Hazard ratio* (pembrolizumab sammenlignet med docetaxel) baseret på den stratificerede Cox *proportional hazard*-model
† Baseret på stratificeret log rank-test
‡ BICR-vurdering i henhold til RECIST 1.1
§ Baseret på patienter med bedste objektive respons, der er bekræftet komplet respons eller partielt respons
¶ Igangværende respons inkluderer alle patienter med respons, som på tidspunktet for analysen, var i live, progressionsfri, ikke initierede ny behandling mod cancer og ikke blev anset for være *lost to follow-up*

106

Figur 11: Kaplan-Meier-kurve for samlet overlevelse i hver behandlingsarm i KEYNOTE-010 (patienter med PD-L1-ekspression TPS ≥ 1%, *intent to treat*-population)

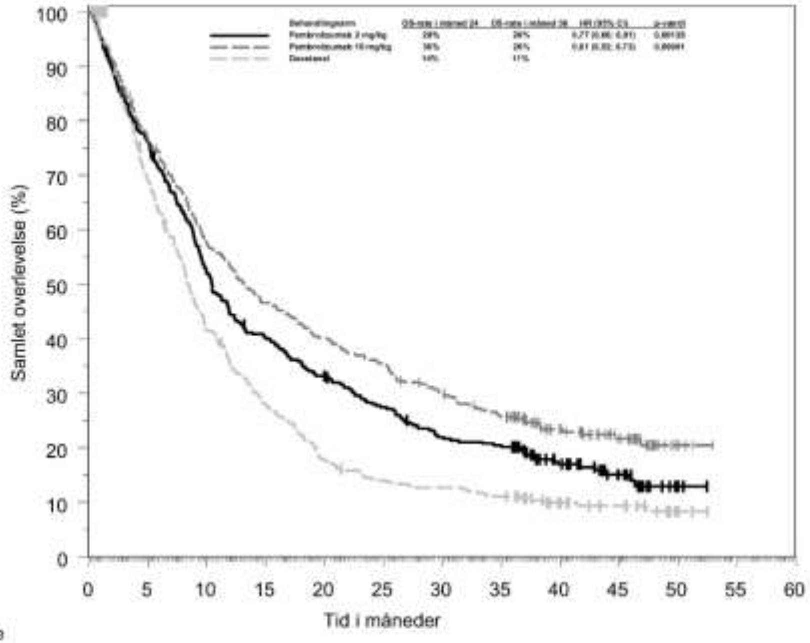

Antal i risikogruppe													
Pembrolizumab 2 mg/kg:	344	261	177	136	111	91	72	67	36	17	2	0	0
Pembrolizumab 10 mg/kg:	346	262	197	159	137	120	99	84	50	28	3	0	0
Docetaxel:	343	226	135	90	57	44	40	35	20	13	2	0	0

Effektresultaterne var sammenlignelige i armene med 2 mg/kg og 10 mg/kg pembrolizumab. Effektresultaterne for OS var konsistente uanset tumorprøvens alder (nyt væv *versus* arkivvæv) baseret på en sammenligning mellem grupperne.

I analyser af subgrupper viste pembrolizumab en reduceret fordel med hensyn til overlevelse sammenlignet med docetaxel hos patienter, som aldrig havde røget, og hos patienter med EGFR-aktiverende mutationer, der som minimum havde fået platinbaseret kemoterapi og en tyrosinkinasehæmmer. På grund af det lave antal patienter kan der dog ikke drages nogle definitive konklusioner på baggrund af disse data.

Pembrolizumabs virkning og sikkerhed hos patienter, hvis tumorer ikke udtrykker PD-L1, er ikke klarlagt.

Klassisk Hodgkins lymfom

KEYNOTE-087 og KEYNOTE-013: Åbne studier med patienter med recidiverende eller refraktært cHL

Pembrolizumabs virkning blev undersøgt i KEYNOTE-087 og KEYNOTE-013, to åbne multicenterstudier hos 241 patienter med cHL. Disse studier indrullerede patienter, som havde oplevet svigt af ASCT og svigt af behandling med BV; som var uegnede til ASCT, da de ikke var i stand til at opnå komplet eller partiel remission efter salvage-kemoterapi og havde oplevet svigt af behandling med BV; eller som havde oplevet svigt af ASCT og ikke havde fået BV. 5 forsøgspersoner var uegnede til ASCT af andre årsager end behandlingssvigt efter salvage-kemoterapi. Begge studier inkluderede patienter uanset PD-L1-ekspression. Patienter med aktiv, ikke-infektiøs pneumonitis, allogen transplantation inden for de seneste 5 år (eller > 5 år, men med GVHD), aktiv autoimmun sygdom eller en lidelse, som nødvendiggjorde behandling med immunsuppressiva, var uegnede til at deltage i begge

107

studier. Patienterne fik pembrolizumab 200 mg hver 3. uge (n=210; KEYNOTE-087) eller 10 mg/kg hver 2. uge (n=31; KEYNOTE-013) indtil uacceptabel toksicitet eller dokumenteret sygdomsprogression.

Hos patienterne i KEYNOTE-087 var *baseline*-karakteristika: medianalder 35 år (9% ≥ 65 år); 54% mænd; 88% kaukasere; og henholdsvis 49% og 51% havde ECOG-performance-status 0 og 1. Det mediane antal forudgående behandlingslinjer for cHL var 4 (1-12). 81% var refraktære over for mindst en forudgående behandling, inklusive 35%, som var refraktære over for førstelinjebehandling. 61% af patienterne havde gennemgået ASCT, 38% var uegnede til transplantation; 17% havde ikke tidligere fået brentuximab vedotin; og 36% af patienterne havde tidligere fået strålebehandling. Sygdomsundergrupperne var 80% nodulær sklerose, 11% blandet cellularitet, 4% lymfocytrig og 2% lymfocytfattig.

Hos patienterne i KEYNOTE-013 var *baseline*-karakteristika: medianalder 32 år (7% ≥ 65 år), 58% mænd, 94% kaukasere; og 45% og 55% havde henholdsvis ECOG-performance-status 0 og 1. Det mediane antal forudgående behandlingslinjer for cHL var 5 (2-15). 84% var refraktære over for mindst en forudgående behandling, inklusive 35%, som var refraktære over for førstelinjebehandling. 74% af patienterne havde gennemgået ASCT, 26% var uegnede til transplantation, og 45% af patienterne havde tidligere fået strålebehandling. Sygdomsundergrupperne var 97% nodulær sklerose og 3% blandet cellularitet.

De vigtigste resultatmål objektiv responsrate (ORR) og komplet remissionsrate (CRR) blev baseret på BICR-vurdering i henhold til IWG (*International Working Group*)-kriterierne revideret i 2007. Sekundære resultatmål var responsvarighed, PFS og OS. Respons blev vurderet i henholdsvis hver 12. (KEYNOTE-087) og hver 8. uge (KEYNOTE-013) med den første planlagte vurdering efter *baseline* i uge 12. Effektresultaterne opsummeres i tabel 18.

Tabel 18: Effektresultater i KEYNOTE-087 og KEYNOTE-013

Endepunkt	KEYNOTE-087[a] Pembrolizumab 200 mg hver 3. uge n=210	KEYNOTE-013[b] Pembrolizumab 10 mg/kg hver 2. uge n=31
**Objektiv responsrate**[c]		
ORR % (95% CI)	69% (62,3; 75,2)	58% (39,1; 75,5)
Komplet remission	22%	19%
Partiel remission	47%	39%
**Responsvarighed**[c]		
Median i måneder (interval)	11,1 (0,0+; 11,1)[d]	Ikke nået (0,0+; 45,6+)[e]
% med varighed ≥ 6 måneder	76%[f]	80%[g]
% med varighed ≥ 12 måneder	---	70%[h]
**Tid til respons**		
Median i måneder (interval)	2,8 (2,1; 8,8)[d]	2,8 (2,4; 8,6)[e]
**PFS**[c]		
Antal (%) patienter med hændelse	70 (33%)	19 (61%)
Median i måneder (95% CI)	11,3 (10,8; Ikke nået)	11,4 (4,9; 27,8)
6-måneders PFS-rate	72%	66%
9-måneders PFS-rate	62%	---
12-måneders PFS-rate	---	48%
**OS**		
Antal (%) patienter med hændelse	4 (2%)	6 (19%)
6-måneders OS-rate	99,5%	100%
12-måneders OS-rate	97,6%	87,1%

[a] Median opfølgningstid på 10,1 måneder
[b] Median opfølgningstid på 52,8 måneder
[c] BICR-vurdering i henhold til IWG (*International Working Group*)-kriterierne, revideret i 2007, ud fra PET/CT-scanninger
[d] Baseret på patienter (n=145) med et respons ud fra uafhængig vurdering
[e] Baseret på patienter (n=18) med et respons ud fra uafhængig vurdering
[f] Baseret på Kaplan-Meier-estimering; inkluderer 31 patienter med respons på 6 måneder eller længere
[g] Baseret på Kaplan-Meier-estimering; inkluderer 9 patienter med respons på 6 måneder eller længere
[h] Baseret på Kaplan-Meier-estimering; inkluderer 7 patienter med respons på 12 måneder eller længere

Sikkerhed og virkning hos ældre patienter

Alt i alt blev 20 cHL-patienter ≥ 65 år behandlet med pembrolizumab i studierne KEYNOTE-087 og KEYNOTE-013. Data fra disse patienter er for begrænsede til at kunne drage konklusioner om sikkerhed eller virkning for denne population.

Urotelialt karcinom

KEYNOTE-045: Kontrolleret studie med patienter med urotelialt karcinom, som tidligere har fået platin-baseret kemoterapi

Pembrolizumabs sikkerhed og virkning blev vurderet i KEYNOTE-045, et åbent, randomiseret (1:1), kontrolleret multicenterstudie til behandling af lokalt fremskredent eller metastatisk urotelialt karcinom hos patienter med sygdomsprogression ved eller efter platinbaseret kemoterapi. Patienterne skal have fået et *first line* platinbaseret regime for lokalt fremskredent/metastatisk sygdom eller som neoadjuvant/adjuvant behandling med tilbagevenden/progression ≤ 12 måneder efter afsluttet behandling. Patienterne blev randomiseret (1:1) til at få pembrolizumab 200 mg hver 3. uge (n=270) eller investigators valg af en hvilken som helst af følgende kemoterapi-regimer, der alle gives intravenøst hver 3. uge (n=272): paclitaxel 175 mg/m^2 (n=84), docetaxel 75 mg/m^2 (n=84) eller vinflunin 320 mg/m^2 (n=87). Patienterne blev behandlet med pembrolizumab indtil uacceptabel toksicitet eller sygdomsprogression. Behandlingen kunne fortsætte efter sygdomsprogression, hvis patienten var klinisk stabil, og investigator mente, at patienten havde en klinisk fordel. Patienter uden sygdomsprogression kunne behandles i op til 24 måneder. Patienter med autoimmun sygdom, en medicinsk tilstand som krævede behandling med immunsuppressiva, og patienter som tidligere havde fået mere end 2 omgange

systemisk kemoterapi mod metastatisk urotelialt karcinom blev ekskluderet fra studiet. Patienter med ECOG-performance-status på 2 skulle have et hæmoglobinniveau på ≥ 10 g/dl, måtte ikke have levermetastaser og måtte have fået den sidste dosis af det seneste tidligere kemoterapiregime ≥ 3 måneder før inklusion. Vurdering af tumorstatus blev foretaget 9 uger efter første dosis, derefter hver 6. uge i det første år, og efterfølgende hver 12. uge.

Blandt de 542 randomiserede patienter i KEYNOTE-045 var *baseline*-karakteristika følgende: median alder 66 år (interval: 26 til 88), 58% i alderen 65 år eller ældre; 74% mænd; 72% hvide og 23% asiater; 56% havde ECOG-performance-status på 1, og 1% havde ECOG-performance-status på 2; og 96% havde M1 sygdom, og 4% havde M0 sygdom. 87% af patienterne havde viscerale metastaser, herunder 34% med levermetastaser. 86% havde en primær tumor i de nedre urinveje, og 14% havde en primær tumor i de øvre urinveje. 15% af patienterne havde sygdomsprogression efter tidligere platinbaseret neoadjuvant eller adjuvant kemoterapi. 21% havde tidligere fået 2 systemiske regimer til behandling af metastaser. 76% af patienterne havde tidligere fået cisplatin, 23% havde tidligere fået carboplatin, og 1% var blevet behandlet med andre platinbaserede regimer.

De primære resultatmål var OS og PFS baseret på BICR i henhold til RECIST version 1.1. De sekundære resultatmål var ORR (baseret på BICR i henhold til RECIST version 1.1) og responsvarighed. I tabel 19 opsummeres de centrale effektresultater for ITT-populationen ved den endelige analyse. Kaplan-Meier-kurven baseret på den endelige analyse for OS er vist i figur 12. Studiet viste statistisk signifikante forbedringer i OS og ORR for patienter, der var randomiseret til pembrolizumab sammenlignet med kemoterapi. Der var ingen statistisk signifikant forskel mellem pembrolizumab og kemoterapi med hensyn til PFS.

Tabel 19: Respons på pembrolizumab 200 mg hver 3. uge hos patienter med urotelialt karcinom, der tidligere var blevet behandlet med kemoterapi i KEYNOTE-045

Endepunkt	Pembrolizumab 200 mg hver 3. uge n=270	Kemoterapi n=272
**OS**		
Antal (%) patienter med hændelse	200 (74%)	219 (81%)
HR* (95% CI)	0,70 (0,57; 0,85)	
p-værdi†	< 0,001	
Median i måneder (95% CI)	10,1 (8,0; 12,3)	7,3 (6,1; 8,1)
**PFS‡**		
Antal (%) patienter med hændelse	233 (86%)	237 (87%)
HR* (95% CI)	0,96 (0,79; 1,16)	
p-værdi†	0,313	
Median i måneder (95% CI)	2,1 (2,0; 2,2)	3,3 (2,4; 3,6)
**Objektiv responsrate‡**		
ORR % (95% CI)	21% (16; 27)	11% (8; 15)
p-værdi§	< 0,001	
Komplet respons	9%	3%
Partielt respons	12%	8%
Stabil sygdom	17%	34%
**Responsvarighed‡,¶**		
Median i måneder (interval)	Ikke nået (1,6+; 30,0+)	4,4 (1,4+; 29,9+)
Antal (%#) patienter med varighed ≥ 6 måneder	46 (84%)	8 (47%)
Antal (%#) patienter med varighed ≥ 12 måneder	35 (68%)	5 (35%)

* *Hazard ratio* (pembrolizumab sammenlignet med kemoterapi) baseret på den stratificerede Cox *proportional hazard-model*
† Baseret på stratificeret log rank-test
‡ BICR-vurdering i henhold til RECIST 1.1
§ Baseret på Miettinen- og Nurminen-metoden
¶ Baseret på patienter med bedste objektive respons, der er bekræftet komplet eller partielt respons
# Baseret på Kaplan-Meier-estimering

111

Figur 12: Kaplan-Meier-kurve for samlet overlevelse i hver behandlingsarm i KEYNOTE-045 (*intent to treat*-population)

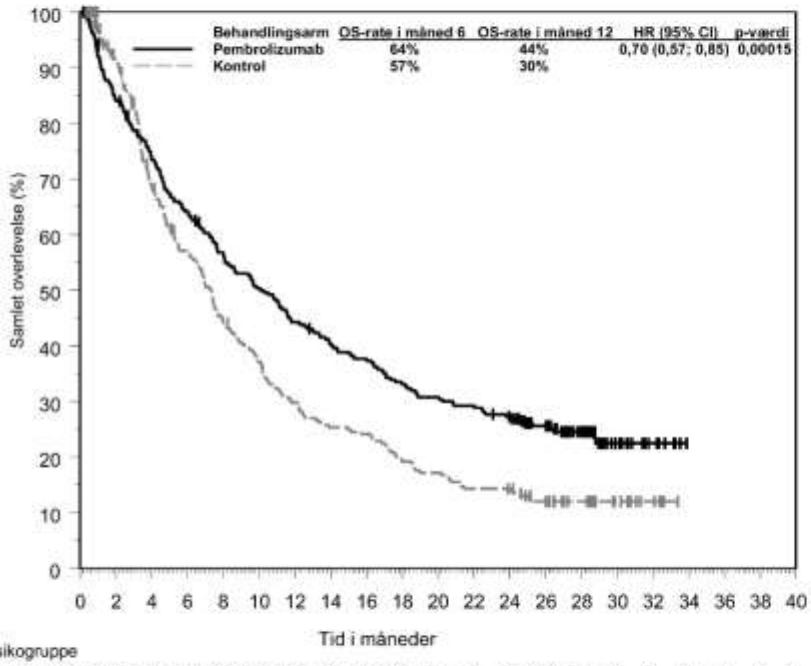

Antal i risikogruppe

Pembrolizumab:	270	226	195	170	148	132	116	105	98	86	80	76	67	52	33	14	7	0	0	0	0
Kontrol:	272	234	173	140	109	91	73	62	59	47	42	35	34	24	18	10	4	0	0	0	0

Der blev udført en analyse i KEYNOTE-045 hos patienter, der havde PD-L1 CPS < 10 [pembrolizumab: n=186 (69%) *versus* kemoterapi: n=176 (65%)] eller ≥ 10 [pembrolizumab: n=74 (27%) *versus* kemoterapi: n=90 (33%)] i både pembrolizumab- og kemoterapi-armene (se tabel 20).

Tabel 20: OS ved PD-L1-ekspression

PD-L1-ekspression	Pembrolizumab	Kemoterapi	
	OS ved PD-L1-ekspression Antal (%) patienter med hændelse*		HR[†] (95% CI)
CPS < 10	140 (75%)	144 (82%)	0,75 (0,59; 0,95)
CPS ≥ 10	53 (72%)	72 (80%)	0,55 (0,37; 0,81)

* Baseret på den endelige analyse
† *Hazard ratio* (pembrolizumab sammenlignet med kemoterapi) baseret på stratificeret Cox *proportional hazard*-model

Patient-rapporterede oplysninger (PRO) blev vurderet i henhold til EORTC QLQ-C30. I EORTC QLQ-C30 global helbredstilstand/QoL sås en forlængelse af tiden til forværring hos patienter, der var blevet behandlet med pembrolizumab sammenlignet med investigators valg af kemoterapi (HR 0,70; 95% CI 0,55-0,90). I løbet af 15 ugers opfølgning opnåede patienter, der var blevet behandlet med pembrolizumab, en stabil global helbredstilstand/QoL, hvorimod de der var blevet behandlet med investigators valg af kemoterapi fik en nedgang i global helbredstilstand/QoL. Disse resultater skal fortolkes i konteksten af det åbne studiedesign og skal derfor fortolkes med forsigtighed.

KEYNOTE-052: Åbent studie med patienter med urotelialt karcinom, der er uegnede til cisplatinbaseret kemoterapi

Pembrolizumabs sikkerhed og virkning blev undersøgt i KEYNOTE-052, et åbent, multicenterstudie til behandling af lokalt fremskredent eller metastatisk urotelialt karcinom hos patienter, som var uegnede til behandling med cisplatinbaseret kemoterapi. Patienterne fik pembrolizumab i doser på 200 mg hver 3. uge, indtil uacceptabel toksicitet eller sygdomsprogression. Behandlingen kunne fortsætte efter progression, hvis patienten var klinisk stabil, og investigator mente, at patienten havde en klinisk fordel. Patienter uden sygdomsprogression kunne behandles i op til 24 måneder. Patienter med autoimmun sygdom eller en medicinsk tilstand, som krævede behandling med immunsuppressiva blev ekskluderet fra studiet. Vurdering af tumorstatus blev foretaget 9 uger efter første dosis, derefter hver 6. uge i det første år, og efterfølgende hver 12. uge.

Blandt de 370 patienter med urotelialt karcinom, som var uegnede til cisplatinbaseret kemoterapi var *baseline*-karakteristika følgende: median alder 74 år (82% i alderen 65 år eller ældre); 77% mænd; og 89% hvide og 7% asiater. 88% havde M1-sygdom, og 12% havde M0-sygdom. 85% af patienterne havde viscerale metastaser, herunder 21% med levermetastaser. Årsager til cisplatin-uegnethed var: creatinin-clearance på < 60 ml/min (50%) ved *baseline*, ECOG-performance-status på 2 (32%), ECOG-performance-status på 2 og creatinin-clearance på < 60 ml/min (9%) ved *baseline* og andre (hjertesvigt klasse III, perifer neuropati grad 2 eller højere og høretab grad 2 eller højere; 9%). 90% af patienterne var behandlingsnaive, og 10% havde tidligere fået adjuvant eller neoadjuvant platinbaseret kemoterapi. 81% havde en primær tumor i den nederste del af urinvejene, og 19% havde en primær tumor i de øvre urinveje.

Det primære resultatmål var ORR baseret på BICR i henhold til RECIST version 1.1. De sekundære resultatmål var responsvarighed, PFS og OS. I tabel 21 opsummeres de centrale effektresultater for studiepopulationen ved den endelige analyse på basis af en median opfølgningstid på 11,4 måneder (interval: 0,1; 41,2 måneder) for alle patienter.

Tabel 21: Respons på pembrolizumab 200 mg hver 3. uge hos patienter med urotelialt karcinom, der var uegnede til behandling med cisplatinbaseret kemoterapi i KEYNOTE-052

**Endepunkt**	**n=370**
**Objektiv responsrate***	
ORR %, (95% CI)	29% (24; 34)
Sygdomskontrolrate†	47%
Komplet respons	9%
Partielt respons	20%
Stabil sygdom	18%
**Responsvarighed**	
Median i måneder (interval)	30,1 (1,4+; 35,9+)
% med varighed ≥ 6 måneder	81%‡
**Tid til respons**	
Median i måneder (interval)	2,1 (1,3; 9,0)
**PFS***	
Median i måneder (95% CI)	2,2 (2,1; 3,4)
6-måneders PFS-rate	33%
12-måneders PFS-rate	22%
**OS**	
Median i måneder (95% CI)	11,3 (9,7; 13,1)
6-måneders OS-rate	67%
12-måneders OS-rate	47%

* BICR-vurdering i henhold til RECIST 1.1
† Baseret på bedst respons for stabil sygdom eller bedre
‡ Baseret på Kaplan-Meier-estimering; omfatter 84 patienter med 6 måneders respons eller længere

Der blev udført en analyse i KEYNOTE-052 hos patienter, som havde tumorer, der udtrykte PD-L1 med CPS < 10 (n=251; 68%) eller ≥ 10 (n=110; 30%) baseret på PD-L1 IHC 22C3 pharmDx™-analysen (se tabel 22).

Tabel 22: ORR og OS ved PD-L1-ekspression

**Endepunkt**	**CPS < 10 N=251**	**CPS ≥ 10 N=110**
**Objektiv responsrate***		
ORR %, (95% CI)	20% (16; 26)	47% (38; 57)
**OS**		
Median i måneder (95% CI)	10 (8; 12)	19 (12; 29)
12-måneders OS-rate	41%	61%

* BICR i henhold til RECIST 1.1

KEYNOTE-361 er et igangværende randomiseret, kontrolleret, åbent klinisk fase III-studie af pembrolizumab med eller uden platinbaseret kombineret kemoterapi *versus* kemoterapi som førstelinjebehandling hos forsøgspersoner med fremskredent eller metastatisk urotelialt karcinom. Foreløbige data fra en tidlig gennemgang viste en nedsat overlevelse med pembrolizumab som monoterapi hos patienter, hvis tumorer udtrykker PD-L1 med CPS < 10 sammenlignet med standardkemoterapi.

På basis af en anbefaling fra en ekstern datamonitoreringskomité blev tilgangen af patienter i armen med pembrolizumab som monoterapi stoppet for patienter, hvis tumorer udtrykker PD-L1 med CPS < 10.

114

Armen med pembrolizumab som monoterapi forbliver kun åben for patienter, hvis tumorer udtrykker PD-L1 med CPS ≥ 10. Forsøgspersoner, hvis tumorer udtrykker PD-L1 CPS < 10 og som allerede er indrulleret i armen med pembrolizumab som monoterapi, kan fortsætte behandlingen. Randomisering til kemoterapi- og kemoterapi-pembrolizumab-armene forbliver åben.

Planocellulært hoved-hals karcinom
KEYNOTE-048: Kontrolleret studie af monoterapi og kombinationsbehandling hos behandlingsnaive HNSCC-patienter i behandling for recidiverende eller metastatisk sygdom
Pembrolizumabs virkning blev undersøgt i KEYNOTE-048, et randomiseret, åbent, aktivt kontrolleret multicenterstudie hos patienter med histologisk bekræftet metastatisk eller recidiverende HNSCC i mundhulen, svælget (farynx) eller struben (larynx), som ikke tidligere havde fået systemisk behandling for recidiverende eller metastatisk sygdom, og som blev anset for at være uhelbredeligt med lokal behandling. Patienter med nasofaryngealt karcinom, aktiv autoimmun sygdom, der havde krævet systemisk behandling inden for 2 år efter behandling, eller lidelser, som krævede behandling med immunsuppressiva, var uegnede til studiet. Randomiseringen blev stratificeret ud fra PD-L1-tumorekspression (TPS ≥ 50% eller < 50%), HPV-status (positiv eller negativ) og ECOG-PS (0 *versus* 1). Patienterne blev randomiseret i forholdet 1:1:1 til en af følgende behandlingsarme:

- Pembrolizumab 200 mg hver 3. uge
- Pembrolizumab 200 mg hver 3. uge, carboplatin AUC 5 mg/ml/min hver 3. uge eller cisplatin 100 mg/m^2 hver 3. uge, og 5-FU 1.000 mg/m^2/dag kontinuerligt i 4 dage hver 3. uge (maksimalt 6 serier med platinbaseret behandling og 5-FU)
- Cetuximab 400 mg/m^2 støddosis og herefter 250 mg/m^2 en gang ugentligt, carboplatin AUC 5 mg/ml/min hver 3. uge eller cisplatin 100 mg/m^2 hver 3. uge, og 5-FU 1.000 mg/m^2/dag kontinuerligt i 4 dage hver 3. uge (maksimalt 6 serier med platinbaseret behandling og 5-FU)

Behandling med pembrolizumab fortsatte indtil sygdomsprogression defineret i henhold til RECIST 1.1 efter investigators vurdering, uacceptabel toksicitet eller i højst 24 måneder. Administration af pembrolizumab var tilladt efter sygdomsprogression defineret i henhold til RECIST, hvis patienten var klinisk stabil, og investigator vurderede, at patienten havde en klinisk fordel. Vurdering af tumorstatus blev foretaget i uge 9 og herefter hver 6. uge i løbet af det første år, og efterfølgende hver 9. uge til og med måned 24.

Blandt de 882 patienter i KEYNOTE-048, havde 754 (85%) tumorer, som udtrykte PD-L1 med CPS ≥ 1 baseret på PD-L1 IHC 22C3 pharmDx™-analysen. Karakteristika ved *baseline* for disse 754 patienter inkluderede: medianalder på 61 år (interval: 20 til 94); 36% i alderen 65 eller ældre; 82% mænd; 74% kaukasere og 19% asiatere; 61% med ECOG-performance-status 1; og 77% tidligere/nuværende rygere. Sygdomskarakteristika var: 21% HPV-positive og 95% havde sygdom i stadie IV (stadie IVa 21%, stadie IVb 6% og stadie IVc 69%).

De primære resultatmål var OS og PFS (BICR-vurdering i henhold til RECIST 1.1). Studiet viste en statistisk signifikant forbedring i OS for alle patienter, som var randomiseret til pembrolizumab i kombination med kemoterapi sammenlignet med standardbehandling (HR: 0,72; 95% CI 0,60-0,87), og hos patienter, hvis tumorer udtrykte PD-L1 CPS ≥ 1, som var randomiseret til pembrolizumab som monoterapi sammenlignet med standardbehandling. I tabel 23 og 24 opsummeres de centrale effektresultater for pembrolizumab hos patienter, hvis tumorer udtrykte PD-L1 med CPS ≥ 1 i KEYNOTE-048 ved den endelige analyse udført med en median opfølgningstid på 13 måneder for pembrolizumab i kombination med kemoterapi og med en median opfølgningstid på 11,5 måneder for pembrolizumab som monoterapi. Kaplan-Meier-kurver for OS baseret på den endelige analyse er vist i figur 13 og 14.

Tabel 23: Effektresultater for pembrolizumab plus kemoterapi i KEYNOTE-048 med PD-L1-ekspression (CPS ≥ 1)

Endepunkt	Pembrolizumab + platinbaseret kemoterapi + 5-FU n=242	Standardbehandling* n=235
**OS**		
Antal (%) patienter med hændelse	177 (73%)	213 (91%)
Median i måneder (95% CI)	13,6 (10,7; 15,5)	10,4 (9,1; 11,7)
HR[†] (95% CI)	0,65 (0,53; 0,80)	
p-værdi[‡]	0,00002	
**PFS**		
Antal (%) patienter med hændelse	212 (88%)	221 (94%)
Median i måneder (95% CI)	5,1 (4,7; 6,2)	5,0 (4,8; 6,0)
HR[†] (95% CI)	0,84 (0,69; 1,02)	
p-værdi[‡]	0,03697	
**Objektiv responsrate**		
ORR[§] (95% CI)	36% (30,3; 42,8)	36% (29,6; 42,2)
Komplet respons	7%	3%
Partielt respons	30%	33%
p-værdi[¶]	0,4586	
**Responsvarighed**		
Median i måneder (interval)	6,7 (1,6+; 39,0+)	4,3 (1,2+; 31,5+)
% med varighed ≥ 6 måneder	54%	34%

* Cetuximab, platinbaseret behandling og 5-FU
† Baseret på den stratificerede Cox *proportional hazard*-model
‡ Baseret på stratificeret log rank-test
§ Respons: Bedste objektive respons, der er bekræftet komplet eller partielt respons
¶ Baseret på Miettinen- og Nurminen-metoden stratificeret ud fra ECOG (0 *versus* 1), HPV-status (positiv *versus* negativ) og PD-L1-status (markant positiv *versus* ikke markant positiv)

Figur 13: Kaplan-Meier-kurve for samlet overlevelse for pembrolizumab plus kemoterapi i KEYNOTE-048 med PD-L1-ekspression (CPS ≥ 1)

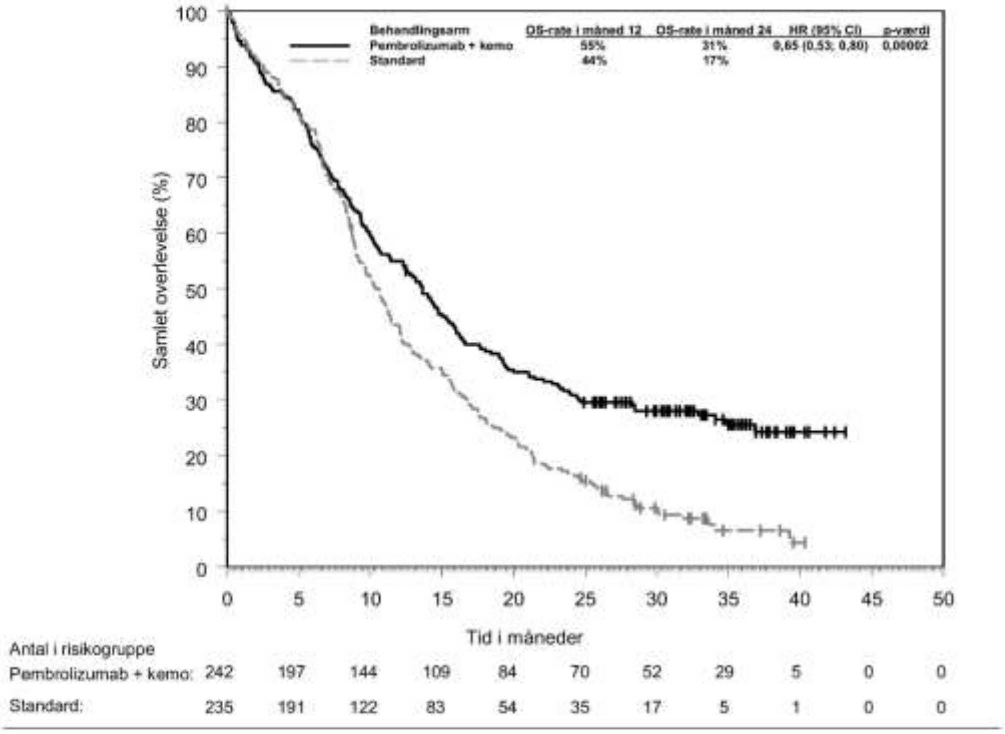

117

Tabel 24: Effektresultater for pembrolizumab som monoterapi i KEYNOTE-048 med PD-L1-ekspression (CPS ≥ 1)

Endepunkt	Pembrolizumab n=257	Standardbehandling* n=255
**OS**		
Antal (%) patienter med hændelse	197 (77%)	229 (90%)
Median i måneder (95% CI)	12,3 (10,8; 14,3)	10,3 (9,0; 11,5)
HR[†] (95% CI)	0,74 (0,61; 0,90)	
p-værdi[‡]	0,00133	
**PFS**		
Antal (%) patienter med hændelse	228 (89%)	237 (93%)
Median i måneder (95% CI)	3,2 (2,2; 3,4)	5,0 (4,8; 6,0)
HR[†] (95% CI)	1,13 (0,94; 1,36)	
p-værdi[‡]	0,89580	
**Objektiv responsrate**		
ORR[§] (95% CI)	19,1% (14,5; 24,4)	35% (29,1; 41,1)
Komplet respons	5%	3%
Partielt respons	14%	32%
p-værdi[¶]	1,0000	
**Responsvarighed**		
Median i måneder (interval)	23,4 (1,5+; 43,0+)	4,5 (1,2+; 38,7+)
% med varighed ≥ 6 måneder	81%	36%

* Cetuximab, platinbaseret behandling og 5-FU
† Baseret på den stratificerede Cox *proportional hazard*-model
‡ Baseret på stratificeret log rank-test
§ Respons: Bedste objektive respons, der er bekræftet komplet eller partielt respons
¶ Baseret på Miettinen- og Nurminen-metoden stratificeret ud fra ECOG (0 *versus* 1), HPV-status (positiv *versus* negativ) og PD-L1-status (markant positiv *versus* ikke markant positiv)

Figur 14: Kaplan-Meier-kurve for samlet overlevelse for pembrolizumab som monoterapi i KEYNOTE-048 med PD-L1-ekspression (CPS ≥ 1)

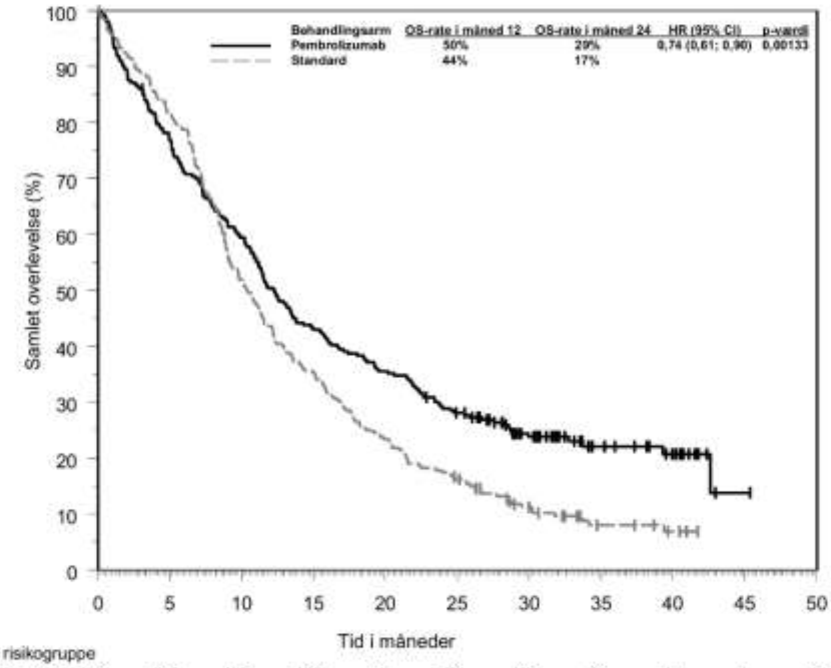

Antal i risikogruppe										
Pembrolizumab: 257	197	152	110	91	70	43	21	13	1	0
Standard: 255	207	131	89	59	40	21	9	5	0	0

Der blev udført en analyse i KEYNOTE-048 hos patienter, hvis tumorer udtrykte PD-L1 CPS ≥ 20 [pembrolizumab plus kemoterapi: n=126 (49%) *versus* standardbehandling: n=110 (43%) og pembrolizumab som monoterapi: n=133 (52%) *versus* standardbehandling: n=122 (48%)] (se tabel 25).

119

Tabel 25: Effektresultater for pembrolizumab plus kemoterapi og pembrolizumab som monoterapi ud fra PD-L1-ekspression i KEYNOTE-048 (CPS ≥ 20)

Endepunkt	Pembrolizumab + platinbaseret kemoterapi + 5-FU n=126	Standard-behandling* n=110	Pembrolizumab-monoterapi n=133	Standard-behandling* n=122
**OS**				
Antal (%) patienter med hændelse	84 (66,7)	98 (89,1)	94 (70,7)	108 (88,5)
Median i måneder (95% CI)	14,7 (10,3; 19,3)	11,0 (9,2; 13,0)	14,8 (11,5; 20,6)	10,7 (8,8; 12,8)
HR† (95% CI)	0,60 (0,45; 0,82)		0,58 (0,44; 0,78)	
p-værdi‡	0,00044		0,00010	
OS-rate i måned 6 (95% CI)	74,6 (66,0; 81,3)	80,0 (71,2; 86,3)	74,4 (66,1; 81,0)	79,5 (71,2; 85,7)
OS-rate i måned 12 (95% CI)	57,1 (48,0; 65,2)	46,1 (36,6; 55,1)	56,4 (47,5; 64,3)	44,9 (35,9; 53,4)
OS-rate i måned 24 (95% CI)	35,4 (27,2; 43,8)	19,4 (12,6; 27,3)	35,3 (27,3; 43,4)	19,1 (12,7; 26,6)
**PFS**				
Antal (%) patienter med hændelse	106 (84,1)	104 (94,5)	115 (86,5)	114 (93,4)
Median i måneder (95% CI)	5,8 (4,7; 7,6)	5,3 (4,9; 6,3)	3,4 (3,2; 3,8)	5,3 (4,8; 6,3)
HR† (95% CI)	0,76 (0,58; 1,01)		0,99 (0,76; 1,29)	
p-værdi‡	0,02951		0,46791	
PFS-rate i måned 6 (95% CI)	49,4 (40,3; 57,9)	47,2 (37,5; 56,2)	33,0 (25,2; 41,0)	46,6 (37,5; 55,2)
PFS-rate i måned 12 (95% CI)	23,9 (16,7; 31,7)	14,0 (8,2; 21,3)	23,5 (16,6; 31,1)	15,1 (9,3; 22,2)
PFS-rate i måned 24 (95% CI)	14,6 (8,9; 21,5)	5,0 (1,9; 10,5)	16,8 (10,9; 23,8)	6,1 (2,7; 11,6)
**Objektiv responsrate**				
ORR§ (95% CI)	42,9 (34,1; 52,0)	38,2 (29,1; 47,9)	23,3 (16,4; 31,4)	36,1 (27,6; 45,3)
**Responsvarighed**				
Antal patienter med respons	54	42	31	44
Median i måneder (interval)	7,1 (2,1+; 39,0+)	4,2 (1,2+; 31,5+)	22,6 (2,7+; 43,0+)	4,2 (1,2+; 31,5+)

* Cetuximab, platinbaseret behandling og 5-FU
† Baseret på den stratificerede Cox *proportional hazard*-model
‡ Baseret på stratificeret log rank-test
§ Respons: Bedste objektive respons, der er bekræftet komplet eller partielt respons

Der blev udført en eksploratorisk subgruppeanalyse i KEYNOTE-048 hos patienter, hvis tumorer udtrykte PD-L1 CPS ≥ 1 til < 20 [pembrolizumab plus kemoterapi: n=116 (45%) *versus* standardbehandling: n=125 (49%) og pembrolizumab som monoterapi: n=124 (48%) *versus* standardbehandling: n=133 (52%)] (se tabel 26).

120

Tabel 26: Effektresultater for pembrolizumab plus kemoterapi og pembrolizumab som monoterapi ud fra PD-L1-ekspression i KEYNOTE-048 (CPS ≥ 1 til < 20)

Endepunkt	Pembrolizumab + platinbaseret kemoterapi + 5-FU n=116	Standard-behandling* n=125	Pembrolizumab-monoterapi n=124	Standard-behandling* n=133
**OS**				
Antal (%) patienter med hændelse	93 (80,2)	115 (92,0)	103 (83,1)	121 (91,0)
Median i måneder (95% CI)	12,7 (9,4; 15,3)	9,9 (8,6; 11,5)	10,8 (9,0; 12,6)	10,1 (8,7; 12,1)
HR† (95% CI)	0,71 (0,54; 0,94)		0,86 (0,66; 1,12)	
OS-rate i måned 6 (95% CI)	76,7 (67,9; 83,4)	77,4 (69,0; 83,8)	67,6 (58,6; 75,1)	78,0 (70,0; 84,2)
OS-rate i måned 12 (95% CI)	52,6 (43,1; 61,2)	41,1 (32,4; 49,6)	44,0 (35,1; 52,5)	42,4 (33,9; 50,7)
OS-rate i måned 24 (95% CI)	25,9 (18,3; 34,1)	14,5 (9,0; 21,3)	22,0 (15,1; 29,6)	15,9 (10,3; 22,6)
**PFS**				
Antal (%) patienter med hændelse	106 (91,4)	117 (93,6)	113 (91,1)	123 (92,5)
Median i måneder (95% CI)	4,9 (4,2; 5,3)	4,9 (3,7; 6,0)	2,2 (2,1; 2,9)	4,9 (3,8; 6,0)
HR† (95% CI)	0,93 (0,71; 1,21)		1,25 (0,96; 1,61)	
PFS-rate i måned 6 (95% CI)	40,1 (31,0; 49,0)	40,0 (31,2; 48,5)	24,2 (17,1; 32,0)	41,4 (32,8; 49,7)
PFS-rate i måned 12 (95% CI)	15,1 (9,1; 22,4)	11,3 (6,4; 17,7)	17,5 (11,4; 24,7)	12,1 (7,2; 18,5)
PFS-rate i måned 24 (95% CI)	8,5 (4,2; 14,7)	5,0 (1,9; 10,1)	8,3 (4,3; 14,1)	6,3 (2,9; 11,5)
**Objektiv responsrate**				
ORR‡ (95% CI)	29,3 (21,2; 38,5)	33,6 (25,4; 42,6)	14,5 (8,8; 22,0)	33,8 (25,9; 42,5)
**Responsvarighed**				
Antal patienter med respons	34	42	18	45
Median i måneder (interval)	5,6 (1,6+; 25,6+)	4,6 (1,4+; 31,4+)	NR (1,5+; 38,9+)	5,0 (1,4+; 38,7+)

* Cetuximab, platinbaseret behandling og 5-FU
† Baseret på den stratificerede Cox *proportional hazard*-model
‡ Respons: Bedste objektive respons, der er bekræftet komplet eller partielt respons

KEYNOTE-040: Kontrolleret studie med HNSCC-patienter, som tidligere har været behandlet med platinbaseret kemoterapi

Pembrolizumabs sikkerhed og virkning blev undersøgt i KEYNOTE-040, et åbent, randomiseret, kontrolleret multicenterstudie til behandling af histologisk bekræftet recidiverende eller metastatisk HNSCC i mundhulen, svælget (farynx) eller struben (larynx) hos patienter, som havde sygdomsprogression under eller efter platinbaseret kemoterapi administreret til behandling af recidiverende eller metastatisk HNSCC eller efter platinbaseret kemoterapi administreret som en del af induktionsbehandling, samtidig behandling eller adjuverende behandling, og som ikke var modtagelige for lokal kurativt intenderet behandling. Patienterne blev stratificeret ud fra PD-L1-tumorekspression (TPS ≥ 50%), HPV-status og ECOG-performance-status og blev herefter randomiseret i forholdet 1:1 til at få enten pembrolizumab 200 mg hver 3. uge (n=247) eller en af tre standardbehandlinger (n=248): methotrexat 40 mg/m^2 en gang ugentligt (n=64), docetaxel 75 mg/m^2 en gang hver 3. uge (n=99) eller cetuximab 400 mg/m^2 støddosis og herefter 250 mg/m^2 en gang ugentligt (n=71). Behandlingen kunne fortsætte efter sygdomsprogression, hvis patienten var klinisk stabil, og investigator vurderede, at patienten havde en klinisk fordel. Patienter med nasofaryngealt karcinom, aktiv autoimmun sygdom, der

havde krævet systemisk behandling inden for de seneste 2 år efter behandling, lidelser, som krævede behandling med immunsuppressiva, eller som tidligere havde fået 3 eller flere systemiske regimer til behandling af recidiverende og/eller metastatisk HNSCC, blev ekskluderet fra studiet. Vurdering af tumorstatus blev foretaget i uge 9, herefter hver 6. uge til og med uge 52, og efterfølgende hver 9. uge til og med måned 24.

Blandt de 495 patienter i KEYNOTE-040 havde 129 (26%) tumorer, der udtrykte PD-L1 med TPS ≥ 50% baseret på PD-L1 IHC 22C3 pharmDx™-analysen. *Baseline*-karakteristika for disse 129 patienter inkluderede: medianalder 62 år (40% i alderen 65 år eller ældre); 81% mænd; 78% kaukasere, 11% asiater og 2% negroide; henholdsvis 23% og 77% havde ECOG-performance-status på 0 eller 1; og 19% havde HPV-positive tumorer. 67% af patienterne havde M1-sygdom og størstedelen havde sygdom i stadie IV (stadie IV 32%, stadie IVa 14%, stadie IVb 4% og stadie IVc 44%). 16% havde sygdomsprogression efter platinbaseret neoadjuverende eller adjuverende kemoterapi, og 84% havde tidligere fået 1-2 systemiske regimer for metastatisk sygdom.

Det primære resultatmål var OS i ITT-populationen. Den initiale analyse resulterede i en HR for OS på 0,82 (95% CI: 0,67; 1,01) med ensidet p-værdi på 0,0316. Den mediane OS var 8,4 måneder for pembrolizumab sammenlignet med 7,1 måneder for standardbehandling. I tabel 27 opsummeres de centrale effektresultater for populationen med TPS ≥ 50%. Kaplan-Meier-kurver for OS for populationen med TPS ≥ 50% er vist i figur 15.

Tabel 27: Effekt af pembrolizumab 200 mg hver 3. uge hos HNSCC-patienter med TPS ≥ 50%, som tidligere har været behandlet med platinbaseret kemoterapi i KEYNOTE-040

**Endepunkt**	**Pembrolizumab 200 mg hver 3. uge n=64**	**Standardbehandling* n=65**
**OS**		
Antal (%) patienter med hændelse	41 (64)	56 (86)
HR† (95% CI)	0,53 (0,35; 0,81)	
p-værdi‡	0,001	
Median i måneder (95% CI)	11,6 (8,3; 19,5)	6,6 (4,8; 9,2)
**PFS§**		
Antal (%) patienter med hændelse	52 (81)	58 (89)
HR† (95% CI)	0,58 (0,39; 0,86)	
p-værdi‡	0,003	
Median i måneder (95% CI)	3,5 (2,1; 6,3)	2,1 (2,0; 2,4)
6-måneders rate (%) (95% CI)	40,1 (28,1; 51,9)	17,1 (8,8; 27,7)
**Objektiv responsrate§**		
ORR% (95% CI)	26,6 (16,3; 39,1)	9,2 (3,5; 19,0)
p-værdi¶	0,0009	
Komplet respons	5%	2%
Partielt respons	22%	8%
Stabil sygdom	23%	23%
**Responsvarighed§,#**		
Median i måneder (interval)	Ikke nået (2,7; 13,8+)	6,9 (4,2; 18,8)
Antal (%[b]) patienter med varighed ≥ 6 måneder	9 (66)	2 (50)

* Methotrexat, docetaxel eller cetuximab
† *Hazard ratio* (pembrolizumab sammenlignet med standardbehandling) baseret på stratificeret Cox *proportional hazard*-model
‡ Ensidet p-værdi baseret på log-rank test
§ BICR-vurdering i henhold til RECIST 1.1
¶ Baseret på Miettinen- og Nurminen-metoden
\# Baseret på patienter med bedste objektive respons, der er bekræftet komplet eller partielt respons
b Baseret på Kaplan-Meier-estimering

122

Figur 15: Kaplan-Meier-kurve for samlet overlevelse for hver behandlingsarm hos patienter med PD-L1-ekspression (TPS ≥ 50%) i KEYNOTE-040

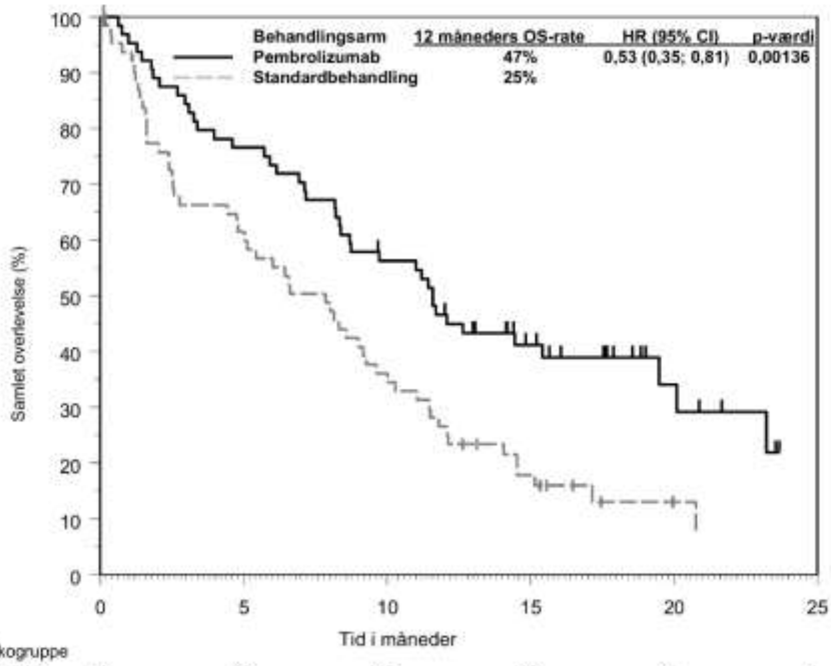

Antal i risikogruppe						
Pembrolizumab:	64	49	35	19	7	1
Standardbehandling:	65	38	22	9	2	0

Renalcellekarcinom

KEYNOTE-426: Kontrolleret studie af kombinationsbehandling hos behandlingsnaive RCC-patienter

Pembrolizumabs virkning i kombination med axitinib blev undersøgt i KEYNOTE-426, et randomiseret, åbent, aktivt kontrolleret multicenterstudie hos patienter med fremskredent RCC med en *clear cell*-komponent, uanset PD-L1-tumorekspressionsstatus og risikogruppekategorier i henhold til IMDC (*International Metastatic RCC Database Consortium*). Patienter med autoimmun sygdom eller lidelser, som krævede immunsuppression, blev ekskluderet fra studiet. Randomiseringen blev stratificeret ud fra risikokategorier (lav risiko *versus* middel risiko *versus* høj risiko) og geografisk område (Nordamerika *versus* Vesteuropa *versus* "resten af verden"). Patienterne blev randomiseret i forholdet 1:1 til en af følgende behandlingsarme:

- pembrolizumab 200 mg intravenøst hver 3. uge i kombination med axitinib 5 mg oralt, to gange dagligt. Hos patienter, som tålte axitinib 5 mg to gange dagligt i 2 konsekutive behandlingsserier (dvs. 6 uger) uden > grad 2 behandlingsrelaterede bivirkninger ved axitinib og som havde et velkontrolleret blodtryk på ≤ 150/90 mm Hg, var det tilladt at øge dosis af axitinib til 7 mg to gange dagligt. Det var tilladt at øge dosis af axitinib til 10 mg to gange dagligt ud fra de samme kriterier. Axitinib kunne pauseres eller reduceres til 3 mg to gange dagligt og efterfølgende til 2 mg to gange dagligt med henblik på at behandle toksicitet.
- sunitinib 50 mg oralt, en gang dagligt i 4 uger og herefter stop af behandling i 2 uger.

Behandlingen med pembrolizumab og axitinib fortsatte indtil sygdomsprogression defineret i henhold til RECIST v1.1 baseret på BICR-vurdering eller bekræftet af investigator, uacceptabel toksicitet, eller for for pembrolizumab, i højst 24 måneder. Administration af pembrolizumab og axitinib var tilladt efter sygdomsprogression i henhold til RECIST, hvis patienten var klinisk stabil, og investigator mente, at patienten havde en klinisk fordel. Der blev foretaget vurdering af tumorstatus ved *baseline*, efter randomiseringen i uge 12, herefter hver 6. uge indtil uge 54, og herefter hver 12. uge. Kemiske og hæmatologiske laboratorietests blev udført ved hver behandlingsserie.

123

Der blev randomiseret i alt 861 patienter. Karakteristika for studiepopulationen var: medianalder på 62 år (interval: 26 til 90); 38% i alderen 65 år eller ældre; 73% mænd; 79% kaukasere og 16% asiatere; 80% havde en Karnofsky Performance-Score (KPS) på 90-100, og 20% havde en KPS på 70-80; patientfordelingen ifølge IMDC-risikokategorier var 31% lav risiko, 56% middel risiko og 13% høj risiko.

De primære resultatmål var OS og PFS (baseret på BICR-vurdering i henhold til RECIST 1.1). De sekundære resultatmål var ORR og responsvarighed baseret på BICR-vurdering i henhold til RECIST 1.1. Den mediane opfølgningstid var 12,8 måneder (interval: 0,1 til 21,5 måneder). I tabel 28 opsummeres de vigtigste effektresultater fra den præspecificerede interimanalyse. Kaplan-Meier-kurverne for OS og PFS baseret på yderligere fire måneders opfølgning er vist i figur 16 og 17.

Tabel 28: Effektresultater i KEYNOTE-426

**Endepunkt**	**Pembrolizumab Axitinib n=432**	**Sunitinib n=429**
**OS**		
Antal hændelser (%)	59 (14%)	97 (23%)
Median i måneder (95% CI)	Ikke nået (NA; NA)	Ikke nået (NA; NA)
HR* (95% CI)	0,53 (0,38; 0,74)	
p-værdi†	0,00005	
**PFS‡**		
Antal hændelser (%)	183 (42%)	213 (50%)
Median i måneder (95% CI)	15,1 (12,6; 17,7)	11,0 (8,7; 12,5)
HR* (95% CI)	0,69 (0,56; 0,84)	
p-værdi†	0,00012	
**Objektiv responsrate**		
ORR§% (95% CI)	59 (54; 64)	36 (31; 40)
Komplet respons	6%	2%
Partielt respons	53%	34%
p-værdi¶	< 0,0001	
**Responsvarighed**		
Median i måneder (interval)	Ikke nået (1,4+; 18,2+)	15,2 (1,1+; 15,4+)
Antal (%#) patienter med varighed ≥ 6 måneder	161 (88%)	84 (81%)
Antal (%#) patienter med varighed ≥ 12 måneder	58 (71%)	26 (62%)

* Baseret på den stratificerede Cox *proportional hazard*-model
† Baseret på stratificeret log rank-test
‡ BICR-vurdering i henhold til RECIST 1.1
§ Baseret på patienter med bedste objektive respons, der er bekræftet komplet eller partielt respons
¶ Baseret på Miettinen- og Nurminen-metoden stratificeret ud fra IMDC-risikogruppe og geografisk område
\# Baseret på Kaplan-Meier-estimering
NA = Foreligger ikke

Figur 16: Kaplan-Meier-kurve for samlet overlevelse i hver behandlingsarm i KEYNOTE-426 (*intent to treat*-population)*

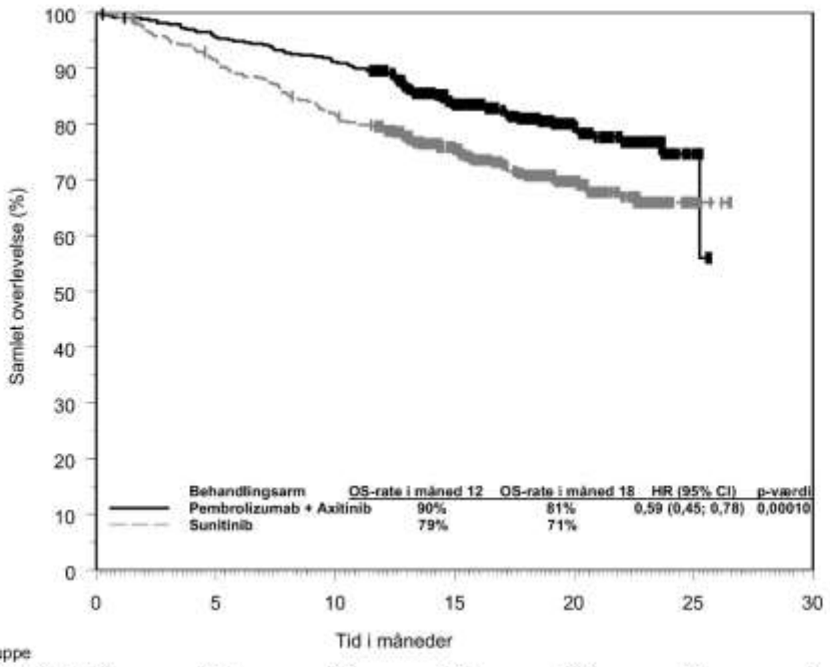

Antal i risikogruppe							
Pembrolizumab + Axitinib:	432	411	392	275	133	9	0
Sunitinib:	429	389	346	230	111	6	0

*p-værdien er nominel. Se tabel 28 for p-værdien for inferentiel test af OS baseret på den præspecificerede interimanalyse, hvor der er nået statistisk signifikans.

Figur 17: Kaplan-Meier-kurve for progressionsfri overlevelse i hver behandlingsarm i KEYNOTE-426 (*intent to treat*-population)*

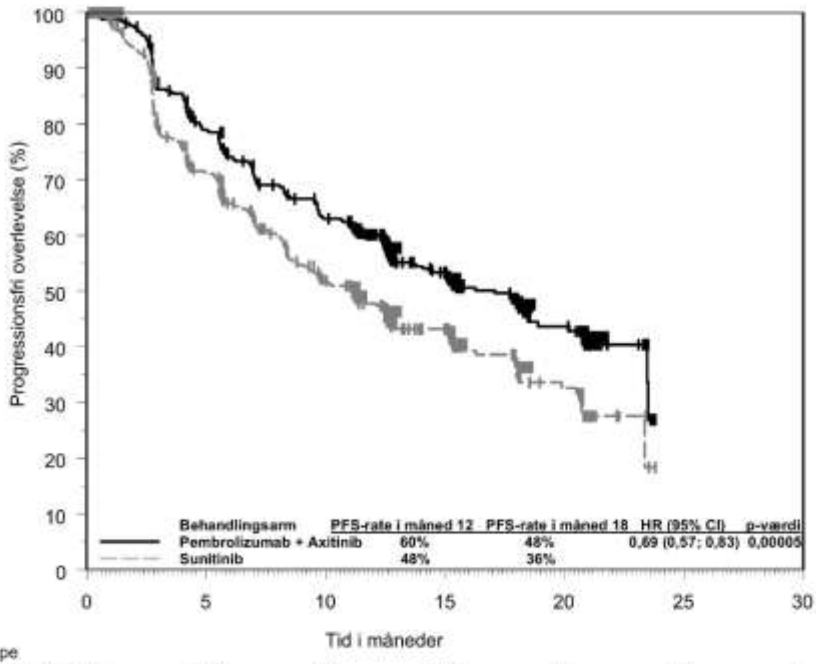

Antal i risikogruppe

	0	5	10	15	20	25	30
Pembrolizumab + Axitinib:	432	324	247	145	51	0	0
Sunitinib:	429	277	175	90	32	0	0

*p-værdien er nominel. Se tabel 28 for p-værdien for inferentiel test af PFS baseret på den præspecificerede interimanalyse, hvor der er nået statistisk signifikans.

Der blev udført subgruppeanalyser ud fra inklusion i KEYNOTE-426 hos patienter med PD-L1 CPS ≥ 1 [pembrolizumab/axitinib-kombinationen: n=243 (56%) *versus* sunitinib: n=254 (59%)]; CPS < 1 [pembrolizumab/axitinib-kombinationen: n=167 (39%) *versus* sunitinib: n=158 (37%)], og hos patienter med IMDC-risikokategorierne lav risiko [pembrolizumab/axitinib-kombinationen: n=138 (32%) *versus* sunitinib: n=131 (31%)]; middel risiko [pembrolizumab/axitinib-kombinationen: n=238 (55%) *versus* sunitinib: n=246 (57%)]; og høj risiko [pembrolizumab/axitinib-kombinationen: n=56 (13%) *versus* sunitinib: n=52 (12%)]. OS- og PFS-fordel sås uanset PD-L1-ekspressionsniveau. KEYNOTE-426-studiet var ikke designet til at vurdere virkningen i individuelle subgrupper. I tabel 29 opsummeres effektresultater ud fra IMDC-risikokategori fra den præspecificerede interimanalyse.

Tabel 29: Effektresultater i KEYNOTE-426 ud fra IMDC-risikokategori

**Endepunkt**	**Pembrolizumab + Axitinib N = 432**	**Sunitinib N = 429**	**Pembrolizumab + Axitinib *versus* Sunitinib**
**OS**	**OS-rate i måned 12, % (95% CI)**		**HR for OS (95% CI)**
Lav risiko	95,2 (89,6; 97,9)	93,8 (87,4; 97,0)	0,64 (0,24; 1,68)
Middel risiko	92,1 (84,7; 96,0)	76,7 (70,6; 81,8)	0,53 (0,35; 0,82)
Høj risiko	70,3 (56,1; 80,7)	45,2 (30,0; 59,3)	0,43 (0,23; 0,81)

PFS	Median (95% CI), måneder		HR for PFS (95% CI)
Lav risiko	17,7 (15,2; NA)	12,7 (11,5; NA)	0,81 (0,53; 1,24)
Middel risiko	14,5 (12,4; 18,0)	9,5 (8,0; 12,5)	0,69 (0,53; 0,90)
Høj risiko	4,9 (2,9; 12,4)	2,9 (2,7; 4,2)	0,58 (0,35; 0,94)
**Bekræftet ORR**	**% (95% CI)**		**ORR-forskel, % (95% CI)**
Lav risiko	66,7 (58,1; 74,5)	49,6 (40,8; 58,5)	17,0 (5,3; 28,4)
Middel risiko	59,2 (52,7; 65,5)	33,7 (27,9; 40,0)	25,5 (16,7; 33,9)
Høj risiko	41,1 (28,1; 55,0)	9,6 (3,2; 21,0)	31,5 (15,7; 46,2)

NA = Foreligger ikke

En opdateret OS-analyse blev udført, når patienterne havde en median opfølgingstid på 16,6 måneder (interval: 0,1 til 26,3 måneder). På tidspunktet for denne analyse var HR i den samlede population (95% CI): 0,59 (0,45; 0,78) med 84/432 (19,4%) hændelser i armen med kombinationsbehandling og 122/429 (28,4%) hændelser i sunitinib-armen. OS-raten i måned 12 var 89,5% (95% CI: 86,2; 92,1) for pembrolizumab i kombination med axitinib og 78,8% (95% CI: 74,7; 82,4) for sunitinib. OS-raten i måned 18 var 81,0% (95% CI: 76,7; 84,6) for pembrolizumab i kombination med axitinib og 70,7% (95% CI: 65,8; 75,1) for sunitinib. For IMDC-risikokategorien: HR for OS for risikogruppen med lav risiko var 0,94 (95% CI: 0,43; 2,07), HR for OS for risikogruppen med middel risiko var 0,52 (95% CI: 0,36; 0,75) og HR for OS for risikogruppen med høj risiko var 0,50 (95% CI: 0,29; 0,87).

Pædiatrisk population
I KEYNOTE-051 blev pembrolizumab 2 mg/kg hver 3. uge administreret til 154 pædiatriske patienter (60 børn i alderen 6 måneder til under 12 år og 94 unge i alderen 12 år til 18 år) med fremskredent melanom eller PD-L1-positive fremskredne, recidiverende eller refraktære solide tumorer eller lymfomer. Alle patienter fik pembrolizumab med en median på 3 doser (interval: 1-35 doser), hvor 130 patienter (84,4%) fik 2 eller flere doser pembrolizumab. Deltagerne blev inkluderet på tværs af 28 tumortyper ud fra primær diagnose. De hyppigste tumortyper ud fra histologi var Hodgkins lymfom (11,7%), glioblastom multiforme (9,1%), neuroblastom (6,5%), osteosarkom (6,5%) og melanom (5,2%). Ud af 154 patienter blev 134 inkluderet med solide tumorer, 18 med Hodgkins lymfom og 2 med andre lymfomer. Hos patienter med solide tumorer og andre lymfomer var ORR 5,9%, ingen patienter havde komplet respons og 8 patienter (5,9%) havde partielt respons. I populationen med Hodgkins lymfom var ORR 50,0%, 2 patienter (11,1%) havde komplet respons og 7 patienter (38,9%) havde partielt respons.

Det Europæiske Lægemiddelagentur har udsat forpligtelsen til at fremlægge resultaterne af studier med pembrolizumab i en eller flere undergrupper af den pædiatriske population ved behandling af Hodgkins lymfom (se pkt. 4.2 for oplysninger om pædiatrisk anvendelse).

5.2 Farmakokinetiske egenskaber

Pembrolizumabs farmakokinetik blev undersøgt hos 2.993 patienter med metastatisk eller inoperabelt melanom, NSCLC eller karcinom, som har fået doser i intervallet 1 til 10 mg/kg hver 2. uge, 2 til 10 mg/kg hver 3. uge eller 200 mg hver 3. uge.

Absorption
Pembrolizumab administreres intravenøst, og biotilgængeligheden er derfor øjeblikkelig og fuldstændig.

Fordeling
I overensstemmelse med en begrænset ekstravaskulær fordeling er det gennemsnitlige fordelingsvolumen for pembrolizumab ved *steady state* lille (~6,0 liter; variationskoefficient (CV) 20%). Som forventet for et antistof binder pembrolizumab ikke specifikt til plasmaproteiner.

Biotransformation
Pembrolizumab nedbrydes via non-specifikke metaboliseringsveje; metaboliseringen bidrager ikke til pembrolizumabs *clearance*.

Elimination
Pembrolizumabs *clearance* er cirka 23% lavere (geometrisk gennemsnit, 195 ml/dag [CV%: 40%]) efter at have opnået maksimal ændring ved *steady state* sammenlignet med den første dosis (252 ml/dag [CV%: 37%]). Dette fald i *clearance* over tid anses ikke for at være klinisk betydningsfuldt. Den geometriske gennemsnitsværdi (CV%) for den terminale halveringstid er 22 dage (32%) ved *steady state*.

Linearitet/non-linearitet
Eksponeringen for pembrolizumab udtrykt ved *peak*-koncentration (C_{max}) eller areal under kurven for plasmakoncentration over tid (AUC) steg dosisproportionalt inden for det terapeutiske dosisinterval. *Steady state*-koncentrationer af pembrolizumab blev nået efter 16 uger med gentagen dosering med et regime med administration hver 3. uge, og den systemiske akkumulering var cirka 2,1 gange. Den mediane dalkoncentration (C_{min}) ved *steady state* var ca. 22 µg/ml ved en dosis på 2 mg/kg hver 3. uge og 29 µg/ml ved en dosis på 200 mg hver 3. uge. Det mediane areal under kurven for koncentration over tid ved *steady state* over 3 uger ($AUC_{0\text{-}3uger}$) var 794 µg·dag/ml ved en dosis på 2 mg/kg hver 3. uge og 1.053 µg·dag/ml ved en dosis på 200 mg hver 3. uge.

Efter administration af pembrolizumab 200 mg hver 3. uge til patienter med cHL var den observerede mediane C_{min} ved *steady state* op til 40% højere end den, som blev observeret ved andre tumortyper, der blev behandlet med samme dosis. Intervallerne for dalkoncentrationerne er imidlertid sammenlignelige. Der er ingen væsentlig forskel i median C_{max} mellem cHL og andre tumortyper. Baseret på tilgængelige sikkerhedsdata for cHL og andre tumortyper er disse forskelle ikke klinisk relevante.

Særlige populationer
Indvirkningen af forskellige kovariater på pembrolizumabs farmakokinetik blev vurderet i farmakokinetiske populationsanalyser. Følgende faktorer havde ingen klinisk betydningsfuld indvirkning på pembrolizumabs *clearance*: alder (interval: 15-94 år), køn, race, let eller moderat nedsat nyrefunktion, let nedsat leverfunktion og tumorbyrde. Forholdet mellem kropsvægt og *clearance* understøtter dosering med enten fast dosis eller dosis baseret på kropsvægt til at give tilstrækkelig og sammenlignelig eksponeringskontrol. Ved vægtbaseret dosering på 2 mg/kg hver 3. uge hos pædiatriske patienter (2 til 17 år) er koncentrationen af pembrolizumab sammenlignelig med koncentrationen hos voksne ved samme dosis.

Nedsat nyrefunktion
Effekten af nedsat nyrefunktion på pembrolizumabs *clearance* blev vurderet ud fra farmakokinetiske populationsanalyser, hvor patienter med let eller moderat nedsat nyrefunktion blev sammenlignet med patienter med normal nyrefunktion. Der blev ikke påvist klinisk betydningsfulde forskelle i pembrolizumabs *clearance* mellem patienter med let eller moderat nedsat nyrefunktion og patienter med normal nyrefunktion. Pembrolizumab er ikke undersøgt hos patienter med svært nedsat nyrefunktion.

Nedsat leverfunktion
Effekten af nedsat leverfunktion på pembrolizumabs *clearance* blev vurderet ud fra farmakokinetiske populationsanalyser, hvor patienter med let nedsat leverfunktion (defineret i henhold til *US National Cancer Institute* (NCI) kriterier for leverinsufficiens) blev sammenlignet med patienter med normal leverfunktion. Der blev ikke påvist klinisk betydningsfulde forskelle i pembrolizumabs *clearance* mellem patienter med let nedsat leverfunktion og patienter med normal leverfunktion. Pembrolizumab er ikke undersøgt hos patienter med moderat eller svært nedsat leverfunktion (se pkt. 4.2).

5.3 Non-kliniske sikkerhedsdata

Pembrolizumabs sikkerhed blev vurderet i toksicitetsstudier med gentagne doser af 1 måned og 6 måneders varighed, hvor cynomolgusaber fik administreret intravenøse doser på 6, 40 eller 200 mg/kg en gang om ugen i studiet af 1 måneds varighed og en gang hver anden uge i studiet af 6 måneders varighed, efterfulgt af en behandlingsfri periode på 4 måneder. Der blev ikke observeret fund af

toksikologisk betydning, og NOAEL-værdien (*No Observed Adverse Effect Level*) i begge studier var ≥ 200 mg/kg, hvilket gav multiple eksponeringer på 19 og 94 gange eksponeringen hos mennesker ved doser på henholdsvis 10 og 2 mg/kg. Den multiple eksponering mellem NOAEL og en human dosis på 200 mg var 74.

Der er ikke udført reproduktionsstudier med pembrolizumab hos dyr. Det menes, at PD-1/PD-L1-signalvejen er involveret i at opretholde tolerancen over for fostret under graviditeten. I dyremodeller med drægtige mus er det blevet påvist, at blokering af PD-L1-signalet kan påvirke tolerancen for fostret og medføre et stigende antal fostertab.

Der er ikke udført fertilitetsstudier med pembrolizumab hos dyr. Der var ingen betydningsfuld indvirkning på forplantningsorganerne hos han- og hunaber i toksicitetsstudier af 1 måned og 6 måneders varighed med gentagne doser. Mange dyr i disse studier var imidlertid ikke kønsmodne.

6. FARMACEUTISKE OPLYSNINGER

6.1 Hjælpestoffer

L-histidin
L-histidin-hydrochloridmonohydrat
Saccharose
Polysorbat 80
Vand til injektionsvæsker

6.2 Uforligeligheder

Da der ikke foreligger studier af eventuelle uforligeligheder, må dette lægemiddel ikke blandes med andre lægemidler end dem, der er anført under pkt. 6.6.

6.3 Opbevaringstid

Uåbnet hætteglas
2 år.

Efter klargøring af infusionsvæsken
Ud fra en mikrobiologisk synsvinkel skal lægemidlet anvendes straks efter fortynding. Den fortyndede infusionsvæske må ikke nedfryses. Hvis KEYTRUDA ikke anvendes straks, er der påvist kemisk og fysisk stabilitet i 96 timer ved 2 °C-8 °C. Disse 96 timer kan inkludere opbevaring i op til 6 timer ved stuetemperatur (ved eller under 25 °C). Hætteglassene og/eller de intravenøse infusionsposer skal opnå stuetemperatur inden brug, hvis de har været opbevaret i køleskab.

6.4 Særlige opbevaringsforhold

Opbevares i køleskab (2 °C – 8 °C).
Må ikke nedfryses.
Opbevares i den originale karton for at beskytte mod lys.

Opbevaringsforhold efter fortynding af lægemidlet, se pkt. 6.3.

6.5 Emballagetype og pakningsstørrelser

4 ml koncentrat i et 10 ml klart hætteglas (type I-glas) med en coated grå chlorbutylprop og en aluminiumshætte med et mørkeblåt flip-off-låg, der indeholder 100 mg pembrolizumab.

Hver karton indeholder et hætteglas.

6.6 Regler for bortskaffelse og anden håndtering

Klargøring og administration af infusionsvæske

- Hætteglasset må ikke rystes.
- Lad hætteglasset opnå stuetemperatur (ved temperaturer på eller under 25 °C).
- Før fortynding kan hætteglasset med koncentrat opbevares uden for køleskab (ved temperaturer på eller under 25 °C) i op til 24 timer.
- Parenterale lægemidler skal inspiceres visuelt for partikler og misfarvning inden administration. KEYTRUDA-koncentratet er en klar til let opaliserende og farveløs til lysegul opløsning. Hætteglasset skal kasseres, hvis der observeres synlige partikler.
- Træk det påkrævede volumen (op til 4 ml (100 mg)) koncentrat op og overfør det til en intravenøs infusionspose, der indeholder natriumchlorid 9 mg/ml (0,9%) eller glucose 50 mg/ml (5%) for at fremstille den færdige infusionsvæske med en endelig koncentration på 1 til 10 mg/ml. Hvert hætteglas indeholder et overskud på 0,25 ml (samlet indhold pr. hætteglas 4,25 ml) for at sikre, at der kan udtrækkes 4 ml koncentrat. Bland den færdige infusionsvæske ved forsigtigt at vende infusionsposen.
- Ud fra en mikrobiologisk synsvinkel skal lægemidlet anvendes straks efter fortynding. Den fortyndede infusionsvæske må ikke nedfryses. Hvis KEYTRUDA ikke anvendes straks, er der påvist kemisk og fysisk stabilitet i 96 timer ved 2 °C-8 °C. Disse 96 timer kan inkludere opbevaring i op til 6 timer ved stuetemperatur (ved eller under 25 °C). Hætteglassene og/eller de intravenøse infusionsposer skal opnå stuetemperatur inden brug, hvis de har været opbevaret i køleskab. Gennemsigtige til hvide proteinholdige partikler kan forekomme i den fortyndede infusionsvæske. Administrer infusionsvæsken intravenøst over en periode på 30 minutter med et infusionssæt med et *in-line* eller *add-on* sterilt, ikke-pyrogent, minimalt proteinbindende filter (porestørrelse 0,2 til 5 µm).
- KEYTRUDA må ikke administreres sammen med andre lægemidler i samme infusionsslange.
- KEYTRUDA er kun til engangsbrug. Den ubrugte rest i hætteglasset bortskaffes.

Ikke anvendt lægemiddel samt affald heraf skal bortskaffes i henhold til lokale retningslinjer.

7. INDEHAVER AF MARKEDSFØRINGSTILLADELSEN

Merck Sharp & Dohme B.V.
Waarderweg 39
2031 BN Haarlem
Holland

8. MARKEDSFØRINGSTILLADELSESNUMMER (-NUMRE)

EU/1/15/1024/002

9. DATO FOR FØRSTE MARKEDSFØRINGSTILLADELSE/FORNYELSE AF TILLADELSEN

Dato for første markedsføringstilladelse: 17. juli 2015
Dato for seneste fornyelse: 24. marts 2020

10. DATO FOR ÆNDRING AF TEKSTEN

Yderligere oplysninger om dette lægemiddel findes på Det Europæiske Lægemiddelagenturs hjemmeside http://www.ema.europa.eu.

BILAG II

A. **FREMSTILLERE AF DET BIOLOGISK AKTIVE STOF OG FREMSTILLERE ANSVARLIGE FOR BATCHFRIGIVELSE**

B. **BETINGELSER ELLER BEGRÆNSNINGER VEDRØRENDE UDLEVERING OG ANVENDELSE**

C. **ANDRE FORHOLD OG BETINGELSER FOR MARKEDSFØRINGSTILLADELSEN**

D. **BETINGELSER ELLER BEGRÆNSNINGER MED HENSYN TIL SIKKER OG EFFEKTIV ANVENDELSE AF LÆGEMIDLET**

A. FREMSTILLERE AF DET BIOLOGISK AKTIVE STOF OG FREMSTILLERE ANSVARLIGE FOR BATCHFRIGIVELSE

Navn og adresse på fremstillerne af det biologisk aktive stof

AstraZeneca Pharmaceuticals LP,
Frederick Manufacturing Center (FMC)
633 Research Court
Frederick, Maryland (MD) 21703
USA

Boehringer Ingelheim (BIB)
Pharma GmbH & Co. KG
Birkendorfer Straße 65
88397 Biberach an der Riss
Tyskland

N.V. Organon
Veersemeer 4
5347 JN Oss
Holland

Navn og adresse på den fremstiller, der er ansvarlig for batchfrigivelse

Schering-Plough Labo NV
Industriepark 30, Heist-op-den-Berg
B-2220, Belgien

B. BETINGELSER ELLER BEGRÆNSNINGER VEDRØRENDE UDLEVERING OG ANVENDELSE

Lægemidlet må kun udleveres efter ordination på en recept udstedt af en begrænset lægegruppe (se bilag I: Produktresumé, pkt. 4.2).

C. ANDRE FORHOLD OG BETINGELSER FOR MARKEDSFØRINGSTILLADELSEN

- **Periodiske, opdaterede sikkerhedsindberetninger (PSUR'er)**

Kravene for fremsendelse af PSUR'er for dette lægemiddel fremgår af listen over EU-referencedatoer (EURD list), som fastsat i artikel 107c, stk. 7, i direktiv 2001/83/EF, og alle efterfølgende opdateringer offentliggjort på Det Europæiske Lægemiddelagenturs hjemmeside http://www.ema.europa.eu.

D. BETINGELSER ELLER BEGRÆNSNINGER MED HENSYN TIL SIKKER OG EFFEKTIV ANVENDELSE AF LÆGEMIDLET

- **Risikostyringsplan (RMP)**

Indehaveren af markedsføringstilladelsen skal udføre de påkrævede aktiviteter og foranstaltninger vedrørende lægemiddelovervågning, som er beskrevet i den godkendte RMP, der fremgår af modul 1.8.2 i markedsføringstilladelsen, og enhver efterfølgende godkendt opdatering af RMP.

En opdateret RMP skal fremsendes:
- på anmodning fra Det Europæiske Lægemiddelagentur
- når risikostyringssystemet ændres, særlig som følge af, at der er modtaget nye oplysninger, der kan medføre en væsentlig ændring i benefit/risk-forholdet, eller som følge af, at en vigtig milepæl (lægemiddelovervågning eller risikominimering) er nået.

- **Yderligere risikominimeringsforanstaltninger**

Forud for lancering af KEYTRUDA i hvert enkelt medlemsland skal indehaveren af markedsføringstilladelsen aftale indholdet og udformningen af uddannelsesprogrammet for KEYTRUDA, herunder en kommunikationsplan, distributionsmåde og andre aspekter af programmet, med den relevante nationale myndighed.

Formålet med uddannelsesprogrammet er at øge patienternes og/eller omsorgsgivernes bevidsthed om tegn og symptomer, der er relevante for tidlig erkendelse/identifikation af potentielle immunrelaterede bivirkninger.

I alle medlemslande, hvor KEYTRUDA markedsføres, skal indehaveren af markedsføringstilladelsen sikre, at alle sundhedspersoner og patienter/omsorgsgivere, der forventes at ordinere og anvende KEYTRUDA, er forsynet med/får udleveret uddannelsesmateriale til patienter.

Uddannelsesmaterialet til patienter skal indeholde:
- Patientinformationsbrochure
- Patientinformationskortet

Patientinformationsbrochuren og patientinformationskortet skal indeholde følgende væsentlige punkter:
- Beskrivelse af de vigtigste tegn eller symptomer på immunrelaterede bivirkninger og vigtigheden af straks at fortælle det til den behandlende læge, hvis symptomer opstår
- Vigtigheden af ikke selv at behandle eventuelle symptomer uden først at tale med en sundhedsperson
- Vigtigheden af altid at have patientinformationskortet på sig og at vise det ved alle besøg hos andre læger (f.eks. sundhedspersoner på skadestue) end den ordinerende læge.

Kortet minder patienten om de vigtigste symptomer, som straks skal rapporteres til lægen/sygeplejersken. Det indeholder også en opfordring om at påføre lægens kontaktoplysninger og om at informere andre læger om, at patienten er i behandling med KEYTRUDA

- **Forpligtelse til at gennemføre foranstaltninger efter udstedelse af markedsføringstilladelse**

Indehaveren af markedsføringstilladelsen skal inden for den fastsatte tidsramme gennemføre følgende foranstaltninger:

Beskrivelse	Tidsfrist
1. Virkningsstudie efter tilladelse til markedsføring (PAES): Indehaveren af markedsføringstilladelsen skal indsende den endelige studierapport for studie P087: Et klinisk fase II-studie af MK-3475 (pembrolizumab) hos forsøgspersoner med recidiverende eller refraktært (R/R) klassisk Hodgkins lymfom (cHL) – Endelig studierapport	3. kvartal 2021
2. Virkningsstudie efter tilladelse til markedsføring (PAES): Indehaveren af markedsføringstilladelsen skal indsende den endelige studierapport for studie P204: Et randomiseret, åbent klinisk fase III-studie, der sammenligner pembrolizumab med brentuximab vedotin hos forsøgspersoner med recidiverende eller refraktært klassisk Hodgkins lymfom – Endelig studierapport	2. kvartal 2021

Beskrivelse	Tidsfrist
3. Værdien af biomarkører til at forudsige pembrolizumabs virkning skal undersøges yderligere, især:<br><br>Yderligere biomarkører, ud over PD-L1-ekspressionsstatus ved immunhistokemi (IHC) (f.eks. PD-L2, RNA-signatur osv.), der er prædiktive for pembrolizumabs virkning, skal undersøges sammen med yderligere information om mønstret for PD-L1-ekspression indhentet i igangværende NSCLC-studier (P001, P010, P024 og P042)<br>og studier med urotelialt karcinom (KN045, KN052),<br>HNSCC-studiet (KN040) og<br>studiet med adjuverende behandling af resekteret stadie II-melanom (KN716):<br>• Genomanalyser med fuld exomsekventering og/eller RNAseq (f.eks. Nanostring RNA-gensignatur)<br>• IHC-farvning for PD-L2<br>• Data om RNA og proteomisk profilering af serum	2. kvartal 2020<br>2. kvartal 2019<br>4. kvartal 2021<br>4. kvartal 2024
4. Virkningsstudie efter tilladelse til markedsføring (PAES): Indehaveren af markedsføringstilladelsen skal sørge for studierapporten for studie P361: Et randomiseret kontrolleret klinisk fase III-studie af pembrolizumab med eller uden platinbaseret kombineret kemoterapi *versus* kemoterapi hos forsøgspersoner med fremskredent eller metastatisk urotelialt karcinom – Studierapport	2. kvartal 2020
5. Virkningsstudie efter tilladelse til markedsføring (PAES): Indehaveren af markedsføringstilladelsen skal indsende den endelige studierapport med RFS/DMFS- og OS-data for studie KN054: Et klinisk fase III-studie af pembrolizumab (MK-3475) hos forsøgspersoner med komplet resektion af højrisiko stadie III-melanom – Endelig studierapport	4. kvartal 2023
6. Virkningsstudie efter tilladelse til markedsføring (PAES): Indehaveren af markedsføringstilladelsen skal indsende den endelige studierapport for studie P407: Et randomiseret, dobbeltblindet fase III-studie af carboplatin - paclitaxel/nab-paclitaxel-kemoterapi med eller uden pembrolizumab (MK-3475) som førstelinjebehandling hos forsøgspersoner med metastatisk planocellulær ikke-småcellet lungecancer – Endelig studierapport	3. kvartal 2021
7. Virkningsstudie efter tilladelse til markedsføring (PAES): Indehaveren af markedsføringstilladelsen skal indsende den endelige studierapport for studie P426: Et randomiseret, åbent fase III-studie, der vurderer pembrolizumabs (MK-3475) virkning og sikkerhed i kombination med axitinib *versus* sunitinib som monoterapi som førstelinjebehandling for lokalt fremskredent eller metastatisk renalcellekarcinom (mRCC)	1. kvartal 2021

BILAG III

ETIKETTERING OG INDLÆGSSEDDEL

A. ETIKETTERING

MÆRKNING, DER SKAL ANFØRES PÅ DEN YDRE EMBALLAGE

YDRE KARTON

1. LÆGEMIDLETS NAVN

KEYTRUDA 50 mg pulver til koncentrat til infusionsvæske, opløsning
pembrolizumab

2. ANGIVELSE AF AKTIVT STOF/AKTIVE STOFFER

Et hætteglas med pulver indeholder 50 mg pembrolizumab. Efter rekonstitution indeholder 1 ml koncentrat 25 mg pembrolizumab.

3. LISTE OVER HJÆLPESTOFFER

Hjælpestoffer: L-histidin, L-histidin-hydrochloridmonohydrat, saccharose, polysorbat 80.

4. LÆGEMIDDELFORM OG INDHOLD (PAKNINGSSTØRRELSE)

pulver til koncentrat til infusionsvæske, opløsning
1 hætteglas

5. ANVENDELSESMÅDE OG ADMINISTRATIONSVEJ(E)

Intravenøs anvendelse.
Kun til engangsbrug.
Læs indlægssedlen inden brug.

6. SÆRLIG ADVARSEL OM, AT LÆGEMIDLET SKAL OPBEVARES UTILGÆNGELIGT FOR BØRN

Opbevares utilgængeligt for børn.

7. EVENTUELLE ANDRE SÆRLIGE ADVARSLER

8. UDLØBSDATO

EXP

Rekonstituerede hætteglas og/eller fortyndede intravenøse infusionsposer kan opbevares sammenlagt op til 96 timer i køleskab (2 °C - 8 °C).

9.	SÆRLIGE OPBEVARINGSBETINGELSER

Opbevares i køleskab (2 °C - 8 °C).

10.	EVENTUELLE SÆRLIGE FORHOLDSREGLER VED BORTSKAFFELSE AF IKKE ANVENDT LÆGEMIDDEL SAMT AFFALD HERAF

11.	NAVN OG ADRESSE PÅ INDEHAVEREN AF MARKEDSFØRINGSTILLADELSEN

Merck Sharp & Dohme B.V.
Waarderweg 39
2031 BN Haarlem
Holland

12.	MARKEDSFØRINGSTILLADELSESNUMMER (-NUMRE)

EU/1/15/1024/001 (1 hætteglas)

13.	BATCHNUMMER

Lot

14.	GENEREL KLASSIFIKATION FOR UDLEVERING

15.	INSTRUKTIONER VEDRØRENDE ANVENDELSEN

16.	INFORMATION I BRAILLESKRIFT

Fritaget fra krav om brailleskrift

17.	ENTYDIG IDENTIFIKATOR – 2D-STREGKODE

Der er anført en 2D-stregkode, som indeholder en entydig identifikator.

18.	ENTYDIG IDENTIFIKATOR - MENNESKELIGT LÆSBARE DATA

PC
SN
NN

MINDSTEKRAV TIL MÆRKNING PÅ SMÅ INDRE EMBALLAGER<br><br>HÆTTEGLAS

1. LÆGEMIDLETS NAVN OG ADMINISTRATIONSVEJ(E)

KEYTRUDA 50 mg pulver til koncentrat til infusionsvæske, opløsning
pembrolizumab
Intravenøs anvendelse
i.v.

2. ADMINISTRATIONSMETODE

3. UDLØBSDATO

EXP

4. BATCHNUMMER

Lot

5. INDHOLD ANGIVET SOM VÆGT, VOLUMEN ELLER ENHEDER

6. ANDET

MÆRKNING, DER SKAL ANFØRES PÅ DEN YDRE EMBALLAGE

YDRE KARTON

1. LÆGEMIDLETS NAVN

KEYTRUDA 25 mg/ml koncentrat til infusionsvæske, opløsning
pembrolizumab
100 mg/4 ml

2. ANGIVELSE AF AKTIVT STOF/AKTIVE STOFFER

Et hætteglas med 4 ml indeholder 100 mg pembrolizumab. Hver ml koncentrat indeholder 25 mg pembrolizumab.

3. LISTE OVER HJÆLPESTOFFER

Hjælpestoffer: L-histidin, L-histidin-hydrochloridmonohydrat, saccharose, polysorbat 80, vand til injektionsvæsker.

4. LÆGEMIDDELFORM OG INDHOLD (PAKNINGSSTØRRELSE)

Koncentrat til infusionsvæske, opløsning
1 hætteglas

5. ANVENDELSESMÅDE OG ADMINISTRATIONSVEJ(E)

Intravenøs anvendelse efter fortynding.
Kun til engangsbrug.
Læs indlægssedlen inden brug.

6. SÆRLIG ADVARSEL OM, AT LÆGEMIDLET SKAL OPBEVARES UTILGÆNGELIGT FOR BØRN

Opbevares utilgængeligt for børn.

7. EVENTUELLE ANDRE SÆRLIGE ADVARSLER

Må ikke rystes.

8. UDLØBSDATO

EXP

Den fortyndede infusionsvæske kan opbevares op til 96 timer i køleskab (2 °C - 8 °C).

9. SÆRLIGE OPBEVARINGSBETINGELSER

Opbevares i køleskab (2 °C - 8 °C).
Må ikke nedfryses.
Opbevares i den originale karton for at beskytte mod lys.

10. EVENTUELLE SÆRLIGE FORHOLDSREGLER VED BORTSKAFFELSE AF IKKE ANVENDT LÆGEMIDDEL SAMT AFFALD HERAF

11. NAVN OG ADRESSE PÅ INDEHAVEREN AF MARKEDSFØRINGSTILLADELSEN

Merck Sharp & Dohme B.V.
Waarderweg 39
2031 BN Haarlem
Holland

12. MARKEDSFØRINGSTILLADELSESNUMMER (-NUMRE)

EU/1/15/1024/002 (1 hætteglas)

13. BATCHNUMMER

Lot

14. GENEREL KLASSIFIKATION FOR UDLEVERING

15. INSTRUKTIONER VEDRØRENDE ANVENDELSEN

16. INFORMATION I BRAILLESKRIFT

Fritaget fra krav om brailleskrift

17. ENTYDIG IDENTIFIKATOR – 2D-STREGKODE

Der er anført en 2D-stregkode, som indeholder en entydig identifikator.

18. ENTYDIG IDENTIFIKATOR - MENNESKELIGT LÆSBARE DATA

PC
SN
NN

MINDSTEKRAV TIL MÆRKNING PÅ SMÅ INDRE EMBALLAGER

HÆTTEGLAS

1. LÆGEMIDLETS NAVN OG ADMINISTRATIONSVEJ(E)

KEYTRUDA 25 mg/ml koncentrat til infusionsvæske, opløsning
pembrolizumab
100 mg/4 ml
i.v.

2. ADMINISTRATIONSMETODE

3. UDLØBSDATO

EXP

4. BATCHNUMMER

Lot

5. INDHOLD ANGIVET SOM VÆGT, VOLUMEN ELLER ENHEDER

6. ANDET

B. INDLÆGSSEDDEL

Indlægsseddel: Information til patienten

Keytruda 50 mg pulver til koncentrat til infusionsvæske, opløsning
pembrolizumab

Læs denne indlægsseddel grundigt, inden du får dette lægemiddel, da den indeholder vigtige oplysninger.
- Gem indlægssedlen. Du kan få brug for at læse den igen.
- Det er vigtigt, at du har patientinformationskortet med dig under behandlingen.
- Spørg lægen, hvis der er mere, du vil vide.
- Kontakt lægen, hvis du får bivirkninger, herunder bivirkninger, som ikke er nævnt i denne indlægsseddel. Se punkt 4.

Se den nyeste indlægsseddel på www.indlaegsseddel.dk

Oversigt over indlægssedlen

1. Virkning og anvendelse
2. Det skal du vide, før du får Keytruda
3. Sådan får du Keytruda
4. Bivirkninger
5. Opbevaring
6. Pakningsstørrelser og yderligere oplysninger

1. Virkning og anvendelse

Keytruda indeholder det aktive stof pembrolizumab, som er et monoklonalt antistof. Keytruda styrker immunsystemets evne til at bekæmpe kræft.

Keytruda anvendes til voksne til at behandle:
- en bestemt type hudkræft kaldet melanom (modermærkekræft)
- en bestemt type lungekræft kaldet ikke-småcellet lungekræft
- en bestemt type lymfeknudekræft kaldet klassisk Hodgkins lymfom
- en bestemt type blærekræft kaldet urotelialt karcinom
- en bestemt type hoved-halskræft kaldet planocellulært hoved-hals karcinom
- en bestemt type nyrekræft kaldet renalcellekarcinom.

Keytruda gives til patienter, når deres kræft har spredt sig eller ikke kan fjernes ved en operation.

Keytruda gives til patienter, når de har fået foretaget en operation for at fjerne modermærkekræft for at forebygge, at deres kræft vender tilbage (adjuverende behandling).

Keytruda kan gives i kombination med anden kræftmedicin. Det er vigtigt, at du også læser indlægssedlerne for disse andre lægemidler. Spørg lægen, hvis du har eventuelle spørgsmål vedrørende disse lægemidler.

2. Det skal du vide, før du får Keytruda

Du må ikke få Keytruda
- hvis du er allergisk over for pembrolizumab eller et af de øvrige indholdsstoffer i Keytruda (angivet i punkt 6 "Pakningsstørrelser og yderligere oplysninger"). Er du i tvivl, så spørg lægen.

Advarsler og forsigtighedsregler
Kontakt lægen eller sygeplejersken, før du får Keytruda.

Fortæl det til lægen, før du får Keytruda:
- hvis du har en autoimmun sygdom (en tilstand, hvor kroppen angriber sine egne celler)
- hvis du har lungebetændelse eller inflammation (en betændelseslignende tilstand) i lungerne (pneumonitis)
- hvis du tidligere har fået ipilimumab, der er et andet lægemiddel til behandling af melanom, og har oplevet alvorlige bivirkninger på grund af dette lægemiddel
- hvis du har haft en allergisk reaktion ved behandling med andre monoklonale antistoffer
- hvis du har eller har haft en kronisk virusinfektion i leveren, herunder hepatitis B (hbv) eller hepatitis C (hcv)
- hvis du har hiv- (human immundefekt virus) infektion eller aids (erhvervet immundefekt syndrom)
- hvis du har leverskader
- hvis du har nyreskader
- hvis du har gennemgået en transplantation af et fast organ eller en knoglemarvstransplantation (stamcelletransplantation), hvor der anvendes stamceller fra en donor (allogen transplantation).

Når du får Keytruda, kan du få nogle alvorlige bivirkninger. Disse bivirkninger kan nogle gange blive livstruende og kan medføre døden. Disse bivirkninger kan forekomme når som helst under behandlingen, eller selv efter afslutning af din behandling. Du kan få mere end en bivirkning på samme tid.

Kontakt straks lægen, hvis du får en eller flere af følgende tilstande. Din læge vil muligvis give dig anden medicin for at forebygge mere alvorlige komplikationer og mildne dine symptomer. Din læge vil muligvis udsætte den næste dosis af Keytruda eller helt stoppe din behandling med Keytruda.
- inflammation (en betændelseslignende tilstand) i lungerne, som kan inkludere kortåndethed, brystsmerter eller hoste
- betændelse i tarmene, som kan inkludere diarré eller flere afføringer end normalt, sort, tjæreagtig, klistret afføring, blod eller slim i afføringen, svære mavesmerter eller ømhed i maveområdet, kvalme, opkastning
- leverbetændelse, som kan inkludere kvalme eller opkastning, nedsat appetit, smerter i højre side af maven, gulfarvning af huden eller det hvide i øjnene, mørk urin, øget blødningstendens eller tendens til lettere at få blå mærker
- inflammation i nyrerne, som kan inkludere ændringer i mængden eller farven af urinen
- inflammation i hormonproducerende kirtler (især skjoldbruskkirtlen, hypofysen og binyrerne), som kan inkludere hurtig hjerterytme (puls), vægttab, øget svedtendens, vægtstigning, hårtab, kuldefornemmelse, forstoppelse, dybere stemme, muskelsmerter, svimmelhed eller besvimelse, hovedpine, som ikke vil forsvinde, eller usædvanlig hovedpine
- type 1-diabetes (sukkersyge), som kan inkludere øget appetit eller tørst, hyppigere vandladning eller vægttab
- inflammation i øjnene, som kan inkludere synsændringer
- inflammation i musklerne, som kan inkludere muskelsmerter eller muskelsvaghed
- inflammation i hjertemusklen, som kan inkludere kortåndethed, uregelmæssig hjerterytme, træthed eller brystsmerter
- inflammation i bugspytkirtlen, som kan inkludere mavesmerter, kvalme og opkastning
- inflammation i huden, som kan inkludere udslæt, kløe, blæredannelse i huden, afskalning af huden eller sårdannelse, og/eller læsioner i munden eller i slimhinderne i næsen, halsen eller i området omkring kønsorganerne
- en sygdom i immunsystemet, der kan ramme lungerne, huden, øjnene og/eller lymfeknuderne (sarkoidose)
- inflammation i hjernen, som kan inkludere forvirring, feber, hukommelsesproblemer eller krampeanfald (encephalitis)
- smerter, følelsesløshed, en snurrende fornemmelse eller svaghed i arme og ben; blære- eller tarmproblemer inklusive behov for hyppigere vandladning, ufrivillig vandladning, vandladningsbesvær og forstoppelse (myelitis (rygmarvsbetændelse))
- infusionsreaktioner, som kan inkludere kortåndethed, kløe eller udslæt, svimmelhed eller feber.

Komplikationer, inklusive *graft versus host*-sygdom (GVHD), hos personer der gennemgår knoglemarvstransplantation (stamcelletransplantation), hvor der anvendes stamceller fra en donor (allogen transplantation). Disse komplikationer kan være alvorlige og kan medføre døden. De kan

forekomme, hvis du tidligere har gennemgået denne form for transplantation, eller hvis du skal have en sådan transplantation engang i fremtiden. Lægen vil overvåge dig for tegn og symptomer, som kan inkludere hududslæt, leverbetændelse, mavesmerter eller diarré.

Børn og unge
Keytruda må ikke anvendes til børn og unge under 18 år.

Brug af anden medicin sammen med Keytruda
Fortæl det altid til lægen,
- hvis du tager anden medicin, som svækker dit immunsystem. Eksempler herpå kan være kortikosteroider, som f.eks. prednison. Disse typer medicin kan påvirke virkningen af Keytruda. Når du er i behandling med Keytruda, kan lægen imidlertid godt give dig kortikosteroider for at mildne de bivirkninger, du kan få af Keytruda. Du kan også få kortikosteroider, før du får Keytruda i kombination med kemoterapi for at forebygge og/eller behandle kvalme, opkastning og andre bivirkninger forårsaget af kemoterapi.
- hvis du tager anden medicin, for nylig har taget anden medicin eller planlægger at tage anden medicin.

Graviditet
- Du må ikke få Keytruda, hvis du er gravid, medmindre lægen specifikt anbefaler det.
- Fortæl det til lægen, hvis du er gravid, har mistanke om, at du er gravid, eller planlægger at blive gravid.
- Keytruda kan skade det ufødte barn eller øge risikoen for, at det dør.
- Hvis du er en kvinde i den fødedygtige alder, skal du anvende sikker prævention, mens du bliver behandlet med Keytruda og i mindst 4 måneder efter din sidste dosis.

Amning
- Fortæl det til lægen, hvis du ammer.
- Du må ikke amme, mens du får Keytruda.
- Det er ukendt, om Keytruda udskilles i mælken hos mennesker.

Trafik- og arbejdssikkerhed
Keytruda påvirker i mindre grad evnen til at føre motorkøretøj og betjene maskiner. Svimmelhed, træthed eller svaghedsfølelse er mulige bivirkninger ved Keytruda. Undlad at føre motorkøretøj og betjene maskiner, hvis du har fået Keytruda, medmindre du er sikker på, at du har det godt.

3. Sådan får du Keytruda

Du får Keytruda på et hospital under tilsyn af en læge med erfaring i behandling af kræft.
- Den anbefalede dosis af Keytruda er enten 200 mg hver 3. uge eller 400 mg hver 6. uge.
- Lægen vil give dig Keytruda som en infusion i en vene (intravenøst) over en periode på ca. 30 minutter.
- Lægen beslutter, hvor mange behandlinger du har brug for.

Hvis du glemmer en aftale om at få behandling med Keytruda
- Kontakt straks lægen for at lave en ny aftale.
- Det er meget vigtigt, at du ikke springer en infusion med dette lægemiddel over.

Hvis du holder op med at få Keytruda
Hvis du stopper behandlingen, kan virkningen af medicinen ophøre. Du må ikke stoppe behandlingen med Keytruda, medmindre du har aftalt det med lægen.
Spørg lægen, hvis der er noget, du er i tvivl om.

Du kan også finde disse oplysninger på det patientinformationskort, du har fået af din læge. Det er vigtigt, at du gemmer dette patientinformationskort og viser det til din partner eller dine omsorgspersoner.

4. Bivirkninger

Dette lægemiddel kan som alle andre lægemidler give bivirkninger, men ikke alle får bivirkninger.

Når du får Keytruda, kan du få nogle alvorlige bivirkninger. Se punkt 2.

Følgende bivirkninger er indberettet med pembrolizumab alene:

Meget almindelig (kan forekomme hos flere end 1 ud af 10 personer)
- nedsat antal røde blodlegemer
- nedsat funktion af skjoldbruskkirtlen
- nedsat appetit
- hovedpine
- kortåndethed; hoste
- diarré; mavesmerter; kvalme; opkastning; forstoppelse
- kløe; hududslæt
- smerter i muskler og knogler; ledsmerter
- træthedsfølelse; usædvanlig træthed eller svaghed; hævelser; feber.

Almindelig (kan forekomme hos op til 1 ud af 10 personer)
- lungeinfektion (lungebetændelse)
- nedsat antal blodplader (øget tendens til at få blå mærker eller øget blødningstendens); nedsat antal hvide blodlegemer (lymfocytter)
- reaktion i forbindelse med infusion af medicinen
- overaktiv skjoldbruskkirtel; hedeture
- nedsat indhold af natrium, kalium eller kalcium i blodet
- søvnbesvær
- svimmelhed; inflammation i nerverne, som medfører følelsesløshed, svaghed og en snurrende fornemmelse eller brændende smerter i arme og ben; manglende energi; ændret smagssans
- tørre øjne
- unormal hjerterytme
- højt blodtryk
- inflammation i lungerne
- inflammation i tarmene; mundtørhed
- rødt hævet hududslæt, nogle gange med blæredannelse; pletvise farveændringer i huden (hvide pletter); tør, kløende hud; hårtab; aknelignende hudproblemer
- muskelsmerter eller muskelømhed; smerter i arme eller ben; ledsmerter med hævelser
- kuldegysninger; influenzalignende sygdom
- forhøjede leverenzymværdier i blodet; forhøjet indhold af kalcium i blodet; unormale prøveresultater for nyrefunktion.

Ikke almindelig (kan forekomme hos op til 1 ud af 100 personer)
- nedsat antal hvide blodlegemer (neutrofiler, leukocytter og eosinofiler)
- en sygdom i immunsystemet, der kan ramme lungerne, huden, øjnene og/eller lymfeknuderne (sarkoidose)
- inflammation i hypofysen, som sidder i bunden af hjernen; nedsat udskillelse af hormoner produceret af binyrerne; inflammation i skjoldbruskkirtlen
- type 1-diabetes (sukkersyge)
- krampeanfald
- inflammation i øjnene; øjensmerter, øjenirritation, kløe eller røde øjne; ubehagelig lysfølsomhed; pletter for øjnene
- betændelse i hjertesækken; ophobning af væske omkring hjertet
- inflammation i bugspytkirtlen
- et sår i slimhinden i mavesækken eller i øvre del af tyndtarmen
- inflammation i leveren

- fortykkelse og nogle gange afskalning af huden; inflammation i huden; ændret hårfarve; små buler eller sår på huden
- seneskedehindebetændelse
- inflammation i nyrerne
- forhøjet indhold af amylase, et enzym, som nedbryder stivelse.

Sjælden (kan forekomme hos op til 1 ud af 1.000 personer)
- betændelsesreaktion (inflammatorisk respons) på blodplader eller røde blodlegemer; følelse af svaghed, svimmelhed, kortåndethed, eller hvis du har bleg hud (tegn på et nedsat antal røde blodlegemer, der muligvis skyldes en type blodmangel (anæmi) kaldet ren erytrocyt aplasi); en tilstand kaldet hæmofagocytisk lymfohistiocytose, hvor immunsystemet danner for mange infektionsbekæmpende celler kaldet histiocytter og lymfocytter, som kan give forskellige symptomer
- inflammation i hjernen, som kan fremstå som forvirring, feber, hukommelsesproblemer eller krampeanfald (encephalitis)
- midlertidig inflammation i nerverne, der medfører smerter, svaghed og lammelse af arme og ben; en tilstand hvor musklerne bliver svage og hurtigere bliver trætte
- smerter, følelsesløshed, en snurrende fornemmelse eller svaghed i arme og ben; blære- eller tarmproblemer inklusive behov for hyppigere vandladning, ufrivillig vandladning, vandladningsbesvær og forstoppelse (myelitis (rygmarvsbetændelse))
- betændelse i den hinde, der omgiver rygmarven og hjernen, som kan vise sig som nakkestivhed, hovedpine, feber, lysfølsomhed i øjnene, kvalme eller opkastning (meningitis)
- inflammation i hjertemusklen, som kan vise sig som kortåndethed, uregelmæssig hjerterytme, træthed eller brystsmerter
- hul på tyndtarmen
- ømme, røde buler på huden
- kløe, blæredannelse i huden, afskalning af huden eller sårdannelse, og/eller læsioner i munden eller i slimhinderne i næsen, halsen eller i området omkring kønsorganerne (toksisk epidermal nekrolyse eller Stevens-Johnsons syndrom).

Følgende bivirkninger er indberettet i kliniske studier med pembrolizumab i kombination med kemoterapi:

Meget almindelig (kan forekomme hos flere end 1 ud af 10 personer)
- nedsat antal røde blodlegemer
- nedsat antal hvide blodlegemer; nedsat antal blodplader (øget tendens til at få blå mærker eller øget blødningstendens)
- nedsat indhold af kalium i blodet; nedsat appetit
- svimmelhed; hovedpine; inflammation i nerverne, som medfører følelsesløshed, svaghed, en snurrende fornemmelse eller brændende smerter i arme og ben; ændret smagssans
- kortåndethed; hoste
- diarré; kvalme; opkastning; forstoppelse; mavesmerter
- hududslæt; kløe; hårtab
- smerter i muskler og knogler; ledsmerter
- usædvanlig træthed eller svaghed; hævelser; feber
- unormale prøveresultater for nyrefunktion.

Almindelig (kan forekomme hos op til 1 ud af 10 personer)
- lungeinfektion (lungebetændelse)
- nedsat antal hvide blodlegemer (neutrofiler) med feber
- reaktion i forbindelse med infusion af medicinen
- problemer med skjoldbruskkirtlen
- nedsat indhold af natrium eller kalcium i blodet
- søvnbesvær
- tørre øjne
- unormal hjerterytme
- højt blodtryk

- inflammation i lungerne
- inflammation i tarmene; mundtørhed
- rødt hævet hududslæt, nogle gange med blæredannelse
- muskelsmerter eller muskelømhed; ledsmerter med hævelser; smerter i arme eller ben
- inflammation i nyrerne; pludseligt opstået nyreskade
- kuldegysninger; influenzalignende sygdom
- forhøjet indhold af kalcium i blodet; forhøjede leverenzymværdier i blodet.

Ikke almindelig (kan forekomme hos op til 1 ud af 100 personer)
- inflammation i hypofysen, som sidder i bunden af hjernen; inflammation i skjoldbruskkirtlen; nedsat udskillelse af hormoner produceret af binyrerne
- type 1-diabetes (sukkersyge)
- krampeanfald
- ophobning af væske omkring hjertet
- inflammation i bugspytkirtlen
- et sår i slimhinden i mavesækken eller i øvre del af tyndtarmen
- inflammation i leveren
- fortykkelse og nogle gange afskalning af huden; aknelignende hudproblemer; inflammation i huden; pletvise farveændringer i huden (hvide pletter)
- seneskedehindebetændelse
- forhøjet indhold af amylase, et enzym, som nedbryder stivelse; forhøjet indhold af bilirubin i blodet.

Sjælden (kan forekomme hos op til 1 ud af 1.000 personer)
- nedsat antal hvide blodlegemer (eosinofiler)
- inflammation i hjertemusklen, som kan vise sig som kortåndethed, uregelmæssig hjerterytme, træthed eller brystsmerter; betændelse i hjertesækken
- ændret hårfarve; tør, kløende hud; små buler eller sår på huden.

Følgende bivirkninger er indberettet i kliniske studier med pembrolizumab i kombination med axitinib:

Meget almindelig (kan forekomme hos flere end 1 ud af 10 personer)
- problemer med skjoldbruskkirtlen
- nedsat appetit
- hovedpine; ændret smagssans
- højt blodtryk
- kortåndethed; hoste; hæshed
- diarré; mavesmerter; kvalme; opkastning; forstoppelse
- blærer eller udslæt på håndflader eller fodsåler; hududslæt; kløe
- træthedsfølelse; usædvanlig træthed eller svaghed; feber
- forhøjede leverenzymværdier i blodet; unormale prøveresultater for nyrefunktion
- smerter i muskler og knogler; ledsmerter eller muskelsmerter, smerter eller ømhed; smerter i arme eller ben.

Almindelig (kan forekomme hos op til 1 ud af 10 personer)
- lungeinfektion (lungebetændelse)
- nedsat antal røde blodlegemer; nedsat antal hvide blodlegemer (neutrofiler, leukocytter); nedsat antal blodplader (øget tendens til at få blå mærker eller øget blødningstendens)
- reaktion i forbindelse med infusion af medicinen
- inflammation i hypofysen, som sidder i bunden af hjernen; inflammation i skjoldbruskkirtlen; nedsat udskillelse af hormoner produceret af binyrerne
- nedsat indhold af kalium, natrium eller kalcium i blodet
- søvnbesvær
- svimmelhed; manglende energi; inflammation i nerverne, som medfører følelsesløshed, svaghed og en snurrende fornemmelse eller brændende smerter i arme og ben
- tørre øjne
- unormal hjerterytme

- inflammation i lungerne
- inflammation i tarmene; mundtørhed
- rødt hævet hududslæt, nogle gange med blæredannelse; aknelignende hudproblemer; inflammation i huden; tør, kløende hud; hårtab
- inflammation i leveren
- muskelsmerter eller muskelømhed; ledsmerter eller muskelsmerter med hævelser; seneskedehindebetændelse
- pludseligt opstået nyreskade; inflammation i nyrerne
- hævelser; influenzalignende sygdom; kuldegysninger
- forhøjet indhold af kalcium i blodet; forhøjede leverenzymværdier i blodet.

Ikke almindelig (kan forekomme hos op til 1 ud af 100 personer)
- nedsat antal hvide blodlegemer (lymfocytter og eosinofiler)
- type 1-diabetes (sukkersyge)
- en tilstand hvor musklerne bliver svage og hurtigere bliver trætte
- inflammation i hjertemusklen, som kan inkludere kortåndethed, uregelmæssig hjerterytme, træthed eller brystsmerter
- inflammation i øjnene; øjensmerter, øjenirritation, kløe eller røde øjne; ubehagelig lysfølsomhed; pletter for øjnene
- inflammation i bugspytkirtlen
- et sår i slimhinden i mavesækken eller i øvre del af tyndtarmen
- ændret hårfarve; fortykkelse og nogle gange afskalning af huden; små buler eller sår på huden; pletvise farveændringer i huden (hvide pletter)
- forhøjet indhold af amylase, et enzym, som nedbryder stivelse.

Indberetning af bivirkninger
Hvis du oplever bivirkninger, bør du tale med din læge. Dette gælder også mulige bivirkninger, som ikke er medtaget i denne indlægsseddel. Du eller dine pårørende kan også indberette bivirkninger direkte til Lægemiddelstyrelsen via det nationale rapporteringssystem anført i Appendiks V. Ved at indrapportere bivirkninger kan du hjælpe med at fremskaffe mere information om sikkerheden af dette lægemiddel.

5. Opbevaring

Opbevar lægemidlet utilgængeligt for børn.

Brug ikke lægemidlet efter den udløbsdato, der står på kartonen eller etiketten på hætteglasset efter EXP. Udløbsdatoen er den sidste dag i den nævnte måned.

Opbevares i køleskab (2 °C - 8 °C).

Ud fra en mikrobiologisk synsvinkel skal det rekonstituerede koncentrat eller den fortyndede infusionsvæske anvendes straks. Den rekonstituerede eller fortyndede opløsning må ikke nedfryses. Hvis Keytruda ikke anvendes straks, er der påvist kemisk og fysisk stabilitet i 96 timer ved 2 °C-8 °C. Den samlede opbevaringstid på 96 timer efter rekonstitution kan inkludere opbevaring i op til 6 timer ved stuetemperatur (ved eller under 25 °C). Hætteglassene og/eller de intravenøse infusionsposer skal opnå stuetemperatur inden brug, hvis de har været opbevaret i køleskab.

Eventuelt resterende infusionsvæske må ikke gemmes til senere brug. Ikke anvendt lægemiddel samt affald heraf skal bortskaffes i henhold til lokale retningslinjer.

6. Pakningsstørrelser og yderligere oplysninger

Keytruda indeholder:
- Aktivt stof: pembrolizumab. Et hætteglas indeholder 50 mg pembrolizumab. Efter rekonstitution indeholder 1 ml koncentrat 25 mg pembrolizumab.

- Øvrige indholdsstoffer: L-histidin, L-histidin-hydrochloridmonohydrat, saccharose og polysorbat 80.

Udseende og pakningsstørrelser
Keytruda er et hvidt til offwhite frysetørret pulver.
Det fås i pakninger med et hætteglas.

Indehaver af markedsføringstilladelsen
Merck Sharp & Dohme B.V.
Waarderweg 39
2031 BN Haarlem
Holland

Fremstiller
Schering-Plough Labo NV
Industriepark 30
B-2220 Heist-op-den-Berg
Belgien

Hvis du ønsker yderligere oplysninger om dette lægemiddel, skal du henvende dig til den lokale repræsentant for indehaveren af markedsføringstilladelsen:

België/Belgique/Belgien
MSD Belgium BVBA/SPRL
Tél/Tel: +32(0)27766211
dpoc_belux@merck.com

България
Мерк Шарп и Доум България ЕООД
Тел.: +359 2 819 3737
info-msdbg@merck.com

Česká republika
Merck Sharp & Dohme s.r.o.
Tel: +420 233 010 111
dpoc_czechslovak@merck.com

Danmark
MSD Danmark ApS
Tlf: + 45 4482 4000
dkmail@merck.com

Deutschland
MSD SHARP & DOHME GMBH
Tel: 0800 673 673 673 (+49 (0) 89 4561 2612)
e-mail@msd.de

Eesti
Merck Sharp & Dohme OÜ
Tel.: +372 6144 200
msdeesti@merck.com

Lietuva
UAB Merck Sharp & Dohme
Tel. + 370 5 278 02 47
msd_lietuva@merck.com

Luxembourg/Luxemburg
MSD Belgium BVBA/SPRL
Tél/Tel: +32(0)27766211
dpoc_belux@merck.com

Magyarország
MSD Pharma Hungary Kft.
Tel.: +36 1 888 5300
hungary_msd@merck.com

Malta
Merck Sharp & Dohme Cyprus Limited
Tel: 8007 4433 (+356 99917558)
malta_info@merck.com

Nederland
Merck Sharp & Dohme B.V.
Tel: 0800 9999000
(+31 23 5153153)
medicalinfo.nl@merck.com

Norge
MSD (Norge) AS
Tlf: +47 32 20 73 00
msdnorge@msd.no

Ελλάδα
MSD A.Φ.B.E.E.
Τηλ: +30 210 98 97 300
dpoc_greece@merck.com

España
Merck Sharp & Dohme de España, S.A.
Tel: +34 91 321 06 00
msd_info@merck.com

France
MSD France
Tél: + 33 (0) 1 80 46 40 40

Hrvatska
Merck Sharp & Dohme d.o.o.
Tel: + 385 1 6611 333
croatia_info@merck.com

Ireland
Merck Sharp & Dohme Ireland (Human Health) Limited
Tel: +353 (0)1 2998700
medinfo_ireland@merck.com

Ísland
Vistor hf.
Sími: + 354 535 7000

Italia
MSD Italia S.r.l.
Tel: +39 06 361911
medicalinformation.it@merck.com

Κύπρος
Merck Sharp & Dohme Cyprus Limited
Τηλ.: 800 00 673 (+357 22866700)
cyprus_info@merck.com

Latvija
SIA Merck Sharp & Dohme Latvija
Tel: + 371 67364224
msd_lv@merck.com

Österreich
Merck Sharp & Dohme Ges.m.b.H.
Tel: +43 (0) 1 26 044
msd-medizin@merck.com

Polska
MSD Polska Sp. z o.o.
Tel: +48 22 549 51 00
msdpolska@merck.com

Portugal
Merck Sharp & Dohme, Lda
Tel: +351 21 4465700
inform_pt@merck.com

România
Merck Sharp & Dohme Romania S.R.L.
Tel: +40 21 529 29 00
msdromania@merck.com

Slovenija
Merck Sharp & Dohme, inovativna zdravila d.o.o.
Tel: +386 1 5204 201
msd.slovenia@merck.com

Slovenská republika
Merck Sharp & Dohme, s. r. o.
Tel: +421 2 58282010
dpoc_czechslovak@merck.com

Suomi/Finland
MSD Finland Oy
Puh/Tel: +358 (0)9 804 650
info@msd.fi

Sverige
Merck Sharp & Dohme (Sweden) AB
Tel: +46 77 5700488
medicinskinfo@merck.com

United Kingdom
Merck Sharp & Dohme Limited
Tel: +44 (0) 1992 467272
medicalinformationuk@merck.com

Denne indlægsseddel blev senest ændret

Andre informationskilder

Du kan finde yderligere oplysninger om dette lægemiddel på Det Europæiske Lægemiddelagenturs hjemmeside http://www.ema.europa.eu.

Nedenstående oplysninger er til læger og sundhedspersonale:

Klargøring og administration

- Før rekonstitution kan hætteglasset med frysetørret pulver (lyofilisat) opbevares uden for køleskab (ved temperaturer på eller under 25 °C) i op til 24 timer.
- Tilføj 2,3 ml vand til injektionsvæsker aseptisk for at få en opløsning af Keytruda på 25 mg/ml (pH: 5,2-5,8). Hvert hætteglas indeholder et overskud på 10 mg (0,4 ml) for at sikre, at der kan udtrækkes 50 mg Keytruda pr. hætteglas. Efter rekonstitution indeholder 1 ml koncentrat 25 mg pembrolizumab.
- Tilsæt vandet ned langs siden af hætteglasset og ikke direkte ned i det frysetørrede pulver for at undgå skumdannelse.
- Hvirvl langsomt hætteglasset rundt således, at det frysetørrede pulver bliver opløst. Lad der gå 5 minutter, så eventuelle bobler forsvinder. Hætteglasset må ikke rystes.
- Parenterale lægemidler skal inspiceres visuelt for partikler og misfarvning inden administration. Det rekonstituerede Keytruda-koncentrat er en klar til let opaliserende og farveløs til lysegul opløsning. Hætteglasset skal kasseres, hvis der observeres synlige partikler.
- Træk det påkrævede volumen (op til 2 ml (50 mg)) Keytruda op og overfør det til en intravenøs infusionspose, der indeholder natriumchlorid 9 mg/ml (0,9%) eller glucose 50 mg/ml (5%) for at fremstille den færdige infusionsvæske med en endelig koncentration på 1 til 10 mg/ml. Bland den færdige infusionsvæske ved forsigtigt at vende infusionsposen.
- Ud fra en mikrobiologisk synsvinkel skal det rekonstituerede koncentrat eller den fortyndede infusionsvæske anvendes straks. Den rekonstituerede eller fortyndede opløsning må ikke nedfryses. Hvis Keytruda ikke anvendes straks, er der påvist kemisk og fysisk stabilitet i 96 timer ved 2 °C-8 °C. Den samlede opbevaringstid på 96 timer efter rekonstitution kan inkludere opbevaring i op til 6 timer ved stuetemperatur (ved eller under 25 °C). Hætteglassene og/eller de intravenøse infusionsposer skal opnå stuetemperatur inden brug, hvis de har været opbevaret i køleskab. Gennemsigtige til hvide proteinholdige partikler kan forekomme i den fortyndede infusionsvæske. Administrer infusionsvæsken intravenøst over en periode på 30 minutter med et infusionssæt med et *in-line* eller *add-on* sterilt, ikke-pyrogent, minimalt proteinbindende filter (porestørrelse 0,2 til 5 µm).
- Keytruda må ikke administreres sammen med andre lægemidler i samme infusionsslange.
- Keytruda er kun til engangsbrug. Den ubrugte rest i hætteglasset bortskaffes.

Ikke anvendt lægemiddel samt affald heraf skal bortskaffes i henhold til lokale retningslinjer.

Indlægsseddel: Information til patienten

Keytruda 25 mg/ml koncentrat til infusionsvæske, opløsning
pembrolizumab

Læs denne indlægsseddel grundigt, inden du får dette lægemiddel, da den indeholder vigtige oplysninger.
- Gem indlægssedlen. Du kan få brug for at læse den igen.
- Det er vigtigt, at du har patientinformationskortet med dig under behandlingen.
- Spørg lægen, hvis der er mere, du vil vide.
- Kontakt lægen, hvis du får bivirkninger, herunder bivirkninger, som ikke er nævnt i denne indlægsseddel. Se punkt 4.

Se den nyeste indlægsseddel på www.indlaegsseddel.dk

Oversigt over indlægssedlen

1. Virkning og anvendelse
2. Det skal du vide, før du får Keytruda
3. Sådan får du Keytruda
4. Bivirkninger
5. Opbevaring
6. Pakningsstørrelser og yderligere oplysninger

1. Virkning og anvendelse

Keytruda indeholder det aktive stof pembrolizumab, som er et monoklonalt antistof. Keytruda styrker immunsystemets evne til at bekæmpe kræft.

Keytruda anvendes til voksne til at behandle:
- en bestemt type hudkræft kaldet melanom (modermærkekræft)
- en bestemt type lungekræft kaldet ikke-småcellet lungekræft
- en bestemt type lymfeknudekræft kaldet klassisk Hodgkins lymfom
- en bestemt type blærekræft kaldet urotelialt karcinom
- en bestemt type hoved-halskræft kaldet planocellulært hoved-hals karcinom
- en bestemt type nyrekræft kaldet renalcellekarcinom.

Keytruda gives til patienter, når deres kræft har spredt sig eller ikke kan fjernes ved en operation.

Keytruda gives til patienter, når de har fået foretaget en operation for at fjerne modermærkekræft for at forebygge, at deres kræft vender tilbage (adjuverende behandling).

Keytruda kan gives i kombination med anden kræftmedicin. Det er vigtigt, at du også læser indlægssedlerne for disse andre lægemidler. Spørg lægen, hvis du har eventuelle spørgsmål vedrørende disse lægemidler.

2. Det skal du vide, før du får Keytruda

Du må ikke få Keytruda
- hvis du er allergisk over for pembrolizumab eller et af de øvrige indholdsstoffer i Keytruda (angivet i punkt 6 "Pakningsstørrelser og yderligere oplysninger"). Er du i tvivl, så spørg lægen.

Advarsler og forsigtighedsregler
Kontakt lægen eller sygeplejersken, før du får Keytruda.

Fortæl det til lægen, før du får Keytruda:

- hvis du har en autoimmun sygdom (en tilstand, hvor kroppen angriber sine egne celler)
- hvis du har lungebetændelse eller inflammation (en betændelseslignende tilstand) i lungerne (pneumonitis)
- hvis du tidligere har fået ipilimumab, der er et andet lægemiddel til behandling af melanom, og har oplevet alvorlige bivirkninger på grund af dette lægemiddel
- hvis du har haft en allergisk reaktion ved behandling med andre monoklonale antistoffer
- hvis du har eller har haft en kronisk virusinfektion i leveren, herunder hepatitis B (hbv) eller hepatitis C (hcv)
- hvis du har hiv- (human immundefekt virus) infektion eller aids (erhvervet immundefekt syndrom)
- hvis du har leverskader
- hvis du har nyreskader
- hvis du har gennemgået en transplantation af et fast organ eller en knoglemarvstransplantation (stamcelletransplantation), hvor der anvendes stamceller fra en donor (allogen transplantation).

Når du får Keytruda, kan du få nogle alvorlige bivirkninger. Disse bivirkninger kan nogle gange blive livstruende og kan medføre døden. Disse bivirkninger kan forekomme når som helst under behandlingen, eller selv efter afslutning af din behandling. Du kan få mere end en bivirkning på samme tid.

Kontakt straks lægen, hvis du får en eller flere af følgende tilstande. Din læge vil muligvis give dig anden medicin for at forebygge mere alvorlige komplikationer og mildne dine symptomer. Din læge vil muligvis udsætte den næste dosis af Keytruda eller helt stoppe din behandling med Keytruda.

- inflammation (en betændelseslignende tilstand) i lungerne, som kan inkludere kortåndethed, brystsmerter eller hoste
- betændelse i tarmene, som kan inkludere diarré eller flere afføringer end normalt, sort, tjæreagtig, klistret afføring, blod eller slim i afføringen, svære mavesmerter eller ømhed i maveområdet, kvalme, opkastning
- leverbetændelse, som kan inkludere kvalme eller opkastning, nedsat appetit, smerter i højre side af maven, gulfarvning af huden eller det hvide i øjnene, mørk urin, øget blødningstendens eller tendens til lettere at få blå mærker
- inflammation i nyrerne, som kan inkludere ændringer i mængden eller farven af urinen
- inflammation i hormonproducerende kirtler (især skjoldbruskkirtlen, hypofysen og binyrerne), som kan inkludere hurtig hjerterytme (puls), vægttab, øget svedtendens, vægtstigning, hårtab, kuldefornemmelse, forstoppelse, dybere stemme, muskelsmerter, svimmelhed eller besvimelse, hovedpine, som ikke vil forsvinde, eller usædvanlig hovedpine
- type 1-diabetes (sukkersyge), som kan inkludere øget appetit eller tørst, hyppigere vandladning eller vægttab
- inflammation i øjnene, som kan inkludere synsændringer
- inflammation i musklerne, som kan inkludere muskelsmerter eller muskelsvaghed
- inflammation i hjertemusklen, som kan inkludere kortåndethed, uregelmæssig hjerterytme, træthed eller brystsmerter
- inflammation i bugspytkirtlen, som kan inkludere mavesmerter, kvalme og opkastning
- inflammation i huden, som kan inkludere udslæt, kløe, blæredannelse i huden, afskalning af huden eller sårdannelse, og/eller læsioner i munden eller i slimhinderne i næsen, halsen eller i området omkring kønsorganerne
- en sygdom i immunsystemet, der kan ramme lungerne, huden, øjnene og/eller lymfeknuderne (sarkoidose)
- inflammation i hjernen, som kan inkludere forvirring, feber, hukommelsesproblemer eller krampeanfald (encephalitis)
- smerter, følelsesløshed, en snurrende fornemmelse eller svaghed i arme og ben; blære- eller tarmproblemer inklusive behov for hyppigere vandladning, ufrivillig vandladning, vandladningsbesvær og forstoppelse (myelitis (rygmarvsbetændelse))
- infusionsreaktioner, som kan inkludere kortåndethed, kløe eller udslæt, svimmelhed eller feber.

Komplikationer, inklusive *graft versus host*-sygdom (GVHD), hos personer der gennemgår knoglemarvstransplantation (stamcelletransplantation), hvor der anvendes stamceller fra en donor (allogen transplantation). Disse komplikationer kan være alvorlige og kan medføre døden. De kan

forekomme, hvis du tidligere har gennemgået denne form for transplantation, eller hvis du skal have en sådan transplantation engang i fremtiden. Lægen vil overvåge dig for tegn og symptomer, som kan inkludere hududslæt, leverbetændelse, mavesmerter eller diarré.

Børn og unge
Keytruda må ikke anvendes til børn og unge under 18 år.

Brug af anden medicin sammen med Keytruda
Fortæl det altid til lægen,
- hvis du tager anden medicin, som svækker dit immunsystem. Eksempler herpå kan være kortikosteroider, som f.eks. prednison. Disse typer medicin kan påvirke virkningen af Keytruda. Når du er i behandling med Keytruda, kan lægen imidlertid godt give dig kortikosteroider for at mildne de bivirkninger, du kan få af Keytruda. Du kan også få kortikosteroider, før du får Keytruda i kombination med kemoterapi for at forebygge og/eller behandle kvalme, opkastning og andre bivirkninger forårsaget af kemoterapi.
- hvis du tager anden medicin, for nylig har taget anden medicin eller planlægger at tage anden medicin.

Graviditet
- Du må ikke få Keytruda, hvis du er gravid, medmindre lægen specifikt anbefaler det.
- Fortæl det til lægen, hvis du er gravid, har mistanke om, at du er gravid, eller planlægger at blive gravid.
- Keytruda kan skade det ufødte barn eller øge risikoen for, at det dør.
- Hvis du er en kvinde i den fødedygtige alder, skal du anvende sikker prævention, mens du bliver behandlet med Keytruda og i mindst 4 måneder efter din sidste dosis.

Amning
- Fortæl det til lægen, hvis du ammer.
- Du må ikke amme, mens du får Keytruda.
- Det er ukendt, om Keytruda udskilles i mælken hos mennesker.

Trafik- og arbejdssikkerhed
Keytruda påvirker i mindre grad evnen til at føre motorkøretøj og betjene maskiner. Svimmelhed, træthed eller svaghedsfølelse er mulige bivirkninger ved Keytruda. Undlad at føre motorkøretøj og betjene maskiner, hvis du har fået Keytruda, medmindre du er sikker på, at du har det godt.

3. Sådan får du Keytruda

Du får Keytruda på et hospital under tilsyn af en læge med erfaring i behandling af kræft.
- Den anbefalede dosis af Keytruda er enten 200 mg hver 3. uge eller 400 mg hver 6. uge.
- Lægen vil give dig Keytruda som en infusion i en vene (intravenøst) over en periode på ca. 30 minutter.
- Lægen beslutter, hvor mange behandlinger du har brug for.

Hvis du glemmer en aftale om at få behandling med Keytruda
- Kontakt straks lægen for at lave en ny aftale.
- Det er meget vigtigt, at du ikke springer en infusion med dette lægemiddel over.

Hvis du holder op med at få Keytruda
Hvis du stopper behandlingen, kan virkningen af medicinen ophøre. Du må ikke stoppe behandlingen med Keytruda, medmindre du har aftalt det med lægen.
Spørg lægen, hvis der er noget, du er i tvivl om.

Du kan også finde disse oplysninger på det patientinformationskort, du har fået af din læge. Det er vigtigt, at du gemmer dette patientinformationskort og viser det til din partner eller dine omsorgspersoner.

4. Bivirkninger

Dette lægemiddel kan som alle andre lægemidler give bivirkninger, men ikke alle får bivirkninger.

Når du får Keytruda, kan du få nogle alvorlige bivirkninger. Se punkt 2.

Følgende bivirkninger er indberettet med pembrolizumab alene:

Meget almindelig (kan forekomme hos flere end 1 ud af 10 personer)
- nedsat antal røde blodlegemer
- nedsat funktion af skjoldbruskkirtlen
- nedsat appetit
- hovedpine
- kortåndethed; hoste
- diarré; mavesmerter; kvalme; opkastning; forstoppelse
- kløe; hududslæt
- smerter i muskler og knogler; ledsmerter
- træthedsfølelse; usædvanlig træthed eller svaghed; hævelser; feber.

Almindelig (kan forekomme hos op til 1 ud af 10 personer)
- lungeinfektion (lungebetændelse)
- nedsat antal blodplader (øget tendens til at få blå mærker eller øget blødningstendens); nedsat antal hvide blodlegemer (lymfocytter)
- reaktion i forbindelse med infusion af medicinen
- overaktiv skjoldbruskkirtel; hedeture
- nedsat indhold af natrium, kalium eller kalcium i blodet
- søvnbesvær
- svimmelhed; inflammation i nerverne, som medfører følelsesløshed, svaghed og en snurrende fornemmelse eller brændende smerter i arme og ben; manglende energi; ændret smagssans
- tørre øjne
- unormal hjerterytme
- højt blodtryk
- inflammation i lungerne
- inflammation i tarmene; mundtørhed
- rødt hævet hududslæt, nogle gange med blæredannelse; pletvise farveændringer i huden (hvide pletter); tør, kløende hud; hårtab; aknelignende hudproblemer
- muskelsmerter eller muskelømhed; smerter i arme eller ben; ledsmerter med hævelser
- kuldegysninger; influenzalignende sygdom
- forhøjede leverenzymværdier i blodet; forhøjet indhold af kalcium i blodet; unormale prøveresultater for nyrefunktion.

Ikke almindelig (kan forekomme hos op til 1 ud af 100 personer)
- nedsat antal hvide blodlegemer (neutrofiler, leukocytter og eosinofiler)
- en sygdom i immunsystemet, der kan ramme lungerne, huden, øjnene og/eller lymfeknuderne (sarkoidose)
- inflammation i hypofysen, som sidder i bunden af hjernen; nedsat udskillelse af hormoner produceret af binyrerne; inflammation i skjoldbruskkirtlen
- type 1-diabetes (sukkersyge)
- krampeanfald
- inflammation i øjnene; øjensmerter, øjenirritation, kløe eller røde øjne; ubehagelig lysfølsomhed; pletter for øjnene
- betændelse i hjertesækken; ophobning af væske omkring hjertet
- inflammation i bugspytkirtlen
- et sår i slimhinden i mavesækken eller i øvre del af tyndtarmen
- inflammation i leveren

- fortykkelse og nogle gange afskalning af huden; inflammation i huden; ændret hårfarve; små buler eller sår på huden
- seneskedehindebetændelse
- inflammation i nyrerne
- forhøjet indhold af amylase, et enzym, som nedbryder stivelse.

Sjælden (kan forekomme hos op til 1 ud af 1.000 personer)
- betændelsesreaktion (inflammatorisk respons) på blodplader eller røde blodlegemer; følelse af svaghed, svimmelhed, kortåndethed, eller hvis du har bleg hud (tegn på et nedsat antal røde blodlegemer, der muligvis skyldes en type blodmangel (anæmi) kaldet ren erytrocyt aplasi); en tilstand kaldet hæmofagocytisk lymfohistiocytose, hvor immunsystemet danner for mange infektionsbekæmpende celler kaldet histiocytter og lymfocytter, som kan give forskellige symptomer
- inflammation i hjernen, som kan fremstå som forvirring, feber, hukommelsesproblemer eller krampeanfald (encephalitis)
- midlertidig inflammation i nerverne, der medfører smerter, svaghed og lammelse af arme og ben; en tilstand hvor musklerne bliver svage og hurtigere bliver trætte
- smerter, følelsesløshed, en snurrende fornemmelse eller svaghed i arme og ben; blære- eller tarmproblemer inklusive behov for hyppigere vandladning, ufrivillig vandladning, vandladningsbesvær og forstoppelse (myelitis (rygmarvsbetændelse))
- betændelse i den hinde, der omgiver rygmarven og hjernen, som kan vise sig som nakkestivhed, hovedpine, feber, lysfølsomhed i øjnene, kvalme eller opkastning (meningitis)
- inflammation i hjertemusklen, som kan vise sig som kortåndethed, uregelmæssig hjerterytme, træthed eller brystsmerter
- hul på tyndtarmen
- ømme, røde buler på huden
- kløe, blæredannelse i huden, afskalning af huden eller sårdannelse, og/eller læsioner i munden eller i slimhinderne i næsen, halsen eller i området omkring kønsorganerne (toksisk epidermal nekrolyse eller Stevens-Johnsons syndrom).

Følgende bivirkninger er indberettet i kliniske studier med pembrolizumab i kombination med kemoterapi:

Meget almindelig (kan forekomme hos flere end 1 ud af 10 personer)
- nedsat antal røde blodlegemer
- nedsat antal hvide blodlegemer; nedsat antal blodplader (øget tendens til at få blå mærker eller øget blødningstendens)
- nedsat indhold af kalium i blodet; nedsat appetit
- svimmelhed; hovedpine; inflammation i nerverne, som medfører følelsesløshed, svaghed, en snurrende fornemmelse eller brændende smerter i arme og ben; ændret smagssans
- kortåndethed; hoste
- diarré; kvalme; opkastning; forstoppelse; mavesmerter
- hududslæt; kløe; hårtab
- smerter i muskler og knogler; ledsmerter
- usædvanlig træthed eller svaghed; hævelser; feber
- unormale prøveresultater for nyrefunktion.

Almindelig (kan forekomme hos op til 1 ud af 10 personer)
- lungeinfektion (lungebetændelse)
- nedsat antal hvide blodlegemer (neutrofiler) med feber
- reaktion i forbindelse med infusion af medicinen
- problemer med skjoldbruskkirtlen
- nedsat indhold af natrium eller kalcium i blodet
- søvnbesvær
- tørre øjne
- unormal hjerterytme
- højt blodtryk

- inflammation i lungerne
- inflammation i tarmene; mundtørhed
- rødt hævet hududslæt, nogle gange med blæredannelse
- muskelsmerter eller muskelømhed; ledsmerter med hævelser; smerter i arme eller ben
- inflammation i nyrerne; pludseligt opstået nyreskade
- kuldegysninger; influenzalignende sygdom
- forhøjet indhold af kalcium i blodet; forhøjede leverenzymværdier i blodet.

Ikke almindelig (kan forekomme hos op til 1 ud af 100 personer)
- inflammation i hypofysen, som sidder i bunden af hjernen; inflammation i skjoldbruskkirtlen; nedsat udskillelse af hormoner produceret af binyrerne
- type 1-diabetes (sukkersyge)
- krampeanfald
- ophobning af væske omkring hjertet
- inflammation i bugspytkirtlen
- et sår i slimhinden i mavesækken eller i øvre del af tyndtarmen
- inflammation i leveren
- fortykkelse og nogle gange afskalning af huden; aknelignende hudproblemer; inflammation i huden; pletvise farveændringer i huden (hvide pletter)
- seneskedehindebetændelse
- forhøjet indhold af amylase, et enzym, som nedbryder stivelse; forhøjet indhold af bilirubin i blodet.

Sjælden (kan forekomme hos op til 1 ud af 1.000 personer)
- nedsat antal hvide blodlegemer (eosinofiler)
- inflammation i hjertemusklen, som kan vise sig som kortåndethed, uregelmæssig hjerterytme, træthed eller brystsmerter; betændelse i hjertesækken
- ændret hårfarve; tør, kløende hud; små buler eller sår på huden.

Følgende bivirkninger er indberettet i kliniske studier med pembrolizumab i kombination med axitinib:

Meget almindelig (kan forekomme hos flere end 1 ud af 10 personer)
- problemer med skjoldbruskkirtlen
- nedsat appetit
- hovedpine; ændret smagssans
- højt blodtryk
- kortåndethed; hoste; hæshed
- diarré; mavesmerter; kvalme; opkastning; forstoppelse
- blærer eller udslæt på håndflader eller fodsåler; hududslæt; kløe
- træthedsfølelse; usædvanlig træthed eller svaghed; feber
- forhøjede leverenzymværdier i blodet; unormale prøveresultater for nyrefunktion
- smerter i muskler og knogler; ledsmerter eller muskelsmerter, smerter eller ømhed; smerter i arme eller ben.

Almindelig (kan forekomme hos op til 1 ud af 10 personer)
- lungeinfektion (lungebetændelse)
- nedsat antal røde blodlegemer; nedsat antal hvide blodlegemer (neutrofiler, leukocytter); nedsat antal blodplader (øget tendens til at få blå mærker eller øget blødningstendens)
- reaktion i forbindelse med infusion af medicinen
- inflammation i hypofysen, som sidder i bunden af hjernen; inflammation i skjoldbruskkirtlen; nedsat udskillelse af hormoner produceret af binyrerne
- nedsat indhold af kalium, natrium eller kalcium i blodet
- søvnbesvær
- svimmelhed; manglende energi; inflammation i nerverne, som medfører følelsesløshed, svaghed og en snurrende fornemmelse eller brændende smerter i arme og ben
- tørre øjne
- unormal hjerterytme

- inflammation i lungerne
- inflammation i tarmene; mundtørhed
- rødt hævet hududslæt, nogle gange med blæredannelse; aknelignende hudproblemer; inflammation i huden; tør, kløende hud; hårtab
- inflammation i leveren
- muskelsmerter eller muskelømhed; ledsmerter eller muskelsmerter med hævelser; seneskedehindebetændelse
- pludseligt opstået nyreskade; inflammation i nyrerne
- hævelser; influenzalignende sygdom; kuldegysninger
- forhøjet indhold af kalcium i blodet; forhøjede leverenzymværdier i blodet.

Ikke almindelig (kan forekomme hos op til 1 ud af 100 personer)
- nedsat antal hvide blodlegemer (lymfocytter og eosinofiler)
- type 1-diabetes (sukkersyge)
- en tilstand hvor musklerne bliver svage og hurtigere bliver trætte
- inflammation i hjertemusklen, som kan inkludere kortåndethed, uregelmæssig hjerterytme, træthed eller brystsmerter
- inflammation i øjnene; øjensmerter, øjenirritation, kløe eller røde øjne; ubehagelig lysfølsomhed; pletter for øjnene
- inflammation i bugspytkirtlen
- et sår i slimhinden i mavesækken eller i øvre del af tyndtarmen
- ændret hårfarve; fortykkelse og nogle gange afskalning af huden; små buler eller sår på huden; pletvise farveændringer i huden (hvide pletter)
- forhøjet indhold af amylase, et enzym, som nedbryder stivelse.

Indberetning af bivirkninger
Hvis du oplever bivirkninger, bør du tale med din læge. Dette gælder også mulige bivirkninger, som ikke er medtaget i denne indlægsseddel. Du eller dine pårørende kan også indberette bivirkninger direkte til Lægemiddelstyrelsen via det nationale rapporteringssystem anført i Appendiks V. Ved at indrapportere bivirkninger kan du hjælpe med at fremskaffe mere information om sikkerheden af dette lægemiddel.

5. Opbevaring

Opbevar lægemidlet utilgængeligt for børn.

Brug ikke lægemidlet efter den udløbsdato, der står på kartonen eller etiketten på hætteglasset efter EXP. Udløbsdatoen er den sidste dag i den nævnte måned.

Opbevares i køleskab (2 °C - 8 °C).
Må ikke nedfryses.
Opbevares i den originale karton for at beskytte mod lys.

Ud fra en mikrobiologisk synsvinkel skal lægemidlet anvendes straks efter fortynding. Den fortyndede infusionsvæske må ikke nedfryses. Hvis Keytruda ikke anvendes straks, er der påvist kemisk og fysisk stabilitet i 96 timer ved 2 °C-8 °C. Disse 96 timer kan inkludere opbevaring i op til 6 timer ved stuetemperatur (ved eller under 25 °C). Hætteglassene og/eller de intravenøse infusionsposer skal opnå stuetemperatur inden brug, hvis de har været opbevaret i køleskab.

Eventuelt resterende infusionsvæske må ikke gemmes til senere brug. Ikke anvendt lægemiddel samt affald heraf skal bortskaffes i henhold til lokale retningslinjer.

6. Pakningsstørrelser og yderligere oplysninger

Keytruda indeholder:

- Aktivt stof: pembrolizumab. Et hætteglas med 4 ml indeholder 100 mg pembrolizumab. Hver ml koncentrat indeholder 25 mg pembrolizumab.
- Øvrige indholdsstoffer: L-histidin, L-histidin-hydrochloridmonohydrat, saccharose, polysorbat 80 og vand til injektionsvæsker.

Udseende og pakningsstørrelser
Keytruda er en klar til let opaliserende, farveløs til lysegul opløsning med pH 5,2 – 5,8.
Det fås i pakninger med et hætteglas.

Indehaver af markedsføringstilladelsen
Merck Sharp & Dohme B.V.
Waarderweg 39
2031 BN Haarlem
Holland

Fremstiller
Schering-Plough Labo NV
Industriepark 30
B-2220 Heist-op-den-Berg
Belgien

Hvis du ønsker yderligere oplysninger om dette lægemiddel, skal du henvende dig til den lokale repræsentant for indehaveren af markedsføringstilladelsen:

België/Belgique/Belgien
MSD Belgium BVBA/SPRL
Tél/Tel: +32(0)27766211
dpoc_belux@merck.com

България
Мерк Шарп и Доум България ЕООД
Тел.: +359 2 819 3737
info-msdbg@merck.com

Česká republika
Merck Sharp & Dohme s.r.o.
Tel: +420 233 010 111
dpoc_czechslovak@merck.com

Danmark
MSD Danmark ApS
Tlf: + 45 4482 4000
dkmail@merck.com

Deutschland
MSD SHARP & DOHME GMBH
Tel: 0800 673 673 673 (+49 (0) 89 4561 2612)
e-mail@msd.de

Lietuva
UAB Merck Sharp & Dohme
Tel. + 370 5 278 02 47
msd_lietuva@merck.com

Luxembourg/Luxemburg
MSD Belgium BVBA/SPRL
Tél/Tel: +32(0)27766211
dpoc_belux@merck.com

Magyarország
MSD Pharma Hungary Kft.
Tel.: +36 1 888 5300
hungary_msd@merck.com

Malta
Merck Sharp & Dohme Cyprus Limited
Tel: 8007 4433 (+356 99917558)
malta_info@merck.com

Nederland
Merck Sharp & Dohme B.V.
Tel: 0800 9999000
(+31 23 5153153)
medicalinfo.nl@merck.com

Eesti
Merck Sharp & Dohme OÜ
Tel.: +372 6144 200
msdeesti@merck.com

Ελλάδα
MSD A.Φ.B.E.E.
Τηλ: +30 210 98 97 300
dpoc_greece@merck.com

España
Merck Sharp & Dohme de España, S.A.
Tel: +34 91 321 06 00
msd_info@merck.com

France
MSD France
Tél: + 33 (0) 1 80 46 40 40

Hrvatska
Merck Sharp & Dohme d.o.o.
Tel: + 385 1 6611 333
croatia_info@merck.com

Ireland
Merck Sharp & Dohme Ireland (Human Health) Limited
Tel: +353 (0)1 2998700
medinfo_ireland@merck.com

Ísland
Vistor hf.
Sími: + 354 535 7000

Italia
MSD Italia S.r.l.
Tel: +39 06 361911
medicalinformation.it@merck.com

Κύπρος
Merck Sharp & Dohme Cyprus Limited
Τηλ.: 800 00 673 (+357 22866700)
cyprus_info@merck.com

Latvija
SIA Merck Sharp & Dohme Latvija
Tel: + 371 67364224
msd_lv@merck.com

Norge
MSD (Norge) AS
Tlf: +47 32 20 73 00
msdnorge@msd.no

Österreich
Merck Sharp & Dohme Ges.m.b.H.
Tel: +43 (0) 1 26 044
msd-medizin@merck.com

Polska
MSD Polska Sp. z o.o.
Tel: +48 22 549 51 00
msdpolska@merck.com

Portugal
Merck Sharp & Dohme, Lda
Tel: +351 21 4465700
inform_pt@merck.com

România
Merck Sharp & Dohme Romania S.R.L.
Tel: +40 21 529 29 00
msdromania@merck.com

Slovenija
Merck Sharp & Dohme, inovativna zdravila d.o.o.
Tel: +386 1 5204 201
msd.slovenia@merck.com

Slovenská republika
Merck Sharp & Dohme, s. r. o.
Tel: +421 2 58282010
dpoc_czechslovak@merck.com

Suomi/Finland
MSD Finland Oy
Puh/Tel: +358 (0)9 804 650
info@msd.fi

Sverige
Merck Sharp & Dohme (Sweden) AB
Tel: +46 77 5700488
medicinskinfo@merck.com

United Kingdom
Merck Sharp & Dohme Limited
Tel: +44 (0) 1992 467272
medicalinformationuk@merck.com

Denne indlægsseddel blev senest ændret

Andre informationskilder

Du kan finde yderligere oplysninger om dette lægemiddel på Det Europæiske Lægemiddelagenturs hjemmeside http://www.ema.europa.eu.

Nedenstående oplysninger er til læger og sundhedspersonale:

Klargøring og administration af infusionsvæsken

- Hætteglasset må ikke rystes.
- Lad hætteglasset opnå stuetemperatur (ved temperaturer på eller under 25 °C).
- Før fortynding kan hætteglasset med koncentrat opbevares uden for køleskab (ved temperaturer på eller under 25 °C) i op til 24 timer.
- Parenterale lægemidler skal inspiceres visuelt for partikler og misfarvning inden administration. Keytruda-koncentratet er en klar til let opaliserende og farveløs til lysegul opløsning. Hætteglasset skal kasseres, hvis der observeres synlige partikler.
- Træk det påkrævede volumen (op til 4 ml (100 mg)) koncentrat op og overfør det til en intravenøs infusionspose, der indeholder natriumchlorid 9 mg/ml (0,9%) eller glucose 50 mg/ml (5%) for at fremstille den færdige infusionsvæske med en endelig koncentration på 1 til 10 mg/ml. Hvert hætteglas indeholder et overskud på 0,25 ml (samlet indhold pr. hætteglas 4,25 ml) for at sikre, at der kan udtrækkes 4 ml koncentrat. Bland den færdige infusionsvæske ved forsigtigt at vende infusionsposen.
- Ud fra en mikrobiologisk synsvinkel skal lægemidlet anvendes straks efter fortynding. Den fortyndede infusionsvæske må ikke nedfryses. Hvis Keytruda ikke anvendes straks, er der påvist kemisk og fysisk stabilitet i 96 timer ved 2 °C-8 °C. Disse 96 timer kan inkludere opbevaring i op til 6 timer ved stuetemperatur (ved eller under 25 °C). Hætteglassene og/eller de intravenøse infusionsposer skal opnå stuetemperatur inden brug, hvis de har været opbevaret i køleskab. Gennemsigtige til hvide proteinholdige partikler kan forekomme i den fortyndede infusionsvæske. Administrer infusionsvæsken intravenøst over en periode på 30 minutter med et infusionssæt med et *in-line* eller *add-on* sterilt, ikke-pyrogent, minimalt proteinbindende filter (porestørrelse 0,2 til 5 µm).
- Keytruda må ikke administreres sammen med andre lægemidler i samme infusionsslange.
- Keytruda er kun til engangsbrug. Den ubrugte rest i hætteglasset bortskaffes.

Ikke anvendt lægemiddel samt affald heraf skal bortskaffes i henhold til lokale retningslinjer.

BILAG IV

VIDENSKABELIGE KONKLUSIONER OG BEGRUNDELSER FOR ÆNDRING AF BETINGELSERNE FOR MARKEDSFØRINGSTILLADELSEN/-TILLADELSERNE

Videnskabelige konklusioner

Under hensyntagen til PRAC's vurderingsrapport om PSUR'en/PSUR'erne for pembrolizumab er CHMP nået frem til følgende videnskabelige konklusioner:

Indehaveren af markedsføringstilladelsen har identificeret 7 tilfælde af glomerulonefritis, hvoraf 4 blev bekræftet ved biopsi sammen med en bedring af bivirkningen efter behandling med steroider, hvilket tyder på, at virkningsmekanismen var en immunrelateret bivirkning. Derfor indføres termen "glomerulonefritis" i fodnoterne til bivirkningstabellen i pkt. 4.8 i produktresuméet under beskrivelsen af termen "nefritis".

En gennemgang af risikoen for gastrisk ulceration viste et relativt højt antal indberettede tilfælde. Af de 15 tilfælde med ulcus ventriculi og ulcus duodeni, som blev gennemgået, blev der i flere tilfælde set en bedring efter behandling med steroider, eller bivirkningen blev indberettet som værende relateret til behandlingen, hvilket indikerer, at pembrolizumab spiller en rolle i at forårsage ulceration. Ulceration er en kendt risiko, der er anført i produktresuméet for andre klasseprodukter. Perforation af tyndtarmen og potentiel risiko for gastrointestinal perforation er allerede anført i produktresuméet for pembrolizumab i hhv. pkt. 4.8 og 4.4, og det anses for at være sandsynligt, at perforation indtræder efter ulceration. Sammenlagt anses en årsagssammenhæng mellem pembrolizumab og gastrointestinal ulceration for at være sandsynlig. Termen "gastrointestinal ulceration" inkluderes i pkt. 4.8 i produktresuméet med hyppigheden "ikke almindelig".

Af de 24 tilfælde med myelitis identificeret i indehaveren af markedsføringstilladelsens sikkerhedsdatabase forekommer 4 tilfælde (3 tilfælde indberettet som PT [preferred term (foretrukken term)] "transversel myelitis" og et tilfælde som "myelitis") med positiv *dechallenge* af pembrolizumab og/eller bedring efter behandling med steroider, hvilket understøtter en årsagssammenhæng mellem myelitis og anvendelse af pembrolizumab. Termen "myelitis" inkluderes i bivirkningstabellen i pkt. 4.8 i produktresuméet med hyppigheden "sjælden" baseret på CT-data for pembrolizumab som monoterapi (2 tilfælde (1 tilfælde med myelitis og 1 tilfælde med transversel myelitis) hos 5.884 behandlede patienter (0,03%)). Termen "transversel myelitis" tilføjes også i fodnoterne til bivirkningstabellen forbundet med risikoen for myelitis. Derudover inkluderes termen "myelitis" i pkt. 4.4 "Særlige advarsler og forsigtighedsregler vedrørende brugen" i produktresuméet i underafsnittet "Andre immunrelaterede bivirkninger".

CHMP tilslutter sig PRAC's videnskabelige konklusioner.

Begrundelser for ændring af betingelserne for markedsføringstilladelsen/-tilladelserne

På baggrund af de videnskabelige konklusioner for pembrolizumab er CHMP af den opfattelse, at benefit/risk-forholdet for det lægemiddel/de lægemidler, der indeholder pembrolizumab, forbliver uændret under forudsætning af, at de foreslåede ændringer indføres i produktinformationen.

CHMP anbefaler, at betingelserne for markedsføringstilladelsen/-tilladelserne ændres.

APPENDIX C: ADVERSE EVENT REGISTRATION AND EXEMPTIONS

Recording of Adverse Events

All AEs will be documented in the appropriate section of the electronic case report form (CRF) and a note will be made in the event list of the trial master file (TMF). The following aspects will be recorded for each event in the CRF:

- A description of the AE in medical terms, not as reported by the subject
- The date of onset
- The date of recovery
- Characterization of duration (transient vs permanent/chronic)
- AE grade (more information below)
- AE causality estimation (more information below)

At each contact with the subject, the investigator will seek information on adverse events by specific questioning and, as appropriate, by physical examination.

The clinical course of each event will be followed until resolution, stabilization or until it has been determined that study treatment or participation is not the cause. SAEs which are still on-going at the end of the active treatment period will be followed up to determine the final outcome.

The grade of the AE is assessed by the investigator according to the ICH-GCP grading system if the AE is possibly related to the immunotherapy treatment. All SAEs are reported to the sponsor within the time limits described by ICH-GCP. All adverse event possibly resulting from immunotherapy or the IRE procedure will be registered in the electronic CRF according to the definitions in NCI-CTC, version 5.0 grade 1-5:

- Grade 1 = mild
- Grade 2 = moderate
- Grade 3 = severe
- Grade 4 = life-threatening or disabling
- Grade 5 = death related to AE

All possibly IRE-related adverse events of grade 3 or above will be reported to the sponsor as “events of special interest”.

The causal relationship to immunotherapy is assessed by the sponsor in conjunction with the principal investigator. The sponsor does not have the authority to downgrade the seriousness of the event. Both assessments will be included in the report, if the causal relationship or the seriousness of the event is disputed between the sponsor and principal investigator.

The decisive factor in the documentation is the temporal relation between the AE and the intervention. The following judgments of the causality to the interventions are to be used:

- Not Related: There is not a temporal relationship to study drug administration (too early, too late, or study drug not taken), or there is a reasonable causal relationship between another drug, concurrent disease, or circumstance and the AE.
- Not likely: There is a temporal relationship to study drug administration, but there is not a reasonable causal relationship between the study drug and the AE.

- Possible: There is a reasonable causal relationship between the study drug and the AE. Dechallenge information (information referring to withdrawal of drug) is lacking or unclear.
- Probable: There is a reasonable causal relationship between the study drug and the AE. The event responds to dechallenge (withdrawal of study drug). Rechallenge is not required.
- Certain/Definite: There is a reasonable causal relationship between the study drug and the AE. The event responds to dechallenge and recurs with rechallenge, when clinically feasible.

Exemptions from reporting as serious adverse events

Progression of disease is not to be regarded as a SAE. Due to the seriousness of the disease in this study, certain conditions defined, as SAEs will be excluded from expedited reporting on a SAE report form:

- Elective hospitalization and surgery for treatment of disease e.g. palliative surgery or endoscopy

The following will not be considered serious for this study: An event that results in hospitalization or prolongs an existing hospitalization if the only reason for the hospitalization or prolongation is for the following:

- Hospitalization is secondary to expected immunotherapy morbidity e.g.
    1. Anemia, lymphopenia and/or thrombocytopenia
    2. Hypo- og hyperthyroidism
    3. Dyspnea and/or cough
    4. Diarrhea
    5. Obstipation
    6. Rash
    7. Muscle, joint or bone pain
    8. Oedema
    9. Fever
    10. Electrolyte disturbances
    11. Elevated liver enzymes

- Hospitalization is secondary to expected cancer morbidity e.g.
    1. Weight loss
    2. Fatigue
    3. Electrolyte disturbances
    4. Pain management
    5. Anxiety
    6. DVT or pulmonary embolism
    7. Admission for palliative care
    8. Admission for immunotherapy, chemotherapy or radiotherapy
    9. Transfusion of blood product

10. Administration of study procedure
11. Placement of permanent intravenous catheter
12. Hospice placement for terminal care

These events will be recorded on the data collection form. Any patient's death must be recorded on the data collection form.

An algorithm of the SAE- reporting process is available below:

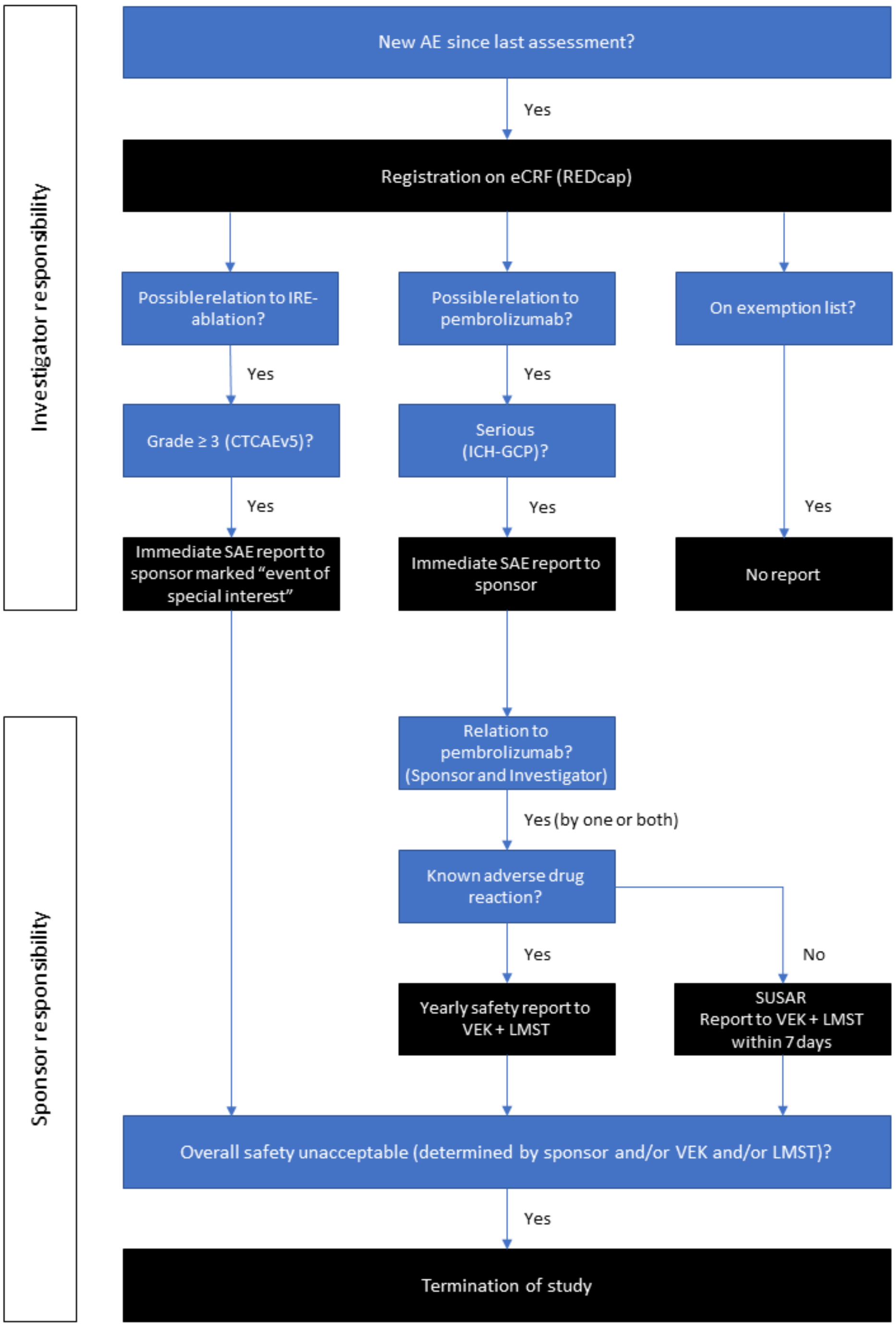

APPENDIX D: VURDERING OG HÅNDTERING AF BIVIRKNINGER RELATERET TIL IMMUNTERAPI MED CHECK-POINT HÆMMERE (ANTI-CTLA-4, ANTI PD-1 OG ANTI-PD-L1)

Vurdering og håndtering af bivirkninger relateret til immunterapi med check-point hæmmere
(anti-CTLA-4, anti PD-1 og anti-PD-L1)

Version 10-07-2018

www.immuntox.dk

Kan downloades som app til IOS og Android: Immuntox

Ansvarshavende redaktører:

Professor, overlæge Inge Marie Svane
E-mail: inge.marie.svane@regionh.dk
Onkologisk Afd. R
Herlev Hospital

Overlæge Henrik Schmidt
E-mail: henrschm@rm.dk
Kræftafdelingen
Aarhus Universitetshospital

Overlæge Lars Bastholt
E-mail: lars.bastholt@rsyd.dk
Onkologisk Afd. R
Odense Universitetshospital

Bivirkninger relateret til behandling med immun checkpoint hæmmere 3

Patient kort - immunterapi. 4

Information – immunterapi - Praktiserende læge 5

Blodprøver 6

Infusionsreaktion 7

Håndtering af hudtoksicitet 8

Håndtering af diarré/colitis 11

Håndtering af behandlingsrelaterede endokrinopatier 14

Håndtering af immunrelateret hepatotoksicitet 18

Håndtering af immunrelateret pulmonal toksicitet 20

Håndtering af okulær toksicitet 222

Håndtering af myocarditis 244

Håndtering af neuropati 277

Håndtering af immunrelateret nefrotoksicitet 299

Steroidbehandling af immunrelaterede bivirkninger 311

Vaccination i forbindelse med immunterapi 32

Referenceliste 33

Bivirkninger relateret til behandling med immun checkpoint hæmmere

Antistoffer rettet mod CTLA4, PD-1 eller PD-L1 udøver deres virkning på aktiverede T-celler i immunsystemet. Antistofferne blokerer nogle af de naturlige bremser på T-cellerne. Derved muliggøres en immunologisk reaktion rettet mod patientens tumorceller.

Grundet disse virkningsmekanismer kan der opstå immunrelaterede bivirkninger (irAE), som for de flestes vedkommende er milde, men som kan udvikle sig til at være alvorlige og behandlingskrævende. Patienterne er instrueret i at henvende sig, selv med relativt svage symptomer, med henblik på en vurdering af, om disse skal undersøges nærmere og behandles. Det er vigtigt, at der reageres tidligt og adækvat på disse immunrelaterede bivirkninger.

I denne manual præsenteres bivirkninger og vejledning til diagnostik, behandling og opfølgning på bivirkninger i de forskellige organområder; primært ved hjælp af algoritmer.

Grad 3-4-bivirkninger til CTLA4 antistoffer ses hos ca. 22 % af de behandlede, og for PD-1/PD-L1-antistofferne er det ca. 5 %. Det er den samme type bivirkninger, man ser for de to stofgrupper, men det er ofte ikke de samme bivirkninger, man ser ved patienter, som konsekutivt får de to behandlingstyper.
Ved kombination af anti-CTLA4 og anti-PD1 øges risikoen for grad 3-4 bivirkninger til ca 55%. Ved anvendelse af anti-CTLA4 umiddelbart efter ophør med anti-PD1 er der beskrevet øget risiko for bivirkninger på niveau med risikoen ved kombinationsbehandling.

Endelig er det vigtigt at vide, at den enkelte patient godt kan få immunrelaterede bivirkninger fra flere organsystemer; det ses især hyppigt hos ptt, der får immun-kombinations behandling. Desuden er det vigtigt at bemærke, at bivirkningerne også kan opstå sent i behandlingsforløbet.

Alle bivirkninger er graderet i henhold til CTCAE version 4.03. Uddybende litteratur vedr. håndtering af immunrelaterede bivirkninger [1-9].

Patient kort - immunterapi.

Immunterapi

Når man får immunterapi er der risiko for alvorlige bivirkninger. Disse skal erkendes så tidligt som muligt, og det er derfor vigtigt, at du kontakter sygehuset hvis du oplever nogle af disse symptomer:

- Pneumonitis: bivirkning i lungerne karakteriseret ved nyopstået, tør hoste, åndenød.
- Diarre/colitis: bivirkning i tarmsystemet karakteriseret ved øget antal afføringer evt med samtidige mavesmerter, blod i afføringen, eller mørkfarvet, ildelugtende afføring.
- Nefritis: bivirkning i nyrene karakteriseret mørkfarvning af urinen, skummende el meget uklar urin
- Hormonforstyrrelser: bivirkning i de hormonproducerende kirtler. Svære at erkende: hovedpine, ekstraordinær træthed, smerter bag øjnene, depression, synsforstyrrelser
- Dermatitis: bivirkning i huden karakteriseret ved rødme og udslæt, ofte kløende. Væskende sår er et mere alvorligt tegn
- Neuritis: bivirkninger i nervesystemet med påvirkning af eksempelvis bevidsthed, kraft- og følesans i arme/ben.

Kontakt information

Information – immunterapi - Praktiserende læge

Kære kollega

Denne pt er påbegyndt immunterapi. Vi vil gerne henlede din opmærksomhed på at immunterapien, kan medføre bivirkninger der ligner autoimmune sygdomme. Det er vigtigt, at disse bivirkninger erkendes tidligt og at korrekt behandling iværksættes så tidligt som muligt

Hvis nogle af nedenstående symptomer erkendes bør der akut rettes henvendelse til behandlende sygehus med henblik på råd og vejledning:

- ✓ Pneumonitis: karakteriseret ved nyopstået, tør hoste, åndenød.
- ✓ Diare/colitis: karakteriseret ved øget antal afføringer evt med samtidige mavesmerter, blod i afføringen, eller mørkfarvet, ildelugtende afføring.
- ✓ Nefritis: karakteriseret mørkfarvning af urinen, skummende el meget uklar urin
- ✓ Endokrin toxicitet. Svære at erkende: hovedpine, ekstraordinær træthed, smerter bag øjnene, depression, synsforstyrrelser
- ✓ Dermatitis: karakteriseret ved rødme og udslæt, ofte kløende. Væskende sår er et mere alvorligt tegn.

Der kan også opstå bivirkninger fra andre organsystemer: neurologiske, hæmatologiske, vaskulære, okulære etc

Håndtering af disse dysimmune toxiciteter kræver specifikke handlinger og tidlig intervention kan være vigtig for at bremse disse bivirkninger, der kan udvikle sig livstruende. Det er derfor vigtigt, at der skabes tidlig kontakt til den behandlende afdeling.

Kontakt information

Blodprøver

Basisblodprøver som tages før start og før hver behandling:

- Hæmoglobin, leukocytter, leukocyttype, trombocytter,
- CRP,
- kreatinin, kalium, natrium, glukose,
- albumin, basisk fosfatase, ALAT el ASAT, INR, bilirubin, LDH,
- TSH, T4, kortisol.
- Amylase
- Troponin (TnT el TnI) (standard ved immunocombo under kombinationshandlingen)
- Kreatinkinase, MB (standard ved immunocombo under kombinationshandlingen)

Ved mistanke om endokrin toksicitet suppleres med:
- Corticotropin (ACTH), frit T4, T3
- FSH, LH (kvinder)
- Testosteron (mænd), evt rutinemæssigt.

Ved mistanke om hepatitis suppleres med:
- Hepatitis A virus (HAV) antistof (total, IgM)
- Hepatitis B virus (HBV) c-antistof (total & IgM)
- Hepatitis B virus (HBV) s-antigen (HBsAg)
- Hepatitis B virus (HBV) s-antistof (immuntest)
- Hepatitis C virus (HCV) antistof (IgG) (screening)
- Hepatitis E virus (HEV) antistof (IgG, IgM)
- Epstein-Barr virus (EBV) antistof (EBNA, IgG, IgM)
- Cytomegalovirus (CMV) antistof (total, IgM)

Ved colitis skal der som forberedelse til behandling med Infliximab, foreligge:
- Hepatitis A virus (HAV) antistof (total, IgM)
- Hepatitis B virus (HBV) s-antigen (HBsAg)
- Hepatitis C virus (HCV) antistof (IgG) (screening)
- Quantiferon-TB gold

Ved mistanke om kardiotoxicitet suppleres med:
- Troponin (TnT el TnI)
- Kreatinkinase, MB

Ved mistanke om myositis suppleres med:
- Kreatinkinase
- Myoglobin

Infusionsreaktion

Infusionsreaktioner i forbindelse med behandling med checkpoint hæmmere

Infusionsrelaterede reaktioner kan opstå i forbindelse med alle checkpoint hæmmere. Frekvensen af infusionsrelateret varierer mellem de enkelte checkpoint hæmmere.

Der kan opstå feber, kulderystelser, kløe og rødme af huden og urticaria. I sværere tilfælde dyspnø, stridorøs vejrtrækning, muskel- ledsmerter og abdominal smerter.
Ved svære tilfælde (grad 3-4) afbrydes infusionen. Tilfældet behandles som andre svære infusionsreaktioner i henhold til lokal retningslinje.

Genoptagelse af behandling bør kun ske efter drøftelse med speciallæge.

Ved lette tilfælde pauseres infusionen. Overvej behandling med NSAID præparat (paracetamol, ibuprofen) og antihistamin. I sjældne tilfælde moderat dosis steroid. Når reaktionen er svundet, kan behandling genoptages, idet infusionshastigheden nedsættes med 50% (infusionstid fordobles).

Ved efterfølgende behandlinger overvejes præmedicinering med NSAID, antihistaminer og evt steroid.

Håndtering af hudtoksicitet

Hudtoksicitet er en af de hyppigere bivirkninger til disse behandlinger.

Det kliniske billede
Der kan udvikles inflammatoriske hudreaktioner, der har karakter af erytem, eksem eller eksantem med generaliseret kløe og nældefeber. Endvidere er det kendt, at hudlidelser på autoimmun basis, specielt psoriasis eller rosacea, kan forværres under immunterapien. Graderingen af hud bivirkninger afhænger selvfølgelig af typen af bivirkning og refererer til den gængse CTCAE version 4. Den mest almindelige hud bivirkning er udslættet og her refererer til % af afficeret hud. Grad 2 til 3 skiller ved 30 % af hud overfladen. Nedenstående figur kan anvendes i denne vurdering.

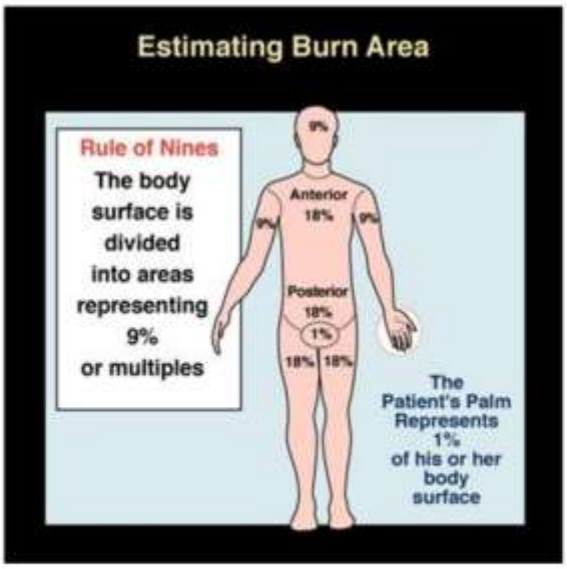

Behandlingsvejledning

Essentielle fedtsyrer til lindring af hudgener
Der er observeret god symptomatisk effekt på hudtørhed og hudkløe af planteoliebaserede kosttilskud med de essentielle fedtsyrer omega-3, omega-6 og omega-9. Følgende produkter er hidtil blevet brugt af patienter: "Perfect Balance" eller "Livets Olie", som er en olie fra hørfrø- og kæmpenatlys-planterne. De omtalte kosttilskud kan købes i helsekostbutikker, og andre fabrikater kan formentlig også anvendes. Kosttilskuddet kan fås såvel i flydende form som i kapsler.

Patienterne kan påbegynde brugen af olien enten inden ved kombinationsbehandling eller senere hvis der opstår symptomer under behandlingen.
Daglig anbefalet dosis er 2 spiseskefulde om morgenen. Man tager en spiseskefuld og holder olien fortil i munden og skyller efter med juice, gerne appelsin- eller æblejuice. Herefter skefuld nummer 2 på samme måde. Ved fortsat hudtørhed eller kløe kan man gradvist øge dosis med 2 spiseskefulde ad gangen og fordele det over 2-3 gange dagligt. Vi har erfaring med patienter, som har anvendt op til 10 spiseskefulde dagligt. Olien kan supplerende smøres direkte på de mest irriterede hudområder, ligeledes 2-3 gange dagligt. Behandlingen bør suppleres med fugtighedscreme.

Ved manglende effekt heraf kan nedenstående anvendes:

Tablet Tavegyl (clemastin) 1 mg x 2 eller Zyrtec (cetirizin) 10 mg dgl.
Ved svær kløe kan glukokortikoid-cremer anvendes.

Alle patienter, der udvikler grad 3-4 hudtoksicitet, bør holde pause med behandlingen.

Videre behandling udsættes til svind af hudbivirkningerne til grad 0-1. Ved udsættelse af behandlingen mere end 30 dage skal videre behandling konfereres med speciallæge.

Konkomitant medicinering
Få tilfælde af fatal toksisk epidermal nekrolyse (TEN) er blevet registreret hos patienter under immunterapi. I de tilfælde fik patienterne samtidig behandling med andre stoffer, som kan give TEN, og det er derfor vigtigt, at vi omhyggeligt registrerer, når patienter har svær hudtoksicitet, og om der er andre medikamenter, som kan forårsage TEN samt evt. forværre immunrelateret hudtoksicitet. Af eksempler på stoffer, som kan give TEN i sjældne tilfælde, og som vi af og til anvender hos vores patienter, kan nævnes:

Lamictal (lamotrigin) – antiepileptikum
Ciprofloxacin – antibiotikum af quinolon-type
Pantoloc (pantoprazol) – syrepumpehæmmer.

Man skal *altid* være opmærksom på, at også andre stoffer kan inducere hudtoksicitet.

Husk at overveje en dermatologisk vurdering i de alvorlige tilfælde.

Håndtering af hudtoksicitet

CTC hud	Håndtering	Followup
Udred for ikke-immunrelateret årsag til toksicitet, og behandl relevant.		
**Grad 1-2**	Symptomatisk behandling<br>Planteolier<br>Steroidcreme<br>Evt. antihistaminer<br>Fortsæt checkpoint-hæmmere	Symptomer bedres: Fortsæt behandling med checkpoint-hæmmere<br>Symptomer forværres eller persisterer:<br>Overvej prednisolon 25-50 mg og udtrapning ved grad 0-1 hvis steroidcreme ikke har været effektivt<br>Herefter kan behandling med checkpoint-hæmmere fortsættes
**Grad 3-4**	Pausér behandling med checkpoint-hæmmere<br>Dosisøgning af planteolier<br>Konferér evt. med dermatolog om behandling med steroidcreme<br>Prednisolon 50-100 mg ved grad 4 toksicitet. Konferér med dermatolog	Symptomer bedres til grad 1:<br>Udtrapning af prednisolon over 3-4 uger<br>Genoptag behandling med checkpoint-hæmmere, medmindre der er registreret grad 4-toksicitet

Side 10 af 33

Version10.07.2018

Håndtering af diarré/colitis

Diarré forekommer under behandling med ipilimumab som en immunrelateret bivirkning, mindre hyppigt ved nivolumab og pembrolizumab. I nogle tilfælde er forløbet fredeligt og remitterer på almindelig symptomatisk behandling, men diarréerne kan også udvikle sig til en livstruende tilstand med risiko for tarmdilatation og ultimativt perforation, og derfor skal disse patienter følges nøje.
Der er set store forskelle på behandlingsresponset for solumedrol/infliximab afhængigt af om patienten er behandlet med enkelt stof immunterapi eller med kombinationsimmunterapi.

- Grad I – tbl. Imodium a 2 mg
- Grad II – vurderes akut med henblik på optimal udredning.
  - Tbl. Imodium a 2 mg
  - Ved manglende effekt af Imodium eller forværring af grad 1 behandlet med Imodium: Prednisolon 25-50 mg dgl.
  - Ved effekt bevares dosis i 1 uge og herefter aftrappes prednisolon over 4-6 uger
  - Ved manglende effekt efter 3 dage behandles som grad III
- Grad 3-4 – indlægges akut.
  - Højdosis i.v. steroid (Solumedrol 80 mg x 1 el. 40 mg x 2)
  - Ved effekt skiftes efter 3 dage til tbl. prednisolon 100 mg dgl., fasthold dosis 1 uge og udtrappes herefter primært med 12.5 mg hver 5-7 dag.
  - Ved manglende effekt efter 3 dage bør der laves koloskopi med biopsi af en erfaren endoskopør grundet øget risiko for perforation. Infliximab 5 mg/kg gives dagen efter. Hvis der udover makroskopisk inflammation er betydeligt reduceret albumin til under 25 g/l bør det overvejes at behandle med en dosis infliximab på 10mg/kg legemsvægt da det intestinale tab af biologisk lægemiddel kan forventes at være øget. Hvis en patient behandles med biologisk terapi i en dosis på 10mg/kg legemesvægt skal der allerede efter en uge foretages en vurdering med henblik på eventuel anden dosis infliximab.
  - Når diarre er i grad 1, kan prednisolon nedtrappes med 12,5 mg hver 5-7 dag.
  - Ved recidiv af diarre under aftrapning af prednisolon udredes pt. for anden årsag (primært C. difficile diagnostik) og infliximab-genbehandling overvejes.

Ved kombinationsimmunterapi med ipilimumab/nivolumab kan tilstanden være yderst behandlingsresistent og det anbefales at behandle efter følgende forskrift (gastroenterologisk afdeling informeres om at der er givet kombinationsbehandling):

  - Hvis der efter 3 døgns intravenøs højdosis steroidbehandling ikke er respons skal der foretages koloskopi med biopsi med henblik på behandling med infliximab. Der anvendes infliximab i doseringen 10 mg/kg med en ugentlig re-vurdering ved speciallæge i gastroenterologi.
  - Ved manglende effekt gives 2. serie infliximab efter en uge i doseringen 10 mg/kg
  - Hvis der efter 2 serier infliximab kun er kortvarig effekt (2-3 dage) kan det overvejes at give 3. serie infliximab i doseringen 10 mg/kg kombineret med vedolizumab 300 mg .

- Ved akut indlæggelse vurderes:
  - Almentilstand
  - Blodprøver: hæmatologi m. differentialtælling, CRP, væske- og levertal
  - Fæcesdyrkninger for tarmpatogene bakterier og clostridium difficile
  - Fæces for calprotectin [7]

- Alle klinisk abdominalt påvirkede patienter skal have foretaget CT-oversigt over abdomen for at udelukke colon dilatation og perforation.

- **Før start** af prednisolon-behandling forberedes pt. til evt. senere behandling med infliximab:
  - Kronisk hepatit serologi (HBsAg, anti-HBc, anti-HCV)
  - Udredning for tuberkulose – QuantiFERON-TB gold
  - Vi har altid en baseline CT af thorax, og derfor behøves ikke frisk rtg. af thorax for at undersøge for latent TB.

Followup efter infliximab

- Effekt og bivirkninger af infliximab vurderes
  - Dg. 10-14 efter første infliximab infusiongentages fæces for calprotectin, og pt. ses ambulant (tidligere ved kombinationsbehandling i henhold til ovenstående)
- Dosering af prednisolon i aftrapningsfasen
- Vurdering af behov for yderligere behandling med infliximab må aftales lokalt med gastroenterologisk afdeling
- Husk at gentage fæces dyrkninger (specielt Clostridium Difficile) ved fortsat diarré.

Håndtering af gastrointestinal toksicitet

Udred for infektion som årsag til diarré, og behandl relevant.

CTC diarré/colitis	Håndtering	Followup
**Grad 1:**<br>**Øget antal aff. < x 4/dag**<br>**Colitis: ingen symptomer**	Symptomatisk behandling - Loperamid<br>Fortsæt checkpoint-hæmmere	Tæt klinisk monitorering<br>Instruér patient i at henvende sig ved forværring af symptomer
**Grad 2:**<br>**Øget antal aff. 4-6 x/dag**<br>**Colitis: abdominalsmerter, blod i afføring**	Klinisk vurdering<br>Loperamid og ved manglende effekt inden for 2 dage. skift til moderate doser steroid<br>Pausér med checkpoint-hæmmere	Toksicitet grad 0-1: genoptag beh. m. checkpoint-hæmmere<br>Ved manglende effekt af Loperamid: udredning mhp. Infliximab, start moderat dosis prednisolon 25-50 mg dgl. Pausér med checkpoint-hæmmere til toksicitet grad 1<br>Ved forværring el. manglende effekt af steroid behandles som grad 3-4. Udtrapning af prednisolon over 4-6 uger
**Grad 3-4:**<br>**Diarré mere end 7 x/dag, inkontinens, i.v. væske > 24 h, alment påvirket**<br>**Colitis: Feber, ileus, mistanke om perforation**	Stop med checkpoint-hæmmere. Indlæg akut, og forbered patienten mhp. eventuel infliximab -behandling. CT-scanning af abdomen anbefales for at vurdere dilatation af colon.<br>Højdosis steroid i.v. Solumedrol (80 mg x 1 el. 40 mg x 2)<br>Ved manglende effekt efter 2-3 døgn foretages endoskopi m. biopsi og efterfølgende infliximab.	Overgå til peroral prednisolon 100 mg x 1 ved effekt af i.v. steroid efter dag 5 (grad 1-2 diarre).<br>Prednisolon udtrappes over 4-6 uger<br>Hvis symptomer persisterer på 3. dag eller recidiverer under udtrapning, da behandling ved gastroenterolog med koloskopi og infliximab (OBS dosering).<br>Efter infliximab: aftrapning af prednisolon over 4-6 uger elle længere tid

Side 13 af 33

Version10.07.2018

Håndtering af behandlingsrelaterede endokrinopatier

Symptomer
De endokrine bivirkninger kan være vanskelige at diagnosticere klinisk, og patienterne bør derfor følges løbende for hormon niveauer. De fleste tilfælde af endokrin toxicitet forløber fredeligt, men de kan medføre langvarig og irreversibelt tab af hormonproduktion og dermed behov for livslang substitution.

Hypofysitis
Specielt er det vigtigt at erkende den behandlingsinducerede hypofysitis, hvor en tidlig iværksættelse af steroidbehandling kan være livsvigtigt. Pt følges med en spot-cortisol som supplement til den kliniske vurdering. Det er vigtigt at huske at dette hormon har en udpræget døgnvariation og det kun er for lave værdier der skal reageres på.

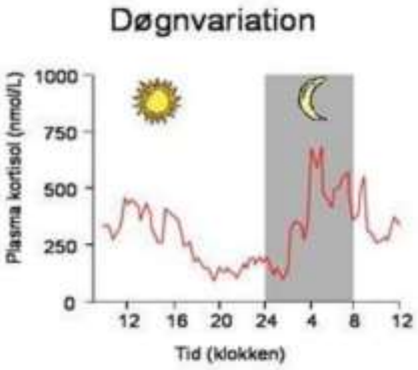

Kilde: Medicinsk Kompendium (1994)

På grund af denne døgnvariation og fordi vi ikke kan få denne analyse foretaget akut vil vi i mange tilfælde være nødsaget til at ordinere den planlagte dosis ud fra en klinisk vurdering kombineret med en vurdering af tidligere værdier af se-Cortisol.

Grundet komorbiditet og andre bivirkninger relateret til den onkologiske behandling er det lige så ofte patientens symptomer som en tilfældig biokemisk screening, der leder til diagnosen hypofysit. Klassisk ses dog *kraftig hovedpine lokaliseret bag øjnene*.
Symptomer på hormonudfald kan være:
Træthed (TSH/ACTH) samt tegn til *akut binyrebarkinsufficiens* (mavesmerter, opkast, diarré, feber, hypotension, hyponatriæmi og evt. hypoglykæmi)
Impotens og nedsat libido
Muskelsvækkelse (FSH, LH, testosteron)
Meget sjældent ses chiasma-tryk, øjenmuskellammelser eller diabetes insipidus.

I den primære udredning af endokrinopatier tages følgende blodprøver *inden eventuel opstart af prednisolon*: Hypofysehormonerne LH, FSH, ACTH (corticotropin) og TSH, de perifere hormoner T3, T4, kortisol og testosteron. MR-scanning af hypofysen er kun relevant i uklare tilfælde, da diagnosen sædvanligvis stilles på blodprøverne.

Videre udredning foretages akut ved kontakt til endokrinologisk bagvagt.

Prognose og kontrol
ACTH-mangel synes oftest permanent. FSH, LH og testosteron-funktionen genvindes ofte (57 %), mens det for TSH er varierende (37-50 %). Hypofysit-patienter følges i endokrinologisk ambulatorium.

Akut binyrebarkinsufficiens på baggrund af hypofysitis (sekundær binyrebark insufficiens)
Symptomer: Bl.a. gastrointestinale klager, hypotension, hyponatriæmi. Diagnosen kan kun stilles ved Synachten®-test. Tolkning er en specialistopgave, da testen kan være normal ved akut ACTH-udfald. Plasma spot-kortisol er sjældent diagnostisk; dog vil tilfældigt målte værdier på hhv. < 50 nmol/l eller > 500 nmol/l tyde på henholdsvis binyrebarkinsufficiens eller normal binyrebarkfunktion.
Primær binyrebarkinsufficiens er sjælden, men beskrevet i litteraturen. Behandles som beskrevet forneden.

Behandling
Hvis endokrinologisk bagvagt ikke træffes, eller situationen er truende, gives *i.v. hydrocortison 100 mg x 1*, hvorefter endokrinolog kontaktes mhp. videre plan og diagnostik. Efter akut endokrinologisk tilsyn opstartes vanligvis hydrocortison substitutionsbehandling, mens stillingtagen til immunsupprimerende prednisolon-behandling påhviler onkolog. Denne påbegyndes samtidig med substitutionsbehandlingen og er prednisolon 50 mg dagligt i en uge, hvorefter aftrapning kan påbegyndes med 12,5 mg hver 5.-7. dag med en samlet behandlingsvarighed på 4-5 uger.

Thyroideapåvirkning
Der er flere former for thyroideadysfunktion relateret til checkpoint-hæmmere-behandling:

		TSH	T3	T4
Hypotyreose	Hypofysær hypotyroidisme	Lav-normal (<10)		Lav
	Primær hypotyroidisme	Høj		Lav
	Subklinisk primær hypotyreose	Høj		Normal
	Hypothyroid fase af Thyreoidit	Høj		Lav
Hypertyreose	Hyperthyroid fase af Thyreoidit	Lav	Høj	Høj T4>T3
	Graves Thyreotoxikose	Lav	Høj T3>T4	Høj
	Iod induceret thyreoitit	Lav	Høj	Høj T4>T3

NB: Ved kronisk sygdom ses ofte lav T3 med samtidig normal TSH og T4. Højdosis prednisolon kan i sig selv supprimere TSH.

Der ses hyppigt TSH ændringer under checkpoint-hæmmer behandling uden ledsagende symptomer eller påvirkning af de perifere hormoner (T3/T4). Disse ændringer skal ikke behandles, blot følges.

Hyperthyreose
Endokrinolog bør vurdere, om der er behov for videre udredning med thyroideaskintigrafi/ultralyd. Thyroideaskintigrafi vil vise opladning ved autoimmun thyroidit, men ikke ved henfalds-thyroidit. Behandling er blokerende behandling og/eller beta-blokkerende midler, afhængig af patientens symptomer, efter anbefaling fra endokrinolog. Til pt med symptomgivende toxisk thyroidit kan man overveje at give en moderat dosis steroid i en kortere periode - f.eks. prednisolon, 25-50 mg dgl.

Vær opmærksom på, at der er risiko for hypothyroidisme efter en primær hypertyroid fase med muligt behov for behandling med T4-præparat, hyppigst i en kortere periode. Derfor kan det være relevant at starte behandling med T4 så snart der ses et fald i T4 og stigende TSH i blodet. Konferer dette med lokal endokrinolog.

Hypothyreose
Hypothyroidisme er mere almindelige med anti-PD-1 behandling end med CTLA-4 hæmmere og er sjældent alvorlig. Det kan være relevant at starte behandling med T4 (eks. Eltroxin el Euthyrox) når der ses et fald i T4 og stigende TSH i blodet. Konferer dette med lokal

endokrinolog. Der er ikke behov for prednisolon ved milde/moderate symptomer. Checkpoint-hæmmer behandlingen kan fortsætte samtidig med substitutionsbehandling. Ved svære symptomer bør man overveje prednisolon. Hypothyroidisme er som hovedregel ikke reversibel.

Håndtering af endokrinopatier

CTC endokrinopati	Håndtering	Followup
**Mistanke om hypofysitis**<br>**(hovedpine eller ekstraordinær træthed eller synsforstyrrelser)**	Blodprøvescreening for endokrinopatier<br>Konferer **akut** med endokrinolog, når blodprøvesvar foreligger.<br>Endokrinolog starter substitutionsbehandling. Onkolog starter immunosuppressiv behandling.<br>Pause checkpoint-hæmmer.<br><br>Ved normal biokemi og persisterende symptomer overvejes ny blodprøvescreening efter 2-3 uger	Efter 1 uges behandling med prednisolon kan aftrapning over yderligere 3 uger foretages<br>Behandling med checkpoint-hæmmere kan genoptages efter udtrapning af prednisolon
**Mistanke om akut binyrebarkinsufficiens:**<br>**Svær dehydrering, hypotension, shocksymptomer**	Udeluk sepsis<br>Akut kontakt til endokrinolog<br>Ved svære symptomer gives i.v. Solu-cortef 100 mg x 1 samt 100 mg prednisolon	Efter 1 uges behandling med prednisolon kan aftrapning over 4 uger foretages<br>Behandling med checkpoint-hæmmere kan genoptages efter udtrapning af prednisolon

Håndtering af immunrelateret hepatotoksicitet

En algoritme (se næste side) for udredning og behandling af hepatitis er udviklet til patienter, som ved baseline har normal ALAT el ASAT (≤ 2,5 x øvre normalområde). Det er ganske væsentligt at monitorere INR, da det er det bedste mål for leverens funktion. Bilirubin kan udelukkende være forhøjet pga kolestase. Stiger INR er der decideret metabolisk leversvigt uanset ALAT forhøjelse. Stiger INR over 1,3 bør man give steroid uanset ALAT. Den tilgang er kopieret fra håndteringen af autoimmun hepatitis, hvor man ved INR stigning IKKE ser tiden an uanset ALAT. ALATs halveringtid er ganske kort (4-6 timer). Hurtigt udviklende nekroser leveren vil udarte sig som en omfattende enzymstigning afløst af INR stigning med enzymfald. Timingen af blodprøverne vil afgøre i hvilken fase dette fanges.

Grad	ALAT el ASAT	Bilirubin	INR
1	< 3 x ULN	< 1,5 x ULN	< 1,5 x ULN
2	3-5 x ULN	1,5-3,0 x ULN	1,5-2,5 x ULN
3	5-20 x ULN	3,0-10 x >ULN	> 2,5 x ULN
4	> 20 x ULN	> 10 x ULN	-

Udredningsprogram for at afklare ikke-immunterapi relateret årsag til leverpåvirkningen gennemføres (se under blodprøver)

Billeddannelse, sædvanligvis ultralydsundersøgelse, af lever og galdeveje skal gennemføres ved forhøjet bilirubin. Patienter med øvre højre kvadrant-mavesmerter og/eller uforklaret opkastning skal have taget levertal akut.

Ved manglende effekt af behandling med prednisolon skal der konfereres med hepatolog mhp yderligere udredning, herunder om leverbiopsi skal overvejes.

Håndtering af levertoksicitet

CTC hepatitis	Håndtering	Followup
**INR < 1,3 og baseline ASAT/ALAT/bilirubin: Stigning fra grad 0 til 1, eller stigning fra grad 1 til 2**	Fortsæt checkpoint-hæmmere	Monitorering af blodprøver ugentligt
**INR > 1,3 el baseline ASAT/ALAT/bilirubin:**<br><br>**Stigning fra grad 0 til ≥ grad 2, eller stigning fra grad 1 til ≥ grad 3**	Pauser checkpoint-hæmmer. Blodprøver for at udelukke anden ætiologi.<br>Ved samtidig forhøjet bilirubin skal der foretages billeddiagnostik	Overvej moderate doser steroid (prednisolon 25-50 mg dgl.) v manglende bedring trods pause. Prednisolon udtrapning over 3-4 uger.<br>Fortsat checkpoint-hæmmer vurderes ved speciellæge.
**ASAT/ALAT > 5 x ULN eller**<br>**bilirubin > 3 x ULN eller**<br>**INR > 2,5 x ULN**	Ophør med checkpoint-hæmmere<br>Højdosis steroid iv., methylprednisolon 1-2 mg/kg<br>Monitorér blodprøver dagligt ved INR > 2,5 x ULN, ASAT el ALAT >10xULN eller bilirubin > 5 x ULN	Fortsæt prednisolon til toksicitetgrad 2, og trap herefter ud over 4-6 uger. Ugentlig monitorering af blodprøver.<br>Hvis symptomer persisterer > 2 dage, forværres eller recidiverer: Mycophenolat mofetil (CellCept) 1g BID

Udred for ikke-immunrelateret årsag til toksicitet, og behandl relevant. **Ptt. på i.v.-steroid kan overgå til peroral behandling, så snart den kliniske situation bedres.**

Side 19 af 33

Version10.07.2018

Håndtering af immunrelateret pulmonal toksicitet

Pneumonitis er en mindre hyppig bivirkning, der graderes som følger:

Grad	Pneumonitis
1	Asymptomatisk; kun klinisk eller diagnostisk observation; ingen indikation for intervention
2	Symptomatisk; indikation for medicinsk intervention; begrænser ADL-aktiviteter
3	Svære symptomer; begrænser ADL-aktiviter; indikation for ilttilskud
4	Livstruende, svært respiratorisk påvirket; indikation for akut intervention (f.eks. trakeostomi eller intubation)

Hos patienter med nye eller forværrede pulmonale symptomer laves CT-scanning af lungerne med henblik på pneumonit.

Hvis der herefter er mistanke om pneumonit relateret til behandling med checkpoint-hæmmere, følges algoritmen på næste side.

Håndtering af pulmonal toksicitet

CTC pulmonal tox	Håndtering	Followup

Ved nye eller forværrede pulmonale symptomer laves CT thorax og udvidet lungefunktionstest.
Udred for ikke-immunrelateret årsag til toksicitet, og behandl relevant.
Patienter på i.v. steroid kan overgå til peroral behandling, så snart den kliniske situation bedres.

**Kat. A: asymptomatisk, alene m. radiologiske forandringer**<br>**(grad 1 pneumonitis)**	Pausér checkpoint-hæmmere<br>Monitorér hver 2.-3 dag, overvej antibiotika, overvej prednisolon (25-50 mg/dg)	Gentag radiologisk us.<br>Fortsæt monitorering, til forandringerne er væk. Genoptag checkpoint-hæmmere, når evt. prednisolonbehandling er ophørt
**Kat. B: lette el. moderate symptomer eller forværring ift. baseline**<br>**(grad 2 pneumonitis)**	Pausér checkpoint-hæmmere<br>Monitorér under indlæggelse, overvej antibiotika, og giv prednisolon (25-50 mg/dg). Overvej BAL	Gentag radiologisk og klinisk vurdering dgl.<br>Stabil: fortsæt evaluering<br>Forværring: se kat. C<br>Bedring: udtrapning af prednisolon over 3-4 uger, og genoptag herefter checkpoint-hæmmere
**Kat. C: Svære, nye symptomer el. markant forværring ift. baseline. Potentielt livstruende**<br>**(grad 3/4 pneumonitis)**	Ophør med checkpoint-hæmmere<br>Overvej antibiotika, og giv methylprednisolon (1-2 mg/kg) dagligt. Overvej bronkoskopi og lungebiopsi efter kontakt til lungemediciner	Gentag radiologisk og klinisk vurdering dgl.<br>Stabil el. forværring efter 48 timer: overvej suppl. immunosuppresiv beh. (mycophenolat mofetil (CellCept) el. infliximab) og supplerende i.v. antibiotika<br>Udtrapning af prednisolon ved bedring over 5-6 uger

Side 21 af 33

Version10.07.2018

Håndtering af okulær toksicitet

Patienter, der behandles med checkpoint-hæmmere, kan udvikle okulær toksicitet. Dette vil ofte være i form af:

- Uveit
- Øget lysfølsomhed
- Sløret syn
- Skleralt erytem
- Tørhed i øjnene
- Smerter i øjnene.

Håndtering af okulær toksicitet

CTC okulær	Håndtering	Followup
Udred for ikke-immunrelateret årsag til toksicitet, og behandl relevant.		
**Grad 1**	Oftalmologisk vurdering og behandling i deres regi.	Symptomer bedres: fortsæt behandling med checkpoint-hæmmere<br>Symptomer persisterer eller recidiverer: Overvej at stoppe behandling
**Grad 2-4**	Pausér behandling med checkpoint-hæmmere<br>Oftalmologisk vurdering og behandling i samråd med denne<br>Start prednisolon 50-100 mg dgl.	Symptomer bedres til grad 1:<br>Udtrapning af prednisolon over 3-4 uger<br>**Genoptag behandling med checkpoint-hæmmere efter konference m. speciallæge, medmindre der er registreret grad 4, relateret toksicitet**

Håndtering af myocarditis

Denne bivirkning er i flere studier rapporteret med dødelig udgang. Den er rapporteret primært i forbindelse med adjuverende behandling med Ipilimumab, 10 mg/kg el i forbindelse med immun-kombinationsterapi (anti-CTLA4 kombineret anti-PD1)[11]. Det vurderes ud fra store toxicitetsdatabaser, at det er en toxicitet der opstår tidligt i behandlingsforløbet. Det er dog stadig en sjælden komplikation og forekommer i de anførte regimer kun hos ca 1% af patienterne.

Symptomer på myocarditis er ikke særligt specifikke. Omfatter brystsmerter, dyspnø, træthed. Syndromet kan være kombineret med myositis og derfor kan muskelsmerter være en del af symptombilledet.

Grad	Myocarditis
1	Asymptomatisk med biokemiske abnormaliteter (ex CK-MB, TnI/TnT stigning ift baseline)
2	Symptomatisk; med biokemiske abnormaliteter (ex CK-MB, TnI/TnT stigning ift baseline)
3	Svære symptomer i hvile; begrænser ADL-aktiviteter.
4	Livstruende, svært kardielt påvirket; indikation for akut intervention.

Autopsi-undersøgelser i ovenstående publikation viste et histologisk billede, der minder om det, der ses efter afstødning efter hjertetransplantation. Der blev observeret forandringer i tværstribet og i kardiel muskulatur.

Symptomgivende myocarditis har en meget dårlig prognose og har i ovenstående publikation responderet dårligt på højdosis steroid behandling. Der angives desværre ikke forslag til forholdsregler for at undgå den høje mortalitet. Kan man ved indgreb tidligt i processen have større chance for bremse den immunologiske reaktion?

Anførte retningslinjer anbefales fulgt ved alle ptt der får immun-kombinationsterapi (CTLA-4 og PD-1 antistof)

Baseline undersøgelser på alle ptt der får immun-kombinationsterapi:
- EKG
- Blodprøver (se under blodprøver)

Blodprøverne følges hver 3. uge i forbindelse med de første 12 ugers behandling. EKG gentages på klinisk indikation. Herefter undersøgelser hver 3. mdr ifm evaluering af sygdomsstatus

I følgende situationer bør pt visiteres til kardiologisk vurdering:
- CK-MB > 10 mikrogr/l
- TnI/TnT > 500 ng/l

Ptt med stigning i parametre, men under disse værdier bør følges tættere

Kardiologisk udredning vil omfatte:
- Echocardiografi.
  - Ved normal echocardiografi tilbagevisiteres pt til onkologisk afd, hvor tæt opfølgning bør fortsætte under det videre behandlingsforløb.
  - Ved patologisk echocardiografi overvejes
    - MR-scanning af cor
    - Myokardiebiopsi

Behandling:

Ptt med påvist myocarditis skal behandles med steroid. Ved grad 1 der ikke bedres og grad 2 anvendes peroral steroid, prednisolon 100 mg/dg. Ved grad 3-4 anvendes Methylprednisolon 1-2 mg/kg iv. Behandlingen bør gives i kardiologisk regi idet telemetri under forløbet er nødvendig. Behandling kan evt kombineres/suppleres med Infliximab.

Behandling aftrappes over 4-6 uger når toxicitet er bedret til <= grad 1.

Håndtering af myocarditis

CTC myocarditis	Håndtering	Followup
**Grad 1**	Kardiologisk vurdering og udredning.	Symptomer bedres:<br>Vurdering v speciallæge af mulig fortsat immunterapi, evt som enkeltstofbehandling<br>Symptomer persisterer eller recidiverer: stop immunkombinationsbehandling
**Grad 2**	Kardiologisk vurdering og udredning.<br>Prednisolon 100 mg/dg med aftrapning over 4-6 uger<br>Behandling med i kardiologisk el onkologisk regi afhængig af behov for telemetri.	
**Grad 3-4**	Kardiologisk vurdering og udredning.<br>HD iv methylprednisolon 1-2 mg/kg i kardiologisk med telemetri.<br>Overvej tillæg af CellCept ved manglende effekt af prednisolon	Symptomer bedres til grad 1:Udtrapning af prednisolon over 4-6 uger<br>Vurdering v speciallæge af mulig fortsat immunterapi, evt som enkeltstofbehandling<br>Stop immunkombinationsbehandling

Side 26 af 33

Version10.07.2018

Håndtering af neuropati

Neuropatiske bivirkninger er meget sjældne, men kan være særdeles alvorlige. Ved mistanke bør neurolog konsulteres. Udredning foretages med hjælp fra neurolog og indbefatter neurologisk undersøgelse, spinal væske undersøgelse, MR scanning af cerebrum + evt hele neuroaksen, herudover EMG ved perifere neuropatier. Behandles igangsættes af onkolog.

Håndtering af neurotoksicitet

CTC neurotoksicitet	Håndtering	Followup
Foretag billeddiagnostik af CNS for at udelukke anden årsag		
**Grad 1**	Fortsæt checkpoint-hæmmere	Monitorering af pt. ved forværring, se nedenfor
**Grad 2**	Pausér checkpoint-hæmmere<br>Overvej prednisolon 25-50 mg dgl.	Genoptag checkpoint-hæmmere ved grad 0-1<br>Ved forværring, se nedenfor
**Grad 3-4 sensorisk toksicitet**	Ophør med checkpoint-hæmmere, og konferér med neurolog<br>Højdosis prednisolon 100 mg dgl.)	**Hvis symptomer bedres til <= grad 2:**Påbegynd udtrapning af steroid over 4-5 uger.<br>**Symptomer bedres ikke el. progredierer: Dosisøgning af prednisolon + overvej Cellcept**
**Grad 3-4 motorisk toksicitet**	Stop checkpoint-hæmmere, og konferér med neurolog<br>Højdosis prednisolon 100 mg dgl	**Hvis symptomer bedres til <= grad 2:**<br>Påbegynd udtrapning af prednisolon over 4-5 uger<br>**Symptomer bedres ikke el. progredierer: Dosisøgning af prednisolon + overvej Cellcept**

Håndtering af immunrelateret nefrotoksicitet

En algoritme (se næste side) for udredning og behandling af nefrotoksicitet er udviklet for patienter, som ved baseline har normalt eller let forhøjet kreatinin (≤ 1,5 x ULN).

Grad	Kreatinin
1	Stigning i kreatinin > 26 mikromol/l, eller > 1,5 x ULN
2	2-3 x ULN
3	> 3 x ULN
4	Livstruende – dialyse indiceret

Billeddannelse af urinveje bør gennemføres for at udelukke andre årsager til forhøjet *kreatinin*. Hvis en autoimmun ætiologi overvejes, konsulteres nefrolog.

Håndtering af nefrotoksicitet

CTC nefrotoksicitet	Håndtering	Followup
Udred for ikke-immunrelateret årsag til toksicitet, og behandl relevant.		
**Grad 1**	Fortsæt checkpoint-hæmmere	Monitorering af Se-kreatinin ugentlig
**Grad 2-3**	Pausér checkpoint-hæmmere<br>Konferér med nefrolog, og overvej nyrebiopsi<br>Steroid 50-100 mg prednisolon dgl.<br>Monitorér Se-kreatinin hv. 2.-3. dag	Genoptag checkpoint-hæmmere ved grad 0-1<br>**Ved forværring el. manglende bedring inden for 1 uge:**<br>Konferér med nefrolog, og overvej nyrebiopsi<br>Pausér el. stop checkpoint-hæmmere<br>Øg steroiddosis til i.v. methylprednisolon 4 mg/kg/dag<br>Genoptag checkpoint-hæmmere ved grad 0-1
**Grad 4**	Ophør med checkpoint-hæmmere<br>Konferér med nefrolog, og overvej nyrebiopsi<br>Højdosis i.v. methylprednisolon 4 mg/kg/dag<br>Monitorér Se-kreatinin hver dag	Når kliniske situation bedres skift fra iv steroid til peroral.<br>Fortsæt prednisolon til toksicitetsgrad 2, og trap herefter ud over 4-6 uger, med ugentlig blodprøve monitoreing

Side 30 af 33

Version10.07.2018

Steroidbehandling af immunrelaterede bivirkninger

Generelt
Det anbefales generelt ved ikke-hudrelaterede bivirkninger, at man overvejer at påbegynde en behandling med en middeldosis af prednisolon allerede ved grad 2-bivirkninger, f.eks 25-50 mg prednisolon og nedtrappe over 3-4 uger, når symptomerne er svundet.

Er bivirkningerne kraftigere, startes patienten typisk med 100 mg prednisolon. Når symptomerne er reduceret til grad 0-1, kan prednisolon nedtrappes langsomt over 4-5 uger med 10 mg ad gangen. Nedtrapning påbegyndes dog tidligst 1 uge efter symptombedring.

Behandlingen kan genoptages, hvis udløsende bivirkning har været maks. grad 1-2, og prednisolon er nedtrappet til 0-10 mg. Vær opmærksom på, at patienten ikke behandles med checkpoint-hæmmere, før prednisolon er trappet ned til 0-10 mg.
Substitutionsbehandling med hydrocortison forhindrer derimod ikke, at behandling fortsættes.

Ved steroidbehandling ud over 7 dage bør der gives tilskud af kalk og D-vitamin. Tabletten skal indeholde 400 mg kalk og 5-10 mikrogr. D-vitamin. To eksempler er Unikalk Plus eller Unikalk Silver. Doseres 1 tbl x 3 dgl.

Bivirkninger
De vigtigste bivirkninger ved systemisk anvendelse af glukokortikoider til immunsuppressiv terapi falder i 3 grupper:

1. Hæmning af hypothalamus-hypofyse-binyrebarkfunktionen
Behandling med glukokortikoider hæmmer hypothalamus-, hypofyse- og binyrebarkfunktionen, således at binyrebarkens egenproduktion af kortisol nedsættes, evt. ophæves. Glukokortikoidernes hæmmende virkning på hypofyse-binyrebarkfunktionen nødvendiggør nøje observation af patienten i stresssituationer, og der skal eventuelt gives substitutionsterapi som anført ovenfor.

Hæmningen af binyrebarkfunktionen er relateret til behandlingens varighed og dosisstørrelsen, men der er stor individuel variation. En nedsat funktion kan ses allerede efter 2 ugers behandling. Ca. 50 % af patienterne, der er i glukokortikoidbehandling, udviser en helt normal stigning i plasma-kortisol i forbindelse med stress, f.eks. større kirurgiske indgreb. Kun omkring 5 % reagerer ikke, og de resterende ca. 50 % udviser et subnormalt respons.

2. Iatrogen hyperkortisolæmi
De vigtigste symptomer og kliniske fund ved iatrogen hyperkorticisme omfatter osteoporose, aseptisk knoglenekrose, cushingoid fedtfordeling, atrofi af huden, striae cutis, purpura, psykiske symptomer i form af søvnløshed, rastløshed, undertiden eufori og hos disponerede patienter regulære psykoser, sløring og aktivering af mikrobielle infektioner (især tuberkulose), forværring af diabetes mellitus og hypertension, hypokaliæmi, væksthæmning hos børn, myopati, posterior subkapsulær katarakt, glaukom og efter ophør med glukokortikoidbehandlingen eventuelt sekundær binyrebarkinsufficiens.

3. Steroid pseudoreumatisme
Ovenstående består af diffuse muskel- og ledsmerter, som ikke påvirkes af nonsteroide antireumatika, endvidere psykisk instabilitet, træthed og mathed. Symptomerne ses også hos patienter, som ikke har reumatisk sygdom. Symptomkomplekset optræder dels ved en permanent og ret høj dosering af glukokortikoider og dels i forbindelse med aftrapning af glukokortikoid behandling. Symptomerne nødvendiggør en meget langsom aftrapning af behandlingen.

Vaccination i forbindelse med immunterapi

Der foreligger aktuelt ikke data der med sikkerhed kan vise om der er en risiko for negativ indflydelse på effekten af immunterapi v samtidig influenza vaccination. Heller ikke om der er en risiko for øget toxicitet ved at kombinere immunterapi og influenza vaccination?

En vaccine indeholder delkomponenter af sygdomsfremkaldende mikroorganismer. Det fremstilles ofte af:

- svækkede eller dræbte former for bakterier eller virus
- dets toksiner eller
- dets proteiner og/eller kulhydrater

Der ses forskellige grader af immunreaktion på de enkelte vacciner. De levende og svækkede, levende vacciner, antages at give de kraftigste reaktioner. Hvorvidt dette vil medføre en øget immunstimulation samtidig med den medikamentelt inducerede immunaktivering, eller starte en så kraftig reaktion, at den igangværende/ønskede reaktion mod tumor svækkes, vides ikke.

Effekt af immunterapi: Den immunreaktion der induceres ved en influenzavaccination skønnes ikke at influere negativt på effekten af immunterapien.

Toxicitet af immunterapi: Firmaet BMS har undersøgt deres database på næsten 40.000 ptt og fundet 4 tilfælde af svær immunrelateret toxicitet, hos ptt, der udover immunterapi havde fået influenzavaccination. Man fandt ikke baggrund for at konkludere at der kunne være en sammenhæng mellem den påviste toxicitet og influenzavaccinationen.

Anbefaling:
Influenzavaccination med peptid kan gives på et hvilket som helst tidspunkt i et behandlingsforløb med immunterapi, idet det vurderes, at dette ikke interfererer med den anti-tumor immunreaktion som immunterapien inducerer.

Vacciner baseret på levende og levende svækkede bakterier el vira: Der foreligger ikke videnskabelig evidens for at dette vil skade en igangværende immunterapi. Anvendelsen af disse vacciner er derfor ikke kontraindiceret. Det foreslås, at anvendelsen må drøftes individuelt med behandlingsansvarlig læge i det relevante team.

Referenceliste

1. Agarwala SS: Practical Approaches to Immunotherapy in the Clinic. Semin Oncol 42 Suppl 3:S20-7, 2015

2. Weber JS, Yang JC, Atkins MB, et al: Toxicities of Immunotherapy for the Practitioner. Journal of Clinical Oncology 33:2092-9, 2015

3. Weber JS, Kähler KC, Hauschild A: Management of Immune-Related Adverse Events and Kinetics of Response With Ipilimumab. Journal of Clinical Oncology 30:2691-2697, 2012

4. Champiat S, Lambotte O, Barreau E, et al: Management of immune checkpoint blockade dysimmune toxicities: a collaborative position paper. Ann Oncol 27:559-74, 2016

5. Naidoo J, Page DB, Li BT, et al: Toxicities of the anti-PD-1 and anti-PD-L1 immune checkpoint antibodies. Ann Oncol 26:2375-91, 2015

6. Spain L, Diem S, Larkin J: Management of toxicities of immune checkpoint inhibitors. Cancer Treat Rev 44:51-60, 2016

7. Postow MA: Managing immune checkpoint-blocking antibody side effects. Am Soc Clin Oncol Educ Book:76-83, 2015

8. Kahler KC, Hassel JC, Heinzerling L, et al: Management of side effects of immune checkpoint blockade by anti-CTLA-4 and anti-PD-1 antibodies in metastatic melanoma. J Dtsch Dermatol Ges 14:662-81, 2016

9. Villadolid J, Amin A: Immune checkpoint inhibitors in clinical practice: update on management of immune-related toxicities. Transl Lung Cancer Res 4:560-75, 2015

10. Theede K, Kiszka-Kanowitz M, Nordgaard-Lassen I, et al: [Faecal calprotectin is a useful biomarker for intestinal inflammation.]. Ugeskr Laeger 176, 2014

11. Johnson DB, Balko JM, Compton ML, et al: Fulminant Myocarditis with Combination Immune Checkpoint Blockade. New England Journal of Medicine 375:1749-1755, 2016

APPENDIX E: GENES INCLUDED IN THE TRUSIGHT™ ONCOLOGY 500 AND TRUSIGHT™ ONCOLOGY HIGH THROUGHPUT PANEL

Gene list:

ABL1, BRD4, CUX1, FAM175A, GATA6, IGF1, MAP3K13, NOTCH4, POLE, RPTOR, TAF1, ABL2, BRIP1, CXCR4, FAM46C, GEN1, IGF1R, MAP3K14, NPM1, PPARG, RUNX1, TBX3, ACVR1, BTG1, CYLD, FANCA, GID4, IGF2, MAP3K4, NRAS, PPM1D, RUNX1T1, TCEB1, ACVR1B, BTK, DAXX, FANCC, GLI1, IKBKE, MAPK1, NRG1, PPP2R1A, RYBP, TCF3, AKT1, C11orf30, DCUN1D1, FANCD2, GNA11, IKZF1, MAPK3, NSD1, PPP2R2A, SDHA, TCF7L2, AKT2, CALR, DDR2, FANCE, GNA13, IL10, MAX, NTRK1, PPP6C, SDHAF2, TERC, AKT3, CARD11, DDX41, FANCF, GNAQ, IL7R, MCL1, NTRK2, PRDM1, SDHB, TERT, ALK, CASP8, DHX15, FANCG, GNAS, INHA, MDC1, NTRK3, PREX2, SDHC, TET1, ALOX12B, CBFB, DICER1, FANCI, GPR124, INHBA, MDM2, NUP93, PRKAR1A, SDHD, TET2,ANKRD11, CBL, DIS3, FANCL, GPS2, INPP4A, MDM4, NUTM1, PRKCI, SETBP1, TFE3,ANKRD26, CCND1, DNAJB1, FAS, GREM1, INPP4B, MED12, PAK1, PRKDC, SETD2, TFRC,APC, CCND2, DNMT1, FAT1, GRIN2A, INSR, MEF2B, PAK3, PRSS8, SF3B1, TGFBR1,AR, CCND3, DNMT3A, FBXW7, GRM3, IRF2, MEN1, PAK7, PTCH1, SH2B3, TGFBR2,ARAF, CCNE1, DNMT3B, FGF1, GSK3B, IRF4, MET, PALB2, PTEN, SH2D1A, TMEM127,ARFRP1, CD274, DOT1L, FGF10, H3F3A, IRS1, MGA, PARK2, PTPN11, SHQ1, TMPRSS2, ARID1A, CD276, E2F3, FGF14, H3F3B, IRS2, MITF, PARP1, PTPRD, SLIT2, TNFAIP3, ARID1B, CD74, EED, FGF19, H3F3C, JAK1, MLH1, PAX3, PTPRS, SLX4, TNFRSF14,ARID2, CD79A, EGFL7, FGF2, HGF, JAK2, MLL, PAX5, PTPRT, SMAD2, TOP1, ARID5B, CD79B, EGFR, FGF23, HIST1H1C, JAK3, MLLT3, PAX7, QKI, SMAD3, TOP2A, ASXL1, CDC73, EIF1AX, FGF3, HIST1H2BD, JUN, MPL, PAX8, RAB35, SMAD4, TP53, ASXL2, CDH1, EIF4A2, FGF4, HIST1H3A, KAT6A, MRE11A, PBRM1, RAC1, SMARCA4, TP63, ATM, CDK12, EIF4E, FGF5, HIST1H3B, KDM5A, MSH2, PDCD1, RAD21, SMARCB1, TRAF2, ATR, CDK4, EML4, FGF6, HIST1H3C, KDM5C, MSH3, PDCD1LG2, RAD50, SMARCD1, TRAF7, ATRX, CDK6, EP300, FGF7, HIST1H3D, KDM6A, MSH6, PDGFRA, RAD51, SMC1A, TSC1, AURKA, CDK8, EPCAM, FGF8, HIST1H3E, KDR, MST1, PDGFRB, RAD51B, SMC3, TSC2, AURKB, CDKN1A, EPHA3, FGF9, HIST1H3F, KEAP1, MST1R, PDK1, RAD51C, SMO, TSHR, AXIN1, CDKN1B, EPHA5, FGFR1, HIST1H3G, KEL, MTOR, PDPK1, RAD51D, SNCAIP, U2AF1, AXIN2, CDKN2A, EPHA7, FGFR2, HIST1H3H, KIF5B, MUTYH, PGR, RAD52, SOCS1, VEGFA, AXL, CDKN2B, EPHB1, FGFR3, HIST1H3I, KIT, MYB, PHF6, RAD54L, SOX10, VHL, B2M, CDKN2C, ERBB2, FGFR4, HIST1H3J, KLF4, MYC, PHOX2B, RAF1, SOX17, VTCN, BAP1, CEBPA, ERBB3, FH, HIST2H3A, KLHL6, MYCL1, PIK3C2B, RANBP2, SOX2, WISP3, BARD1, CENPA, ERBB4, FLCN, HIST2H3C, KMT2B, MYCN, PIK3C2G, RARA, SOX9, WT1,BBC3, CHD2, ERCC1, FLI1, HIST2H3D, KMT2C, MYD88, PIK3C3, RASA1, SPEN, XIAP,BCL10, CHD4, ERCC2, FLT1, HIST3H3, KMT2D, MYOD1, PIK3CA, RB1, SPOP, XPO1,BCL2, CHEK1, ERCC3, FLT3, HLA-A, KRAS, NAB2, PIK3CB, RBM10, SPTA1, XRCC2,BCL2L1, CHEK2, ERCC4, FLT4, HLA-B, LAMP1, NBN, PIK3CD, RECQL4, SRC, YAP1,BCL2L11, CIC, ERCC5, FOXA1, HLA-C, LATS1, NCOA3, PIK3CG, REL, SRSF2, YES1,BCL2L2, CREBBP, ERG, FOXL2, HNF1A, LATS2, NCOR1, PIK3R1, RET, STAG1, ZBTB2,BCL6, CRKL, ERRFI1, FOXO1, HNRNPK, LMO1, NEGR1, PIK3R2, RFWD2, STAG2, ZBTB7A, BCOR, CRLF2, ESR1, FOXP1, HOXB13, LRP1B, NF1, PIK3R3, RHEB, STAT3, ZFHX3, BCORL1, CSF1R, ETS1, FRS2, HRAS, LYN, NF2, PIM1, RHOA, STAT4, ZNF217, BCR, CSF3R, ETV1, FUBP1, HSD3B1, LZTR1, NFE2L2, PLCG2, RICTOR, STAT5A, ZNF703, BIRC3, CSNK1A1, ETV4, FYN, HSP90AA1, MAGI2, NFKBIA, PLK2, RIT1, STAT5B, ZRSR2, BLM, CTCF, ETV5, GABRA6, ICOSLG, MALT1, NKX2-1, PMAIP1, RNF43, STK11, BMPR1A, CTLA4, ETV6, GATA1, ID3, MAP2K1, NKX3-1, PMS1, ROS1, STK40, BRAF, CTNNA1, EWSR1, GATA2, IDH1, MAP2K2, NOTCH1, PMS2, RPS6KA4, SUFU, BRCA1, CTNNB1, EZH2, GATA3, IDH2, MAP2K4, NOTCH2, PNRC1, RPS6KB1, SUZ12, BRCA2, CUL3, FAM123B, GATA4, IFNGR1, MAP3K1, NOTCH3, POLD1, RPS6KB2, SYK
